# Supplementary material for: Kingdom-wide comparison reveals the evolution of diurnal gene expression in Archaeplastida
Source: Nat Commun. 2019 Feb 13;10:737. doi: 10.1038/s41467-019-08703-2 (PMC6374488; doi:10.1038/s41467-019-08703-2)
Supplement: Supplementary file 1 — Supplementary Information [file 41467_2019_8703_MOESM1_ESM.pdf]

Kingdom-wide comparison reveals the evolution of diurnal gene expression in Archaeplastida

Ferrari *et al*.

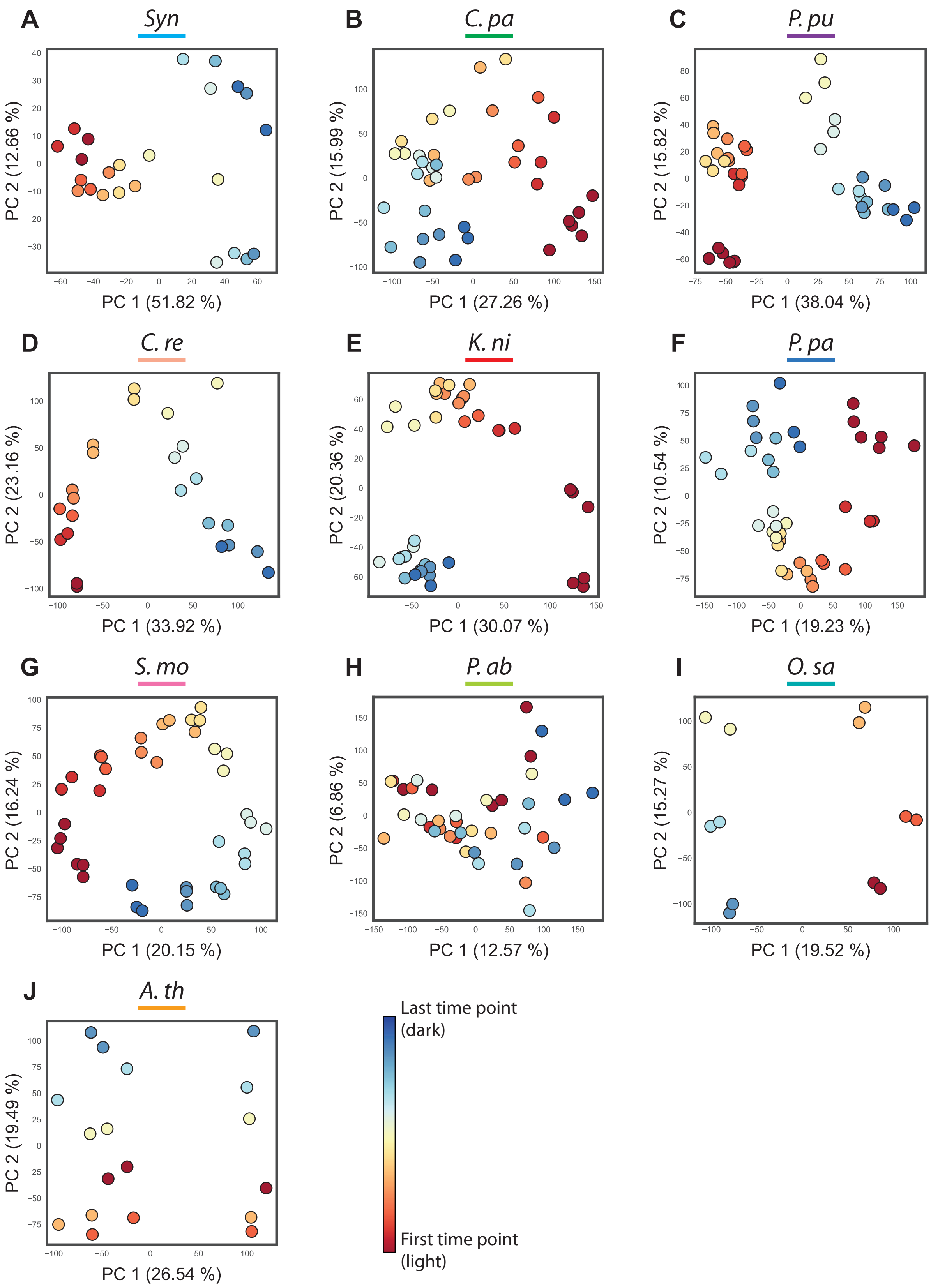

Supplementary Fig. 1. PCA analysis of the expression data. Each dot represents a sample. The dots are color-coded based on the time of the sampling, with dark red being the first sample after the light was turned on.

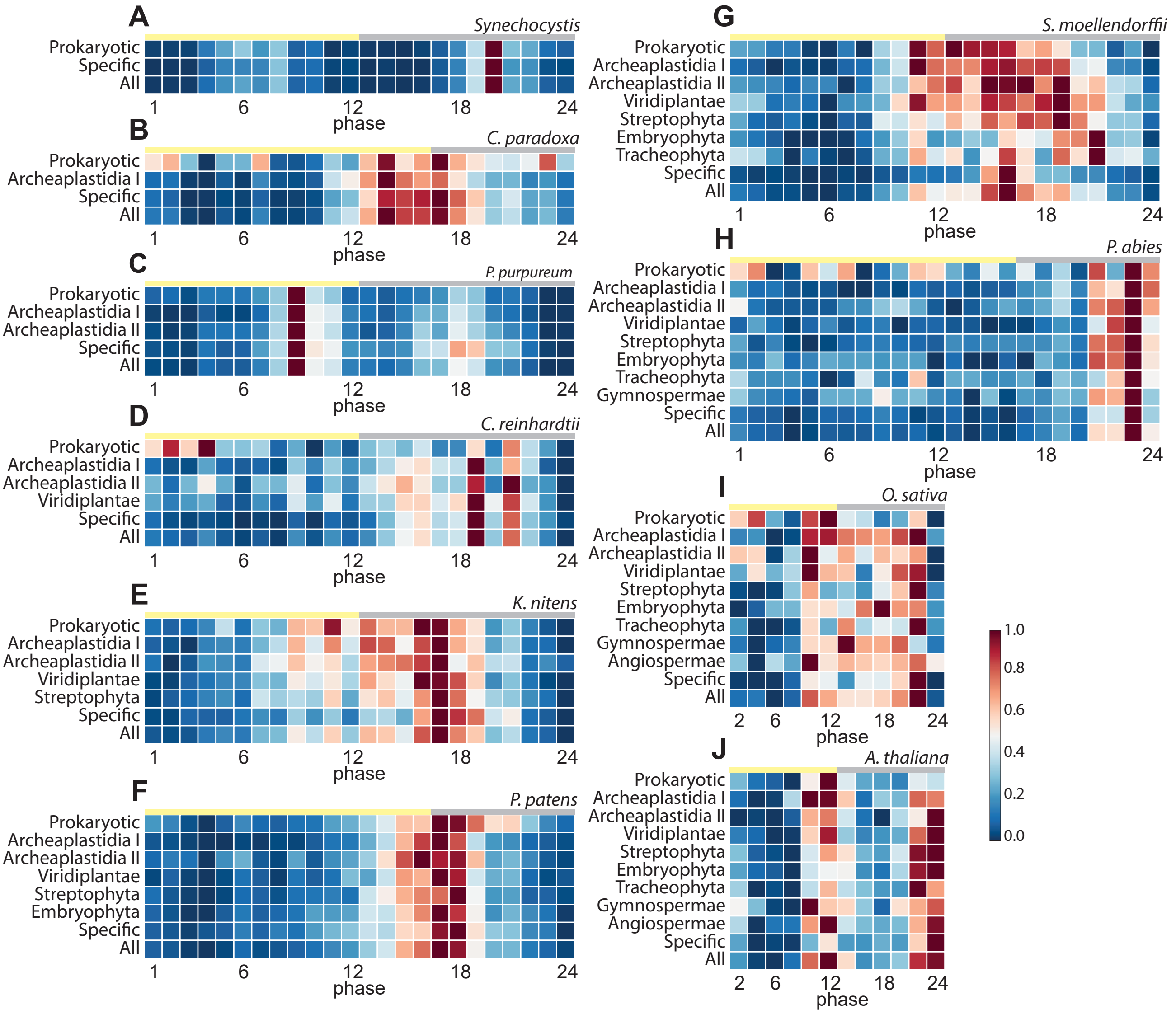

Supplementary Fig. 2. Distribution of expression peaks of rhythmic genes. The genes are divided by the phylostrata (rows), and the expression peaks are plotted through the phases (columns). The color of the cells indicates the 0-1-scaled gene expression values, where 0 and 1 indicate the lowest and highest expression, respectively.

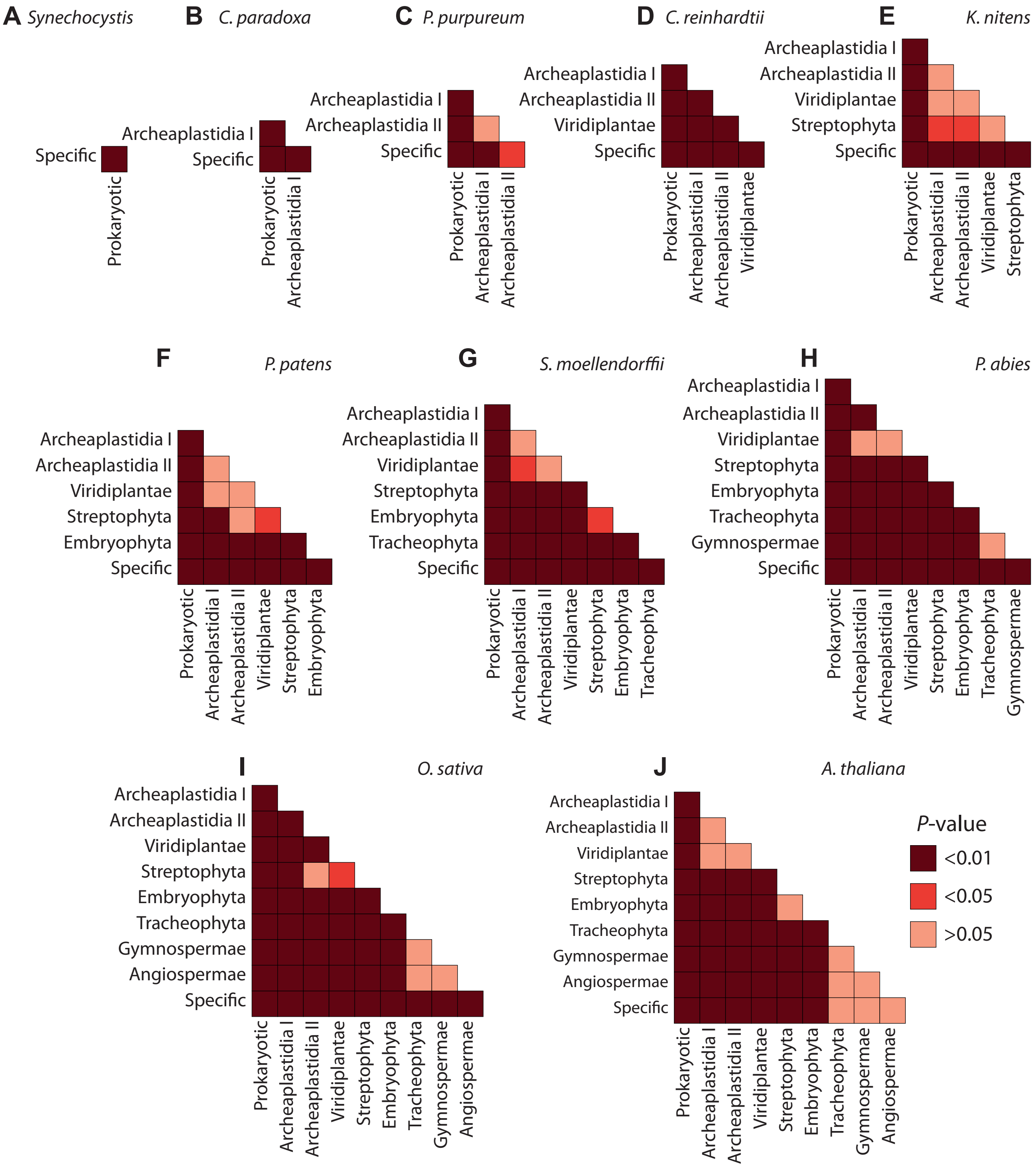

Supplementary Fig. 3. Differential analysis of average expression of phylostrata. The cell colors indicate the two-sample Kolmogorov–Smirnov comparison *p*-value (K–S test, FDR corrected) for all possible phylostrata combinations for average expression distribution.

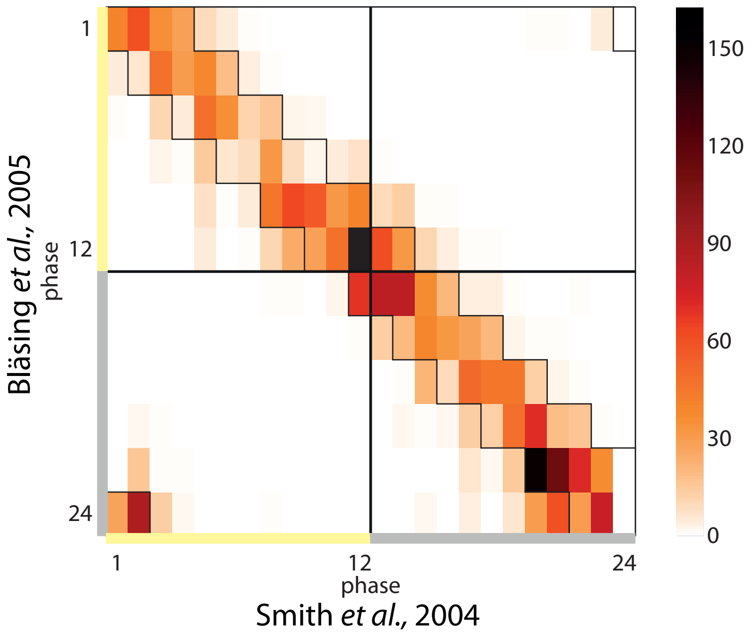

Supplementary Fig. 4. Diurnal transcriptomes comparison of two independent *A. thaliana* experiments. The color intensity of the cells indicates the number of genes that peak at a given phase combination in the two experiments. Black lines indicate a transition from light to dark, while the zigzag lines indicate an interval of 4 hours.

Blasing, O. E. Sugars and Circadian Regulation Make Major Contributions to the Global Regulation of Diurnal Gene Expression in Arabidopsis. *Plant Cell Online* **17,** 3257–3281 (2005)

Smith SM, Fulton DC, Chia T, Thorneycroft D, Chapple A, et al. Diurnal Changes in the Transcriptome Encoding Enzymes of Starch Metabolism Provide Evidence for Both Transcriptional and Posttranscriptional Regulation of Starch Metabolism in Arabidopsis Leaves. *Plant Physiology*. 136: 2687-99 (2004)

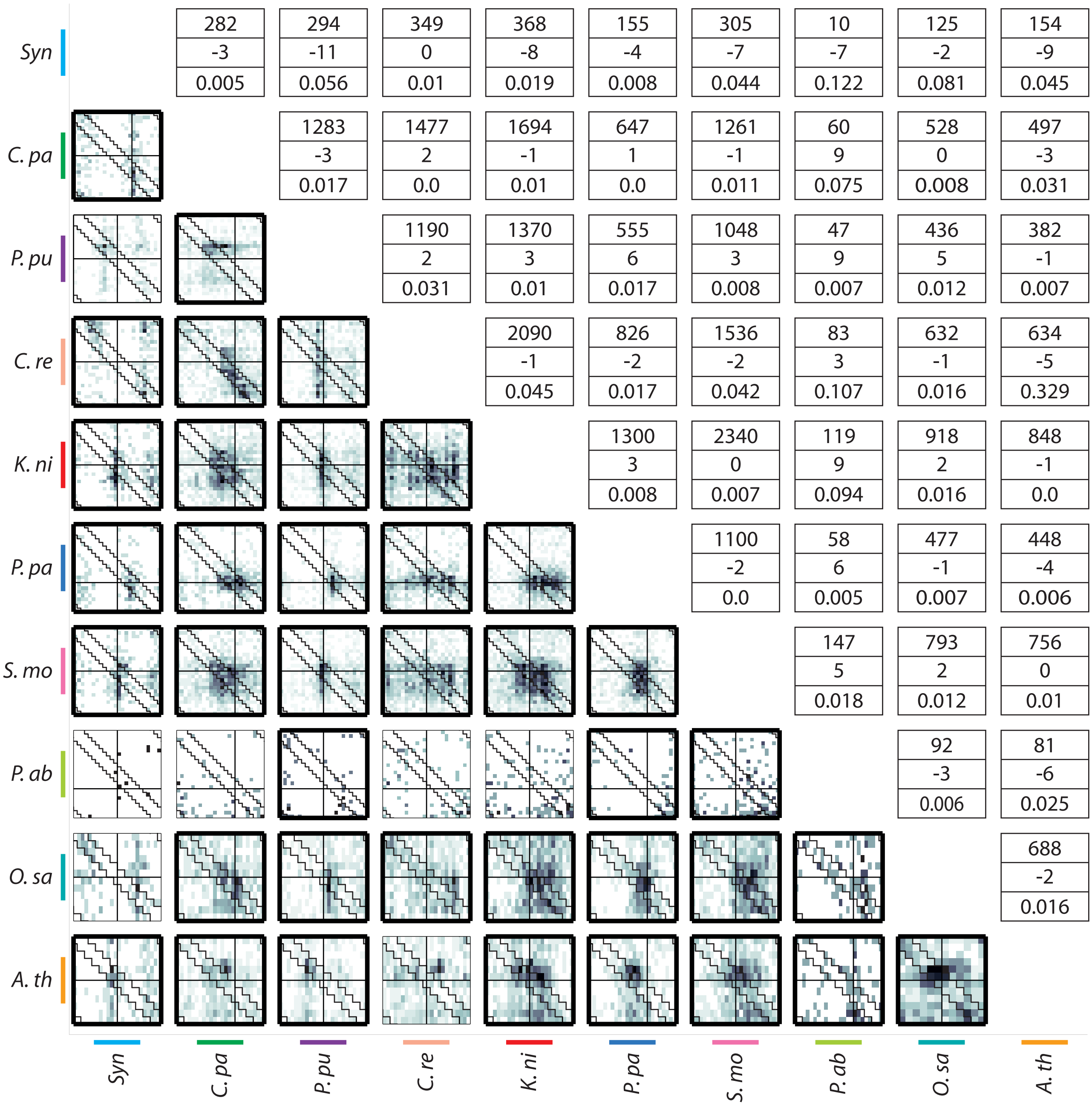

Syn
C. pa
P. pu
C. re
K. ni
P. pa
S. mo
P. ab
O. sa
A. th
282 -3 0.005
294 -11 0.056
349 0 0.01
368 -8 0.019
155 -4 0.008
305 -7 0.044
10 -7 0.122
125 -2 0.081
154 -9 0.045
1283 -3 0.017
1477 2 0.0
1694 -1 0.01
647 1 0.0
1261 -1 0.011
60 9 0.075
528 0 0.008
497 -3 0.031
1190 2 0.031
1370 3 0.01
555 6 0.017
1048 3 0.008
47 9 0.007
436 5 0.012
382 -1 0.007
2090 -1 0.045
826 -2 0.017
1536 -2 0.042
83 3 0.107
632 -1 0.016
634 -5 0.329
1300 3 0.008
2340 0 0.007
119 9 0.094
918 2 0.016
848 -1 0.0
1100 -2 0.0
58 6 0.005
477 -1 0.007
448 -4 0.006
147 5 0.018
793 2 0.012
756 0 0.01
92 -3 0.006
81 -6 0.025
688 -2 0.016
Syn
C. pa
P. pu
C. re
K. ni
P. pa
S. mo
P. ab
O. sa
A. th

Supplementary Fig. 5. Comparison of shifted diurnal transcriptomes. The heatmaps indicate the phase comparisons of orthologs of all possible species combination after the shift is applied to one of the two species (x-axis). The color intensity of the cells indicates the number of orthologs that peak at a given phase combination in the two species. Black lines indicate a transition from light to dark, while the zigzag lines indicate phase differences within ±*Δ*2hours between the two species. The thick black frames of the heatmaps indicate which species combination has a significantly similar $\Delta phase_{expected}$ value (FDR corrected empirical *p*-value < 0.05). The thin frames indicate combinations which are not significantly similar. The upper right triangle shows, from top to bottom, the number of orthologs included in the analysis, the shift and the empirical *p*-value (FDR corrected), respectively.

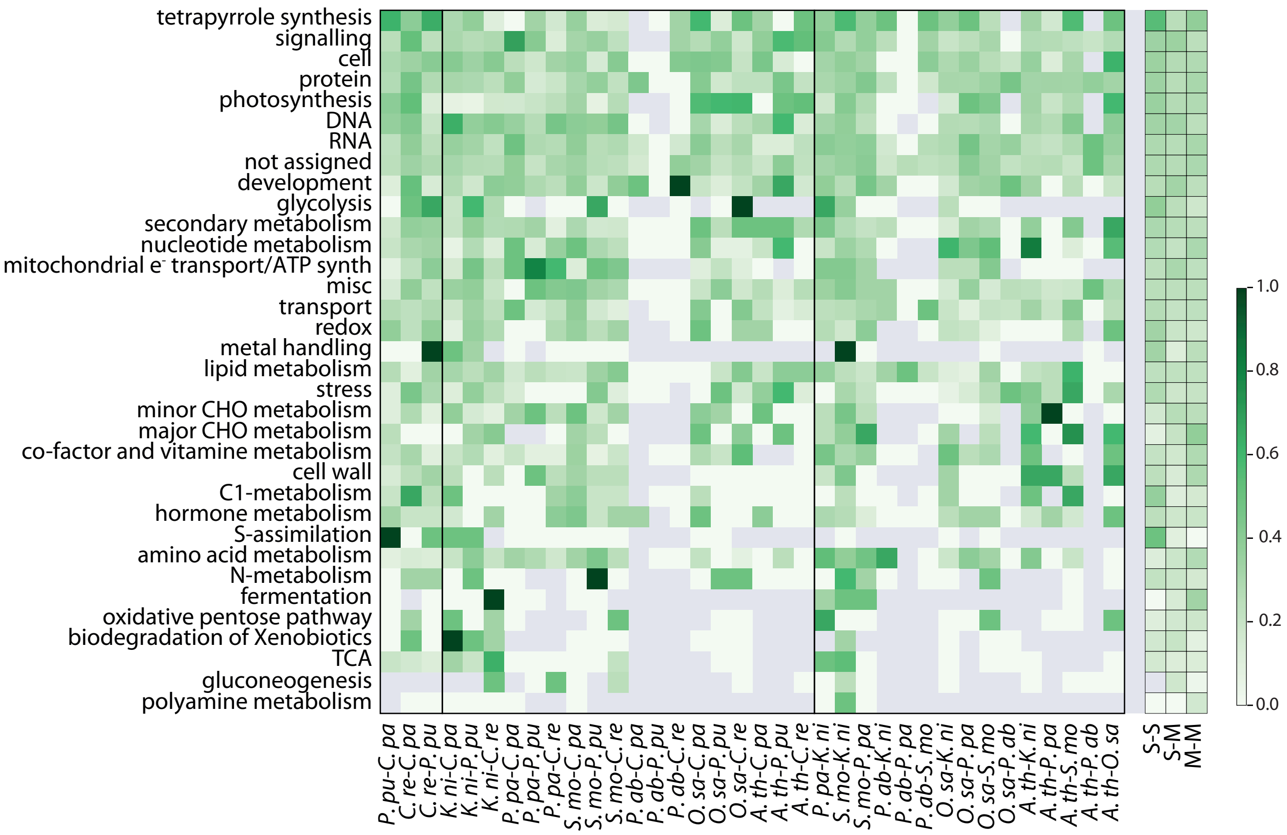

Supplementary Fig. 6. Similarity of the phases of the rhythmic genes in the different biological processes and species. The heatmap shows the fraction of rhythmic orthologs assigned to a specific biological process, that peak within ±2 hours of each other. For example, value of 0.6 would indicate that 60% of orthologs assigned to a given process peak at similar time during the day. The organisms are grouped by organismal complexity: single cellular-single cellular (S-S), single cellular-multicellular (S-M) and multicellular-multicellular (M-M). The last three columns indicate the average expression of the three groups. Biological processes are sorted from the most to the least abundant.

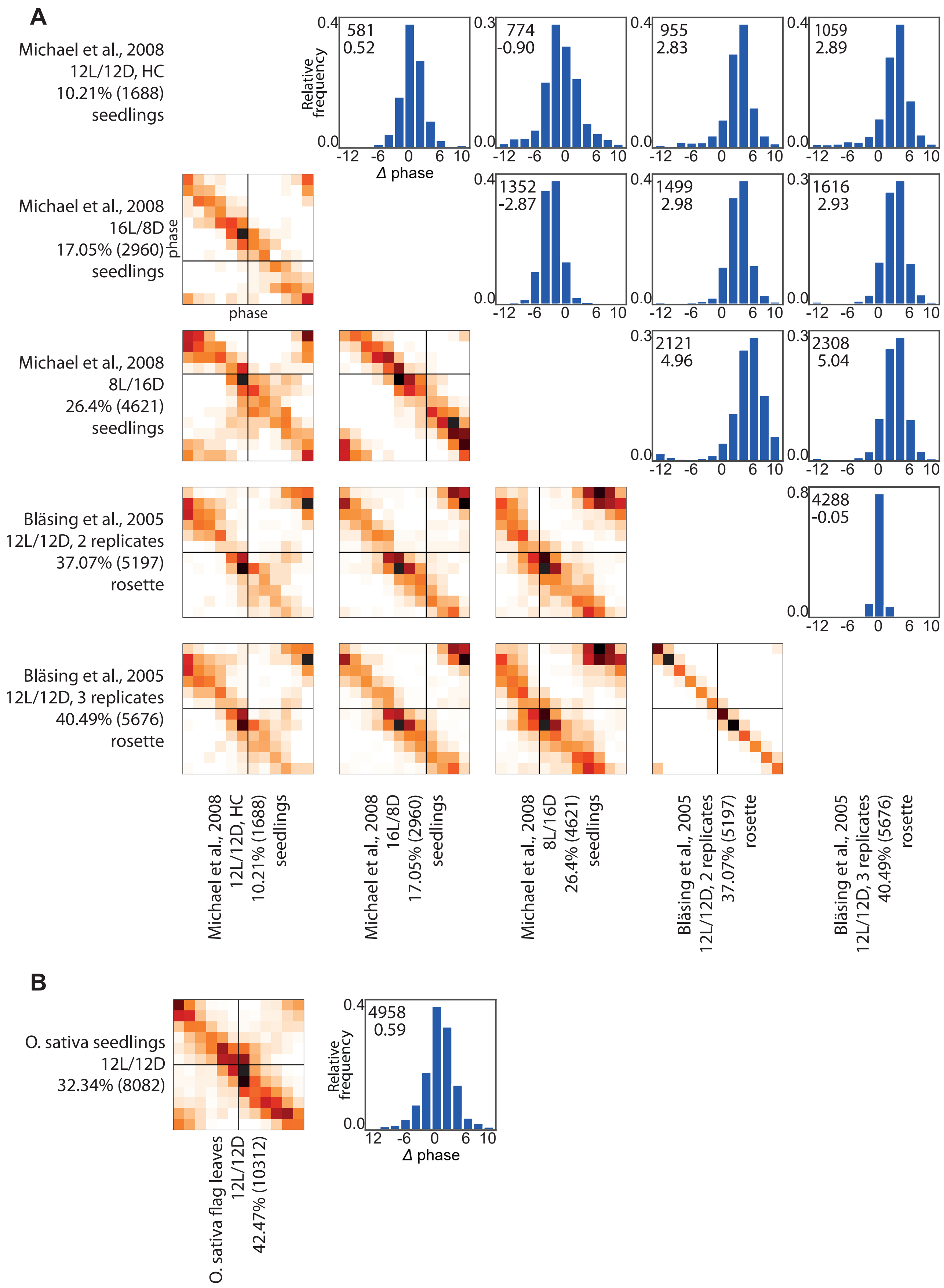

A
Michael et al., 2008
12L/12D, HC
10.21% (1688)
seedlings
Michael et al., 2008
16L/8D
17.05% (2960)
seedlings
Michael et al., 2008
8L/16D
26.4% (4621)
seedlings
Bläsing et al., 2005
12L/12D, 2 replicates
37.07% (5197)
rosette
Bläsing et al., 2005
12L/12D, 3 replicates
40.49% (5676)
rosette
Relative frequency
Δ phase
phase
581
0.52
774
-0.90
955
2.83
1059
2.89
1352
-2.87
1499
2.98
1616
2.93
2121
4.96
2308
5.04
4288
-0.05
B
O. sativa seedlings
12L/12D
32.34% (8082)
O. sativa flag leaves
12L/12D
42.47% (10312)
4958
0.59

Supplementary Fig. 7. Comparison analysis of diurnal expression of different developmental stages in *O. sativa* and *A. thaliana*. The heatmaps indicate the phase comparisons of genes in the different datasets. The color intensity of the cells indicates the number of genes that peak at a given phase combination in the two datasets. Black lines indicate a transition from light to dark. The percentages indicate the rhythmic genes identified in the different datasets. The histograms show the relative frequency of phase differences between genes in the different datasets. The upper number indicates genes which were identified as rhythmic in both datasets while the lower number indicates the average *Δ*phase. A) Four different datasets were compared for *A. thaliana* 12L/12D, 22°C/12°C; 16L/8D; 8L/16D; 12L/12D 22°C/22°C with two and three replicates. B) Two different datasets were compared for *O. sativa*; seedlings and flag leaves.

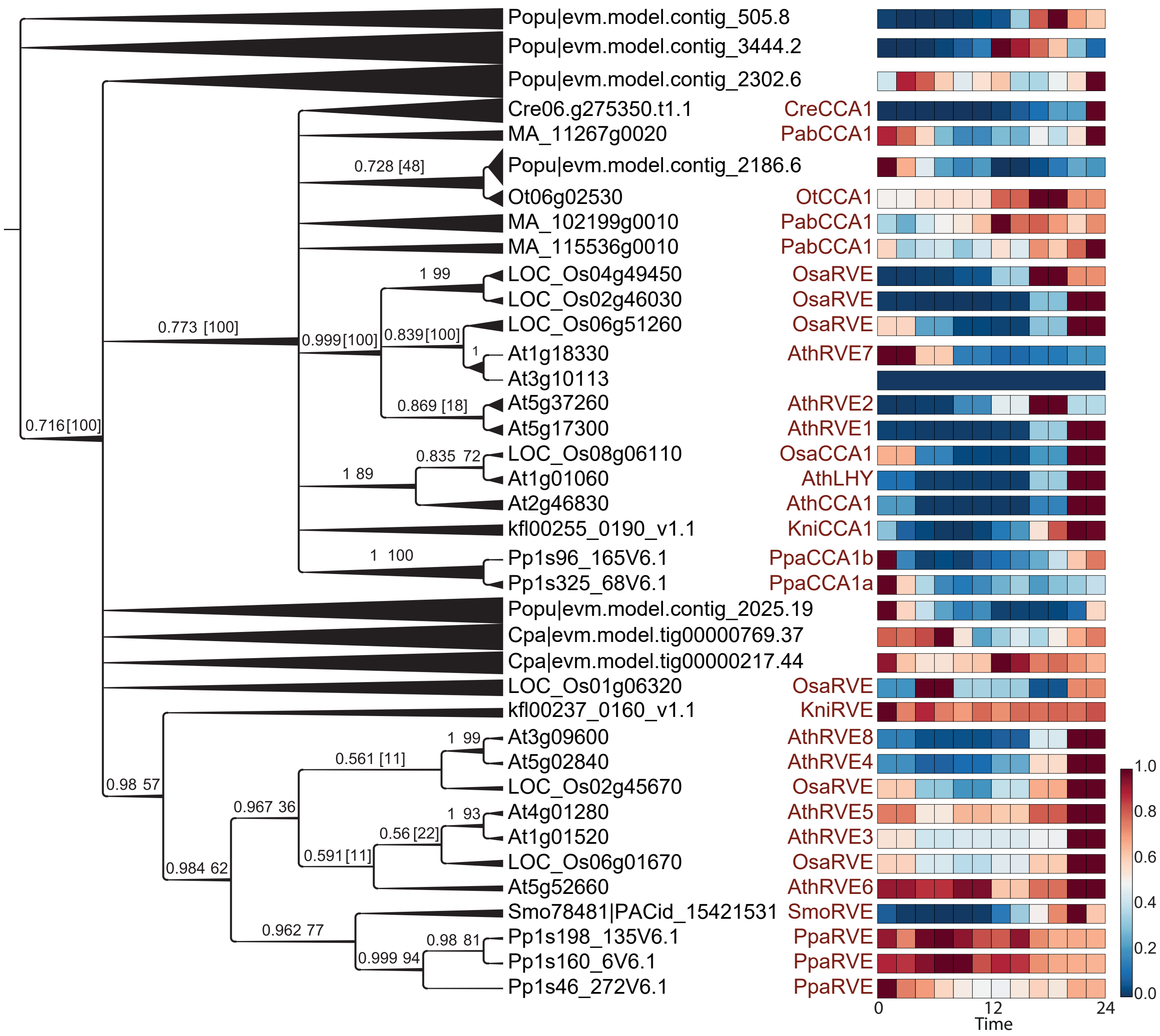

Supplementary Fig. 8. Inferred phylogenetic tree for *CCA1/LHY/RVEs*. The phylogenetic tree was constructed based on Bayesian inference of phylogeny, and the bootstrap values were estimated on Maximum-Likelihood phylogeny of the orthogroup containing *A. thaliana CCA1/LHY* and *RVE* 1-8. The gene annotation was based on[69]. The single heatmaps represent the 0-1-scaled expression profiles of each gene in each time point.

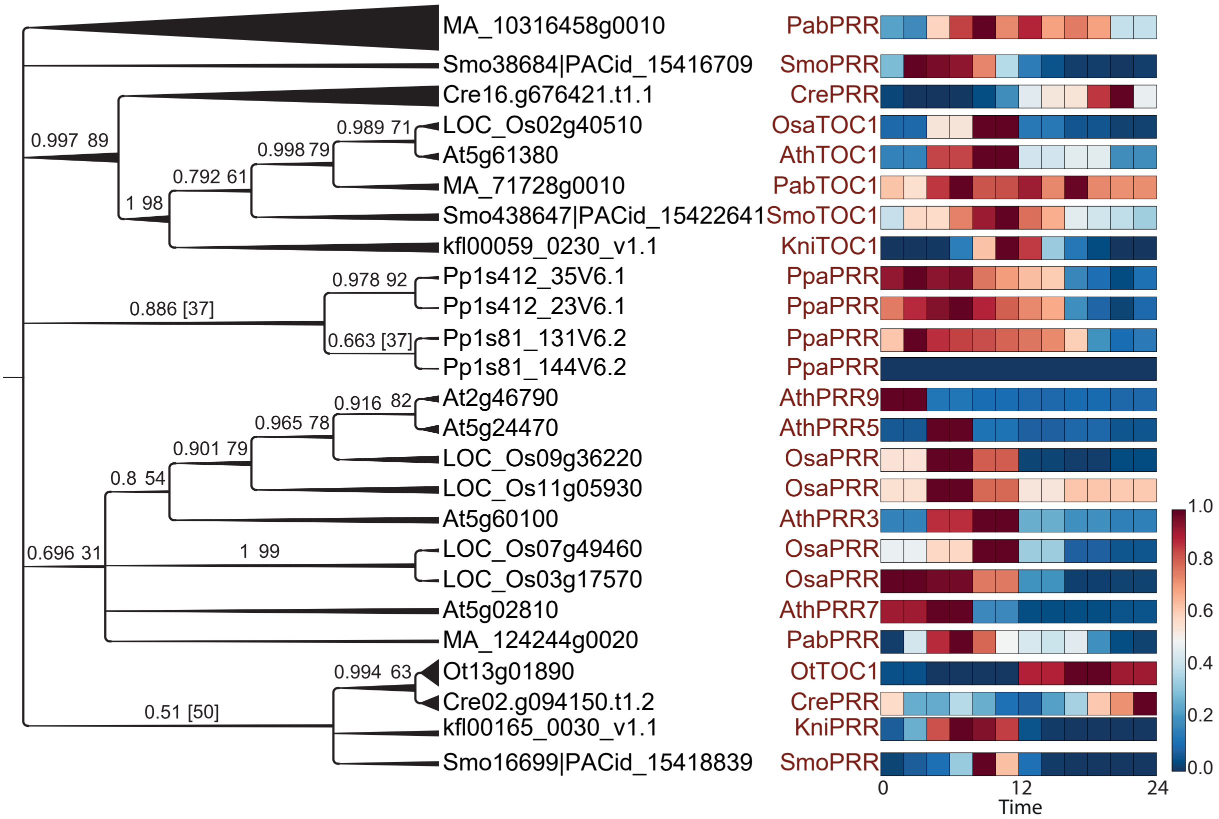

Supplementary Fig. 9. Inferred phylogenetic tree for *PRRs*. The phylogenetic trees were constructed based on the identification of the orthogroup containing *A. thaliana PRR1 (TOC1), 3, 7, 5, 9*.

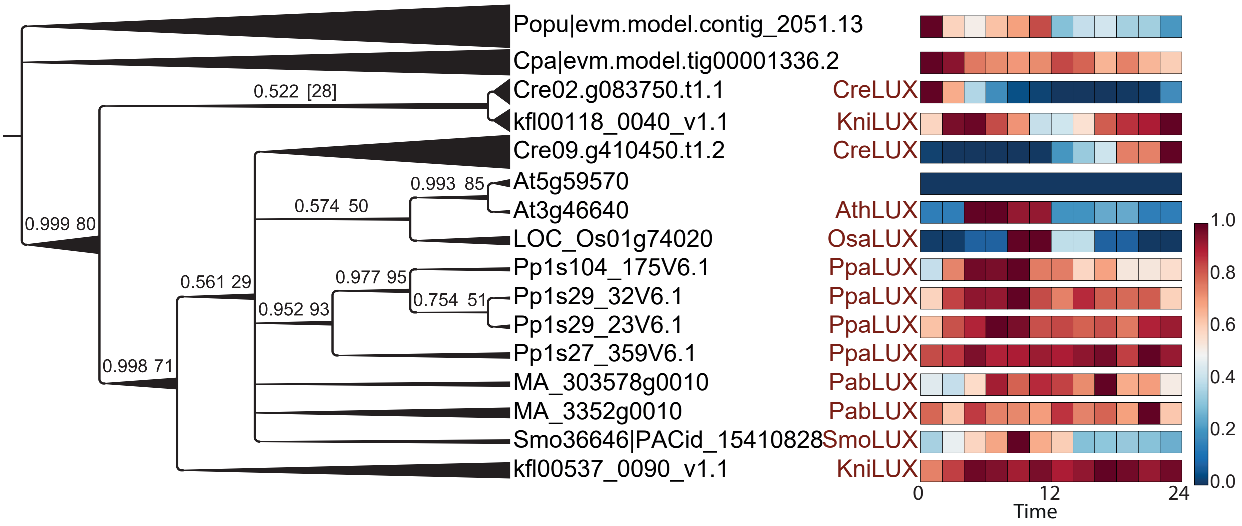

Supplementary Fig. 10. Inferred phylogenetic tree for *LUX*. The phylogenetic trees were constructed based on the identification of the orthogroup containing *A. thaliana LUX*.

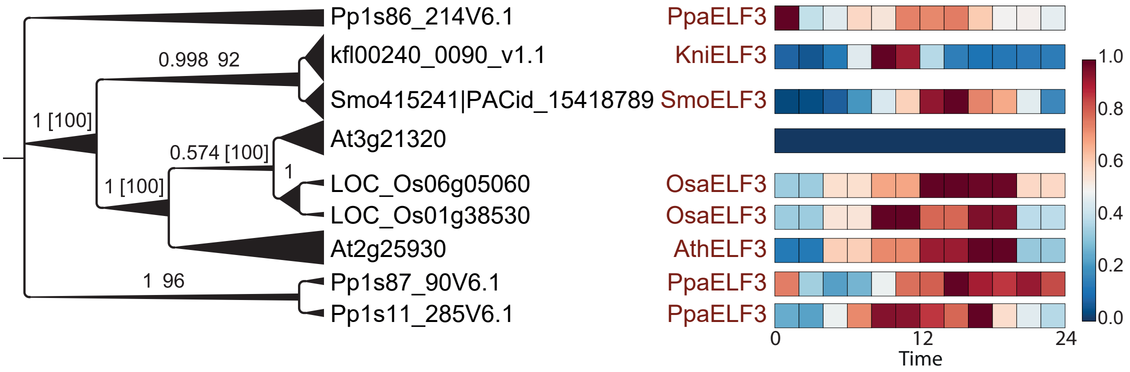

Supplementary Fig. 11. Inferred phylogenetic tree for *ELF3*. The phylogenetic trees were constructed based on the identification of the orthogroup containing *A. thaliana ELF3*.

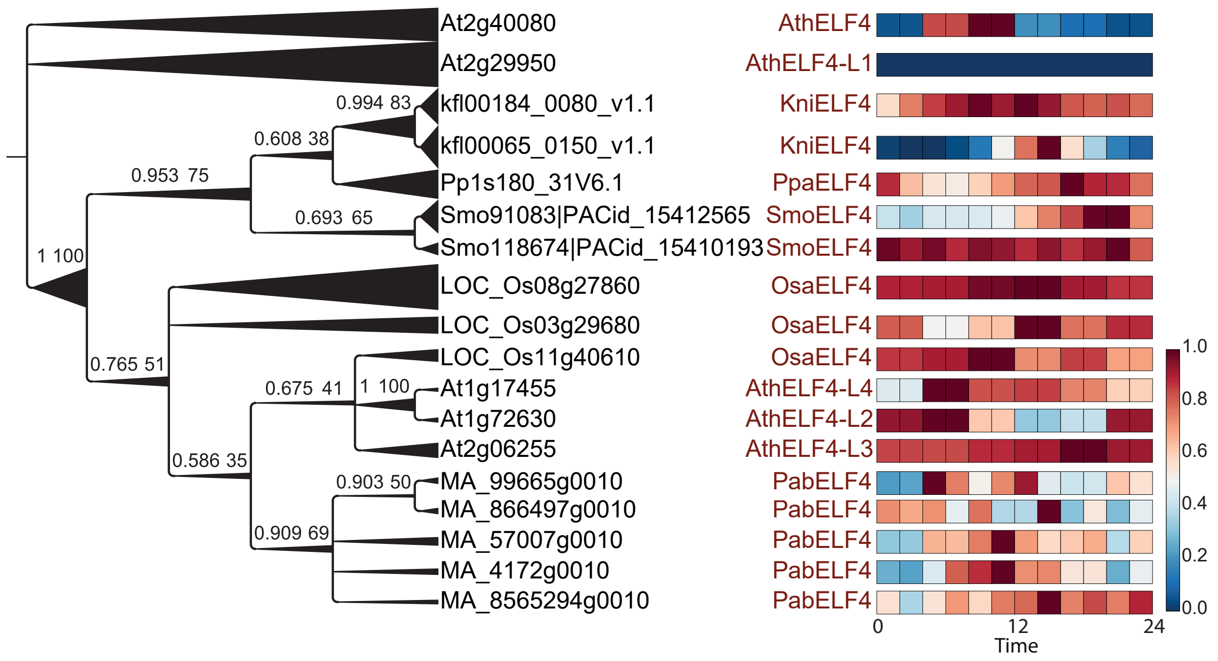

Supplementary Fig. 12. Inferred phylogenetic tree for *ELF4*. The phylogenetic trees were constructed based on the identification of the orthogroup containing *A. thaliana ELF4*.

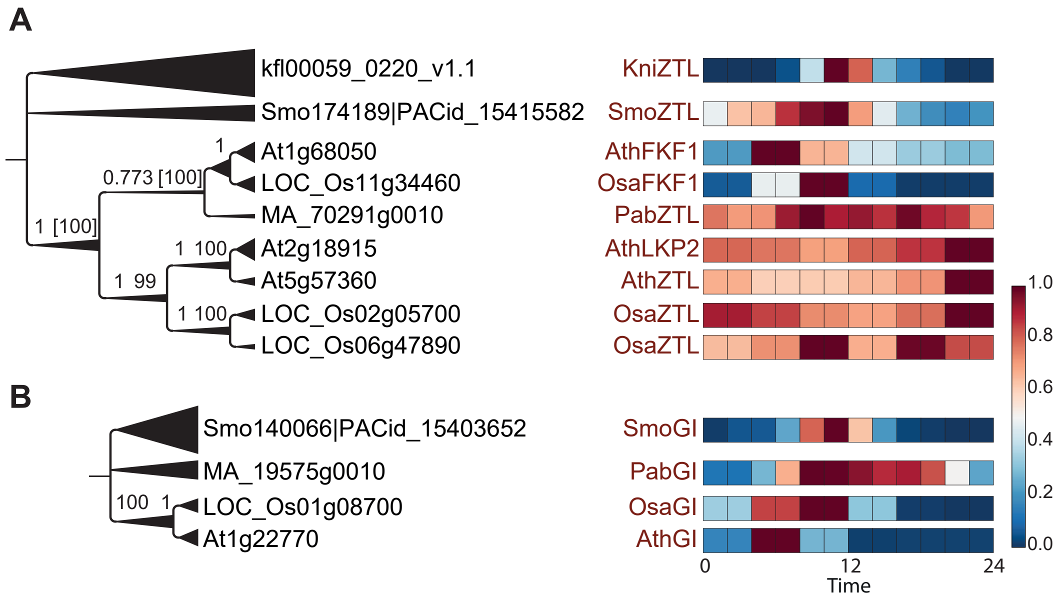

Supplementary Fig. 13. Inferred phylogenetic tree for *ZTL* and *GI*. The phylogenetic trees were constructed based on the identification of the orthogroups containing *A. thaliana* (A) *ZTL* and (B) *GI*.

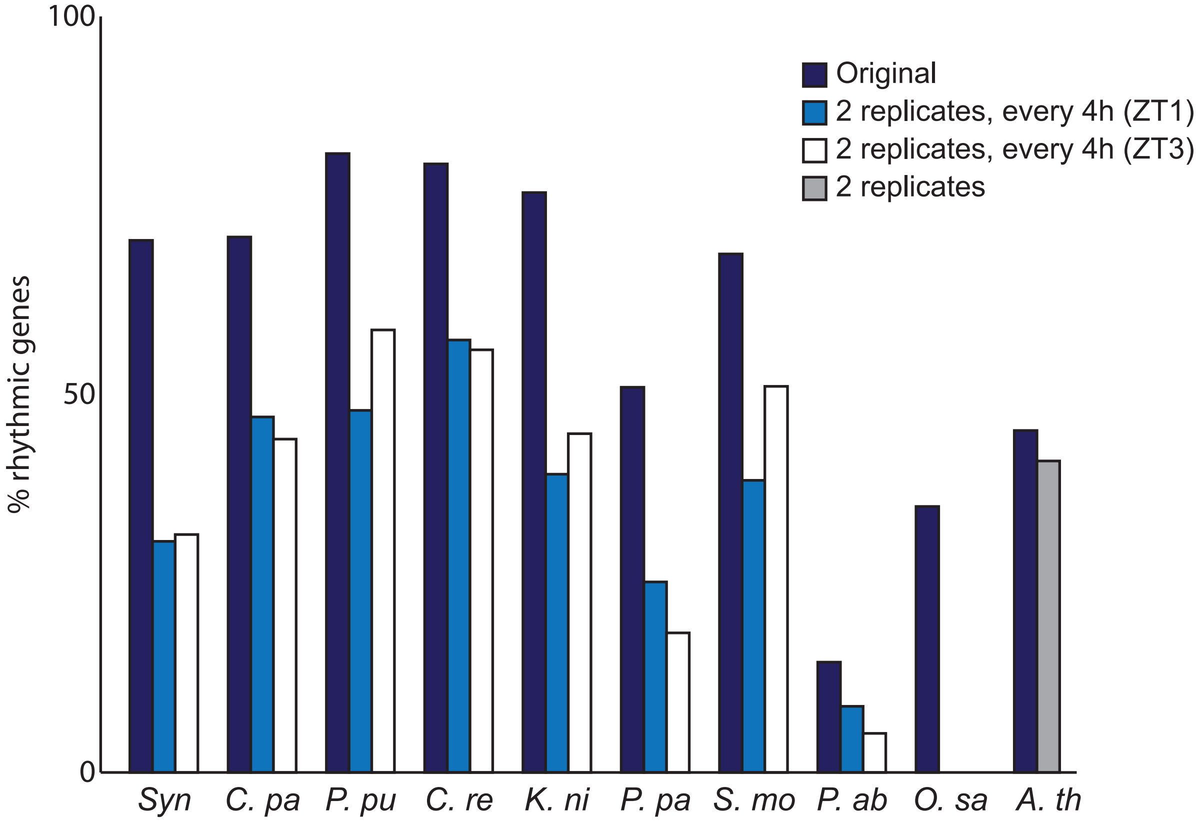

Supplementary Fig. 14. Influence of sample density on the percentage of detected rhythmic genes. The x- and y-axis show the species and the percentage of rhythmic genes in a species, at a given dataset. Bars represent percentages of rhythmic genes identified by JTK algorithm. Dark blue bars are calculated on the original datasets (sampled every 2 hours, in 3 replicates, 2 for *Synechocystis* sp. PCC 6803 and *C. reinhardtii*), light blue bars represent same datasets, but with 2 replicates, 6 time points and starting at ZT1, while white bars represent the datasets with 2 replicates, 6 time points starting at ZT3, the gray bar is calculated on the *A. thaliana* adapted dataset with 2 replicates.

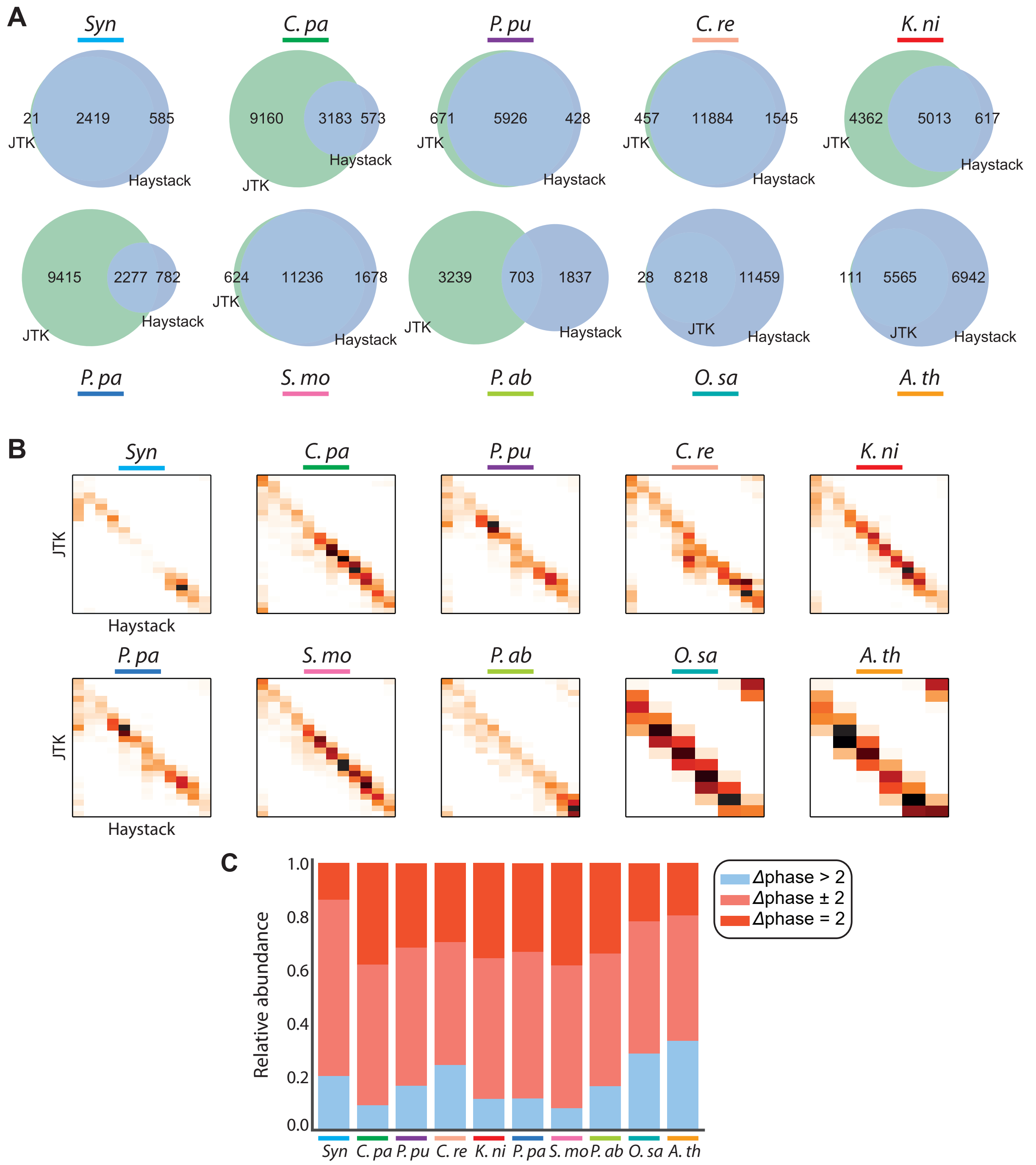

Supplementary Fig. 15. Comparison of JTK and Haystack algorithms for rhythmic genes detection. A) Venn Diagrams representing genes identified as rhythmic by JTK (green) and Haystack (blue). B) Phase comparison of rhythmic genes identified by JTK (y-axis) and Haystack (x-axis). C) Stacked bars showing the percentages of rhythmic genes assigned to the same phase (dark red), to phases whose *abs*(*Δ*phase) <= 2 (pink) or *abs*(*Δ*phase) > 2 (light blue) by the two algorithms.
