# Supplementary material for: Kingdom-wide comparison reveals the evolution of diurnal gene expression in Archaeplastida
Source: Nat Commun. 2019 Feb 13;10:737. doi: 10.1038/s41467-019-08703-2 (PMC6374488; doi:10.1038/s41467-019-08703-2)
Supplement: Supplementary file 3 — Description of Additional Supplementary Files [file 41467_2019_8703_MOESM3_ESM.pdf]

Description of Supplementary Data Files:

Supplementary Data 1. LSTrAP results. Percentages of reads mapped to the genome, counts of reads mapped to coding genes (“mapped”), mapped to non coding genes (“no-feature”) and not uniquely (“ambiguous”) with relative percentages.

Supplementary Data 2. *Synechocystis* sp. PCC 6803 data table containing gene name, phylostratum, JTK adjusted *p*-value, JTK lag, mercator annotation and TPM (RMA) diurnal expression values.

Supplementary Data 3. *C. paradoxa* data table containing gene name, phylostratum, JTK adjusted *p*-value, JTK lag, mercator annotation and TPM (RMA) diurnal expression values.

Supplementary Data 4. *P. purpureum* data table containing gene name, phylostratum, JTK adjusted *p*-value, JTK lag, mercator annotation and TPM (RMA) diurnal expression values.

Supplementary Data 5. *C. reinhardtii* data table containing gene name, phylostratum, JTK adjusted *p*-value, JTK lag, mercator annotation and TPM (RMA) diurnal expression values.

Supplementary Data 6. *K. nitens* data table containing gene name, phylostratum, JTK adjusted *p*-value, JTK lag, mercator annotation and TPM (RMA) diurnal expression values.

Supplementary Data 7. *P. patens* data table containing gene name, phylostratum, JTK adjusted *p*-value, JTK lag, mercator annotation and TPM (RMA) diurnal expression values.

Supplementary Data 8. *S. moellendorffii* data table containing gene name, phylostratum, JTK adjusted *p*-value, JTK lag, mercator annotation and TPM (RMA) diurnal expression values.

Supplementary Data 9. *P. abies* data table containing gene name, phylostratum, JTK adjusted *p*-value, JTK lag, mercator annotation and TPM (RMA) diurnal expression values.

Supplementary Data 10. *O. sativa* data table containing gene name, phylostratum, JTK adjusted *p*-value, JTK lag, mercator annotation and TPM (RMA) diurnal expression values.

Supplementary Data 11. *A. thaliana* data table containing gene name, phylostratum, JTK adjusted *p*-value, JTK lag, mercator annotation and TPM (RMA) diurnal expression values.

Supplementary Data 12. Age of gene families. The first column indicates the oldest and the youngest species present in the families, the second column contains the number of the respective gene families while the third column indicates the gene families.

Supplementary Data 13. Conserved orthologs. The first column contains the species pair, the second column contains the percentage of orthologs found in the diagonal of the phase comparison heatmap (rhythmic orthologs that peak within ±2 hours of each other), the third column shows the percentage of orthologs found in the diagonal of the heatmap after the shift. The fourth column indicates the adjusted *p*-value indicating the significance of the similarity. The fifth and sixth columns indicate the average $\Delta phase_{observed}$ before and after the shift respectively.

Supplementary Data 14. OrthoFinder output containing the gene families.
Supplementary Data 15. Protein alignments for major clock components.
