# Supplementary material for: Kingdom-wide comparison reveals the evolution of diurnal gene expression in Archaeplastida
Source: Nat Commun. 2019 Feb 13;10:737. doi: 10.1038/s41467-019-08703-2 (PMC6374488; doi:10.1038/s41467-019-08703-2)
Supplement: Supplementary file 18 — Supplementary Data 15 [file 41467_2019_8703_MOESM18_ESM.pdf]

```
T-COFFEE, Version_11.00.d625267 (2016-01-11 15:25:41 - Revision d625267 - Build 507)
Cedric Notredame
CPU TIME:0 sec.
SCORE=483
*
 BAD AVG GOOD
*
Cpa 00000217.44      :  59
Cpa 00000769.37      :  55
Popu 3444.2          :  47
Popu 505.8           :  56
Popu 2025.19         :  53
Popu 2186.6          :  65
Popu 2302.6          :  56
Ot06g02530           :  57
Cre06.g275350.t      :  40
kfl00255 0190 v      :  44
kfl00237 0160 v      :  58
Pp1s96 165V6.1       :  43
Pp1s46 272V6.1       :  61
Pp1s198 135V6.1      :  56
Pp1s325 68V6.1       :  44
Pp1s160 6V6.1        :  57
Smo78045|PACid       :  59
MA 11267g0020        :  47
MA 115536g0010       :  51
MA 102199g0010       :  52
LOC Os02g46030       :  53
LOC Os01g06320       :  60
LOC Os02g45670       :  61
LOC Os06g01670       :  60
LOC Os06g51260       :  53
LOC Os04g49450       :  53
LOC Os08g06110       :  49
At5g17300            :  59
At3g09600            :  60
At5g02840            :  60
At1g01060            :  45
At2g46830            :  54
At1g18330            :  59
At5g52660            :  58
At5g37260            :  65
At1g01520            :  51
At4g01280            :  58
At3g10113            :  60
cons                 :  48

Cpa_00000217.44      --MSRRA--------------------------------------------------------
Cpa_00000769.37      --MLPAQ-------------------KSPADSGVEGA--------------------------
Popu_3444.2          --MDGYVGAGTAHQKT--V----RMAGVVGSADAHGA--------------------------
Popu_505.8           --MDPNQIKA------SSRDHATGV-PEAQRVVDAPP--------------------------
Popu_2025.19         --MASAHD-------------------------------------------------------
Popu_2186.6          --MSDMQVPVKVEA-------------------------------------------------
Popu_2302.6          --MMVF---------------------------------------------------------
Ot06g02530           --MGDQG-----------EA-------------------------------------------
Cre06.g275350.t      --MKEAK--------------------------------------------------------
kfl00255_0190_v      --MDMMQTGYQNQSQDGTSPRPSFW-PQFMMGQGGMV--------------------------
kfl00237_0160_v      --MLGQG--------------------------------------------------------
Pp1s96_165V6.1       --MGLWVQSLT-------------FA---------LTDGT-----------------------
Pp1s46_272V6.1       --MNAAS--------------------------------------------------------
Pp1s198_135V6.1      --MNAAG--------------------------------------------------------
Pp1s325_68V6.1       --MIGNQF-------------------------------------------------------
Pp1s160_6V6.1        --MNAAS--------------------------------------------------------
Smo78045|PACid_      --M--AN--------------------------------------------------------
MA_11267g0020        --MASFPAVA-------------VPQ---------GE--------------------------
MA_115536g0010       --MK-----------------------------------------------------------
MA_102199g0010       --MLPLGSRFFSPSFP------AQQI-SLK-----VT-YIGAGWNFCNRDGGELEETIPTHCV
LOC_Os02g46030       MEMACLPGNA-------------MAT---------DE-N------------------------
LOC_Os01g06320       --M------------------------------------------------------------
```

CCA1/LHY/RVEs

```
LOC_Os02g45670   --MVSAN------Q----------------------P--------------------------
LOC_Os06g01670   --MSSAP--------------------------------------------------------
LOC_Os06g51260   --MASMPQLE-------------EKD---------SS--------------------------
LOC_Os04g49450   --MARFQETK-------------ARN---------DQ-G------------------------
LOC_Os08g06110   ---------------------------------------------------------------
At5g17300        --MASSPLTA-------------NVQ---------GT--------------------------
At3g09600        --MSSSP--------------------------------------------------------
At5g02840        --MTSTN--------------------------------------------------------
At1g01060        ---------------------------------------------------------------
At2g46830        ---------------------------------------------------------------
At1g18330        --M------------------------------------------------------------
At5g52660        --MVSRNSDGYFLD----------------------P--------------------------
At5g37260        --MA-----------------------------------------------------------
At1g01520        --MVTVNPSQAHCL----------------------P--------------------------
At4g01280        --MVSVNP-RPKGF----------------------P--------------------------
At3g10113        --MVMMIIIY-------------TEP-------------------------------------

cons

Cpa_00000217.44  ---------------------------------------------AL-P--------------
Cpa_00000769.37  PAS---------------------------I------------WWQ-----------------
Popu_3444.2      TEQ---------------------------Q------------RRYA-GE-LAAKQQQLKRAY
Popu_505.8       ANG---------------------------L------------RNL-----------------
Popu_2025.19     ---------------------------------------------------------------
Popu_2186.6      ---------------------------------------------------------------
Popu_2302.6      ---------------------------------------------------------------
Ot06g02530       ---------------------------------------------------------------
Cre06.g275350.t  ---------------------------------------------------------------
kf100255_0190_v  PPG---------------------------Q------------VPP-----------------
kf100237_0160_v  --------------------------------------------AFS-PGLGP----------
Pp1s96_165V6.1   KEKVV-AL------LKIGLGFQCGYGTP-RTKVMIPMTPSSSAAGYREH--EG----------
Pp1s46_272V6.1   ---------------------------------------------VE----ML----------
Pp1s198_135V6.1  ---------------------------------------------SE----ML----------
Pp1s325_68V6.1   -----------------------------------------------QH--EG----------
Pp1s160_6V6.1    ---------------------------------------------SE----ML----------
Smo78045|PACid_  ---------------------------------------------PA----------------
MA_11267g0020    RTET---------------------------------------LAGQ-QV-IQ----------
MA_115536g0010   --------------------------------------------MSL-PS-------------
MA_102199g0010   QLYTEDKPSQEIERKSKTYDKADQDLTISAV------------RTF--A--VS----------
LOC_Os02g46030   -GAD---------------------------------------DRAG-GE-S-----------
LOC_Os01g06320   ---------------------------------------------------------------
LOC_Os02g45670   ------------------------------P--------------------------------
LOC_Os06g01670   ---------------------------------------------Q--QLL------------
LOC_Os06g51260   DLAINKGP---------------------SL------------DLVK-SP-L-----------
LOC_Os04g49450   -PVA---------------------------------------DHVG-HQ-N-----------
LOC_Os08g06110   ---------------------------------------------------------------
At5g17300        NASL---------------------------------------RNRD----------------
At3g09600        ---------------------------------------------SR-NPT------------
At5g02840        ---------------------------------------------PV-VAEVI----------
At1g01060        ---------------------------------------------------------------
At2g46830        ---------------------------------------------------------------
At1g18330        -----------------------------AA------------EDRS-EE-LS----------
At5g52660        ------------------------------T------------GMTV-PGL------------
At5g37260        ---------------------------------------------------------------
At1g01520        ------------------------------M------------KMSL-PGFN-----------
At4g01280        ------------------------------V------------FDSS-NMSL-----------
At3g10113        EI---SLF---------------------PL------------QDRS-EE-LS----------

cons

Cpa_00000217.44  ------------------------------------------------P-S------------
Cpa_00000769.37  ----------EGLGV--------------PSASGGTSSDQGDFYNREPG------MAQ--A--
Popu_3444.2      AYGSGAGGPATGASALDMRQQQHEQMANMASVMLQGGEGMYATSARFGG------SSGAPATF
Popu_505.8       ----------ISRL--------------SASDSMYPGQKLALGIAAGSE------HEP-----
Popu_2025.19     -----------------------------------------AAE-------------------
Popu_2186.6      ------------------------------------VQVTQRSG-------------------
```

```
Popu_2302.6       ---------------------------------------------------------------
Ot06g02530        -------------------------------------------PS------------------
Cre06.g275350.t   -----------------------------SPKSGPA---------------------------
kf100255_0190_v   ----------LAGG--------------PAVYPSGPTPALARYPSGSVR------PSL-----
kf100237_0160_v   --P-----------------------------PG---------------DM----AAG-----
Pp1s96_165V6.1    ----------------------------------A------------GE------EGT-----
Pp1s46_272V6.1    ---------------------------------G-------------VM------VAG-----
Pp1s198_135V6.1   ---------------------------------G-------------VM------VAG-----
Pp1s325_68V6.1    ----------------------------------A------------GE------GGN-----
Pp1s160_6V6.1     ---------------------------------G-------------VM------VAG-----
Smo78045|PACid_   ---------------------------------------------------------------
MA_11267g0020     ----------------------------------S------------NS------VHY-----
MA_115536g0010    -----------------------------------------------NI------LTL-----
MA_102199g0010    ----------------------------------C------------SV------RGD-----
LOC_Os02g46030    --------------------------------------------------------TV-----
LOC_Os01g06320    ---------------------------------------------------------------
LOC_Os02g45670    ---------------------------------------------------------------
LOC_Os06g01670    ------------------------------------------------DSSS-----------
LOC_Os06g51260    --------------------------------------------------------MM-----
LOC_Os04g49450    --------------------------------------------------------LM-----
LOC_Os08g06110    ---------------------------------------------------------------
At5g17300         ---------------------------------------------------------------
At3g09600         ------------------------------------------------NAEA-----------
At5g02840         ---------------------------------P---------A---ETSTD-----------
At1g01060         ---------------------------------------------------------------
At2g46830         ---------------------------------------------------------------
At1g18330         ----------------------------------S------------NV------ENG-----
At5g52660         ------------------------------------------------GPSFTAAVSS-----
At5g37260         --------------------------------------------------------MQ-----
At1g01520         -------------------------------------------------TLP-----------
At4g01280         -------------------------------------------------PSS-----------
At3g10113         ----------------------------------S------------NV------ENG-----

cons

Cpa_00000217.44   ------------------------------------------------------VLA------
Cpa_00000769.37   -----------SLSSAQLAGPFGA--E-HVRPL---------------A----V--T---A--
Popu_3444.2       TQHQGATMLAGLQ--V-NGGPSVAGSA-SGHA----------------A----M--PQLGQHG
Popu_505.8        ---------K-PQRHLQLER--Q-ETGACNPI----------------V----S--P---H--
Popu_2025.19      ------------------------FGE-H----------------------------------
Popu_2186.6       ------------------RGPSKKERA-P----------------------------NGAA--
Popu_2302.6       ---------------------------------------------------------------
Ot06g02530        ----------------------S----NDTGD----------------E----A--T---V--
Cre06.g275350.t   -----------AL--P------------AQQP----------------S----S--A---G--
kf100255_0190_v   ---------P-APLLMPVPP--Q-PLHHAQSG----------------G----P--L---I--
kf100237_0160_v   ------------------------------------------------------PLT---P--
Pp1s96_165V6.1    --------------------------------Y--------------------W--P---S--
Pp1s46_272V6.1    ------------------------------------------------------STV---P--
Pp1s198_135V6.1   ------------------------------------------------------STV---P--
Pp1s325_68V6.1    --------------------------------C--------------------W--P---S--
Pp1s160_6V6.1     ------------------------------------------------------STL---S--
Smo78045|PACid_   ---------------------------------------------------------------
MA_11267g0020     ---S----------------------EQ-SKSQ--------------------S--P---S--
MA_115536g0010    -----------------------------------------------------S--G---D--
MA_102199g0010    --------------------------------VIETRYELIVYHHFDTGCFETF--K---Y--
LOC_Os02g46030    --------------------------------D--------------------H--L---R--
LOC_Os01g06320    --------------------------------------------------------D---R--
LOC_Os02g45670    ---------------------------------------------------------------
LOC_Os06g01670    ------------------------------------------------------PGP------
LOC_Os06g51260    --------------------------------N--------------------D--A---S--
LOC_Os04g49450    --------------------------------E--------------------N--L---T--
LOC_Os08g06110    ---------------------------------------------------------------
At5g17300         -------------------------------EE--------------------T--A---D--
At3g09600         ------------------------------------------------------PPP------
At5g02840         ------------------------------------------------------ATE------
At1g01060         ---------------------------------------------------------------
At2g46830         ---------------------------------------------------------------
At1g18330         --------------------------------S--------------------C--N---S--
```

```
At5g52660        -------------------------------SS--------------------SPTT---S--
At5g37260        -----------------------------------------------------E--R---C--
At1g01520        ------------------------------------------------------HTA---T--
At4g01280        ------------------------------------------------------DGF---G--
At3g10113        --------------------------------S--------------------C--N---S--

cons


Cpa_00000217.44  --------------------PP-VI-SPIA-PPPNPPKERKPYTITKQRENWTEEEHQKFLEA
Cpa_00000769.37  -----------V-------A-AAAA-AALA-DSSAMKKPRKPYTITKPREVWTDEEHARFIEG
Popu_3444.2      SFANGEQFSLSSS--QDTGT-ANTS-KAGG-ALSAGSRVRKPYRVTKSREAWTTEEHQSFLDA
Popu_505.8       -----------A-----LV--SSDS-DRAR-GGNSTPRVRKPYTKTNGRQVWTAEEHERFLAA
Popu_2025.19     -----------ED--E-HE--GGVV-SAGS-HADGGKKARKPYTITKHRESWSQEEHARFLEA
Popu_2186.6      -----------AT--TAAG--RSFSTASCA-GSA-AGRVRKPYVLTKKREYWSDAEHARFVAA
Popu_2302.6      ----------------------PAP-RAGR-VNKKSDKVRKQYTRTKVREAWTESEHERFLEA
Ot06g02530       -----------T-----TN--DATS-DPTT-TEGKAVKTRKPYTITKKRERWSDEEHALFVES
Cre06.g275350.t  -----------P-----S-A-GPSQ-QADQ-ESTPKIKARKPYIITKQRERWTDEEHARFLEA
kfl00255_0190_v  -----------Q-----QM--QPGA-KPST-SESLSTKVRKPYTITKQRERWTEAEHDRFLEA
kfl00237_0160_v  ---------------V-L-S-PSGE-LSGK-GDDMHGKVRKPYTITKQRENWTEQEHNKFLKA
Pp1s96_165V6.1   -----------V-----S-P-GVP--LSTSGDEGASTKVRKPYTITKQRERWTEEEHQKFLEA
Pp1s46_272V6.1   ---------------------G-IP-SVSV-SEEGSKKIRKPYTITKSRESWTEQEHDKFLEA
Pp1s198_135V6.1  ---------------------G-MT-SVSV-SEEGSKKIRKPYTITKSRESWTEQEHDKFLDA
Pp1s325_68V6.1   -----------V-----A-P-GIP--LFTSGDEGTGTKVRKPYTITKQRERWTEEEHQRFLEA
Pp1s160_6V6.1    ---------------------S-IP-PIPV-SEEGSKKIRKPYTITKSRESWTEQEHDKFLEA
Smo78045|PACid_  ---------------------------LPS-DDAVSKKIRKPYTITKSRESWTEQEHDKFLEA
MA_11267g0020_   -----------I-----R-F-PIDE-VSSS-GDEFTTKVRKPYTITKQRERWTEEEHHKFLEA
MA_115536g0010   -----------S-----N-S-NSNS-ICSS-GDELAAKVRKPYTITKQRERWSEEEHLKFLEA
MA_102199g0010   -----------W-----R-I-EEGE-THFS-IDEPFSKVRKPYTITKQRERWTEDEHKKFLDA
LOC_Os02g46030   -----------S-----H-M-NYGD-MDLS-GEEHVPKARKPYTITKQREKWTDEEHRLFLEA
LOC_Os01g06320   -----------N-----T-N-NNSN-SSSS-SEMPGKKARKPYTITKPRERWSEEEHERFLDA
LOC_Os02g45670   -----------------P-DAAAAA-AGSA-GEDASKKVRKPYTITKSRESWTEQEHDKFLEA
LOC_Os06g01670   ---------------------G--P-GPEV-EDDGGRRVRKPYTITKSRESWTDPEHDKFLEA
LOC_Os06g51260   -----------A-----T-V-TAMQ-PNEG-MEEFPVKVRKPYTITKQREKWTEEEHDKFLEA
LOC_Os04g49450   -----------D-----P-L-DSSG-MDMM-DEARIPKARKPYTITKQREKWTEDEHKLFLEA
LOC_Os08g06110   -----------------------ME-INSS-GEEAVVKVRKPYTITKQRERWTEAEHNRFLEA
At5g17300        -----------K-----Q-I-QFND-QSFG-GNDYAPKVRKPYTITKERERWTDEEHKKFVEA
At3g09600        ---------------------PPTS-TDAV-AEGSSKKVRKPYTITKSRESWTEEEHDKFLEA
At5g02840        ---------------------TTIA-TTEA-GEAPEKKVRKAYTITKSRESWTEGEHDKFLEA
At1g01060        -----------------------MD-TNTS-GEELLAKARKPYTITKQRERWTEDEHERFLEA
At2g46830        -----------------------ME-TNSS-GEDLVIKTRKPYTITKQRERWTEEEHNRFIEA
At1g18330        -----------N-----E-G-INPE-TSSH-WIENVVKVRKPYTVTKQREKWSEEEHDRFLEA
At5g52660        -----------STAVA-V-ADVTAM-VSSS-EEDLSKKIRKPYTITKSRESWTEPEHDKFLEA
At5g37260        -----------E-----S-L-CSDE-LISS-SDAFYLKTRKPYTITKQREKWTEAEHEKFVEA
At1g01520        -------------TIP-V-SIRSNR-TMSF-FEDPTKKVRKPYTITKSRENWTEQEHDKFLEA
At4g01280        -------------SIP-A-TG-RTS-TVSF-SEDPTTKIRKPYTIKKSRENWTDQEHDKFLEA
At3g10113        -----------N-----E-G-INPE-TSSH-WIENVVKVRKPYTVTKQREKWSEEEHDRFLEA

cons                                                  : ** *  .: *: *:  **  *: .


Cpa_00000217.44  LKLYERDWKKIENYIGTKSVVQIRSHAQKFFLKKTGQPQL----------------------T
Cpa_00000769.37  LKLYERDWKRIQQHVETKNVMQIRSHAQKFFLKVQKTGQAG----------------------
Popu_3444.2      LLEHGRDWKLIQKRVKSKDLQQVRSHAQKYFLKVQKNNTGE----------------------
Popu_505.8       LKLYGREWKKIQDHVETKTVPQIMSHAQKHFLKVQKKD-------------V-----------
Popu_2025.19     LRRYDRDWKRIEGHVGTKNVIQIRSHAQKYFLKVQKNQTGE----------------------
Popu_2186.6      LQKHGREWKAIENEVGTKSAVQIRSHAQKFFLRLERNSVPE--E------R------------
Popu_2302.6      LKLFDRNWKCVTEHVGTKDIVQVRSHAQKFFLKLRKTDALE----------------------
Ot06g02530       LKKYGRAWKRIEEYIGTKSAVQIRSHAQKFFAKLQKEQIVASGS------EGS---------G
Cre06.g275350.t  LKLYGRAWRKIEEHVSTKTAVQIRSHAQKFINKLERNKDSGSTK------DGE----------
kfl00255_0190_v  LKLHGRAWRKIEEHIGSKTAVQIRSHAQKFFSKLEREGSTAN-P------AS-----------
kfl00237_0160_v  LELFDRDWRKIEEYVGTKTVIQIRSHAQKYFLKVQKLGQD----------------AHVPPPR
Pp1s96_165V6.1   LKLYGRAWRRIEEHIGTKTAVQIRSHAQKFFSKIERDVTAGQ-GT----ETGVAQVIDIPPPR
Pp1s46_272V6.1   LQLFDRDWKKIEAFVGSKTVIQIRSHAQKYFLKVQKNGTG----------------EHVPPPR
Pp1s198_135V6.1  LQLFDRDWKKIEAFVGSKTVIQIRSHAQKYFLKVQKNRTG----------------EHVPPPR
Pp1s325_68V6.1   LKLYGRAWRRIEEHIGTKTAVQIRSHAQKFFSRNRKXMSVQGKGP----FSGVAQVVTTG---
Pp1s160_6V6.1    LQLFDRDWKKIEAFVGSKTVIQIRSHAQKYFLKVQKNGTG----------------EHVPPPR
Smo78045|PACid_  LQLFDRDWKKIEAFVGSKTVIQIRSHAQKYFLKVQKNGTG----------------EHVPPPR
```

```
MA_11267g0020      LKMHGRAWRRIEEHIGTKTAVQIRSHAQKFFSKLEREASAGGVP------MGKAQDISIPPPR
MA_115536g0010     LKMYGRAWRRIEEHIGTKTAVQIRSHAQKFFSKLVRGSSSKGVSS-----TERTQDIDIPPPR
MA_102199g0010     LKLYGRSWRHIEEHIGTKSAVQIRSHAQKFFTKLEKGASTGTSS------TMTYQYLEIPPPR
LOC_Os02g46030     LQLHGRAWRRIQEHIGTKTAVQIRSHAQKFFSKVVRESSGSNTGSGGASAAAAAAAIQIPPPR
LOC_Os01g06320     LIMYGRDWKKIEEHVGTKTTIQIRSHAQKYFLKVQKMGLA----------------AGLPPQY
LOC_Os02g45670     LQLFDRDWKKIEAFVGSKTVIQIRSHAQKYFLKVQKNGTS----------------EHVPPPR
LOC_Os06g01670     LQLFDRDWKKIEAYVGSKTVIQIRSHAQKYFLKVQKNGTG----------------EHLPPPR
LOC_Os06g51260     LKLYGRSWRQIQEHIGTKTAVQIRSHAQKFFSKVVREPG----S---------NNAIEIPPPR
LOC_Os04g49450     LQLHGRAWRRIQEHIGTKTAVQIRSHAQKFFSKVIKESSGDNCNS-----LGAASSIQIPPPR
LOC_Os08g06110     LKLYGRAWQRIEEHVGTKTAVQIRSHAQKFFTKLEKEAINNGTS------PGQAHDIDIPPPR
At5g17300          LKLYGRAWRRIEEHVGSKTAVQIRSHAQKFFSKVAREATGGDG--------SSVEPIVIPPPR
At3g09600          LQLFDRDWKKIEDFVGSKTVIQIRSHAQKYFLKVQKNGTL----------------AHVPPPR
At5g02840          LQLFDRDWKKIEDFVGSKTVIQIRSHAQKYFLKVQKNGTL----------------AHVPPPR
At1g01060          LRLYGRAWQRIEEHIGTKTAVQIRSHAQKFFTKLEKEAEVKGIP------VCQALDIEIPPPR
At2g46830          LRLYGRAWQKIEEHVATKTAVQIRSHAQKFFSKVEKEAEAKGVA------MGQALDIAIPPPR
At1g18330          IKLYGRGWRQIQEHIGTKTAVQIRSHAQKFFSKMAQEADSRSEG--------SVKAIVIPPPR
At5g52660          LQLFDRDWKKIEAFIGSKTVIQIRSHAQKYFLKVQKSGTG----------------EHLPPPR
At5g37260          LKLYGRAWRRIEEHVGTKTAVQIRSHAQKFFTKVARDF----GV--------SSESIEIPPPR
At1g01520          LHLFDRDWKKIKAFVGSKTVIQIRSHAQKYFLKVQKNGTK----------------EHLPPPR
At4g01280          LHLFDRDWKKIEAFVGSKTVVQIRSHAQKYFLKVQKSGAN----------------EHLPPPR
At3g10113          IKLYGRGWRQIQEHIGTKTAVQIRSHAQKFFSKMAQEADSRSEG--------SVKAIVIPPPR

cons               :   . * *: :    : :*    *: *****.: :


Cpa_00000217.44    DQQRLAQEQAE-QEQLV----------------------------------------------
Cpa_00000769.37    ----------------------------ITIPPARPKRKNGTGT------AAQAA--------
Popu_3444.2        -----------------------------VIPPARPKRKSALP------YPRSAKMQT-----
Popu_505.8         ---------------------D------GFVPPPRPKRKSTK------PYPRKADSDIKESGP
Popu_2025.19       -----------------------------HVPPPRPKKRTAKRAFSDDQYXXXXXXXX-----
Popu_2186.6        ----------------------------PQVPPPRPRKRASSGSS---PVPSM----------
Popu_2302.6        -----------------------------ATASSQDSAAPAAPG------PANQAEPT-----
Ot06g02530         STRKRGAD--RSTSQSKRSKSSYATDINLEIPPARPKKKPAH------PYPRKATSQQ-----
Cre06.g275350.t    ------------------------G---IRIPPPRPKRKPSRPY------PRKEPILA-----
kfl00255_0190_v    ---------------------S--A-PEIDIPPPRPKRKPSH------PYPRKAGGNM-----
kfl00237_0160_v    PKRKSSQPYPQKASKVVAA--------------------------------------------
Pp1s96_165V6.1     PKRKPTHPYPRKAGRSFGK--------------------------------------------
Pp1s46_272V6.1     PKRKSAQPYPQKAPKC-----------------------------------------------
Pp1s198_135V6.1    PKRKSAQPYPQKASKCVPA--------------------------------------------
Pp1s325_68V6.1     ---------------------------------------------------------------
Pp1s160_6V6.1      PKRKSVQPYPQKAPKTA----------------------------------------------
Smo78045|PACid_    PKRKSAQPYPQKAAKPGKL--------------------------------------------
MA_11267g0020      PKRKPSHPYPRKAGTAIQ---------------------------------------------
MA_115536g0010     PKRKPRHPYPRKAVGTAHQ--------------------------------------------
MA_102199g0010     PKRKPGHPYPKKTGVIDRG--------------------------------------------
LOC_Os02g46030     PKRKPAHPYPRKVDGAAKK--------------------------------------------
LOC_Os01g06320     PRRRLVMQQQQQQSSPAVS--------------------------------------------
LOC_Os02g45670     PKRKAAHPYPQKASKNE----------------------------------------------
LOC_Os06g01670     PKRKAAHPYPHKASKRAPQ--------------------------------------------
LOC_Os06g51260     PKRKPLHPYPRKCANSGSD--------------------------------------------
LOC_Os04g49450     PKRKPVHPYPRNLGSTASK--------------------------------------------
LOC_Os08g06110     PKRKPNSPYPRKSCLSSET--------------------------------------------
At5g17300          PKRKPAHPYPRKFGNEADQ--------------------------------------------
At3g09600          PKRKAAHPYPQKASKNAQM--------------------------------------------
At5g02840          PKRKAAHPYPQKASKNAQM--------------------------------------------
At1g01060          PKRKPNTPYPRKPGNNGTS--------------------------------------------
At2g46830          PKRKPNNPYPRKTGSGTIL--------------------------------------------
At1g18330          PKRKPAHPYPRKSPVPYTQ--------------------------------------------
At5g52660          PKRKAAHPYPQKAHKNV----------------------------------------------
At5g37260          PKRKPMHPYPRKLVIPDAK--------------------------------------------
At1g01520          PKRKANHPYPQKAPKFTL---------------------------------------------
At4g01280          PKRKASHPYPIKAPKNVAY--------------------------------------------
At3g10113          PKRKPAHPYPRKSPVPYTQ--------------------------------------------

cons


Cpa_00000217.44    ---------------------------------------------------------------
```

```
Cpa_00000769.37   ---------------------------------------------------------------
Popu_3444.2       -PQEQEQ-------------PQ----RQEQQ-QQ-ERHQQQM----------DRCG-----GS
Popu_505.8        SQAGSSVSRPQQPAAQGPA-MT----MT-TAGV-------F----------------------
Popu_2025.19      -XXXLHR-----QDRR-QG-GF----LE-ASPVS-GSR--TG----------S----------
Popu_2186.6       ---------------------------------------------------------------
Popu_2302.6       -CQSR------------------------------PAGVPET----------R--A-----AP
Ot06g02530        -PSGGSGER----DN--SG-G------T-GKSSG---------------------------TA
Cre06.g275350.t   -DHSGS------------------------GAGD-VSGINVS----------GM-N-----VS
kfl00255_0190_v   -SESESEYGPSPRTGSRTE-PL----DE-ALSPN-TDSLQRG----------QS-R-----SF
kfl00237_0160_v   ---AHA---A--Q------QAA-------VASSKVYS--------------------------
Pp1s96_165V6.1    ---TSEDE------------CP----LAAAGSIVSSSGVS-T-------------ANISEACL
Pp1s46_272V6.1    ---------------------------------------------------------------
Pp1s198_135V6.1   ---QLQ-----VS------DGLRTHSGQ-TESSHGTCSRI-----------PS----------
Pp1s325_68V6.1    ---------------------------------------------------------------
Pp1s160_6V6.1     ---QVQ-----VS------DGLRTQAVQ-TESSYGTGSHK-----------PP----------
Smo78045|PACid_   ---PIPNCEFHSVFLCSTAPPQRGTPHP-PPPDFAYMVPQ-----------CN----------
MA_11267g0020     ---IGP--------------ST----NE-EENRSPSAVSS-A----------PDR-----LCI
MA_115536g0010    ---SGS--------------PA----SK-EGDILPSVVSS-V----------PLL-----S--
MA_102199g0010    ---GD----------F--S--I----LE-EDSRTRGCKVS-E----------REC-----SKL
LOC_Os02g46030    ---HVPALRQLEKPPLWMQ-SL----SE-QEEGSPTSVLT-A----------AQIG---TEAL
LOC_Os01g06320    ---SSVAATAILHGQPQCL-PP----HH-NVAVQSSIGWE-CPGVLPPATND-----------
LOC_Os02g45670    -------------------------------PGYTIKADS-----------S-----------
LOC_Os06g01670    ---VVL----PQQ------ASH-LME-----QGCLIPMDI-----------S-----------
LOC_Os06g51260    ---ANPATAQLKLAPGSSS-SG----SD-QENGSPISVLS-A----------MQS-----DAF
LOC_Os04g49450    ---NVPALKQLEKPQLQVQ-SL----YD-QDNGSPTSVLT-V----------PQIR---ADTL
LOC_Os08g06110    ---STREVQNDKATISNMT-NN----ST-AQMAGDAALEK-LQRKEISEKGSCSE-----V--
At5g17300         ---TSR-----------SV-SP----SE-RDTQSPTSVLS-T----------VGS-----EAL
At3g09600         ---PLQ-----VS------TSFTTTRNG-DMPGYASWDD------------AS----------
At5g02840         ---SLH-----VS------MSFPTQI-N-NLPGYTPWDDD-----------T-----------
At1g01060         ---SSQVSSAKDAKLVSSA-SS----SQ-LNQA----FLD-LEKMPFSEKTSTGK-----ENQ
At2g46830         ---MSKTGVNDGKESLGSE-KV----SH-PEMA----NED-RQQSKPEEKTL----------Q
At1g18330         ---SP----------PPNL-SA----ME-KGTKSPTSVLS-S----------FGS-----EDQ
At5g52660         ---QLQ-----VP------GSFKSTSEP-NDPSFMFRPES-----------S-----------
At5g37260         ---EMVYAE-----LTGSK-LI----QD-EDNRSPTSVLS-A----------HGS-----DGL
At1g01520         ----------------------SSSNAL-FQHDYLYNTNS-----------H-----------
At4g01280         ---TSL------P--------SSSTLPL-LEPGYLYSSDS-----------K-----------
At3g10113         ---SP----------PPNL-SA----ME-KGTKSPTSVLS-S----------FGS-----EDQ

cons

Cpa_00000217.44   ---------------------------------------------------------------
Cpa_00000769.37   ---------------------------------------------------------------
Popu_3444.2       Q-GSLSEVAE---------------HP--------------VDW---QGLQRQQRLHLQF---
Popu_505.8        TSWH------------SD-----PYPV---------------------ADSSMAFGSV-----
Popu_2025.19      -----SQP-------------------------------------------------------
Popu_2186.6       ---------K-----------------------------------------------------
Popu_2302.6       -----------SKL-------------------------------------HSAVHR------
Ot06g02530        QKWPT----------------------------------------------------------
Cre06.g275350.t   G-INGAAATATGTATGTATATASQGTG--------------LAQ---QQQQQQSKLSLPVPPR
kfl00255_0190_v   SLWRNGSA-----PELPL-----G-PS---------------------PAVSRDPAAL-----
kfl00237_0160_v   ---------------------------------------------------------------
Pp1s96_165V6.1    KEGVWDQDD------TG---VA---GH-------------QDAAKAKKDADTAHSWG------
Pp1s46_272V6.1    ---------------------------------------------------------------
Pp1s198_135V6.1   ------M--------------------------------------------------------
Pp1s325_68V6.1    ---------------------A---FH-------------QDAAKSYNDADATHPWG------
Pp1s160_6V6.1     ------M--------------------------------------------------------
Smo78045|PACid_   ------M--------------------------------------------------------
MA_11267g0020     GSNLGGETH------KN---AA---FA---------------PY-------------------
MA_115536g0010    ---LGDGPC------KR---AR---FD---------------RH-------------------
MA_102199g0010    TELAQHNS-------------K---SR---------------S--------------------
LOC_Os02g46030    GGGFSNNSS------GS---GS---LAPSAAGTDEHVDGGGSPA-------------------
LOC_Os01g06320    MQ-------------------------------------------------------------
LOC_Os02g45670    -----SM--------------------------------------------------------
LOC_Os06g01670    -----PV--------------------------------------------------------
LOC_Os06g51260    GSSVSNPS--------T---RC---TS---------------P-----------A--------
LOC_Os04g49450    GS-----------------------------------ESGGSPT-------------------
LOC_Os08g06110    ---------------------------------------------------------------
At5g17300         CSLDSSSP--------N---RS---LS---------------PV-------------------
```

```
At3g09600         -----M---------------------------------------------------------
At5g02840         -----SA--------------------------------------------------------
At1g01060         DEN------------------------------------------------------------
At2g46830         EDN------------------------------------------------------------
At1g18330         VNRCSSPN------------SC---TS---------------DI------QSIGA--------
At5g52660         -----SM--------------------------------------------------------
At5g37260         GSIGSN--S------PN---SS---SA---------------EL-------------------
At1g01520         ---------------------------------------------------------------
At4g01280         -----SL--------------------------------------------------------
At3g10113         ---------------------------------------------------------------

cons


Cpa_00000217.44   ---------------------------------------------------------------
Cpa_00000769.37   ---------------------------------------------------------------
Popu_3444.2       --------QXXX-----------XX--------------------------------------
Popu_505.8        ------PFP--------------FE--------------------------------------
Popu_2025.19      ---------------------------------------------------------------
Popu_2186.6       -----------R-----------AP--------------------------------------
Popu_2302.6       -H----MSPQSP-----------AV-------S------------------------------
Ot06g02530        ---------------------------------------------------------------
Cre06.g275350.t   SSAMPLQPPTAA-----------LQQLQLQ--LPLGL--------------------------
kfl00255_0190_v   ------PLSLGR-----------SV--------------------------------------
kfl00237_0160_v   -------------------------------------PRE-----------------------
Pp1s96_165V6.1    -----VPKTVNSATSGSPTARPVNVALNGG--LPLN---------------------------
Pp1s46_272V6.1    ---------------------------------------------------------------
Pp1s198_135V6.1   ---------------------------------SPASPSVS----------------------
Pp1s325_68V6.1    -----IPKTVNSTTAGSPPARPANMASNTG--LLLN---------------------------
Pp1s160_6V6.1     ---------------------------------TSTSPSIS----------------------
Smo78045|PACid_   ---------------------------------FVPGVTAS----------------------
MA_11267g0020     -----------------------NA--RPRPLQTND---------------------SIN---
MA_115536g0010    -----------------------NA--KTRLC-NSNN--------------------------
MA_102199g0010    -----------------------------------LDK-------------------------
LOC_Os02g46030    -----------------------SSVDRED--GCLSPSIPTAEL---AMQA------PNT---
LOC_Os01g06320    -----------------------------N-L-EWASTSGT----------------------
LOC_Os02g45670    ---------------------------------LRNSGMNA----------------------
LOC_Os06g01670    ---------------------------------ARNFNAND----------------------
LOC_Os06g51260    -----------------------SSDDGNN-I-PTFTSGEDNNVPCEPTVIDPSQSHKEIDQD
LOC_Os04g49450    -----------------------STIDIEE--RCPTPSIATAEL---AMEL------PPT-ND
LOC_Os08g06110    ----------------------------LN-L-FREVPSAS----------------------
At5g17300         -----------------------SS--ASPPAALTTTANAPEELETLKLEL------FPS---
At3g09600         ---------------------------------LLN----R----------------------
At5g02840         ---------------------------------LLNIAVSG----------------------
At1g01060         ---------------------------CSG-V-STVNKYPL----------------------
At2g46830         ---------------------------CSD-C-FTHQYLSA----------------------
At1g18330         -----------------------TSIDKKN-N-YTTS--------------------------
At5g52660         ---------------------------------LMTSPTTA----------------------
At5g37260         -----------------------SS--------------------------------------
At1g01520         ------------------------------------PVIST----------------------
At4g01280         ---------------------------------MGNQAVCA----------------------
At3g10113         -----------------------------N-N-YTTS--------------------------

cons


Cpa_00000217.44   ---------------------------------------------------------------
Cpa_00000769.37   ---------------------------------------------------------------
Popu_3444.2       ---------------------------------------------------------------
Popu_505.8        ------------VS----A------------H---------NYQS---FYRTNNQPLFVSNPS
Popu_2025.19      ---------------------------------------------------------------
Popu_2186.6       ---------------------------------------------------------------
Popu_2302.6       ---------------------------------------------------------------
Ot06g02530        ---------------------------------------------------------------
Cre06.g275350.t   ------GQPQPPALAPAVAAAAAAAAVVKQQQHQQQQHHLLL---------------------
kfl00255_0190_v   ------------SVGPALP------------D---------T--Q---Q-Q----------YN
kfl00237_0160_v   ----------------------------------------GA--YTPGG-A----------HP
Pp1s96_165V6.1    ----------NI------S---------------------PV--P---I-P----------GF
```

```
Pp1s46_272V6.1       ---------------------------------------------------------------
Pp1s198_135V6.1      ----------------------------------------A---W-----V----------QQ
Pp1s325_68V6.1       ----------NI------S---------------------LG--P---I-P----------AF
Pp1s160_6V6.1        ----------------------------------------A---W-----V----------QH
Smo78045|PACid_      ----------------------------------------P---V-----S----------SW
MA_11267g0020        ----LQETNMES------S---------------------ST--S---I-S----------PS
MA_115536g0010       ----GQEVKMES------P---------------------RL--N---S-S----------PL
MA_102199g0010       -----PKMTQGT------S---------------------QP--S---R-M----------ST
LOC_Os02g46030       KMSIATT---DA------K---------------------EA--S---S-E----------AS
LOC_Os01g06320       ----------------------------------------AA--W---G-N----------HH
LOC_Os02g45670       ----------------------------------------T---VS-SW-T----------HN
LOC_Os06g01670       ----------------------------------------V---FS-SW--------------
LOC_Os06g51260       RKDVNNMSEEDS------S---------------------EE--E---V-Q----------ET
LOC_Os04g49450       EEVKGNGDHEEV------T---------------------CD--R---S-G----------VP
LOC_Os08g06110       ------------------------------------------------F-S----------S-
At5g17300            ----ERLLNRES------S---------------------IK--E---P-T----------KQ
At3g09600            ----------------------------------------V---IS-PQ--------------
At5g02840            ----------------------------------------V---IP-PE--------------
At1g01060            ------------------------------------------------P-T----------KQ
At2g46830            ------------------------------------------------A-S----------SM
At1g18330            ----KQPFKDDS------D---------------------IG--S---T-P----------IS
At5g52660            ----------------------------------------A---AA-PW-T----------NN
At5g37260            ------HTEESL------S---------------------LE--A---E-T----------KQ
At1g01520            ----------------------------------------TR--KH-GL-V----------HC
At4g01280            ----------------------------------------ST--SS-SW-N----------HE
At3g10113            ----KQPFKDDS------D---------------------IG--S---T-P----------IS

cons
```

```
Cpa_00000217.44      ----------------------------------------------------------QAQKL
Cpa_00000769.37      --------------------------------------------------------------A
Popu_3444.2          -X----------------------------------------------------XX----XXX
Popu_505.8           RLVGELRPGGQIPLTVGPEAANLR------------------FTNEQV---------------
Popu_2025.19         --------------------------------------------------------------D
Popu_2186.6          -V----------------------------------------------------TR----ECA
Popu_2302.6          YS-------------------------------------------------------------
Ot06g02530           -----------------------E------------------ASQEFI---ASTS----SSAA
Cre06.g275350.t      ER-------------SLPSSQYS-------------------EVS-------EATV-----AA
kfl00255_0190_v      SLK--------LFG---------Q------------------LLGPKR---GEKDEGDQQRGA
kfl00237_0160_v      AVP-----PQ-------------S-----------------------------------PQEE
Pp1s96_165V6.1       PAM----PPWNRLFG--------GAMNP---------------AMMN---------PAM----
Pp1s46_272V6.1       ------------------------------GIKSEVEGVSLTAVRAS---------SNSISGS
Pp1s198_135V6.1      SVS----PNT-------------SISYDAPGTKPEVGGVSLTSVRAS---------SNSISGS
Pp1s325_68V6.1       PAV----PPWGRLFG--------GAMNP-----------------------------------
Pp1s160_6V6.1        SVS----PNP-------------SISYDAPGIKSEGEGVNLSAVRVP---------SNSISGS
Smo78045|PACid_      VHH----GGP-------------QGTIPFYKGGSFNQNNSSTANTWA---------QDPPAQA
MA_11267g0020        SLK----LFG-------------QTVLVPT------------NESAL---------DVNVKGA
MA_115536g0010       SLK----LFG-------------QTMLVST------------IEGPNTFSTE--PAVSNNQGD
MA_102199g0010       SLK----LFG-------------QTLVVQN------------SFTDD---FL--FTSVENEAI
LOC_Os02g46030       VFR----LFG-------------KSVVVKD------------SDQLH---------LLNGSNI
LOC_Os01g06320       ------------------------------------------GLIEP---------PAAFVSF
LOC_Os02g45670       SIP----PIV-------------ASS----MVKEDL-GAGAMAPNNF---------CSSSTEG
LOC_Os06g01670       -D------S---------------A-----LAQ-SFSPRHTHGAANN---------CSSSVES
LOC_Os06g51260       SLK----LFG-------------RTVVIPD------------PRKRS---------SSD----
LOC_Os04g49450       VLR----LFG-------------KRVMVND------------LHQMS---------APDAGNL
LOC_Os08g06110       --------------------------------------------VNK---------SSSNHGA
At5g17300            SLK----LFG-------------KTVLVSD------------SGMSS---------SLT----
At3g09600            -HE----LAT-------------LR-----GAEADIGSKGLLNVSSP---------STSGMGS
At5g02840            -DE----LDT-------------LC-----GAEVDVGSNDMISETSP---------SASGIGS
At1g01060            ------------------------------------------VSGDI---------ETSKTST
At2g46830            ------------------------------------------NKSCI---------ETSNAST
At1g18330            SIT----LFG-------------KIVLVAE------------ESHKP---------SSY----
At5g52660            AQT----ISF-------------TPL----PKG--------AGANNN---------CSSSSEN
At5g37260            SLK----LFG-------------KTFVVGD------------YNSSMS---------CD----
At1g01520            DVS----IPS-------------SVIKEEFGVSE----------NCC---------STSSSRD
At4g01280            STN----LPK-------------PVIEEEPGVSA-------TAPLPN---------NRCRQED
At3g10113            SIT----LFG-------------KIVLVAE------------ESHKP---------SSY----
```

```
cons

Cpa_00000217.44   L------------NDYMT----------------------AASAQTAQA--------------
Cpa_00000769.37   AA----------------------------------------AAAKAAA----VS-VITHIQA
Popu_3444.2       XXXXXXXXXXXXXHSFQPQ-------------------VQQADWSHVTM----DQ-LEFNYMY
Popu_505.8        --------------------------------------------------------ASVKA--
Popu_2025.19      AHHFF-----------LHD-------------------QQQAQ-VPRVR----EX-XXXXXXX
Popu_2186.6       SGAEQVVP-----GYTEPY-------------------SDRDASKHARV----PE-PRDETLI
Popu_2302.6       --------------------------------------AGPERRGIPGA----SH--------
Ot06g02530        IA----------------------------------------AVLSVAC----DK-MQNNL--
Cre06.g275350.t   VAA---------AASAAAA-------------------AAAAAVVAAAG----QQ-VQAHLQA
kfl00255_0190_v   TK----------------------------------------LLLEDGC----ERHATSER--
kfl00237_0160_v   T---------------------------------------GTLGAPAWG--------------
Pp1s96_165V6.1    --------------LN-----------------------------------------------
Pp1s46_272V6.1    S------------PGGWPQ---------------------HVLPASQIA--------------
Pp1s198_135V6.1   S------------PGGWP----------------------HVVSASPLA--------------
Pp1s325_68V6.1    ---------------------------------------------------------------
Pp1s160_6V6.1     S------------PGGWPQ---------------------HVLPASQVA--------------
Smo78045|PACid_   N------------PQRGRK---------------------TGEKVDRAN--------------
MA_11267g0020     F------------REFTSQKKETHPLDGE--------------NPEGSKL-----EN------
MA_115536g0010    F------------HEFVPVQQETSPLKIK---------------AKNQIL-----DD------
MA_102199g0010    C------------SEIYPEN-------------------------------------------
LOC_Os02g46030    A------------TSG-----------------------------------------------
LOC_Os01g06320    P------------G---------------------ESSFMGAASFSNTSMDWTGTTS------
LOC_Os02g45670    P------------ARAWQ----------------------PGETNDQIN--------------
LOC_Os06g01670    Q------------SGTCP----------------------TSEAIEQEI--------------
LOC_Os06g51260    --------------PKHESE-------------------------------------------
LOC_Os04g49450    Q------------TVA-----------------------------------------------
LOC_Os08g06110    S------------RGLEPTKTEVKDVVILERDSISNGAGKDAKDINDQE----M-ER------
At5g17300         ---------------------------------------------------------------
At3g09600         S------------SRTVS----------------------GSEIVRKAK--------------
At5g02840         S------------SRTLS----------------------DSKGLRLAK--------------
At1g01060         V------------D---------------------NAVQDVPKKNKDKD----G-ND------
At2g46830         F------------R---------------------EFLPSREEGSQNNRVRKES---------
At1g18330         --------------ND-----------------------------------------------
At5g52660         T------------PRPRS----------------------NRDARDHGN--------------
At5g37260         ---------------------------------------------------------------
At1g01520         K------------QRTRI----------------------VTETNDQES--------------
At4g01280         T------------ERVRA----------------------VTKPNNEES--------------
At3g10113         --------------ND-----------------------------------------------

cons

Cpa_00000217.44   ---------------------------------------------------------------
Cpa_00000769.37   LPSSDDDKAVAAQKLA-S---------------------------------------------
Popu_3444.2       H------VRQG------------------ML--------------------------------
Popu_505.8        ---------------------------------------------------------------
Popu_2025.19      X------XRQH----------------------------------------------------
Popu_2186.6       K------EHFS------------------LL--------------------------------
Popu_2302.6       --------------VGFHRPPT-----------------------------------------
Ot06g02530        ---------------------------------------------------------------
Cre06.g275350.t   HPPQGFPF------FG-LPPSLLAQITLQNPALE--------------------TMSWAG---
kfl00255_0190_v   ---------------------------------------------------------------
kfl00237_0160_v   ---------------------------------------------------------------
Pp1s96_165V6.1    ---------------------------------------------------------------
Pp1s46_272V6.1    ---------------------------------------------------------------
Pp1s198_135V6.1   ---------------------------------------------------------------
Pp1s325_68V6.1    ---------------------------------------------------------------
Pp1s160_6V6.1     ---------------------------------------------------------------
Smo78045|PACid_   ---------------------------------------------------------------
MA_11267g0020     --------VKGSLHSFGPKPKIKIVKSENASGVASTILPSESGTSMLEGSGESNANLPCREIY
MA_115536g0010    --------GNEILHSY-------SLKSHTTAAVN----------SVLHSGSVSTADKDCRE--
MA_102199g0010    ---------------------------------------------------------------
LOC_Os02g46030    ---------------------------------------------------------------
LOC_Os01g06320    ---------------------------------------------------------------
LOC_Os02g45670    ---------------------------------------------------------------
LOC_Os06g01670    ---------------------------------------------------------------
```

```
LOC_Os06g51260     ---------------------------------------------------------------
LOC_Os04g49450     ---------------------------------------------------------------
LOC_Os08g06110     --------LNG-IHIS-------SKPDHS------------------HENCLDTSSQQFKP--
At5g17300          ---------------------------------------------------------------
At3g09600          ---------------------------------------------------------------
At5g02840          ---------------------------------------------------------------
At1g01060          ---------------------------------------------------------------
At2g46830          ---------------------------------------------------------------
At1g18330          ---------------------------------------------------------------
At5g52660          ---------------------------------------------------------------
At5g37260          ---------------------------------------------------------------
At1g01520          ---------------------------------------------------------------
At4g01280          ---------------------------------------------------------------
At3g10113          ---------------------------------------------------------------

cons


Cpa_00000217.44    ---------------------T--------GEPV--RGPDFHRIY-----AFLGSL-------
Cpa_00000769.37    -------PAPG-----------------------AVG-A------------------VPQPSR
Popu_3444.2        ------------------------------------LRE------------------AMI-EQ
Popu_505.8         ---------------------------------------------------------------
Popu_2025.19       --------------------------------------A------------------QLL-HQ
Popu_2186.6        ------------------------------------LTA------------------GLV-LE
Popu_2302.6        --------------------------------------------------------LILS-DT
Ot06g02530         ---------------------------------------------------------------
Cre06.g275350.t    -------GATG-----------------------GTG-GT-------------SGTSVPTHAQ
kf100255_0190_v    ---------------------------------------------------------------
kf100237_0160_v    ---------------------P--------DTPSK-ANPDFAKVY-----RFLGDV-------
Pp1s96_165V6.1     -------------------------------------------------------S-------
Pp1s46_272V6.1     ---------------------P--------ESCIR-AAPDFTEVY-----KFIGSV-------
Pp1s198_135V6.1    ---------------------P--------DSCIR-AAPDFAEVY-----KFIGSV-------
Pp1s325_68V6.1     ---------------------------------------------------------------
Pp1s160_6V6.1      ---------------------P--------ESCIR-AAPDFAEVY-----KFIGSV-------
Smo78045|PACid_    ---------------------G--------IVTLS-AAPAFSEVY-----KFIGSI-------
MA_11267g0020_     TGQQDSDEQLS-ELDNNLNYISREHQASVPMP----------------------IY-------
MA_115536g0010     --KTDSDEQISSELDTNSSCLTRNQESPSIFS----------------------GY-------
MA_102199g0010     -------KDA-----------------------------------------------------
LOC_Os02g46030     ---------------------------------------------------------------
LOC_Os01g06320     --EMATAS-------IVQDETI-------ELPLSP-DDLQFAQVY-----RFIGDI-------
LOC_Os02g45670     ---------------------Q--------VPSLR-LMPDFAQVY-----SFLGSV-------
LOC_Os06g01670     ---------------------M--------LPTLR-AMPDFAQVY-----NFLGSI-------
LOC_Os06g51260     -------EQI-----------------------------------------------------
LOC_Os04g49450     ---------------------------------------------------------------
LOC_Os08g06110     --KSNSVE-------TTYVDWS-------AAKASH-YQMDRNGVTGFQATGTEGSH-------
At5g17300          ---------------TSTYCKSPI---------------------------------------
At3g09600          ---------------------Q--------PPVLH-GVPDFAEVY-----NFIGSV-------
At5g02840          ---------------------Q--------APSMH-GLPDFAEVY-----NFIGSV-------
At1g01060          --------------------------------GTT-VHSMQNYPWHFHADIVNGNI-------
At2g46830          -------N-------SDLNAKS--------LENGN-EQGPQTYPMHIPVLVPLGSS-------
At1g18330          ---------------------------------------------------------------
At5g52660          ---------------------V--------GHSLR-VLPDFAQVY-----GFIGSV-------
At5g37260          ------D------S-------------------------------------------------
At1g01520          ---------------------C--------GKPHR-VAPNFAEVY-----NFIGSV-------
At4g01280          ---------------------C--------EKPHR-VMPNFAEVY-----SFIGSV-------
At3g10113          ---------------------------------------------------------------

cons


Cpa_00000217.44    -------------------------------------------------------FDP-----
Cpa_00000769.37    PVTQRASAQR-----------------------------------------------------
Popu_3444.2        EHQALQ---------------------------------------------------------
Popu_505.8         ---------------------------------------------------------------
Popu_2025.19       EPQHHP---------------------------------------------------------
Popu_2186.6        EQQQLK---------------------------------------------------------
Popu_2302.6        VETREQQQ-------------------------------------------------------
Ot06g02530         ---------------------------------------------------------------
```

```
Cre06.g275350.t   PHANGSAAAGGLSAGGTGIGVG---------------------------ANGGAGSLA-----
kf100255_0190_v   ---------------------------------------------------------------
kf100237_0160_v   -------------------------------------------------------FEI-----
Pp1s96_165V6.1    --------AMM----------------------------------------------------
Pp1s46_272V6.1    -------------------------------------------------------FDP-----
Pp1s198_135V6.1   -------------------------------------------------------FDP-----
Pp1s325_68V6.1    --------AMM----------------------------------------------------
Pp1s160_6V6.1     -------------------------------------------------------FDP-----
Smo78045|PACid_   -------------------------------------------------------FDP-----
MA_11267g0020     --------PRHVPVQRVEGGAH---IPLQRAEGGVQEFAAINT---LNGNGSLSDATEKMKTD
MA_115536g0010    --------PRHVSVQCVVDGAV---SE------------NVNE---VVNMTPLSHSALAMSMS
MA_102199g0010    --------------PCE-DEIL---DC-----------------------------------R
LOC_Os02g46030    -------------S------VE---R--------------ATR---NILVPSFAAAPEGS---
LOC_Os01g06320    -------------------------------------------------------FDP-----
LOC_Os02g45670    -------------------------------------------------------FDP-----
LOC_Os06g01670    -------------------------------------------------------FDP-----
LOC_Os06g51260    --------------SQ---------PS--------------------NEEMLQASS------S
LOC_Os04g49450    -------------DMEVDASAE---T--------------PTS---GTGKFSSHGAAEAN---
LOC_Os08g06110    --------PDQTSDQMGGASGTMNQC------------IHPTLPVDPKFDGNAAAQPF-----
At5g17300         ------------------------------------------------QPLPRKLS-------
At3g09600         -------------------------------------------------------FDP-----
At5g02840         -------------------------------------------------------FDP-----
At1g01060         --------A--KCPQNHPSGMVSQDF------------MFHPMREETHGHANLQATTA-----
At2g46830         -------------------------------------------------------ITS-----
At1g18330         ---------------------------------------------------------------
At5g52660         -------------------------------------------------------FDP-----
At5g37260         ------------------------------------------------------EDGKKK---
At1g01520         -------------------------------------------------------FDP-----
At4g01280         -------------------------------------------------------FDP-----
At3g10113         ---------------------------------------------------------------

cons

Cpa_00000217.44   ---------------------------SQ----R-RRG-------------------------
Cpa_00000769.37   ---------------------------------------------------------------
Popu_3444.2       ---------------------------------------------------------------
Popu_505.8        ---------------------------------------------------------------
Popu_2025.19      --------------------------------------AFISQ--------------------
Popu_2186.6       ---------------------------------------------------------------
Popu_2302.6       ---------------------------------------------------------------
Ot06g02530        ---------------------------------------------------------------
Cre06.g275350.t   ---------------------------AGATGSTTGAAGRPNWDLGTLLW-----PA-PASYD
kf100255_0190_v   ---------------------------------------------------------------
kf100237_0160_v   ---------------------------GV----AGNLQ-------------------------
Pp1s96_165V6.1    -------------------------------------------------------NPQGMLMP
Pp1s46_272V6.1    ---------------------------GV----SGHLR-------------------------
Pp1s198_135V6.1   ---------------------------IV----SGHLR-------------------------
Pp1s325_68V6.1    -------------------------------------------------------NPQGMVMP
Pp1s160_6V6.1     ---------------------------GV----SGHLR-------------------------
Smo78045|PACid_   ---------------------------GT----AGHLK-------------------------
MA_11267g0020     VQ---------------TSQLNVLLPFPGMPGSFNPAYNNLERFGCIPGFIAPGTAPNGSLPP
MA_115536g0010    NN---------------TSSL----------------------------------SKDEIMSP
MA_102199g0010    RIRKDTFSPEESETDTFQSVN----------------------------------NS---TG-
LOC_Os02g46030    -------------------------------------------------------SSNPWPSS
LOC_Os01g06320    ---------------------------DSPCPVETHLQ-------------------------
LOC_Os02g45670    ---------------------------ST----SGHLQ-------------------------
LOC_Os06g01670    ---------------------------ET----SGHLQ-------------------------
LOC_Os06g51260    VG------------EIPAAYC----------------------------------APNGWFMS
LOC_Os04g49450    -------------------------------------------------------TWNPWLTN
LOC_Os08g06110    ---------------------------PHNYAAFAPMMQCHCNQDAYRSF-----AN-MSSTF
At5g17300         ---------------------------------------------------------------
At3g09600         ---------------------------ET----RGHVE-------------------------
At5g02840         ---------------------------DS----KGRMK-------------------------
At1g01060         ---------------------------SATTTASHQAFPACHSQDDYRSF-----LQ-ISSTF
At2g46830         ---------------------------SLSHPPSEPDSHPHTVAGDYQS-------------F
At1g18330         ---------------------------------------------------------------
At5g52660         ---------------------------YA----SNHLQ-------------------------
At5g37260         ---------------------------------------------------------------
```

```
At1g01520          ---------------------------KT----TGHVK-------------------------
At4g01280          ---------------------------NT----SGHLQ-------------------------
At3g10113          ---------------------------------------------------------------

cons

Cpa_00000217.44    ------------------------------------------------------ALQGMSMVD
Cpa_00000769.37    ---------------------------------------------------------------
Popu_3444.2        ---------------------------------------------------------------
Popu_505.8         ---------------------------------------------------------------
Popu_2025.19       ---------------------------------------------------------------
Popu_2186.6        ---------------------------------------------------------------
Popu_2302.6        ---------------------------------------------------------------
Ot06g02530         ---------------------------------------------------------------
Cre06.g275350.t    RHHHLHHHHHHHHHHS--------AERGH---------------------PGTLAEATATTND
kfl00255_0190_v    ---------------------------------------------------------------
kfl00237_0160_v    ------------------------------------------------------KLKCMPPID
Pp1s96_165V6.1     WATAP--------------------------------------------NVQGLENGGAVSAM
Pp1s46_272V6.1     ------------------------------------------------------KLKEMSPID
Pp1s198_135V6.1    ------------------------------------------------------TLKEMAAID
Pp1s325_68V6.1     WASAP--------------------------------------------NAQGLENGGAVSAM
Pp1s160_6V6.1      ------------------------------------------------------KLKEMSAID
Smo78045|PACid_    ------------------------------------------------------KLREMAPID
MA_11267g0020_     WHLTPSAALHHPAYTAATLAAAAFWPGLVSGASPVATGDPQPPREGNNGHSFGEETSAMAAVT
MA_115536g0010     WPSTF----------------SPV---------------------FPWAQMQNGDTAVAEAVA
MA_102199g0010     --KSTI--------HKSSVFSNTN---------------------WSP------ALLNSCHSS
LOC_Os02g46030     MQQFLY--------------------------------------------------FLPRSDG
LOC_Os01g06320     ------------------------------------------------------KLKSMDDII
LOC_Os02g45670     ------------------------------------------------------KLKEMNPID
LOC_Os06g01670     ------------------------------------------------------RLREMDPID
LOC_Os06g51260     YNSFPF--------------------------------------------------QFGESAA
LOC_Os04g49450     TQQFLY--------------------------------------------------YLPNGQI
LOC_Os08g06110     SSMLVSTLLSNPAIHAAARLAASYWPTVD-GNTPD-PN--QENLSESAQGSHAGSPPNMASIV
At5g17300          ------------------------------------------------------SSKTLPIIR
At3g09600          ------------------------------------------------------KLKEMDPIN
At5g02840          ------------------------------------------------------KLKEMDPIN
At1g01060          SNLIMSTLLQNPAAHAAATFAASVWPYASVGNS------------GDSSTPMSSSPPSITAIA
At2g46830          PNHIMSTLLQTPALYTAATFASSFWPPD---SS------------GGSPVPGN-SPPNLAAMA
At1g18330          -----------------------------------------------------------DDLK
At5g52660          ------------------------------------------------------KLKKMDPID
At5g37260          ---------------------------------------------------------------
At1g01520          ------------------------------------------------------RLKEMDPIN
At4g01280          ------------------------------------------------------RLKQMDPIN
At3g10113          -----------------------------------------------------------DDLK

cons

Cpa_00000217.44    RETAHVLMHNLSVN-LSNDQ---IR-HNVVAHLSFHQ------QTIA----------------
Cpa_00000769.37    -------------------------------------------FLS-RQQL---L--------
Popu_3444.2        ---------------------------------------------------------------
Popu_505.8         -------------------------------------------------EI---V--------
Popu_2025.19       ------------------------------------------------HPY---Q--------
Popu_2186.6        ---------------------------------------------------------------
Popu_2302.6        --------------------------------------------QL-PSPC------------
Ot06g02530         ---------------------------------------------------------------
Cre06.g275350.t    AENPSAMLGEDLMG-RLGQM---MG-V---------AGGQESRGSD-GNSV---V--------
kfl00255_0190_v    -------------------------------------------------SF---L--------
kfl00237_0160_v    RETIILLMRNLAIN-LASPV---FA-EQQHILALADS------SARGQQPEE-----------
Pp1s96_165V6.1     AATIAAASAWWAMQ-GGMPP---G-VM---------------HPAL-GAMYLGAAPPLAIPTP
Pp1s46_272V6.1     RETVLLLMRNLSIN-LASPD---FE-EHKLYLSVYVN------KEIKGKSTG-----------
Pp1s198_135V6.1    RETVLLLMRNLSIN-LSSPD---FD-EHKLFMPVIDK------NVIREKPSE-----------
Pp1s325_68V6.1     AATIAAASTWWAMH-GGMPP---G-VM---------------HPAL-GVMYVGAAPPVAISTP
Pp1s160_6V6.1      RETVLLLMHNLSIN-LASPD---FE-EHD----------------------------------
Smo78045|PACid_    RETVLLLMRNLAIN-LSSPD---FD-QRVKFA-------------------------------
MA_11267g0020_     AATVAAASAWWTLH-GAIPP---P-YL---------------HPGI-YGPV---V--------
MA_115536g0010     VATVAAASAWWSLY-GAVPS---L--L---------------HPRM-F------S--------
```

```
MA_102199g0010    SSENENIQCSYPQV-LRDGSLEDGLQQ---------------KPEV-P------D--------
LOC_Os02g46030    FA-AQPVMPWLSYN-GSLPC---ALFY----------------PAA-A------A--------
LOC_Os01g06320    VKTILLVLRNLEDN-LLSPQ---FE-P------------------------------------
LOC_Os02g45670    VETALLLMRNLSIN-LTSPD---FE-DQKKLLSSYST------PSD-----------------
LOC_Os06g01670    VETVLLLMKNLSIN-LTNPN---FE-AHRKVLASHGY------GMD-----------------
LOC_Os06g51260    DARIPPLHVWWPYY-GFAP------IS---------------HPRG-L------S--------
LOC_Os04g49450    FS-VHSALPCFTYHNEGVTC---TQFS---------------NPQV-V------A--------
LOC_Os08g06110    TATVAAASAWWATQ-GLLPL---FP-P---------------P--I-AFPF---V--------
At5g17300         NSQEELLSC------------------------------------------------------
At3g09600         FETVLLLMRNLTVN-LSNPD---LE-STRKVLLSYDN------VTT-----------------
At5g02840         FETVLLLMRNLTVN-LSNPD---FE-PTSEYVDAAEE------GHE-----------------
At1g01060         AATVAAATAWWASH-GLLPV---CA-P---------------APIT-CVPF---S--------
At2g46830         AATVAAASAWWAAN-GLLPL---CA-P---------------L--S-SGGF---T--------
At1g18330         QMTCQENHYSGMLV-DTNL-----------------------------------S--------
At5g52660         VETVLLLMRNLSIN-LSSPD---FE-DHRRLLSSYDI------GSE-----------------
At5g37260         ---------------------------------------------------------------
At1g01520         LETVLLLMKNLSVN-LTSPE---FD-EQRKLISSYNA---------S----------------
At4g01280         METVLLLMQNLSVN-LTSPE---FA-EQRRLISSYSA------KALK----------------
At3g10113         QMTCQENHYSGMLV-DTNL-----------------------------------S--------

cons

Cpa_00000217.44   ---------------------MNQ---------------------------------------
Cpa_00000769.37   ---------------------------------------------------------------
Popu_3444.2       ---------------------------------------------------------------
Popu_505.8        ------------------H--------------------------------------------
Popu_2025.19      ------------------N--------------------------------------------
Popu_2186.6       ---------------------------------------------------------------
Popu_2302.6       ---------------------------------------------------------------
Ot06g02530        ---------------------------------------------------------------
Cre06.g275350.t   ------------------TEREGEQ--GG----------------------------------
kfl00255_0190_v   ------------------E--------------------------------------------
kfl00237_0160_v   -------------------PAQPRMIEPE----------------------------------
Pp1s96_165V6.1    YIFSQCVPQEITVAAAPC------A--DGNEVPTAQVVDDGQEVLSNNNHSRSSAMRRAERAQ
Pp1s46_272V6.1    ------------------TQDLTS--APS----------------------------------
Pp1s198_135V6.1   ------------------AEDSTS--IPG----------------------------------
Pp1s325_68V6.1    YIFPQCSPQEVTLAGVPC------A--DGKEDLTVHAVSEGENIPINGN-SRSSAMRRAERAQ
Pp1s160_6V6.1     ---------------------------------------------------------------
Smo78045|PACid_   ---------------------------------------------------------------
MA_11267g0020     ---AAMAAEAAAVAAAAA------A--SGNSSPLCK----------------TEP--------
MA_115536g0010    ---------------GAT------A--AENISSVCK----------------AET--------
MA_102199g0010    -----------------I------K--CK----------------------------------
LOC_Os02g46030    -----------------A------A--NQ----------------------------------
LOC_Os01g06320    -----------------I---------------------------------------------
LOC_Os02g45670    ---------------------------------------------------------------
LOC_Os06g01670    ---------------------------------------------------------------
LOC_Os06g51260    -----------------T------V--MQ----------------------------------
LOC_Os04g49450    -----------------S------D--QQ----------------------------------
LOC_Os08g06110    ---PA-------PSAPFSTADVQRA--QE----------------------------------
At5g17300         ---------------------------------------------------------------
At3g09600         ---------------------------------------------------------------
At5g02840         ---------------------------------------------------------------
At1g01060         ------------------TVAVPTP--AM----------------------------------
At2g46830         ----------------SHPPSTFGP--SC----------------------------------
At1g18330         -----------------L------G--V-----------------------------------
At5g52660         ---------------------------------------------------------------
At5g37260         ---------------------------------------------------------------
At1g01520         ---------------------------------------------------------------
At4g01280         ---------------------------------------------------------------
At3g10113         -----------------L------G--V-----------------------------------

cons

Cpa_00000217.44   ---------------------------------------------------------------
Cpa_00000769.37   ---------------------------------------------------------------
Popu_3444.2       ---------------------------------------------------------------
```

```
Popu_505.8          ---------------------------------------------------------------
Popu_2025.19        ---------------------------------------------------------------
Popu_2186.6         ---------------------------------------------------------------
Popu_2302.6         ---------------------------------------------------------------
Ot06g02530          ---------------------------------------------------------------
Cre06.g275350.t     ---------------------------------------------------------------
kfl00255_0190_v     ---------------------------------------------------------------
kfl00237_0160_v     ---------------------------------------------------------------
Pp1s96_165V6.1      RVKELKARRMGSGNHLLSVSSDICASTSGGSGTNLTTQLTAGGLTSESAVNSLSKGSSLPADL
Pp1s46_272V6.1      ---------------------------------------------------------------
Pp1s198_135V6.1     ---------------------------------------------------------------
Pp1s325_68V6.1      LVKELKARRTGSGINFLSVSSDICGSTSGGSGTNLTTQLTAGGLTSDSAVNSLSKGGSL--DA
Pp1s160_6V6.1       ---------------------------------------------------------------
Smo78045|PACid_     ---------------------------------------------------------------
MA_11267g0020       ---------------------------------------------------------------
MA_115536g0010      ---------------------------------------------------------------
MA_102199g0010      ---------------------------------------------------------------
LOC_Os02g46030      ---------------------------------------------------------------
LOC_Os01g06320      ---------------------------------------------------------------
LOC_Os02g45670      ---------------------------------------------------------------
LOC_Os06g01670      ---------------------------------------------------------------
LOC_Os06g51260      ---------------------------------------------------------------
LOC_Os04g49450      ---------------------------------------------------------------
LOC_Os08g06110      ---------------------------------------------------------------
At5g17300           ---------------------------------------------------------------
At3g09600           ---------------------------------------------------------------
At5g02840           ---------------------------------------------------------------
At1g01060           ---------------------------------------------------------------
At2g46830           ---------------------------------------------------------------
At1g18330           ---------------------------------------------------------------
At5g52660           ---------------------------------------------------------------
At5g37260           ---------------------------------------------------------------
At1g01520           ---------------------------------------------------------------
At4g01280           ---------------------------------------------------------------
At3g10113           ---------------------------------------------------------------

cons


Cpa_00000217.44     --------------------------------------QILAG--------QPVTINPSVELS
Cpa_00000769.37     ---------------------------------------------------------------
Popu_3444.2         ---------------------------------------------------------------
Popu_505.8          ---------------------------------------------------------------
Popu_2025.19        -----------------------------DR--------------------------------
Popu_2186.6         ---------------------------------------------------------------
Popu_2302.6         ---------------------------------------------------------------
Ot06g02530          ---------------------------------------------------------------
Cre06.g275350.t     -----------------------GGG-GGAVA--G-------DD--GPEAEETGQDACAEDAG
kfl00255_0190_v     -----------------------------SG--------------------------------
kfl00237_0160_v     -----------------------S---QSAPPTPSDASVTPGEQGNLVATGAPFLDSPGYRDT
Pp1s96_165V6.1      SKVKHVLNGLIRKDREAPVDGCSADD-GVDNRKRLRGSRPSDDTAMPDGEGYHSTSGLGREAP
Pp1s46_272V6.1      -----------------------AAA-Q-SSSDTGEDTSSVSGQAGTPQS-NALLVSPRSGDA
Pp1s198_135V6.1     -----------------------AVP-QPCSQDAGEATTCVSGQGGTPAPSTPLLVGPRSGDS
Pp1s325_68V6.1      SKVKHGLHSFVRKDREVPADGGSADD-GDDKRKRLRVSRPSDDTIMHDNEGYPSTTSLARDAP
Pp1s160_6V6.1       ---------------------------------AGEDSPSVSGQAGTLAPSTPILVSPRSGNS
Smo78045|PACid_     ---------------------------------------------------------------
MA_11267g0020       ----------------------VREARKHEKMITTE-EDG-----------SDPNITSCNEES
MA_115536g0010      ----------------------VGGVREQSQSNTTE-REGETLL--QGSQ-SENYTSSLIQES
MA_102199g0010      -----------------------YTH-QSQQEDEK----------------------------
LOC_Os02g46030      -----------------------QCH-RDSEG-------------------------------
LOC_Os01g06320      ---------------------------------------------------------------
LOC_Os02g45670      ---------------------------------------------------------------
LOC_Os06g01670      ---------------------------------------------------------------
LOC_Os06g51260      -----------------------QTE-GSDESD------------------------------
LOC_Os04g49450      -----------------------HQH-QTSEA-------------------------------
LOC_Os08g06110      -----------------------KDID-CPMDNAQKELQETRKQ--DNFEAMKVIVSSETDES
At5g17300           -------------------------------------------------------WIQVPLKQ
At3g09600           ---------------------------------------------------------------
At5g02840           ---------------------------------------------------------------
```

```
At1g01060           -----------------------TEMD-TVENTQPFEKQNTALQ--DQNLASKSPASSSDDSD
At2g46830           -----------------------DVEY-TKASTLQHGSVQSREQ--EHSEASKARSSLDSED-
At1g18330           ---------------------------------------------------------------
At5g52660           ---------------------------------------------------------------
At5g37260           ---------------------------------------------------------------
At1g01520           ---------------------------------------------------------------
At4g01280           ---------------------------------------------------------------
At3g10113           ---------------------------------------------------------------

cons


Cpa_00000217.44     RLYQPKYDRCRCRQ-RRSLDALSAR------------P-------------------------
Cpa_00000769.37     ---------------------------------------------------------------
Popu_3444.2         ---------------------------------------------------------------
Popu_505.8          ---------------------------------------------------------------
Popu_2025.19        ---------------------------------------------------------------
Popu_2186.6         ---------------------------------------------------------------
Popu_2302.6         -----------------------------------------------------SGSW------
Ot06g02530          ---------------------------------------------------------------
Cre06.g275350.t     QGAQSNLTDDGQWA-KLGPGAELEQQLAAGGAMATSKY--WHMLGAAAGHPP-RDGHG-----
kfl00255_0190_v     ---------------------------------------------------------------
kfl00237_0160_v     VQQQQQSPLYEQGG-RGVTRASSF----------ADGV--PLGLGLGLG--------------
Pp1s96_165V6.1      GSMDSHLEKVRSVS-GSNSIPGKFTKVDVERLACDEKT--APTQASCGSDVS-EGAGVAPID-
Pp1s46_272V6.1      DHTQQRASLLLAYS-DNTADAGTPRRHSYDGGGAISAS--YTSSGVSPS-PG-DL--------
Pp1s198_135V6.1     DHSQQKASLLQTFA-DNTTDAETPRRHSYDGGGTMSAS--YTSCCVSPASPG-IL--------
Pp1s325_68V6.1      GRLNAHLEKVRSVS-GSSTLPGKFTKVGAERLVYDEEN--TPTDASCGSDVS-EGAGVAPT--
Pp1s160_6V6.1       APTQHRASLLQAYA-DSTADAGTARRHSHDGGRVISAS--FTSCGVSPA-PE-VL--------
Smo78045|PACid_     -----DKRFFRSFQRFCFSGLSRDRSCSFQSTKRRKTE--WILTTSNKVVMK-KQ--------
MA_11267g0020       CDVEMQIENQNCQV-EEQDEIALCINLPDGENTNSRKV--IKERSSSGSNTP-SSADPEIDP-
MA_115536g0010      NDARRHAEKQSCQT-EEEDQIMKET-----------EL--GKERSQCASNT-PSSNDPEIDP-
MA_102199g0010      ---Q---------------------------------R--NKDKIGLEAQP-GN-------I-
LOC_Os02g46030      --V------------------------------EFRVS--QREGSLTGSNTA-SSVVLGSSAA
LOC_Os01g06320      ---------------------------------------------------------------
LOC_Os02g45670      -------------------G---LELGSTRSSVLADRP--LSAPFMIKG--------------
LOC_Os06g01670      -------------------QVKHENLG------DLGSTHTLHLPF------------------
LOC_Os06g51260      ---G---------------------------------V--KSHSSESSSDS-GE-------N-
LOC_Os04g49450      --V------------------------------DYKGI--QREGSWTESNTS-SS-------S
LOC_Os08g06110      GKGEVSLHTELKIS-PADKADTKPAAGAETSDVFGNKK--KQDRSSCGSNTP-SSS-------
At5g17300           ED------------------------VENRCLDSGKAV--QNEGSSTGSNTG-SVDD------
At3g09600           -------------------ELPSVVSL-----VKNSTS----DKS------------------
At5g02840           -------------------HLS-----------------------------------------
At1g01060           ETGVTKLNADSKTN-DDKIEEVVVTAAVHDSNTAQKKN--LVDRSSCGSNTP-SGS-------
At2g46830           ------VENKSKPV-CHEQPSATPESDAKGSDGAGDRK--QVDRSSCGSNTP-SSSD------
At1g18330           -----------------------------------------WETFCTGSNAFGS---------
At5g52660           -------------------T--ATDHGGVNKTLNKDPP--EIS--------------------
At5g37260           ----LYSETQS---------------------------------L------------------
At1g01520           ---------------------------------------------------------------
At4g01280           ---------------------------------------------------------------
At3g10113           -----------------------------------------WETFCTGSNAFGS---------

cons


Cpa_00000217.44     ---------------------------------------------------------------
Cpa_00000769.37     ---------------------------------------------------------------
Popu_3444.2         ---------------------------------------------------------------
Popu_505.8          ---------------------------------------------------------------
Popu_2025.19        ---------------------------------------------------------------
Popu_2186.6         ---------------------------------------------------------------
Popu_2302.6         ---------------------------------------------------------------
Ot06g02530          ---------------------------------------------------------------
Cre06.g275350.t     ---------------------------------------------------------------
kfl00255_0190_v     ---------------------------------------------------------------
kfl00237_0160_v     ---------------------------------------------------------------
Pp1s96_165V6.1      GDGDGDASGGGPSSNSSACGNVPGGNERSGSSDGSSEGEATCNVDRQR--------EAGK---
Pp1s46_272V6.1      ---------------------------------------------------------------
Pp1s198_135V6.1     ---------------------------------------------------------------
```

```
Pp1s325_68V6.1     -DGDGNASGGGPSSNSSACGNALGGNDRSGSSDGSSEGEGTCKVNRQR--------DTGK---
Pp1s160_6V6.1      ---------------------------------------------------------------
Smo78045|PACid_    ---------------------------------------------------------------
MA_11267g0020      C---------------------------LGTNT---EVKGNSERDFEFNWVGSGSSDAGGEEA
MA_115536g0010     Y---------------------------SGTNI------------------------------
MA_102199g0010     V-------------------LQS--NPKPTPLM------------------------------
LOC_Os02g46030     V-------------------PAAAAAAQNSDVA------------------------------
LOC_Os01g06320     ---------------------------------------------------------------
LOC_Os02g45670     ---------------------------------------------------------------
LOC_Os06g01670     ---------------------------------------------------------------
LOC_Os06g51260     V-------------------QMT--APQSSRIV------------------------------
LOC_Os04g49450     V-------------------PETAT--HNSETT------------------------------
LOC_Os08g06110     ---------------------------------------------------------------
At5g17300          ---------------------------------------------------------------
At3g09600          ---------------------------------------------------------------
At5g02840          ---------------------------------------------------------------
At1g01060          ---------------------------------------------------------------
At2g46830          ---------------------------------------------------------------
At1g18330          ---------------------------------------------------------------
At5g52660          ---------------------------------------------------------------
At5g37260          ---------------------------------------------------------------
At1g01520          ---------------------------------------------------------------
At4g01280          ---------------------------------------------------------------
At3g10113          ---------------------------------------------------------------

cons


Cpa_00000217.44    ---------------------------------------------------------------
Cpa_00000769.37    ----------------------------AHGGER------TR------------RDSP---DG
Popu_3444.2        ---------------------------------------------------------------
Popu_505.8         ------------------------------AAAVRAASACMP---------------T---SY
Popu_2025.19       -----------------------------PSSGF------PSL------------PSH---GA
Popu_2186.6        -----------------------------R---------------------------------
Popu_2302.6        -----------------------------TAAHD------FQA------------TEV---S-
Ot06g02530         ---------------------------------------------------------------
Cre06.g275350.t    ----------------GVGLGPITHKSHSHGGTR------TTGSGGGVRAKAGAGAKA---HA
kf100255_0190_v    -----------------------------VSSA------VIE---------------GDATDS
kf100237_0160_v    ---------------------------------------------------------------
Pp1s96_165V6.1     ---NGTS---SSDPEAEEEADEKVGRAAEQ-FTF------NEFL--PV---------------
Pp1s46_272V6.1     ---------------------------------------------------------------
Pp1s198_135V6.1    ---------------------------------------------------------------
Pp1s325_68V6.1     ---NGTS---SSDPEAQLKTDEEVGRATGL-ITF------NEFL--PV---------------
Pp1s160_6V6.1      ---------------------------------------------------------------
Smo78045|PACid_    ---------------------------------------------------------------
MA_11267g0020      LAKKRTNCIFPEAPGKTEREKNNLFTKQDG-KMD------YNEN--YV---------------
MA_115536g0010     ---------QENTRNCVQSLQRNGGGYIPE-AEE------GSSI--DE---------------
MA_102199g0010     --------------------E------------------------------------------
LOC_Os02g46030     --------------------ES-----------------------------------------
LOC_Os01g06320     ---------------------------------------------------------------
LOC_Os02g45670     ---------------------------------------------------------------
LOC_Os06g01670     ---------------------------------------------------------------
LOC_Os06g51260     --------------------ESL----------------------------------------
LOC_Os04g49450     --------------------ES-----------------------------------------
LOC_Os08g06110     ------------D---IE-----ADNAPEN-QEK------ANDK--AK---------------
At5g17300          ---------------------------------------------------------------
At3g09600          ---------------------------------------------------------------
At5g02840          ---------------------------------------------------------------
At1g01060          ------------D---AETDALDKMEKDKE-DVK------ETDE--NQ---------------
At2g46830          ------------D---VEADA---SERQED-GTN------GEVK--ET---------------
At1g18330          ----------------------V----------------------------------------
At5g52660          ---------------------------------------------------------------
At5g37260          ---------------------------------------------------------------
At1g01520          ---------------------------------------------------------------
At4g01280          ---------------------------------------------------------------
At3g10113          ----------------------V----------------------------------------

cons
```

```
Cpa_00000217.44    ---------------------------------------------------------------
Cpa_00000769.37    HQQVPEQQERAFSPSAPRSSRPARPR-------------------------------------
Popu_3444.2        ---------------------------------------------------------------
Popu_505.8         E--------------------------------------------------------------
Popu_2025.19       R--------------------------------------------------------------
Popu_2186.6        ---------------------------------------------------------------
Popu_2302.6        -------------QN--LQQNEQQ---------------------------------------
Ot06g02530         ---------------------------------------------------------------
Cre06.g275350.t    HKEARDQ-------HKKSSARDASPRRYSDVSGHVRGGHHHRDRDLLAHPHSSHHHVPPSPSI
kf100255_0190_v    Y--------------------------------------------------------------
kf100237_0160_v    ---------------------------------------------------------------
Pp1s96_165V6.1     ---------------------------------------------------------------
Pp1s46_272V6.1     ---------------------------------------------------------------
Pp1s198_135V6.1    ---------------------------------------------------------------
Pp1s325_68V6.1     ---------------------------------------------------------------
Pp1s160_6V6.1      ---------------------------------------------------------------
Smo78045|PACid_    ---------------------------------------------------------------
MA_11267g0020      ---------------------------------------------------------------
MA_115536g0010     ---------------------------------------------------------------
MA_102199g0010     ---------------------------------------------------------------
LOC_Os02g46030     ---------------------------------------------------------------
LOC_Os01g06320     ---------------------------------------------------------------
LOC_Os02g45670     ---------------------------------------------------------------
LOC_Os06g01670     ---------------------------------------------------------------
LOC_Os06g51260     ---------------------------------------------------------------
LOC_Os04g49450     ---------------------------------------------------------------
LOC_Os08g06110     ---------------------------------------------------------------
At5g17300          ---------------------------------------------------------------
At3g09600          ---------------------------------------------------------------
At5g02840          ---------------------------------------------------------------
At1g01060          ---------------------------------------------------------------
At2g46830          ---------------------------------------------------------------
At1g18330          ---------------------------------------------------------------
At5g52660          ---------------------------------------------------------------
At5g37260          ---------------------------------------------------------------
At1g01520          ---------------------------------------------------------------
At4g01280          ---------------------------------------------------------------
At3g10113          ---------------------------------------------------------------

cons


Cpa_00000217.44    ---------------------------------------------------------------
Cpa_00000769.37    ---------------------------------------------------------------
Popu_3444.2        ---------------------------------------------------------------
Popu_505.8         --------TKSVKGA-----LSEGGAPGQQMPT------------------------------
Popu_2025.19       --------GDGAASS-----LNDL--------APMSSS-----------------L-SGRS--
Popu_2186.6        ---------------------------------------------------------------
Popu_2302.6        --------------------QALL--------IDENL------------------QVNAQQPS
Ot06g02530         --------------------------------HQ-----------------------------
Cre06.g275350.t    QEHAGGLKRDAGGRHVASSDDTNG--------SDSQQPPNTETREAQGSNPTNHNNGNGNGSS
kf100255_0190_v    --------APSTSGR-----VFP--------SQSHSKD-----------------L-HRQ--Q
kf100237_0160_v    ---------------------------------------------------------------
Pp1s96_165V6.1     ---------------------------------------------------------------
Pp1s46_272V6.1     ---------------------------------------------------------------
Pp1s198_135V6.1    ---------------------------------------------------------------
Pp1s325_68V6.1     ---------------------------------------------------------------
Pp1s160_6V6.1      ---------------------------------------------------------------
Smo78045|PACid_    ---------------------------------------------------------------
MA_11267g0020      ---------------------------------------------------------------
MA_115536g0010     ---------------------------------------------------------------
MA_102199g0010     ---------------------------------------------------------------
LOC_Os02g46030     ---------------------------------------------------------------
LOC_Os01g06320     ---------------------------------------------------------------
LOC_Os02g45670     ---------------------------------------------------------------
LOC_Os06g01670     ---------------------------------------------------------------
LOC_Os06g51260     ---------------------------------------------------------------
LOC_Os04g49450     ---------------------------------------------------------------
```

```
LOC_Os08g06110     ---------------------------------------------------------------
At5g17300          ---------------------------------------------------------------
At3g09600          ---------------------------------------------------------------
At5g02840          ---------------------------------------------------------------
At1g01060          ---------------------------------------------------------------
At2g46830          ---------------------------------------------------------------
At1g18330          ---------------------------------------------------------------
At5g52660          ---------------------------------------------------------------
At5g37260          ---------------------------------------------------------------
At1g01520          ---------------------------------------------------------------
At4g01280          ---------------------------------------------------------------
At3g10113          ---------------------------------------------------------------

cons


Cpa_00000217.44    ---------------------------------------------------------------
Cpa_00000769.37    ---------------------------------------------------------------
Popu_3444.2        ---------------------------------------------------------------
Popu_505.8         --------------------------ARFSAPPII----------------------------
Popu_2025.19       -----------GRKSAHRKNR------------------------------------------
Popu_2186.6        ---------------------------------------------------------------
Popu_2302.6        ---------------QHLPQYGPL---------------------------------------
Ot06g02530         -----------------ELRQG-----YFGIPTGM----------------------------
Cre06.g275350.t    GNGSAGNGNGSGSQGNGSQGNGNNGSSGNGHSGGNGHSGGNGHSGGNGHSGGNGHSGGSGGAG
kfl00255_0190_v    SSSQR-----------GDSKDF-AFAAPPPKGSAP----------------------------
kfl00237_0160_v    ---------------------------------------------------------------
Pp1s96_165V6.1     ---------------------------------------------------------------
Pp1s46_272V6.1     ---------------------------------------------------------------
Pp1s198_135V6.1    ---------------------------------------------------------------
Pp1s325_68V6.1     ---------------------------------------------------------------
Pp1s160_6V6.1      ---------------------------------------------------------------
Smo78045|PACid_    ---------------------------------------------------------------
MA_11267g0020      ---------------------------------------------------------------
MA_115536g0010     ---------------------------------------------------------------
MA_102199g0010     ---------------------------------------------------------------
LOC_Os02g46030     ---------------------------------------------------------------
LOC_Os01g06320     ---------------------------------------------------------------
LOC_Os02g45670     ---------------------------------------------------------------
LOC_Os06g01670     ---------------------------------------------------------------
LOC_Os06g51260     ---------------------------------------------------------------
LOC_Os04g49450     ---------------------------------------------------------------
LOC_Os08g06110     ---------------------------------------------------------------
At5g17300          ---------------------------------------------------------------
At3g09600          ---------------------------------------------------------------
At5g02840          ---------------------------------------------------------------
At1g01060          ---------------------------------------------------------------
At2g46830          ---------------------------------------------------------------
At1g18330          ---------------------------------------------------------------
At5g52660          ---------------------------------------------------------------
At5g37260          ---------------------------------------------------------------
At1g01520          ---------------------------------------------------------------
At4g01280          ---------------------------------------------------------------
At3g10113          ---------------------------------------------------------------

cons


Cpa_00000217.44    ---------------------------------------------------------------
Cpa_00000769.37    ---------------------------------------------------------------
Popu_3444.2        ---------------------------------------------------------------
Popu_505.8         ---------------------------------------------------------------
Popu_2025.19       ---------------------------------------------------------------
Popu_2186.6        ---------------------------------------------------------------
Popu_2302.6        ---------------------------------------------------------------
Ot06g02530         ---------------------------------------------------------------
Cre06.g275350.t    GSGGNGNGHSIKDSNNPTGNGNGNGYSSANGTSHHHHLHPHQHLHGLQRMPAYDAGRYPSHHE
kfl00255_0190_v    -------------------------------------------------------------AG
```

```
kfl00237_0160_v    ---------------------------------------------------------------
Pp1s96_165V6.1     ---------------------------------------------------------------
Pp1s46_272V6.1     ---------------------------------------------------------------
Pp1s198_135V6.1    ---------------------------------------------------------------
Pp1s325_68V6.1     ---------------------------------------------------------------
Pp1s160_6V6.1      ---------------------------------------------------------------
Smo78045|PACid_    ---------------------------------------------------------------
MA_11267g0020      ---------------------------------------------------------------
MA_115536g0010     ---------------------------------------------------------------
MA_102199g0010     ---------------------------------------------------------------
LOC_Os02g46030     ---------------------------------------------------------------
LOC_Os01g06320     ---------------------------------------------------------------
LOC_Os02g45670     ---------------------------------------------------------------
LOC_Os06g01670     ---------------------------------------------------------------
LOC_Os06g51260     ---------------------------------------------------------------
LOC_Os04g49450     ---------------------------------------------------------------
LOC_Os08g06110     ---------------------------------------------------------------
At5g17300          ---------------------------------------------------------------
At3g09600          ---------------------------------------------------------------
At5g02840          ---------------------------------------------------------------
At1g01060          ---------------------------------------------------------------
At2g46830          ---------------------------------------------------------------
At1g18330          ---------------------------------------------------------------
At5g52660          ---------------------------------------------------------------
At5g37260          ---------------------------------------------------------------
At1g01520          ---------------------------------------------------------------
At4g01280          ---------------------------------------------------------------
At3g10113          ---------------------------------------------------------------

cons


Cpa_00000217.44    ---------------------------------------------------------------
Cpa_00000769.37    ---------------------------------------------------------------
Popu_3444.2        ---------------------------------------------------------------
Popu_505.8         ------------------------------------KSQYSH--------------TGISPW-
Popu_2025.19       ------------------------------------------------------AEMEL--A-
Popu_2186.6        ---------------------------------------------------------------
Popu_2302.6        ------------EYHEAAQGQGLVDEELWA--------------------------LF-----
Ot06g02530         ------------------------------------QPQQGM-------------------F-
Cre06.g275350.t    TQDREGNGASGIGVRPPSGSDEDDNNGLNGLNGSGGAGGGAAAGDAAGGSGSGGAGAEVGAS-
kfl00255_0190_v    ------------------------------------RPSQMP-------------------AT
kfl00237_0160_v    ---------------------------------------------------------------
Pp1s96_165V6.1     ---------------------------------------------------------------
Pp1s46_272V6.1     ---------------------------------------------------------------
Pp1s198_135V6.1    ---------------------------------------------------------------
Pp1s325_68V6.1     ---------------------------------------------------------------
Pp1s160_6V6.1      ---------------------------------------------------------------
Smo78045|PACid_    ---------------------------------------------------------------
MA_11267g0020      ---------------------------------------------------------------
MA_115536g0010     ---------------------------------------------------------------
MA_102199g0010     ---------------------------------------------------------------
LOC_Os02g46030     ---------------------------------------------------------------
LOC_Os01g06320     ---------------------------------------------------------------
LOC_Os02g45670     ---------------------------------------------------------------
LOC_Os06g01670     ---------------------------------------------------------------
LOC_Os06g51260     ---------------------------------------------------------------
LOC_Os04g49450     ---------------------------------------------------------------
LOC_Os08g06110     ---------------------------------------------------------------
At5g17300          ---------------------------------------------------------------
At3g09600          ---------------------------------------------------------------
At5g02840          ---------------------------------------------------------------
At1g01060          ---------------------------------------------------------------
At2g46830          ---------------------------------------------------------------
At1g18330          ---------------------------------------------------------------
At5g52660          ---------------------------------------------------------------
At5g37260          ---------------------------------------------------------------
At1g01520          ---------------------------------------------------------------
At4g01280          ---------------------------------------------------------------
```

```
At3g10113          ---------------------------------------------------------------

cons


Cpa_00000217.44    ---------------------------------------------------------------
Cpa_00000769.37    ---------------------------------------------------------------
Popu_3444.2        ----AQQQQRAIGCDIGVGSGAVPWTMYQPSQPTAAPSSSGSPFSMKGTPAYTVSFAG-----
Popu_505.8         ---------------------------------------------------------------
Popu_2025.19       QAPTSKRSKSSVGSRIST-AG-----------------NASTSSDMQGFEMRLSDIGG-----
Popu_2186.6        ----GVTARKEASQVLSQESGV-----------------------------------------
Popu_2302.6        ---------------------------------------------------------------
Ot06g02530         ---------------------------------------------------------------
Cre06.g275350.t    AAPDGS-----------------------RSAGGSGPSASGKAGGIKGAGVSGSDGSGGAGGK
kf100255_0190_v    Q--------------------Q----------T-------PP---------------------
kf100237_0160_v    ---------------------------------------------------------------
Pp1s96_165V6.1     ---------------------------------------------------------------
Pp1s46_272V6.1     ---------------------------------------------------------------
Pp1s198_135V6.1    ---------------------------------------------------------------
Pp1s325_68V6.1     ---------------------------------------------------------------
Pp1s160_6V6.1      ---------------------------------------------------------------
Smo78045|PACid_    ---------------------------------------------------------------
MA_11267g0020      ---------------------------------------------------------------
MA_115536g0010     ---------------------------------------------------------------
MA_102199g0010     ---------------------------------------------------------------
LOC_Os02g46030     ---------------------------------------------------------------
LOC_Os01g06320     ---------------------------------------------------------------
LOC_Os02g45670     ---------------------------------------------------------------
LOC_Os06g01670     ---------------------------------------------------------------
LOC_Os06g51260     ---------------------------------------------------------------
LOC_Os04g49450     ---------------------------------------------------------------
LOC_Os08g06110     ---------------------------------------------------------------
At5g17300          ---------------------------------------------------------------
At3g09600          ---------------------------------------------------------------
At5g02840          ---------------------------------------------------------------
At1g01060          ---------------------------------------------------------------
At2g46830          ---------------------------------------------------------------
At1g18330          ---------------------------------------------------------------
At5g52660          ---------------------------------------------------------------
At5g37260          ---------------------------------------------------------------
At1g01520          ---------------------------------------------------------------
At4g01280          ---------------------------------------------------------------
At3g10113          ---------------------------------------------------------------

cons


Cpa_00000217.44    ---------------------------------------------------------------
Cpa_00000769.37    ------------------------------------AVAFSPAPFEACAPPRRPRHRAPPPQH
Popu_3444.2        -------TD------------------AGSQPSMTAL--MEA------------------S--
Popu_505.8         ---------------------------------------------------------------
Popu_2025.19       -------SS------------------TGLPPSGTTHSFGDA----------------ASL--
Popu_2186.6        ---------------------------------------------------------------
Popu_2302.6        ---------------------------------------------------------RSVDEH
Ot06g02530         ---------------------------------------------------------------
Cre06.g275350.t    AAKGGSGDGRSGSLGSGAAGFRRPAEGTLALGLLATAPDRPVGQIAGSAFSVPAPSITRGGNG
kf100255_0190_v    ---------------------------------------FSK-----------------PEMA
kf100237_0160_v    ---------------------------------------------------------------
Pp1s96_165V6.1     ---------------------------------------------------------------
Pp1s46_272V6.1     ---------------------------------------------------------------
Pp1s198_135V6.1    ---------------------------------------------------------------
Pp1s325_68V6.1     ---------------------------------------------------------------
Pp1s160_6V6.1      ---------------------------------------------------------------
Smo78045|PACid_    ---------------------------------------------------------------
MA_11267g0020      ---------------------------------------------------------------
MA_115536g0010     ---------------------------------------------------------------
MA_102199g0010     ---------------------------------------------------------------
LOC_Os02g46030     ---------------------------------------------------------------
```

```
LOC_Os01g06320     ---------------------------------------------------------------
LOC_Os02g45670     ---------------------------------------------------------------
LOC_Os06g01670     ---------------------------------------------------------------
LOC_Os06g51260     ---------------------------------------------------------------
LOC_Os04g49450     ---------------------------------------------------------------
LOC_Os08g06110     ---------------------------------------------------------------
At5g17300          ---------------------------------------------------------------
At3g09600          ---------------------------------------------------------------
At5g02840          ---------------------------------------------------------------
At1g01060          ---------------------------------------------------------------
At2g46830          ---------------------------------------------------------------
At1g18330          ---------------------------------------------------------------
At5g52660          ---------------------------------------------------------------
At5g37260          ---------------------------------------------------------------
At1g01520          ---------------------------------------------------------------
At4g01280          ---------------------------------------------------------------
At3g10113          ---------------------------------------------------------------

cons


Cpa_00000217.44    ---------------------------------------------------------------
Cpa_00000769.37    GGLAPGSTFSEDECLKKGDAFHYDSDSD-----------------------------------
Popu_3444.2        --RMPT--LL-----------------------------------------------------
Popu_505.8         ---------------------------------------------------------------
Popu_2025.19       --RGDTPPGF-----------------------------------------------------
Popu_2186.6        ---------------------------------------------------------------
Popu_2302.6        GGLVPQMSAT-----------------------------------------------------
Ot06g02530         ---------------------------------------------------------------
Cre06.g275350.t    NTGASGNVANAAAAAGTGGSNDGAGATHGGSGSGGNGGAGGSSGGSVAGAAAVAAVLEVSQVQ
kf100255_0190_v    FSQ-APAPA------------------------------------------------------
kf100237_0160_v    ---------------------------------------------------------------
Pp1s96_165V6.1     ---------------------------------------------------------------
Pp1s46_272V6.1     ---------------------------------------------------------------
Pp1s198_135V6.1    ---------------------------------------------------------------
Pp1s325_68V6.1     ---------------------------------------------------------------
Pp1s160_6V6.1      ---------------------------------------------------------------
Smo78045|PACid_    ---------------------------------------------------------------
MA_11267g0020      ---------------------------------------------------------------
MA_115536g0010     ---------------------------------------------------------------
MA_102199g0010     ---------------------------------------------------------------
LOC_Os02g46030     ---------------------------------------------------------------
LOC_Os01g06320     ---------------------------------------------------------------
LOC_Os02g45670     ---------------------------------------------------------------
LOC_Os06g01670     ---------------------------------------------------------------
LOC_Os06g51260     ---------------------------------------------------------------
LOC_Os04g49450     ---------------------------------------------------------------
LOC_Os08g06110     ---------------------------------------------------------------
At5g17300          ---------------------------------------------------------------
At3g09600          ---------------------------------------------------------------
At5g02840          ---------------------------------------------------------------
At1g01060          ---------------------------------------------------------------
At2g46830          ---------------------------------------------------------------
At1g18330          ---------------------------------------------------------------
At5g52660          ---------------------------------------------------------------
At5g37260          ---------------------------------------------------------------
At1g01520          ---------------------------------------------------------------
At4g01280          ---------------------------------------------------------------
At3g10113          ---------------------------------------------------------------

cons


Cpa_00000217.44    ---------------------------------------------------------------
Cpa_00000769.37    ---------------------------------------------------------------
Popu_3444.2        -----AYQXX--XXXXXX---XXX----XA-MNCNST-P---SSI--------SRRAG-----
Popu_505.8         -------------------------S---EHTL--L-KYGYLKTHAGGSQQQQQRH------Q
Popu_2025.19       -----GVDDEMLLP-FGA---F-E----GM-EK--SSEE---SDP--------EKNAGVYDQL
```

```
Popu_2186.6        ------------SSKVSS---R----------EPDVM-S---LPL--------KQTQM-----
Popu_2302.6        ---------------------------------------------------------------
Ot06g02530         ----------------------------AQPGM--F-PMNAMM--------------------
Cre06.g275350.t    ALLAQLHAQASLVPY-HAAPGAGLAPSFAQ-QAMHTAT----AGA----QPFFATASAAASAI
kf100255_0190_v    -----LFAPA--MSG--------AMPAQLAQMM--A-TQYAAGGQFGG-----SAQ------Q
kf100237_0160_v    ---------------------------------------------------------------
Pp1s96_165V6.1     ---------------------------------------------------------------
Pp1s46_272V6.1     ---------------------------------------------------------------
Pp1s198_135V6.1    ---------------------------------------------------------------
Pp1s325_68V6.1     ---------------------------------------------------------------
Pp1s160_6V6.1      ---------------------------------------------------------------
Smo78045|PACid_    ---------------------------------------------------------------
MA_11267g0020      ---------------------------------------------------------------
MA_115536g0010     ---------------------------------------------------------------
MA_102199g0010     ---------------------------------------------------------------
LOC_Os02g46030     ---------------------------------------------------------------
LOC_Os01g06320     ---------------------------------------------------------------
LOC_Os02g45670     ---------------------------------------------------------------
LOC_Os06g01670     ---------------------------------------------------------------
LOC_Os06g51260     ---------------------------------------------------------------
LOC_Os04g49450     ---------------------------------------------------------------
LOC_Os08g06110     ---------------------------------------------------------------
At5g17300          ---------------------------------------------------------------
At3g09600          ---------------------------------------------------------------
At5g02840          ---------------------------------------------------------------
At1g01060          ---------------------------------------------------------------
At2g46830          ---------------------------------------------------------------
At1g18330          ---------------------------------------------------------------
At5g52660          ---------------------------------------------------------------
At5g37260          ---------------------------------------------------------------
At1g01520          ---------------------------------------------------------------
At4g01280          ---------------------------------------------------------------
At3g10113          ---------------------------------------------------------------

cons


Cpa_00000217.44    ---------------------------------------------------------------
Cpa_00000769.37    ---------------------------------------------------------------
Popu_3444.2        -------------------------------------------------------QGYRQEQA
Popu_505.8         QQGPLSVA----YDNMGNMG----------LGYSTNLHSDFPPEEARSHSLAQNLGGATSTQM
Popu_2025.19       -DQFLAGNEELGS-----RSPLRVRDERTRPAESAALATPLSGAPRTDVV-AEAMQALDDQMV
Popu_2186.6        ---------------------------------------------------------------
Popu_2302.6        ---------------------------------------------------------------
Ot06g02530         ---------------------------------------------------------------
Cre06.g275350.t    -QQGLLGAAH--------QQQQQQ--TLVATAAAAAAIAGSGSAAAASAA---ASA-------
kf100255_0190_v    WMPFGAGG----PWGNAAFEQ----LAMLAQMNAYNTANSNPFGPWGANPAAMWGGFGLGQNA
kf100237_0160_v    ---------------------------------------------------------------
Pp1s96_165V6.1     ---------------------------------------------------------------
Pp1s46_272V6.1     ---------------------------------------------------------------
Pp1s198_135V6.1    ---------------------------------------------------------------
Pp1s325_68V6.1     ---------------------------------------------------------------
Pp1s160_6V6.1      ---------------------------------------------------------------
Smo78045|PACid_    ---------------------------------------------------------------
MA_11267g0020      ---------------------------------------------------------------
MA_115536g0010     ---------------------------------------------------------------
MA_102199g0010     ---------------------------------------------------------------
LOC_Os02g46030     ---------------------------------------------------------------
LOC_Os01g06320     ---------------------------------------------------------------
LOC_Os02g45670     ---------------------------------------------------------------
LOC_Os06g01670     ---------------------------------------------------------------
LOC_Os06g51260     ---------------------------------------------------------------
LOC_Os04g49450     ---------------------------------------------------------------
LOC_Os08g06110     ---------------------------------------------------------------
At5g17300          ---------------------------------------------------------------
At3g09600          ---------------------------------------------------------------
At5g02840          ---------------------------------------------------------------
At1g01060          ---------------------------------------------------------------
At2g46830          ---------------------------------------------------------------
```

```
At1g18330          ---------------------------------------------------------------
At5g52660          ---------------------------------------------------------------
At5g37260          ---------------------------------------------------------------
At1g01520          ---------------------------------------------------------------
At4g01280          ---------------------------------------------------------------
At3g10113          ---------------------------------------------------------------

cons


Cpa_00000217.44    ---------------------------------------------------------------
Cpa_00000769.37    ---------------------------------------------------------------
Popu_3444.2        DEHQIGPG-AA----------------------------------------------------
Popu_505.8         --DGT--LWGARYHDML--AAPLQPVPPLHLLHHLQGPMDELQHERQLQQMQLMQHIQAM---
Popu_2025.19       PAVESKPN-FARVYAFF--ASIFYMPTPN--EAPPLA--------------------------
Popu_2186.6        ---------------------------------------------------------------
Popu_2302.6        ---------------------------------------------------------------
Ot06g02530         -------------------------------------------------------------SP
Cre06.g275350.t    ---AAAAAVASAAGSGSAASSMSQLLT----LAKSARR----------DA-------------
kfl00255_0190_v    --QGA--GG-----------------------------------TGA-EGQSAEEAAAESRAA
kfl00237_0160_v    ---------------------------------------------------------------
Pp1s96_165V6.1     ---------------------------------------------------------------
Pp1s46_272V6.1     ---------------------------------------------------------------
Pp1s198_135V6.1    ---------------------------------------------------------------
Pp1s325_68V6.1     ---------------------------------------------------------------
Pp1s160_6V6.1      ---------------------------------------------------------------
Smo78045|PACid_    ---------------------------------------------------------------
MA_11267g0020      ---------------------------------------------------------------
MA_115536g0010     ---------------------------------------------------------------
MA_102199g0010     ---------------------------------------------------------------
LOC_Os02g46030     ---------------------------------------------------------------
LOC_Os01g06320     ---------------------------------------------------------------
LOC_Os02g45670     ---------------------------------------------------------------
LOC_Os06g01670     ---------------------------------------------------------------
LOC_Os06g51260     ---------------------------------------------------------------
LOC_Os04g49450     ---------------------------------------------------------------
LOC_Os08g06110     ---------------------------------------------------------------
At5g17300          ---------------------------------------------------------------
At3g09600          ---------------------------------------------------------------
At5g02840          ---------------------------------------------------------------
At1g01060          ---------------------------------------------------------------
At2g46830          ---------------------------------------------------------------
At1g18330          ---------------------------------------------------------------
At5g52660          ---------------------------------------------------------------
At5g37260          ---------------------------------------------------------------
At1g01520          ---------------------------------------------------------------
At4g01280          ---------------------------------------------------------------
At3g10113          ---------------------------------------------------------------

cons


Cpa_00000217.44    ---------------------------------------------------------------
Cpa_00000769.37    -------------------------------AWRRSHSRTSSVGSACEDQRVPAGCPGSPGAG
Popu_3444.2        ---------------------------------------------------------------
Popu_505.8         QRP-----------VATSSGL-----------------------------------------H
Popu_2025.19       ---------------------------------------------------------------
Popu_2186.6        ---------------------------------------------------------------
Popu_2302.6        ---------------------------------------------------------------
Ot06g02530         FVA-----------MNTVSGA-----------------------------------------P
Cre06.g275350.t    RVPVEAFDVAAAVAAMAAAAGSGDLDATNAAELAQLQQVAAAAAEPRVS---AAAAADLLPPR
kfl00255_0190_v    AAA-----------VAAATMA-----------------------------------------A
kfl00237_0160_v    ---------------------------------------------------------------
Pp1s96_165V6.1     ---------------------------------------------------------------
Pp1s46_272V6.1     ---------------------------------------------------------------
Pp1s198_135V6.1    ---------------------------------------------------------------
Pp1s325_68V6.1     ---------------------------------------------------------------
Pp1s160_6V6.1      ---------------------------------------------------------------
```

```
Smo78045|PACid_    ---------------------------------------------------------------
MA_11267g0020      ---------------------------------------------------------------
MA_115536g0010     ---------------------------------------------------------------
MA_102199g0010     ---------------------------------------------------------------
LOC_Os02g46030     ---------------------------------------------------------------
LOC_Os01g06320     ---------------------------------------------------------------
LOC_Os02g45670     ---------------------------------------------------------------
LOC_Os06g01670     ---------------------------------------------------------------
LOC_Os06g51260     ---------------------------------------------------------------
LOC_Os04g49450     ---------------------------------------------------------------
LOC_Os08g06110     ---------------------------------------------------------------
At5g17300          ---------------------------------------------------------------
At3g09600          ---------------------------------------------------------------
At5g02840          ---------------------------------------------------------------
At1g01060          ---------------------------------------------------------------
At2g46830          ---------------------------------------------------------------
At1g18330          ---------------------------------------------------------------
At5g52660          ---------------------------------------------------------------
At5g37260          ---------------------------------------------------------------
At1g01520          ---------------------------------------------------------------
At4g01280          ---------------------------------------------------------------
At3g10113          ---------------------------------------------------------------

cons


Cpa_00000217.44    ---------------------------------------------------------------
Cpa_00000769.37    AELLRQPGS-----------------------------------------------------A
Popu_3444.2        ---------------------------------------------------------------
Popu_505.8         GG-------------------------------------------------------------
Popu_2025.19       -------------------------------------------------------------YA
Popu_2186.6        ---------------------------------------------------------------
Popu_2302.6        ---------------------------------------------------------------
Ot06g02530         TP-------------------------------------------------------------
Cre06.g275350.t    GGR-----GGSGSNTVPPLPLAAPESLLALAAAAQQESEQQQQLLQQQQQAALPSRKSVSSAA
kfl00255_0190_v    AS-------------------------------------------------------------
kfl00237_0160_v    ---------------------------------------------------------------
Pp1s96_165V6.1     ---------------------------------------------------------------
Pp1s46_272V6.1     ---------------------------------------------------------------
Pp1s198_135V6.1    ---------------------------------------------------------------
Pp1s325_68V6.1     ---------------------------------------------------------------
Pp1s160_6V6.1      ---------------------------------------------------------------
Smo78045|PACid_    ---------------------------------------------------------------
MA_11267g0020      ---------------------------------------------------------------
MA_115536g0010     ---------------------------------------------------------------
MA_102199g0010     ---------------------------------------------------------------
LOC_Os02g46030     ---------------------------------------------------------------
LOC_Os01g06320     ---------------------------------------------------------------
LOC_Os02g45670     ---------------------------------------------------------------
LOC_Os06g01670     ---------------------------------------------------------------
LOC_Os06g51260     ---------------------------------------------------------------
LOC_Os04g49450     ---------------------------------------------------------------
LOC_Os08g06110     ---------------------------------------------------------------
At5g17300          ---------------------------------------------------------------
At3g09600          ---------------------------------------------------------------
At5g02840          ---------------------------------------------------------------
At1g01060          ---------------------------------------------------------------
At2g46830          ---------------------------------------------------------------
At1g18330          ---------------------------------------------------------------
At5g52660          ---------------------------------------------------------------
At5g37260          ---------------------------------------------------------------
At1g01520          ---------------------------------------------------------------
At4g01280          ---------------------------------------------------------------
At3g10113          ---------------------------------------------------------------

cons
```

```
Cpa_00000217.44     ---------------------------------------------------------------
Cpa_00000769.37     SPCPSEGAATHV---------------------------------------------------
Popu_3444.2         ---------------------------------------------------------------
Popu_505.8          ---------------------------------------------------------------
Popu_2025.19        PKD----------STL-----------------------------------------------
Popu_2186.6         ---------------------------------------------------------------
Popu_2302.6         ------------DVDDEVLSRLESVAGLCE-ED------------------------------
Ot06g02530          ---------------------------------------------------------------
Cre06.g275350.t     ASALPRR--AGSFTAA-GASLVSSRGASPARAGAASPSDLASAAAAAVPSDRRRRAVLLVDSS
kfl00255_0190_v     ---------------------------------------------------------------
kfl00237_0160_v     ---------------------------------------------------------------
Pp1s96_165V6.1      ---------------------------------------------------------------
Pp1s46_272V6.1      ---------------------------------------------------------------
Pp1s198_135V6.1     ---------------------------------------------------------------
Pp1s325_68V6.1      ---------------------------------------------------------------
Pp1s160_6V6.1       ---------------------------------------------------------------
Smo78045|PACid_     ---------------------------------------------------------------
MA_11267g0020       ---------------------------------------------------------------
MA_115536g0010      ---------------------------------------------------------------
MA_102199g0010      ---------------------------------------------------------------
LOC_Os02g46030      ---------------------------------------------------------------
LOC_Os01g06320      ---------------------------------------------------------------
LOC_Os02g45670      ---------------------------------------------------------------
LOC_Os06g01670      ---------------------------------------------------------------
LOC_Os06g51260      ---------------------------------------------------------------
LOC_Os04g49450      ---------------------------------------------------------------
LOC_Os08g06110      ---------------------------------------------------------------
At5g17300           ---------------------------------------------------------------
At3g09600           ---------------------------------------------------------------
At5g02840           ---------------------------------------------------------------
At1g01060           ---------------------------------------------------------------
At2g46830           ---------------------------------------------------------------
At1g18330           ---------------------------------------------------------------
At5g52660           ---------------------------------------------------------------
At5g37260           ---------------------------------------------------------------
At1g01520           ---------------------------------------------------------------
At4g01280           ---------------------------------------------------------------
At3g10113           ---------------------------------------------------------------

cons

Cpa_00000217.44     ---------------------------------------------------------------
Cpa_00000769.37     ---------------------------------------------------------------
Popu_3444.2         ---------------------------------------------------------------
Popu_505.8          ---------------------------------------------------------------
Popu_2025.19        ---------------------------------------------SS----------------
Popu_2186.6         ---------------------------------------------------------------
Popu_2302.6         ---------------------AFGISASEDSSEYTLFAGLASSAPSWENSVKL-------G--
Ot06g02530          ---------------------------------------------------------------
Cre06.g275350.t     ANKALAAAAVVAVGDVEMATVATSMSALGAGSSAAAAAVALSDVPSGSTTVEALRNAVVLGLD
kfl00255_0190_v     ---------------------------------------------------------------
kfl00237_0160_v     ---------------------------------------------------------------
Pp1s96_165V6.1      ---------------------------------------------------------------
Pp1s46_272V6.1      ---------------------------------------------------------------
Pp1s198_135V6.1     ---------------------------------------------------------------
Pp1s325_68V6.1      ---------------------------------------------------------------
Pp1s160_6V6.1       ---------------------------------------------------------------
Smo78045|PACid_     ---------------------------------------------------------------
MA_11267g0020       ---------------------------------------------------------------
MA_115536g0010      ---------------------------------------------------------------
MA_102199g0010      ---------------------------------------------------------------
LOC_Os02g46030      ---------------------------------------------------------------
LOC_Os01g06320      ---------------------------------------------------------------
LOC_Os02g45670      ---------------------------------------------------------------
LOC_Os06g01670      ---------------------------------------------------------------
LOC_Os06g51260      ---------------------------------------------------------------
LOC_Os04g49450      ---------------------------------------------------------------
LOC_Os08g06110      ---------------------------------------------------------------
```

```
At5g17300        ---------------------------------------------------------------
At3g09600        ---------------------------------------------------------------
At5g02840        ---------------------------------------------------------------
At1g01060        ---------------------------------------------------------------
At2g46830        ---------------------------------------------------------------
At1g18330        ---------------------------------------------------------------
At5g52660        ---------------------------------------------------------------
At5g37260        ---------------------------------------------------------------
At1g01520        ---------------------------------------------------------------
At4g01280        ---------------------------------------------------------------
At3g10113        ---------------------------------------------------------------

cons


Cpa_00000217.44  ---------------------------------------------------------------
Cpa_00000769.37  -SRCSSFTMLPALEGDARGPSTSS-TPRLAGVP-PADAPALVVVPRATT--PRR---------
Popu_3444.2      ------------------------------------------------HVYSELSLPMTDH--
Popu_505.8       ---------------AS----LQFDM----------------------SMPFVPVA-YPQS--
Popu_2025.19     --------------------GCSPALCLLRDLN-LMDKEIVRMLAKNLLSNLDCYE---S---
Popu_2186.6      ---------------------------------------------------------------
Popu_2302.6      -----------------------E----------NV-----------SM--LSA---------
Ot06g02530       ---------------PP----MTNPQQFLNYANFF----------SNYWPQF-A---------
Cre06.g275350.t  PVRAPPLYAGDGSPPPPPAL--------------PLPVPVLPR----SV--PTR---------
kfl00255_0190_v  ---------------AW----WTLQNNAAAAAQMG----------RPSALPFPTPP-YPQQQQ
kfl00237_0160_v  ---------------------------------------------------------------
Pp1s96_165V6.1   ---------------------------------------------------------------
Pp1s46_272V6.1   ---------------------------------------------------------------
Pp1s198_135V6.1  ---------------------------------------------------------------
Pp1s325_68V6.1   ---------------------------------------------------------------
Pp1s160_6V6.1    ---------------------------------------------------------------
Smo78045|PACid_  ---------------------------------------------------------------
MA_11267g0020    ---------------------------------------------------------------
MA_115536g0010   ---------------------------------------------------------------
MA_102199g0010   ---------------------------------------------------------------
LOC_Os02g46030   ---------------------------------------------------------------
LOC_Os01g06320   ---------------------------------------------------------------
LOC_Os02g45670   ---------------------------------------------------------------
LOC_Os06g01670   ---------------------------------------------------------------
LOC_Os06g51260   ---------------------------------------------------------------
LOC_Os04g49450   ---------------------------------------------------------------
LOC_Os08g06110   ---------------------------------------------------------------
At5g17300        ---------------------------------------------------------------
At3g09600        ---------------------------------------------------------------
At5g02840        ---------------------------------------------------------------
At1g01060        ---------------------------------------------------------------
At2g46830        ---------------------------------------------------------------
At1g18330        ---------------------------------------------------------------
At5g52660        ---------------------------------------------------------------
At5g37260        ---------------------------------------------------------------
At1g01520        ---------------------------------------------------------------
At4g01280        ---------------------------------------------------------------
At3g10113        ---------------------------------------------------------------

cons


Cpa_00000217.44  ---------------------------------------------------------------
Cpa_00000769.37  ---------------------------------------------------------------
Popu_3444.2      ---------------------------------------------------------------
Popu_505.8       ---------------------------------------ASWMARSGPNQFELCSLRNSASSA
Popu_2025.19     ---------------------------------------------------------------
Popu_2186.6      ---------------------------------------------------------------
Popu_2302.6      ---------------------------------------------------------------
Ot06g02530       -----------------------N---------------------------------------
Cre06.g275350.t  ---------------------------------------------------------------
kfl00255_0190_v  QQQPASAPQGPSPADYGAPPLLRMPTAHSVQRGARSEETP-IRPNSPPASAKESELEQKGAAG
kfl00237_0160_v  ---------------------------------------------------------------
```

```
Pp1s96_165V6.1       ---------------------------------------------------------------
Pp1s46_272V6.1       ---------------------------------------------------------------
Pp1s198_135V6.1      ---------------------------------------------------------------
Pp1s325_68V6.1       ---------------------------------------------------------------
Pp1s160_6V6.1        ---------------------------------------------------------------
Smo78045|PACid_      ---------------------------------------------------------------
MA_11267g0020        ---------------------------------------------------------------
MA_115536g0010       ---------------------------------------------------------------
MA_102199g0010       ---------------------------------------------------------------
LOC_Os02g46030       ---------------------------------------------------------------
LOC_Os01g06320       ---------------------------------------------------------------
LOC_Os02g45670       ---------------------------------------------------------------
LOC_Os06g01670       ---------------------------------------------------------------
LOC_Os06g51260       ---------------------------------------------------------------
LOC_Os04g49450       ---------------------------------------------------------------
LOC_Os08g06110       ---------------------------------------------------------------
At5g17300            ---------------------------------------------------------------
At3g09600            ---------------------------------------------------------------
At5g02840            ---------------------------------------------------------------
At1g01060            ---------------------------------------------------------------
At2g46830            ---------------------------------------------------------------
At1g18330            ---------------------------------------------------------------
At5g52660            ---------------------------------------------------------------
At5g37260            ---------------------------------------------------------------
At1g01520            ---------------------------------------------------------------
At4g01280            ---------------------------------------------------------------
At3g10113            ---------------------------------------------------------------

cons


Cpa_00000217.44      ---------------------------------------------------------------
Cpa_00000769.37      ---------------------------------------------------------------
Popu_3444.2          ------------------------------ESS-AQRSSADPY--------------------
Popu_505.8           SMAVSGVDELGASERSGSGSDLENMSGG-------------------------------KESG
Popu_2025.19         ---------------------------------------------------------------
Popu_2186.6          ---------------------------------------------------------------
Popu_2302.6          ---------------------------------------------------------------
Ot06g02530           ---------------------------AANANAVNVMFQQQQQQQ------------------
Cre06.g275350.t      ---------------------------------------------------------------
kfl00255_0190_v      GTPRMAVPSASAPQPEMEAASSDPRSMGAPGGVPREAAIRRAERANKLRELKGRSGKAGDL--
kfl00237_0160_v      ---------------------------------------------------------------
Pp1s96_165V6.1       ---------------------------------------------------------------
Pp1s46_272V6.1       ---------------------------------------------------------------
Pp1s198_135V6.1      ---------------------------------------------------------------
Pp1s325_68V6.1       ---------------------------------------------------------------
Pp1s160_6V6.1        ---------------------------------------------------------------
Smo78045|PACid_      ---------------------------------------------------------------
MA_11267g0020        ---------------------------------------------------------------
MA_115536g0010       ---------------------------------------------------------------
MA_102199g0010       ---------------------------------------------------------------
LOC_Os02g46030       ---------------------------------------------------------------
LOC_Os01g06320       ---------------------------------------------------------------
LOC_Os02g45670       ---------------------------------------------------------------
LOC_Os06g01670       ---------------------------------------------------------------
LOC_Os06g51260       ---------------------------------------------------------------
LOC_Os04g49450       ---------------------------------------------------------------
LOC_Os08g06110       ---------------------------------------------------------------
At5g17300            ---------------------------------------------------------------
At3g09600            ---------------------------------------------------------------
At5g02840            ---------------------------------------------------------------
At1g01060            ---------------------------------------------------------------
At2g46830            ---------------------------------------------------------------
At1g18330            ---------------------------------------------------------------
At5g52660            ---------------------------------------------------------------
At5g37260            ---------------------------------------------------------------
At1g01520            ---------------------------------------------------------------
At4g01280            ---------------------------------------------------------------
At3g10113            ---------------------------------------------------------------
```

```
cons

Cpa_00000217.44     ---------------------------------------------------------------
Cpa_00000769.37     ---------------------------------------------------------------
Popu_3444.2         ---------------------------------------------------------------
Popu_505.8          TGSGSGSESPAIR--SGSRSVF---R----SESQE-AH---VQISA-----PAKRDSPAN-KT
Popu_2025.19        ---------------------------------------------------------------
Popu_2186.6         ---------------------------------------------------------------
Popu_2302.6         ---------------------------------------------------------------
Ot06g02530          ---------------------------------------------------------------
Cre06.g275350.t     ---------------------------------------------------------------
kfl00255_0190_v     LADSSVTP----GVFTDSCPWASSQTRRSLESSADKESAAVQKGGERGDHKGAAEEGRVQLKG
kfl00237_0160_v     ---------------------------------------------------------------
Pp1s96_165V6.1      -------------------------------------------------------SKDGD-KH
Pp1s46_272V6.1      ---------------------------------------------------------------
Pp1s198_135V6.1     ---------------------------------------------------------------
Pp1s325_68V6.1      -------------------------------------------------------CKDGE-KF
Pp1s160_6V6.1       ---------------------------------------------------------------
Smo78045|PACid_     ---------------------------------------------------------------
MA_11267g0020       -------------------------------------------------------NKDRC-QS
MA_115536g0010      -------------------------------------------------------EEMCY-SN
MA_102199g0010      ---------------------------------------------------------------
LOC_Os02g46030      ---------------------------------------------------------------
LOC_Os01g06320      ---------------------------------------------------------------
LOC_Os02g45670      ---------------------------------------------------------------
LOC_Os06g01670      ---------------------------------------------------------------
LOC_Os06g51260      ---------------------------------------------------------------
LOC_Os04g49450      ---------------------------------------------------------------
LOC_Os08g06110      -------------------------------------------------------QASCS-NS
At5g17300           ---------------------------------------------------------------
At3g09600           ---------------------------------------------------------------
At5g02840           ---------------------------------------------------------------
At1g01060           -------------------------------------------------------PDVIE-LN
At2g46830           -------------------------------------------------------NEDTN-KP
At1g18330           ---------------------------------------------------------------
At5g52660           ---------------------------------------------------------------
At5g37260           ---------------------------------------------------------------
At1g01520           ---------------------------------------------------------------
At4g01280           ---------------------------------------------------------------
At3g10113           ---------------------------------------------------------------

cons

Cpa_00000217.44     ---------------------------------------------------------------
Cpa_00000769.37     -------------------------------------------------PR------------
Popu_3444.2         -----------------THQHQQLRQKQVFKVPSLLSSGAFSYLGCGSVSSLAEPE-------
Popu_505.8          SGRSKEIRSP-----------------------------------------------SLDN
Popu_2025.19        ---------------------------------------------------------------
Popu_2186.6         -------------------------------------SGFGDFM-------------------
Popu_2302.6         --------------------------------------D-----LQESARG------------
Ot06g02530          --------------------------------------------------------Q------
Cre06.g275350.t     --------------------------------------V-----LSVSAAELPPPP-------
kfl00255_0190_v     GEQKEEQHALEAAILKKLKK----------------DERRRDKLRTQSASGSNEPAGRAESAA
kfl00237_0160_v     ---------------------------------------------------------------
Pp1s96_165V6.1      EKPETSSRLP-------------------------------------------------RTVS
Pp1s46_272V6.1      ---------------------------------------------------------------
Pp1s198_135V6.1     ---------------------------------------------------------------
Pp1s325_68V6.1      EKLESSSRLP-------------------------------------------------RTVP
Pp1s160_6V6.1       ---------------------------------------------------------------
Smo78045|PACid_     ---------------------------------------------------------------
MA_11267g0020       YGGDAAGHRS-------------------------------------------------KSGG
MA_115536g0010      NLSTKQGGLN-------------------------------------------------EEKI
MA_102199g0010      ---------------------------------------------------------------
LOC_Os02g46030      ---------------------------------------------------------------
LOC_Os01g06320      ---------------------------------------------------------------
```

```
LOC_Os02g45670     ---------------------------------------------------------------
LOC_Os06g01670     ---------------------------------------------------------------
LOC_Os06g51260     ---------------------------------------------------------------
LOC_Os04g49450     ---------------------------------------------------------------
LOC_Os08g06110     SAGDNNHRRF-------------------------------------------------RSSA
At5g17300          ---------------------------------------------------------------
At3g09600          ---------------------------------------------------------------
At5g02840          ---------------------------------------------------------------
At1g01060          NRKIKMRDNN-------------------------------------------------SNNN
At2g46830          QTSESNARRS-------------------------------------------------RISS
At1g18330          ---------------------------------------------------------------
At5g52660          ---------------------------------------------------------------
At5g37260          ---------------------------------------------------------------
At1g01520          ---------------------------------------------------------------
At4g01280          ---------------------------------------------------------------
At3g10113          ---------------------------------------------------------------

cons


Cpa_00000217.44    ---------------------------------------------------------------
Cpa_00000769.37    -----------------------------------------G---G-----------------
Popu_3444.2        -----------------------------------------SARAF-----------------
Popu_505.8         GS-------------GSDRPSSNSRGRHSG---DGSGHG--------SDLPSSTDENGGSGRI
Popu_2025.19       ---------------------------------------------------------------
Popu_2186.6        ------------------------------------------HGFQ-----------------
Popu_2302.6        ---------------------------------------------------------------
Ot06g02530         ------Q-------------------------------------------QQQ----------
Cre06.g275350.t    -----------------------------------------P---L-----------------
kf100255_0190_v    GHSNDRTAEPAPQRGGSPPEVSNSDGGASGSGKEGSGGGPSSNSSAVGDIPNE-DQQGAR---
kf100237_0160_v    ---------------------------------------------------------------
Pp1s96_165V6.1     ID-------------GEQRKEVTERGQIAF---QA----------------------------
Pp1s46_272V6.1     ---------------------------------------------------------------
Pp1s198_135V6.1    ---------------------------------------------------------------
Pp1s325_68V6.1     ID-------------AEQRKEVTERGQIAF---QA----------------------------
Pp1s160_6V6.1      ---------------------------------------------------------------
Smo78045|PACid_    ---------------------------------------------------------------
MA_11267g0020      HL-------------NETWKEVSQEGRIAF---QA----------------------------
MA_115536g0010     CV-------------QNSRNNSYSRGQIAF---RA----------------------------
MA_102199g0010     ---------------------------------------------------------------
LOC_Os02g46030     ---------------------------------------------------------------
LOC_Os01g06320     ---------------------------------------------------------------
LOC_Os02g45670     ---------------------------------------------------------------
LOC_Os06g01670     ---------------------------------------------------------------
LOC_Os06g51260     ---------------------------------------------------------------
LOC_Os04g49450     ------------------YR-------------------------------------------
LOC_Os08g06110     ST-------------SDSWKEVSEEGRLAF---DA----------------------------
At5g17300          ---------------------------------------------------------------
At3g09600          ---------------------------------------------------------------
At5g02840          ---------------------------------------------------------------
At1g01060          AT-------------TDSWKEVSEEGRIAF---QA----------------------------
At2g46830          NI-------------TDPWKSVSDEGRIAF---QA----------------------------
At1g18330          ---------------------------------------------------------------
At5g52660          ---------------------------------------------------------------
At5g37260          ---------------------------------------------------------------
At1g01520          ---------------------------------------------------------------
At4g01280          ---------------------------------------------------------------
At3g10113          ---------------------------------------------------------------

cons


Cpa_00000217.44    ---------------------------------------------------------------
Cpa_00000769.37    --------------------PR-----------------------------------------
Popu_3444.2        ---------------------RRRQSM-------------ESE--------------------
Popu_505.8         PQLVGPHKQEEGAINTASV---------------SLQ--------------------------
Popu_2025.19       ---------------------FRSV--------------------------------------
Popu_2186.6        ---------------------YKRPSM-------------MSF--------------------
```

```
Popu_2302.6        ---------------------------------------------------------------
Ot06g02530         ---------------------------------------------------------------
Cre06.g275350.t    --------------------PQAKAAA------------------------------------
kf100255_0190_v    --------------GRAGSEGASTEGEREQAGERGGQQKGTSPAEDERPGEGAPEPPQGDDRQ
kf100237_0160_v    ---------------------------------------------------------------
Pp1s96_165V6.1     ---------------------------------------------------------------
Pp1s46_272V6.1     ---------------------------------------------------------------
Pp1s198_135V6.1    ---------------------------------------------------------------
Pp1s325_68V6.1     ---------------------------------------------------------------
Pp1s160_6V6.1      ---------------------------------------------------------------
Smo78045|PACid_    ---------------------------------------------------------------
MA_11267g0020      ---------------------------------------------------------------
MA_115536g0010     ---------------------------------------------------------------
MA_102199g0010     ---------------------------------------------------------------
LOC_Os02g46030     ---------------------------------------------------------------
LOC_Os01g06320     ---------------------------------------------------------------
LOC_Os02g45670     ---------------------------------------------------------------
LOC_Os06g01670     ---------------------------------------------------------------
LOC_Os06g51260     ---------------------------------------------------------------
LOC_Os04g49450     ---------------------------------------------------------------
LOC_Os08g06110     ---------------------------------------------------------------
At5g17300          ---------------------------------------------------------------
At3g09600          ---------------------------------------------------------------
At5g02840          ---------------------------------------------------------------
At1g01060          ---------------------------------------------------------------
At2g46830          ---------------------------------------------------------------
At1g18330          ---------------------------------------------------------------
At5g52660          ---------------------------------------------------------------
At5g37260          ---------------------------------------------------------------
At1g01520          ---------------------------------------------------------------
At4g01280          ---------------------------------------------------------------
At3g10113          ---------------------------------------------------------------

cons

Cpa_00000217.44    ---------------------------------------------------------------
Cpa_00000769.37    ---------------------------------------------------------------
Popu_3444.2        ---------------------------------------------------------------
Popu_505.8         ----------K----------------------------------------------------
Popu_2025.19       ---------------------------------------------------------------
Popu_2186.6        ---------------------------------------------------------------
Popu_2302.6        ---------------------------------------------------------------
Ot06g02530         ---------------------------------------------------------------
Cre06.g275350.t    ---------------------------------------------------------------
kf100255_0190_v    AGSKPGRPFDELFRHQ--PQLQIAPAL--ME----------Q--GSDDRTPT----ARS----
kf100237_0160_v    ------------------V--------------------------------------------
Pp1s96_165V6.1     -----------LFKRDTLPRTFSPPPG--LALGTGTKEAAKQQPSVS---TM--S-RSLSANA
Pp1s46_272V6.1     -----------------EPVS-------------------LG---------------------
Pp1s198_135V6.1    -----------------EPGS-------------------TG---------------------
Pp1s325_68V6.1     -----------LFKLDTLPRTFSPPPG--LVLGTGSKEPTKKVASVH---NT--N-KSMSGSA
Pp1s160_6V6.1      -----------------EPVS-------------------LG---------------------
Smo78045|PACid_    -----------------QETW-------------------RK---------------------
MA_11267g0020      -----------LFNRDVLPQSFIPCQN--LRDQDKVPKVSRK--YYC---NDAKQ-KP-----
MA_115536g0010     -----------LFAQEVLPQSFSPGDG--MFSKI----IGRD--SFF---EDSKQ-KS-----
MA_102199g0010     -----------------GCSFKMA---------------------------------------
LOC_Os02g46030     --------------------RGQG---------------------------------------
LOC_Os01g06320     ---------------RRLLSTYDPN--RGLSGHL-----------------------------
LOC_Os02g45670     ---------------------------------------------------------------
LOC_Os06g01670     ---------------------------------------------------------------
LOC_Os06g51260     ---------------GAIYVRDSG-----------------------------S---------
LOC_Os04g49450     --------------------NGNR---------------------------------------
LOC_Os08g06110     -----------LFSRERLPQSFSPPQVEGSKEIS------KE--EED---EVTTVTVDL----
At5g17300          ---------------------------------------------------------------
At3g09600          ---------------------------------------------------------------
At5g02840          ---------------------------------------------------------------
At1g01060          -----------LFARERLPQSFSPP--QVAENVN------RK--QSD---TSMPLAPN-----
At2g46830          -----------LFSREVLPQSFTYR--EEHREEE------QQ--QQE---QRYPMALDL----
At1g18330          ---------------TEASENLEK---------------------------------------
```

```
At5g52660          ---------------------------------------------------------------
At5g37260          ---------------------------------------------------------------
At1g01520          ---------------------------------------------------------------
At4g01280          ---------------------------------------------------------------
At3g10113          ---------------TEASENLEK---------------------------------------

cons


Cpa_00000217.44    ---------------------------------------------------------------
Cpa_00000769.37    ---------------------------------------------------------------
Popu_3444.2        ---------------------------------------------------------------
Popu_505.8         ---------------------------------------------------------------
Popu_2025.19       ---------------------------------------------------------------
Popu_2186.6        ---------------------------------------------------------------
Popu_2302.6        ---------------------------------------------------------------
Ot06g02530         ---------------------------------------------------------------
Cre06.g275350.t    ---------------------------------------------------------------
kfl00255_0190_v    ---GQAG-----------------------------------------------G-KD---GA
kfl00237_0160_v    ---------------------------------------------------------------
Pp1s96_165V6.1     ITCDSLELRTSQRNHKDQDSAFNTWNHLRGTSSASKVDRCESGI------SIPSSRPTKSLED
Pp1s46_272V6.1     ---------------------------------------------------------P-----
Pp1s198_135V6.1    ---------------------------------------------------------L-----
Pp1s325_68V6.1     IVYDTSESWTPRRNRKDQDSAFNTWEHLRGTSSASKVDRCDSGI------SIPSSRPTKSLED
Pp1s160_6V6.1      ---------------------------------------------------------L-----
Smo78045|PACid_    ---------------------------------------------------------V-----
MA_11267g0020_     --SKTYRDE-----------------------------IQDDAQ------NMIVNQNQACNGD
MA_115536g0010     --SRSIEGE-----------------------------MCPHVE------GSGAGEEQAYTGK
MA_102199g0010     --S--------------------------------------------------ASQSNYADPL
LOC_Os02g46030     -----------------------------------------------------NSREAAASPR
LOC_Os01g06320     ---------------------------------------------------------------
LOC_Os02g45670     ---------------------------------------------------------------
LOC_Os06g01670     ---------------------------------------------------------------
LOC_Os06g51260     --S--------------------------------------------------FELKPSANSA
LOC_Os04g49450     -----------------------------------------------------NEDEMVPSPD
LOC_Os08g06110     ------NKN---------------------------------AAIID-----QELDTADEPRA
At5g17300          ---------------------------------------------------------------
At3g09600          ---------------------------------------------------------------
At5g02840          ---------------------------------------------------------------
At1g01060          --------------------------------------------FKS-----QDSCAADQEGV
At2g46830          ------N-------------------------------------FTAQLTPVDDQEEKRNTGF
At1g18330          --S--------------------------------------------------AEPISSSWKR
At5g52660          ---------------------------------------------------------------
At5g37260          --------------------------------------------------------------Q
At1g01520          ---------------------------------------------------------------
At4g01280          ---------------------------------------------------------------
At3g10113          --S--------------------------------------------------AEPISSSWKR

cons


Cpa_00000217.44    ---------------------------------------------------------------
Cpa_00000769.37    ---------------------------------------------------------------
Popu_3444.2        ---------------------HVDATAAAR---------------------------------
Popu_505.8         --------------------------EHPVTTNNSACGSASGS--------------------
Popu_2025.19       ---------------------------------------------------------------
Popu_2186.6        ---------------------------------------------------------------
Popu_2302.6        ---------------------------------------------------------------
Ot06g02530         ---------------------------------QQH-KQ------------------------
Cre06.g275350.t    ------------------------AARPDA---------------------------------
kfl00255_0190_v    VG-----E------------GLVRS-ASGKHHHHRHHHHAGNK--------------------
kfl00237_0160_v    ---------------------------------------------------------------
Pp1s96_165V6.1     LRSSERRRGSRKGRSHTGANNWLQLWTDTSSLDKDEDADEDDEEDESADSGADDGCK-KQQQK
Pp1s46_272V6.1     ---------------------------------------------------------------
Pp1s198_135V6.1    ---------------------------------------------------------------
Pp1s325_68V6.1     LQSSERRRGNRKVRSRSIANNWLQLWTDTSSRDRDEDADEEDEDDESAHSGADEGCK-KQQHE
Pp1s160_6V6.1      ---------------------------------------------------------------
Smo78045|PACid_    ---------------------------------------------------------------
```

```
MA_11267g0020       ALTEDCSC---------------PHKITLKAACSPVQVKKIHDSEATDNYRSLEKYGDSET--
MA_115536g0010      VHTQSSKA---------------P-------FVSISREKVLEDSEG------VVSH-------
MA_102199g0010      ---------------------------------------------------------------
LOC_Os02g46030      ---------------------------------------------------------------
LOC_Os01g06320      ---------------------------------------------------------------
LOC_Os02g45670      ---------------------------------------------------------------
LOC_Os06g01670      ---------------------------------------------------------------
LOC_Os06g51260      ---------------------------------------------------------------
LOC_Os04g49450      ---------------------------------------------------------------
LOC_Os08g06110      SF-------------------------------------------------------------
At5g17300           ----------------------------------------------TGHTEKTTEPE---T--
At3g09600           ---------------------------------------------------------------
At5g02840           ---------------------------------------------------------------
At1g01060           VM-------------------------------------------------------------
At2g46830           LG-------------------------------------------------------------
At1g18330           ---------------------------------------------------------------
At5g52660           ---------------------------------------------------------------
At5g37260           CSSSTSEN-------------------------A---------ETE------V----------
At1g01520           ---------------------------------------------------------------
At4g01280           ---------------------------------------------------------------
At3g10113           ---------------------------------------------------------------

cons


Cpa_00000217.44     ---------------------------------------------------------------
Cpa_00000769.37     ---------------------------------------------------------------
Popu_3444.2         ---------------------------------------------------------------
Popu_505.8          -------------------------------------------------------RSEGQE--
Popu_2025.19        ---------------------------------------------------------------
Popu_2186.6         ---------------------------------------------------------------
Popu_2302.6         ---------------------------------------------------------------
Ot06g02530          ---------------------------------------------------------------
Cre06.g275350.t     ---------------------------------------------------------------
kfl00255_0190_v     -------------------------------------------------------NASAAT--
kfl00237_0160_v     ---------------------------------------------------------------
Pp1s96_165V6.1      ASFVCKLDMSISNQ--EVELISDVEGTGASSLNISSTVRSQTLFLSVGTGGLKNAVS-N-V--
Pp1s46_272V6.1      ------------------------------------------------------------P--
Pp1s198_135V6.1     ------------------------------------------------------------P--
Pp1s325_68V6.1      ESFVCKLDVSISNQ--DTDLISNINDQDTSSFGLSSTVTSRTLSLSIGAGGLKNADS-S-V--
Pp1s160_6V6.1       ------------------------------------------------------------P--
Smo78045|PACid_     ------------------------------------------------------------R--
MA_11267g0020       ------------SLLLNKSVAVDFKP----------SNSSV--------------IS-S-F--
MA_115536g0010      ------------GL--LEASAVNFCK----------ANKFI--------------AS-S----
MA_102199g0010      -----------------KE------------------------------------EY-K-I--
LOC_Os02g46030      -------------------------------------------------------LT-K-CE-
LOC_Os01g06320      ---------------------------------------------------------------
LOC_Os02g45670      ---------------------------------------------------------------
LOC_Os06g01670      ---------------------------------------------------------------
LOC_Os06g51260      -------------------------------------------------------FV-R-VKP
LOC_Os04g49450      -------------------------------------------------------SR-K-CV-
LOC_Os08g06110      -------------------------------------------------------PN-E-L--
At5g17300           ------------ML----CQWEFKP----------SERSA--------------FS-E-L--
At3g09600           ---------------------------------------------------------------
At5g02840           ---------------------------------------------------------------
At1g01060           -------------------------------------------------------IG-V-G--
At2g46830           -------------------------------------------------------IG-L-D--
At1g18330           -------------------------------------------------------LS-S-LEK
At5g52660           ---------------------------------------------------------------
At5g37260           -------------------VVSEFKR----------SERSA--------------FS-Q-LK-
At1g01520           ---------------------------------------------------------------
At4g01280           ---------------------------------------------------------------
At3g10113           -------------------------------------------------------LS-S-LEK

cons


Cpa_00000217.44     ---------------------------------------------------------------
```

```
Cpa_00000769.37   --RRRLPAKRAR---------------------------------------------------
Popu_3444.2       EC---LR-SD-GVFVQQTDI---------AE--------------------------------
Popu_505.8        DS---S--LEGIGGSDSSLTQRRVNHAVTETKHIGHTKRERERERKRS------SSPSVLHKW
Popu_2025.19      ---------------------------------------------------------------
Popu_2186.6       ---------------V-------------SN--------------------------------
Popu_2302.6       ----YLPP-------------------------------------------------------
Ot06g02530        ---------------------RAG---------------------------------------
Cre06.g275350.t   KRTSSVALGDGDGAAAGTADPP-------PR--------------------------------
kf100255_0190_v   LG---S--STAGGSSGFVPYKGYQGTFVSQGSLDSQGRGREIKHDRPDPGAD--LPSPAKRQ-
kf100237_0160_v   -S---------Q---T-----------------------------------------------
Pp1s96_165V6.1    PT-----RTVKYSG-------------------------------------------------
Pp1s46_272V6.1    -S---------A---A-----------------------------------------------
Pp1s198_135V6.1   TS---------T---A-----------------------------------------------
Pp1s325_68V6.1    SA-----RTEKYSG-------------------------------------------------
Pp1s160_6V6.1     -C---------T---A-----------------------------------------------
Smo78045|PACid_   ---------------------------------------------------------------
MA_11267g0020     GN-----FRPDSSS-------------------DLNFFQTSS------VFHPHPVGGSAAALL
MA_115536g0010    -------FAKSKSG-------------------------------------------------
MA_102199g0010    RG-----KNRTYSG-------------------------------------------------
LOC_Os02g46030    SS-----ASVTLLQ-------------------------------------------------
LOC_Os01g06320    ---------------------------------------------------------------
LOC_Os02g45670    ---------------------------------------------------------------
LOC_Os06g01670    ---------------------------------------------------------------
LOC_Os06g51260    SN-----SGDEEVI-------------------------------------------------
LOC_Os04g49450    SP-----GS--NCR-------------------------------------------------
LOC_Os08g06110    SN-----LKLKSRR-------------------------------------------------
At5g17300         RR-----TNSESNS-------------------------------------------------
At3g09600         ---------------------------------------------------------------
At5g02840         ---------------------------------------------------------------
At1g01060         TC-----KSLKTRQ-------------------------------------------------
At2g46830         AS-----KLMSRGR-------------------------------------------------
At1g18330         QG-----SCNPVNA-------------------------------------------------
At5g52660         ---------------------------------------------------------------
At5g37260         SS-----VTEMNNM-------------------------------------------------
At1g01520         ---------------------------------------------------------------
At4g01280         ---------------------------------------------------------------
At3g10113         QG-----SCNPVNA-------------------------------------------------

cons


Cpa_00000217.44   -----------------------------------------------------------Q---
Cpa_00000769.37   ---------------LA----------------------------------------------
Popu_3444.2       ---------------LSDA---A-AS-------L-----------------------------
Popu_505.8        LSHQEPDQSKDTLASSP-KRSR-----------------------------------------
Popu_2025.19      ---------------------------------------------------------------
Popu_2186.6       ---------------D-ED--DA-MN-------LD----------------------------
Popu_2302.6       ---------------------------------------------------------------
Ot06g02530        ------------------GET------------------------------------------
Cre06.g275350.t   ---------------LP-P------R-------------------------------------
kf100255_0190_v   ---RVQVVAGEGVTRDG-GEERRSRSPPTEKRSSPA---------------GG-------GG-
kf100237_0160_v   --------------------------------IQRD---------------MRTESKEPA---
Pp1s96_165V6.1    ------------------------------VGFVPY---------------QR-------AST
Pp1s46_272V6.1    --------------------------------LQPQ---------------IPVLTMKPERVY
Pp1s198_135V6.1   --------------------------------LQPQ---------------SPVLTSEPESVY
Pp1s325_68V6.1    ------------------------------VGFVPY---------------QR-------VSI
Pp1s160_6V6.1     --------------------------------LQPQ---------------SPVLTTKPESVY
Smo78045|PACid_   ---------------------------------------------------------------
MA_11267g0020     AIS-------------------------FSYMFQIGQLADLQIVHIGSVTWET------LCTF
MA_115536g0010    ------------------------------VGFVPY---------------ER-------CS-
MA_102199g0010    ------------------------------AGFVPY---------------KG-------CQK
LOC_Os02g46030    ------------------------------RGFMPY---------------KR-------CAA
LOC_Os01g06320    ---------------------------------------------------------------
LOC_Os02g45670    ---------------------------------------------------------------
LOC_Os06g01670    ---------------------------------------------------------------
LOC_Os06g51260    ------------------------------RGFVPY---------------KR-------CKF
LOC_Os04g49450    ------------------------------RGFVPY---------------KR-------CVA
LOC_Os08g06110    ------------------------------TGFKPY---------------KR-------CSV
At5g17300         ------------------------------RGFGPY---------------KK-------RKM
```

```
At3g09600          --------------------------------------------------------------
At5g02840          --------------------------------------------------------------
At1g01060          -----------------------------TGFKPY--------------KR-------CSM
At2g46830          -----------------------------TGFKPY--------------KR-------CSM
At1g18330          -----------------------------SGFRPY--------------KR-------CLS
At5g52660          --------------------------------------------------------------
At5g37260          -----------------------------RGFMPY--------------KK-------RVK
At1g01520          --------------------------------------------------------------
At4g01280          --------------------------------------------------------------
At3g10113          -----------------------------SGFRPY--------------KR-------CLS

cons


Cpa_00000217.44    -------------------------------------R
Cpa_00000769.37    ----------------------------------PAEAAL
Popu_3444.2        ----------------------------------QQFAHQ
Popu_505.8         -------------------------------------H
Popu_2025.19       -------------------------------------S
Popu_2186.6        ----------------------------------QVVPQE
Popu_2302.6        -----------------------------------AALWN
Ot06g02530         -------------------------------------K
Cre06.g275350.t    ----------------------------------KRHHQL
kfl00255_0190_v    ------------G--GGGVT-GL-----------SMLMQF
kfl00237_0160_v    ----------------------------KS--------VGG
Pp1s96_165V6.1     RTE-PLV-D-----------------K-----------L
Pp1s46_272V6.1     -SAP-------S---LPSLTSLPSLPAGLVD-G-SVDTWWT
Pp1s198_135V6.1    -SLPRL-PSLPS---LPSLPSLPSLPAGLVGDGIGVDNWWT
Pp1s325_68V6.1     HVE-ATV-A-----------------K-----------L
Pp1s160_6V6.1      -SIT---------------SLPSLPAGLAGDE-SVDTWWT
Smo78045|PACid_    -----------------------------K------K----H
MA_11267g0020      DHM-GHEG-P-----------MMAQI-E-----------P
MA_115536g0010     -------------------------------------M
MA_102199g0010     DGQ-YP-G-----------ESDAEEC-N-----------L
LOC_Os02g46030     ESE-LLR-SEA-A--GGEEAVADGELTR------LC----L
LOC_Os01g06320     ----------------------------------------
LOC_Os02g45670     -------------------------------------E
LOC_Os06g01670     -------------------------------------M
LOC_Os06g51260     -------------------------------------Q
LOC_Os04g49450     DSE-ALL-KSQ-A--PQEE--ADGEMTR------LC----L
LOC_Os08g06110     EAK-EN-R----V--PASDEVGTKRI-R------LESEAST
At5g17300          VTE-EE-E-H------------EIHL-H------------L
At3g09600          -------------------------------------A
At5g02840          -------------------------------------S
At1g01060          EVK-ES-QVGNIN--NQSDEKVCKRL-R------LEGEAST
At2g46830          EAK-ES-RILNNNPIIHVEQKDPKRM-R------LETQAST
At1g18330          ERE-VT-SSLT-L--VASDEKKSQRA-R------I-----C
At5g52660          -------------------------------------T
At5g37260          VEE-NI-DNVK-L--SY----------P------L-----W
At1g01520          ----------------------------------------
At4g01280          ----------------------------------------
At3g10113          ERE-VT-SSLT-L--VASDEKKSQRA-R------I-----C

cons
```

PRRs

```
T-COFFEE, Version_11.00.d625267 (2016-01-11 15:25:41 - Revision d625267 - Build 507)
Cedric Notredame
CPU TIME:0 sec.
SCORE=453
*
 BAD AVG GOOD
*
Ot13g01890         :  40
Cre16.g676421.t    :  43
Cre02.g094150.t    :  40
kf100165_0030_v    :  41
kf100059_0230_v    :  45
Pp1s81_131V6.2     :  33
Pp1s412_35V6.1     :  48
Pp1s412_23V6.1     :  48
Pp1s81_144V6.2     :  48
Smo16699|PACid     :  99
Smo38684|PACid     :  99
Smo438647|PACid    :  50
MA_71728g0010      :  49
MA_10316458g001    :  57
MA_124244g0020     :  45
LOC_Os02g40510     :  50
LOC_Os11g05930     :  44
LOC_Os03g17570     :  48
LOC_Os07g49460     :  49
LOC_Os09g36220     :  47
At2g46790          :  51
At5g02810          :  45
At5g24470          :  46
At5g60100          :  48
At5g61380          :  56
cons               :  45

Ot13g01890         ---------------------------------------------------------------
Cre16.g676421.t    ---------------------------------------------------------------
Cre02.g094150.t    ---------------------------------------------------------------
kf100165_0030_v    MTGGLQRQNTGAGGPHQTSTGRSNRQ-------------------------------------
kf100059_0230_v    M--------------------------------------------------------------
Pp1s81_131V6.2     ---------------------------------------------------------------
Pp1s412_35V6.1     MTGDSC--------------------------------------KAEP---------------
Pp1s412_23V6.1     MTGDSC--------------------------------------KAEP---------------
Pp1s81_144V6.2     MTADLC--------------------------------------EFES---------------
Smo16699|PACid_    ---------------------------------------------------------------
Smo38684|PACid_    ---------------------------------------------------------------
Smo438647|PACid    ---------------------------------------------------------------
MA_71728g0010      MGK------------------------------------------------------------
MA_10316458g001    MSACSC--------------------------------------LGFP---------------
MA_124244g0020     MQKTSFSVILSAVLKLLETAFRSKDIFASDPRFTVFRSEGWDLTRFKVEKDVFCRLFPSKSWL
LOC_Os02g40510     MVGA-----------------------------------------------------------
LOC_Os11g05930     MSPD-----------------------------------------------------------
LOC_Os03g17570     ---------------------------------------------------------------
LOC_Os07g49460     ---------------------------------------------------------------
LOC_Os09g36220     MGGG-----------------------------------------------------------
At2g46790          MGEI-----------------------------------------------------------
At5g02810          MNANEE--------------------------------------GEGSRYPITDRKTGETKFD
At5g24470          MWQT-----------------------------------------------------------
At5g60100          MCFNNI--------------------------------------ETGDEVET-----------
At5g61380          MDLN-----------------------------------------------------------

cons


Ot13g01890         ---------------------------------------------------------------
Cre16.g676421.t    ---------------------------------------------------------------
Cre02.g094150.t    ---------------------------------------------------------------
kf100165_0030_v    ----------------------------TVESPREVGT--EQGSRHSQEEQGRDGSETPELGQ
kf100059_0230_v    ---------------------------------------------------------------
```

```
Pp1s81_131V6.2       ---------------------------------------------------------------
Pp1s412_35V6.1       ----------------------------------------ESRPLRPLLAGGTACAELNVEAP
Pp1s412_23V6.1       ----------------------------------------ESRPLRPLLAGGTACAELNVEAP
Pp1s81_144V6.2       ----------------------------------------ESDPLQPLSAVGRAWVEPIVGTP
Smo16699|PACid_      ---------------------------------------------------------------
Smo38684|PACid_      ---------------------------------------------------------------
Smo438647|PACid      ---------------------------------------------------------------
MA_71728g0010        ---------------------------------------------------------------
MA_10316458g001      ---------------------------------------------------------------
MA_124244g0020       FEEGIFKLDRTSQIFVREGYGYALVNSSGVQNIKVDEVTSKASGAMSTWRYKRDA---DPKHS
LOC_Os02g40510       ---------------------------------------------------------------
LOC_Os11g05930       ---------------------------------------------------------------
LOC_Os03g17570       ---------------------------------------------------------------
LOC_Os07g49460       ---------------------------------------------------------------
LOC_Os09g36220       --------------------------------------------VEERK--------------
At2g46790            ---------------------------------------------------------------
At5g02810            R--------------------------------------------------------------
At5g24470            --------------------------------------------WPRQPILLDIFSNPNTLST
At5g60100            ---------------------------------------------------------------
At5g61380            ---------------------------------------------------------------

cons


Ot13g01890           ---------------------------------------------------------------
Cre16.g676421.t      ---------------------------------------------------------------
Cre02.g094150.t      ---------------------------------------------------------------
kf100165_0030_v      VENGSNEKRAVEHRKGGGSGTGS-----GPSGMDTEMGNSSGQQGCA-GSS-GDGEGTQEPVM
kf100059_0230_v      ---------------------------------------------------------------
Pp1s81_131V6.2       ---------------------------------------------------------------
Pp1s412_35V6.1       VGAEWRIKGGYKAHKEVDRGREQ-----VGSKVAEDSE-NGARLEKGRNVAGRSAVSVLKTRE
Pp1s412_23V6.1       VGAEWRIKGGYKAHKEVDRGREQ-----VGSKVAEDSE-NGARLEKGRNVAGRSAVSVLKTRE
Pp1s81_144V6.2       VGAEWRIKGGFKAHKEVDRSREQ-----VGSKRVDDREKNSGRLENGCRFADRTGGAVLKARE
Smo16699|PACid_      ---------------------------------------------------------------
Smo38684|PACid_      ---------------------------------------------------------------
Smo438647|PACid      ---------------------------------------------------------------
MA_71728g0010        ---------------------------------------------------------------
MA_10316458g001      ---------------------------------------------------------------
MA_124244g0020       EEGLAKRVKFVVNFESGGENDGEQTLGKVADKVNLRYV-DGSNGQKG-SISTRNKEVVFPKNM
LOC_Os02g40510       ---------------------------------------------------------------
LOC_Os11g05930       ---------------------------------------------------------------
LOC_Os03g17570       ---------------------------------------------------------------
LOC_Os07g49460       ---------------------------------------------------------------
LOC_Os09g36220       ---------------------------------------------------------------
At2g46790            ---------------------------------------------------------------
At5g02810            ---------------------------------------------------------------
At5g24470            TVRSWSVRHPLSIITVKTFARFF-----LDIFFSPHYYRKNKVLFFA--------------LF
At5g60100            ---------------------------------------------------------------
At5g61380            ---------------------------------------------------------------

cons


Ot13g01890           -------MS---------DARTRSRGTVDIDGETLKRAD-R-GRRGRESGDRRSET-------
Cre16.g676421.t      --------------------------------------------------MQ-AHG-------
Cre02.g094150.t      ---------------------------------------------------------------
kf100165_0030_v      QERVVDEVARTVA-NTGLEDGKARLDVGRNVGEMQVRGG-E-TEQE----PERGGRDR--RVP
kf100059_0230_v      ---------------------------------------------------------------
Pp1s81_131V6.2       ---------------------------------------------------------------
Pp1s412_35V6.1       DLKDIAEQI------RRELD---HQFPGNDVLRTSESDD-D-GRRDDSAEDHYEEGDAVAAVA
Pp1s412_23V6.1       DLKDIAEQI------RRELD---HQFPGNDVLRTSESDD-D-GRRDGSAEDHYEEGDAVAAVA
Pp1s81_144V6.2       DPKDIAEQI------RRELD---HQFPVNDVLRTSESDE-D-GRREDSAEDHYEEGDAVAAVV
Smo16699|PACid_      ---------------------------------------------------------------
Smo38684|PACid_      ---------------------------------------------------------------
Smo438647|PACid      ---------------------------------------------------------------
MA_71728g0010        ---------------------------------------------------------------
MA_10316458g001      ---------------------------------------------------------------
MA_124244g0020       QVRPDAEGDNRLAEMNRHGRQ------DQKEA-RG--GIMGDGQEL----SEQDESRDD-EGM
```

```
LOC_Os02g40510     ---------------------------------------------------------------
LOC_Os11g05930     --------------------------------------------------------------A
LOC_Os03g17570     ------------M-GSACEAGTDEPSRDDVKGTGNGILE-N-GHSH----KPEEEEWR--NGM
LOC_Os07g49460     -----------MM-GTAHHNQTAG----SALGVG---VG-D-ANDA----VP---------GA
LOC_Os09g36220     ----------------------------------------------------VVDL-------
At2g46790          ---------------------------------------------------------------
At5g02810          ------------VESRTEKH--------------------------------SEEEKT--NGI
At5g24470          SFISPLTNI------LICFVTVSLSLELSSSSSIIDLGFS--KLSV----C-VVIMTSS-E-E
At5g60100          ---------------ERQVFG-------------------------------SSEEDE--FRV
At5g61380          ---------------------------------------------------------------

cons

Ot13g01890         --------------------------------------NAVAAAGEDGGERDADALRVLLATD
Cre16.g676421.t    --------------------------------------PQGHAGTQALGLPEPSKLRILVVDS
Cre02.g094150.t    --------------------------------------------------MEANGFHVVLVED
kfl00165_0030_v    AK---VYM-LESSDEDEA-----DVD----DEEGEGLDEVDDLDGSWQATLPSKHLRVLLVED
kfl00059_0230_v    ----------------------------------------DDKRFHDVTTFHRSSVHILLADR
Pp1s81_131V6.2     ---------------------------------------------------------------
Pp1s412_35V6.1     RD---KAR-SQGIARMKE-QQQH-G-----GD----LITKTQGGGGWESFLLQRSLNVLLVED
Pp1s412_23V6.1     RD---KAR-SQGIARTKE-QQQH-G-----GD----VAAKTQGGVGWESFLLKRSLRILLVEY
Pp1s81_144V6.2     FE---KQR-PREIAQTRE-QQQG-GNA---AA----AAAGTQGGGGWESFLLKRNLKVLLVED
Smo16699|PACid_    ---------------------------------------------------------------
Smo38684|PACid_    ---------------------------------------------------------VLLVED
Smo438647|PACid    ------------------------------------------MEGEGKIGFDRSRVRILLCDK
MA_71728g0010      ----------------------------------------GSISDAGSSVLDRSCVRILLCDK
MA_10316458g001    -----EIA----------------------------------------RTKQFLKLEIAIPIE
MA_124244g0020     ID---VGDGD-WHLPDANLLSQRLIH----QR----APQPQGSVIRWERFLPQRCLKILLVEN
LOC_Os02g40510     ----------GEG------------------D----RVGGGAAVGGGQQFVDRSKVRILLCDS
LOC_Os11g05930     DAAAAAAA-GGEGAA---------AAG---VG----TAGEGRGVIRWDQILPRRSLRVLLVEH
LOC_Os03g17570     GE---DLP-NGHSTPPEP-----QQT----DE----QKEHQVQIVRWERFLPVKTLRVLLVEN
LOC_Os07g49460     GG-------GGYSDPDGG-----PIS----GV----QRPPQ---VCWERFIQKKTIKVLLVDS
LOC_Os09g36220     ----------EDGDG---------EE---GED----AAAVAAGSSRETRMLPRMPVRVLLAEG
At2g46790          --VVL-----SSDDG---------MET---IK----NRVKSSEVVQWEKYLPKTVLRVLLVES
At5g02810          TM---DVR-NGSSGGLQI-----PLS----QQ----T-AA---TVCWERFLHVRTIRVLLVEN
At5g24470          VVE--VTV-VKAPEAGGG-----KLSRRKIRK----KDAGVDGLVKWERFLPKIALRVLLVEA
At5g60100          ED---TAR-NT--NN--V-----QIS----QQ----QQQPLAHVVKWERYLPVRSLKVLLVEN
At5g61380          ----------GEC------------------K-------------GGDGFIDRSRVRILLCDN

cons

Ot13g01890         DAHTRGMVHKMLRELGVEVVMATNGKEVLEVLRGRPKGVAAAAEDVSVDMILLDVLMPALDGE
Cre16.g676421.t    KAVSRQTTTQLLRECAYQVTAVKTAREGLQKLIE------A-EHGANFDLVLKEHEPPSANAC
Cre02.g094150.t    DNICLKVVEQLLRKLSYRVSTASDGAAALKVLAD------CKQRGDKVDLILTDILMPEVTGF
kfl00165_0030_v    DDCTRHVVAALLRNCGYEVTPAADGFQALKLLSD---------KMKQFDLVLTEVVMPGLSGI
kfl00059_0230_v    DLQTRKEVFELLRRCSYKVTAVESAKHVLEVLRA---------GGPAVDVILSEVEFPHGKGL
Pp1s81_131V6.2     ---------------------------------------------------------------
Pp1s412_35V6.1     DDATRHVVGALLRNCDCEVTAVANGSIAWGMLED---------ANSNFDLVLTDVVMPCLSGV
Pp1s412_23V6.1     DDATRHVVGALLRNCDYEVTAVANGSIAWGLLED---------ANSNFDLVLTDVVMPRLSGV
Pp1s81_144V6.2     DDATRHVVGALLRNCNYEVTPVANGSLAWGLLEE---------ANSNFDLVLTDVVMPYLSGV
Smo16699|PACid_    ----------------------------------------------EIDLVLTDVVMPGLSGL
Smo38684|PACid_    DDSTRHVVAALLRNCGYEVTPAASGLQAWEILET---------RSSSVDLVLTDLMMPRLSGI
Smo438647|PACid    DSATAQEVKELLCRCSYQVSVVKTARQVVEVLNI---------TDSKVDLVLSEVELPNGRGF
MA_71728g0010      DPTNSQQLLELLRKCMYQVTAVSTARGVVGVLNA---------EGQEIDLILADVDLPKSKGF
MA_10316458g001    TAPDLQEEKIFYQACARKEDIRSSNSEGGGQIGDT------LARAC-------ECVEPLQEAI
MA_124244g0020     DDSTRQVVSALLRNCSYEVVAVANGQQAWKLLED---------PSNHFDLVLTEVVMPCLSGI
LOC_Os02g40510     DPSSSREVLRLLCNCSYQVTCAKSPRQVINVLNC---------EAGEIDIILAEVDLPVSKCF
LOC_Os11g05930     DDSTRQVVTALLRKCGYRVAAVADGMKAWGVMRE---------RAYAFDLVLTEVTMPTLSGI
LOC_Os03g17570     DDSTRQVVSALLRKCCYEVIPAENGLHAWQCLED---------LQNHIDLVLTEVVMPRLSGI
LOC_Os07g49460     DDSTRQVVSALLRHCMYEVIPAENGQQAWTYLED---------MQNSIDLVLTEVVMPGVSGI
LOC_Os09g36220     DDSTRHIICALLRKCGYRVAAASDGVKAWDILKE---------KSFNIDLVLTEVELPLMSGF
At2g46790          DYSTRQIITALLRKCCYKVVAVSDGLAAWEVLKE---------KSHNIDLILTELDLPSISGF
At5g02810          DDCTRYIVTALLRNCSYEVVEASNGIQAWKVLED---------LNNHIDIVLTEVIMPYLSGI
At5g24470          DDSTRQIIAALLRKCSYRVAAVPDGLKAWEMLKG---------KPESVDLILTEVDLPSISGY
At5g60100          DDSTRHIVTALLKNCSYEVTAVPDVLEAWRILED---------EKSCIDLVLTEVDMPVHSGT
At5g61380          DSTSLGEVFTLLSECSYQVTAVKSARQVIDALNA---------EGPDIDIILAEIDLPMAKGM
```

```
cons

Ot13g01890          VELVEVCKSNEALRGVPIVIMSTVDERKAC-GTRYEHSGAAGFLTKPVNRTELKESLSHTRML
Cre16.g676421.t     -RLLKRMAKTEGLTRTPVVVTSSQDERETV-M-SCLSLGAIDYLIKPLRQNELRHIWTRVWWW
Cre02.g094150.t     -DLINEVVHGETFADIPVVVMSSQDSQESV-L-QAFQAGAADYLIKPIRKNELATLWQHVWRA
kfl00165_0030_v     -QLLSRIQQRDQHKRMPVIMMSSKDATDVV-F-KCFQRGAADFLVKPVRKNELKNLWQHVWRR
kfl00059_0230_v     -KLLKHIMREEKLKQIPVVMMSQRDEMALV--VKSLRLGAADYLVKPLRTNELLNLWTHMWRR
Pp1s81_131V6.2      -------MR---------VFVRGYG--------------------------------------
Pp1s412_35V6.1      -GLLSKMMKREACKRVPIVIMSSYDSLDIV-F-RCISKGACDYLVKPVRKNELRNLWQHVWRK
Pp1s412_23V6.1      -GLLSKMMKREACRRVPIVIMSSYDSLDIV-F-RCLSKGACDYLVKPVRKNELRNLWQHVWRK
Pp1s81_144V6.2      -GLLSKMMKREACKRVPIVIMSSYDSLGIV-F-RCLSKGACDYLVKPVRKNELKNLWQHVWRK
Smo16699|PACid_     -GLLSKIMHHKNHQKVPVVMMSSHDSTNVV-F-KCLTKGAADFLVKPVRKNELKNLWQH----
Smo38684|PACid_     -ELLGKIMSRDSPKRIPVVMMSCLDSMDVV-L-KCLSKGAVDFLVKPVRKNELKNL-------
Smo438647|PACid     -KMLKHIVKSENFKHIPIVMMSARDEMAVV--VKCLKLGAADYLVKPLRINELLNLWTHMWRR
MA_71728g0010       -KMLKYITRSTCLQRIPIVMMSARDEVTVV--MKCLKLGAADYLVKPLRINELLNLWMHMWRR
MA_10316458g001     -DLIGSIARK------PIEANEKVEDYQRAEL-EDKEGNGLQVNDETICDSNFTPLLELTLRR
MA_124244g0020      -GLLCKIMGRSTCKNIPVIMMSSHDSMGTV-F-KCLSKGAVDFLVKPVRKNELKNLWQHVWRR
LOC_Os02g40510      -KMLKYIARNKELRHIPIIMMSNRDEVSVV--VKCLRLGAAEYLVKPLRMNELLNLWTHVWRR
LOC_Os11g05930      -ELLSRIVASDECKNIPVIMMSSQDSIGTV-L-RCMQKGAVDFLVKPVRKNELRNLWQHVWRR
LOC_Os03g17570      -GLLSKITSHKICKDIPVIMMSSNDSMGTV-F-KCLSKGAVDFLVKPIRKNELKNLWQHVWRR
LOC_Os07g49460      -SLLSRIMNHNICKNIPVIMMSSNDAMGTV-F-KCLSKGAVDFLVKPIRKNELKNLWQHVWRR
LOC_Os09g36220      -LLLSTIMEHDACKNIPVIMMSSNDSVSMV-F-KCMLKGAADFLVKPIRKNELRNLWQHVWRK
At2g46790           -ALLALVMEHEACKNIPVIMMSSQDSIKMV-L-KCMLRGAADYLIKPMRKNELKNLWQHVWRR
At5g02810           -GLLCKILNHKSRRNIPVIMMSSHDSMGLV-F-KCLSKGAVDFLVKPIRKNELKILWQHVWRR
At5g24470           -ALLTLIMEHDICKNIPVIMMSTQDSVNTV-Y-KCMLKGAADYLVKPLRRNELRNLWQHVWRR
At5g60100           -GLLSKIMSHKTLKNIPVIMMSSHDSMVLV-F-KCLSNGAVDFLVKPIRKNELKNLWQHVWRR
At5g61380           -KMLRYITRDKDLRRIPVIMMSRQDEVPVV--VKCLKLGAADYLVKPLRTNELLNLWTHMWRR

cons

Ot13g01890          KSHESG----SAEN---D---G-SG-N------DP-NVG--------SAKS-----------S
Cre16.g676421.t     RKSQGS----GPHG---A---G-PP-P------IA-AAHGTRHYG-AFRGPS-SANR----QY
Cre02.g094150.t     NRAKGG----QTSS---G---A-AH-V-------G-AGGRGGT-S-SRDGG----------GV
kfl00165_0030_v     CKSSSG----SGSG---G---SGTN-A-------ATKVP-RKER--GR---V-RDGSGDR-S-
kfl00059_0230_v     RRMLGMAD--RLSGHPNM---N-GP-P-------L-DLAF---S--NT---S-DSNSGSV-D-
Pp1s81_131V6.2      --QSIG----SRSG---S---G-NQ-T-------G-EVARPQSRGVEA---A-DNPIGSN-D-
Pp1s412_35V6.1      CRSSSG----SRSG---S---G-SQ-T-------G-EVARPQSRGVEA---D-DNPSGSN-D-
Pp1s412_23V6.1      CHSSSG----SKSG---S---G-SQ-T-------G-EVARPQSRGVEA---D-DNPSGSN-D-
Pp1s81_144V6.2      CHSSSG----SRSG---S---G-SQ-T-------G-EVAKPRSRGVAA---A-DNPSGSN-D-
Smo16699|PACid_     ---------------------------------------------------------------
Smo38684|PACid_     ---------------------------------------------------------------
Smo438647|PACid     RRMLGLTE--KH-----I---L-KG-HLSSRNTIA-EIFV---S--DT---S-ESNTFST-D-
MA_71728g0010       RRMLGLAD--KN-----I---I-SK-NLNHD---V-DMLV---S--DP---S-DSNTNST-N-
MA_10316458g001     SHPISD----GDGV---HEPHVLRHSG-------A-SAFSRYNTKCNH---I-PQPSGDT-F-
MA_124244g0020      CQSSSD----SGGE---T---G-SQ-T-------Q-KVARLNST--AD---P-DNNTGSN-D-
LOC_Os02g40510      RRMLGLSE--KN-----F---F-ND-N-------F-ELAL---S--EP---S-DANTNST-T-
LOC_Os11g05930      HAMNSQTNA---S----E---N---------------------------------NAASNH--
LOC_Os03g17570      CHSSSG----SGSE---S---G-IR-T-------Q-KCTKPKVD--DE---Y-ENNSGSN-N-
LOC_Os07g49460      CHSSSG----SGSE---S---G-IQ-T-------Q-KCAKSKSG--DE---S-NNNNGSN-D-
LOC_Os09g36220      QLSSGVLDVQHTQQ---E---D-N------------L------T--ER---HEQKTG-VTKA-
At2g46790           LTLRDDPTA---H----A---Q-S-------------------L--PA----SQHNLE-----
At5g02810           CQSSSG----SGSE---S---G-THQT-------Q-KSVKSKSI--KK---S-DQDSGSS-D-
At5g24470           QTSLAPDSF---P----W---N-E-------------------S--VG---QQKAEG-AS---
At5g60100           CHSSSG----SGSE---S---G-IH-D-------K-KSVKPEST--QG---S-ENDASIS-D-
At5g61380           RRMLGLAE--KN-----M---L-SY-D-------F-DLVG---S--DQ---S-DPNTNST-N-

cons

Ot13g01890          -------------LTKLT--------NGEKAAGGS-GDGGSGGGGSGQG-QND--SGSD----
Cre16.g676421.t     DQATSSDS-KQTKCQEEEEPTSKEGSAPDNGN-GHGSKG-NG-----SN-GSK--EGNGT--S
Cre02.g094150.t     -------------AGTRCGPGDRGGSGGDAEGSGL-GGG-AG---A-VK-DS-----SG----
kfl00165_0030_v     GSG---------------------SD-----S---A----GGQVN-VGGNGSD--NGSGT--R
kfl00059_0230_v     M--LSNET------------------------VGER----RTRL-EGQV-N------------
Pp1s81_131V6.2      GNGSSDGS-----------------D-----N---G----SSRLN-AQG-GSD--NGSGN--Q
```

```
Pp1s412_35V6.1     GNGSSDGN-----------------D-----N---G----SSRLN-AQG-GSD--NGSGN--Q
Pp1s412_23V6.1     GNGSSDGS-----------------D-----N---G----SSRLN-AQG-GSD--NGSGN--Q
Pp1s81_144V6.2     GNGSSDGS-----------------D-----N---G----SSRVN-AQG-GSD--NGSGN--Q
Smo16699|PACid_    ---------------------------------------------------------------
Smo38684|PACid_    ---------------------------------------------------------------
Smo438647|PACid    I--FSEDS------------------------NDNK----VKKL-KPIS--------------
MA_71728g0010      L--FSDDT------------------------NDKK----VRNHAGPEI-S------------
MA_10316458g001    PLNK-HSF-----------------P-----P---H----FVMPF-ERM-NSA--NGSGIDVS
MA_124244g0020     ES-----------------------D-----D---A----SIGLN-NRD-GSD--NGSGT--Q
LOC_Os02g40510     L--LSDDT------------------------DDKP----KENI-NQET-S------------
LOC_Os11g05930     LS-----------------------A-----N---G----GNGSK-TGE-H--SDEESDA--Q
LOC_Os03g17570     DNEDDDDNDED-------------DD-----D---L----SVGHN-ARD-GSD--NGSGT--Q
LOC_Os07g49460     D--------DD-------------DD-----G---V----IMGLN-ARD-GSD--NGSGT--Q
LOC_Os09g36220     EH-----------------------V-----T---E----NVVHK-NME-C--SEQESD----
At2g46790          -------------------------D-----T---D----ETCED-SRY-H--SDQGSGA--Q
At5g02810          EN-----------------------E-----N---G----SIGLN-ASD-GSS--DGSGA--Q
At5g24470          -------------------------------A---N----NSNGK-RDD-HVVSGNGGD----
At5g60100          EHRN--------------------ES-----G---S----SGGLS-NQDGGSD--NGSGT--Q
At5g61380          L--FSDDT------------------------DDRS----LRST-NPQR-G------------

cons


Ot13g01890         ---------------------------------------------------------------
Cre16.g676421.t    --RVD-AKPHCDI-K-------------D----TG----------------------------
Cre02.g094150.t    --GSTGAATSVLHST-------------G----GT---TLPSRAATGRHASTSAGHGVTSADP
kf100165_0030_v    NESSP-VP--DE--K-------------V----FA----------------------------
kf100059_0230_v    ---ED-NERDRE-DSRPQMSPVLSLSLK--------PSS------------------------
Pp1s81_131V6.2     ----------VP-RN-------------N----AV----------------------------
Pp1s412_35V6.1     --ACM-QPVQVP-RN-------------N----AA----------------------------
Pp1s412_23V6.1     --ART-LPVLVP-MN-------------N----AV----------------------------
Pp1s81_144V6.2     --ACM-QPVQVL-RN-------------S----AI----------------------------
Smo16699|PACid_    ---------------------------------------------------------------
Smo38684|PACid_    ---------------------------------------------------------------
Smo438647|PACid    -----------E-EE--EQAPELELSLT--------PKS------------------------
MA_71728g0010      ---TL-ASHP-E-CE-SLDSSRLELSLK--------RSS------------------------
MA_10316458g001    --LHA-SKHPET-SH-------------NSGNQQD----------------------------
MA_124244g0020     --ISW-TKRVVE-VE-------------S----PQ----------------------------
LOC_Os02g40510     ---TS-NQHEYE-SN-PSDAEPKQK-----------GTP------------------------
LOC_Os11g05930     --SSG-SKREVE-IQ-------------S----AE----------------------------
LOC_Os03g17570     --SSW-TKRAVE-ID-------------S----PQ----------------------------
LOC_Os07g49460     AQSSW-TKRAVE-ID-------------S----PQ----------------------------
LOC_Os09g36220     AQSSC-TRSELE-AD-------------S----RQ----------------------------
At2g46790          AINYN-GHNKLM-EN-------------G----KS----------------------------
At5g02810          --SSW-TKKAVD-VD-------------D----SP----------------------------
At5g24470          AQSSC-TRPEME-GE-------------S----AD----------------------------
At5g60100          --SSW-TKRASD-TK-------------S----TS----------------------------
At5g61380          ---NL-SHQENE-WS-VATAPVHARDGGL----GADGTA------------------------

cons


Ot13g01890         -------------------------------NV------------------------------
Cre16.g676421.t    ----------HG---SNGNGH---GSNNGNGHGSNGNGSNGSSDNKNGGNGNSATKGDGNGNG
Cre02.g094150.t    SNNQTSHAHAHAHAHAHGNAH---AHAHLHMHGATDRAAQGSSANGPA---------------
kf100165_0030_v    ----------NGDG----GET---S----------------------------------PPL-
kf100059_0230_v    ----------EG------------------------------------VEIKA----KSGYEM
Pp1s81_131V6.2     ----------PEA------A-----D----------------------GDEEGQATSQDKGAE
Pp1s412_35V6.1     ----------PAA------A-----D----------------------GDEEGQATSQGMGAN
Pp1s412_23V6.1     ----------TAA------A-----D----------------------GDEEGQATSQETGAN
Pp1s81_144V6.2     ----------PEA------V-----D----------------------GDEEGQATSQDKGAD
Smo16699|PACid_    ---------------------------------------------------------------
Smo38684|PACid_    ---------------------------------------------------------------
Smo438647|PACid    ----------KD---------------------------------------ST----------
MA_71728g0010      ----------EG---------------------------------------PP----------
MA_10316458g001    ----------MDS--------------------------------------------------
MA_124244g0020     ----------RRSE----WDL---SQ----------------------PHSSTGGQVIQQKVE
LOC_Os02g40510     ----------EG------------------------------------LLVST----------
```

```
LOC_Os11g05930    ----------KLPE----VVA---DG----------------------GAGSSREHK------
LOC_Os03g17570    ----------QMSP----DQP---SD----------------------LPDSTCAQVIHPTSE
LOC_Os07g49460    ----------AMSP----DQL---AD----------------------PPDSTCAQVIHLKSD
LOC_Os09g36220    ----------TNNL----LEY---KQ----------------------PMGRHFSKP------
At2g46790         ----------VDE--------------------------------------------------
At5g02810         ----------RAVS------L---WD----------------------RVDSTCAQVVHSNPE
At5g24470         ----------VEV------SA---RD----------------------AVQMECAKS------
At5g60100         ----------PSNQ---FPDAPNKKG----------------------TYENGCAHVN---RL
At5g61380         ----------TS------------------------------------SLAVT----------

cons


Ot13g01890        ---------------------------------------------------------------
Cre16.g676421.t   SNGNGASTTLAVDRHYQALVN--TAEGKMPPNLLLGGSGSGH-DG-L---PGSAAKG-GTIGR
Cre02.g094150.t   N-------HGAAG------TGLQS--AGMAGSTAA----GAAAPA-GESLAKPPFAS------
kfl00165_0030_v   EEKA--LEVQREETPE--RRV--EN-----LLQTVGGKDGSR-RE-E---V-----S-R----
kfl00059_0230_v   L------------------------PLSRKPSYGSPAKFRTP-P-------------------
Pp1s81_131V6.2    LGYSWIRNKLDACVILLTTSA--LANEEMGHDLEMATRPSAC-NTTG----KDQQPE-V----
Pp1s412_35V6.1    L------------------------DEEMGHDLEMATRRSTC-DTAK----LDQQPD-V----
Pp1s412_23V6.1    L------------------------DEEMGHDLEMATRRSTC-NTAK----LDQQPD-V----
Pp1s81_144V6.2    L------------------------DGEMGHDLEMATRRSAC-VTTG----KDQQPE-D----
Smo16699|PACid_   ---------------------------------------------------------------
Smo38684|PACid_   ---------------------------------------------------------------
Smo438647|PACid   ---------------------------------------------------------------
MA_71728g0010     ---------------------------------------------------------------
MA_10316458g001   ---------------------------------------------------------------
MA_124244g0020    TAQNGWMQMAVT-SGECQDKK--TLDFAMGQDLEIAVPGQTD-SE-F---EHQEEEK-V----
LOC_Os02g40510    ---------------------------------------------------------------
LOC_Os11g05930    -IQNGFIDGMNT-K----------------S-HALKGNDDAP-SG-N---ACGDSELQV----
LOC_Os03g17570    ICSNRWLPTANKRSGKKHKEN--NDD-SMGKYLEIGAPRNSS-ME-Y---QSSPREM-S----
LOC_Os07g49460    ICSNRWLPCTSNKNSKKQKET--NDD-FKGKDLEIGSPRNLN-TA-Y---QSSPNER-S----
LOC_Os09g36220    ------------------------------D-HKNTEKNGGT-KI-H---ASN---DGN----
At2g46790         ---------------------------------------------------------------
At5g02810         FPSNQLVAPPAEKETQEHDDK--FEDVTMGRDLEISIRRNCD-LA-L---EPKDEPL-S----
At5g24470         ------------------------------Q--------------------------------
At5g60100         KEAEDQKEQIGT-------------------------GSQTG-MS-M---SKKAEEP------
At5g61380         --------------------------AI--EPPLD--HLAGS-H-------------------

cons


Ot13g01890        ---------------------------------------------------------------
Cre16.g676421.t   SSAFAQ---------------------------------------------------YTGPSS
Cre02.g094150.t   ----LAV---HFD---------LHSVLAGAGAA-----A--ANGG---ANAAAHTAGATGRES
kfl00165_0030_v   -----GQPM--RER-G--GDAGVGS----TRR--P--E---EEEGS-P---------S----R
kfl00059_0230_v   --------A------------------------------------------------------
Pp1s81_131V6.2    -----ARQLDE----------DAAC----VFQD-----AGQSPDGI-NGESPSSSLRNDAAEE
Pp1s412_35V6.1    -----GRQQDE----------DDAC----VMQD-----AGPSPGED-NGESPSTSGNDGTAEE
Pp1s412_23V6.1    -----GRQQDD----------DDAC----VMQD-----VGPSPGED-NVESPSTSGKDGTTEE
Pp1s81_144V6.2    -----AQKQDE----------DAVC----ILQD-----AGPSPDGA-NAESPSSSGRNDAAEE
Smo16699|PACid_   ---------------------------------------------------------------
Smo38684|PACid_   ---------------------------------------------------------------
Smo438647|PACid   --------R------------------------------------------------------
MA_71728g0010     --------K------------------------------------------------------
MA_10316458g001   -----A-VGSS----------GQDM----AA-S-----LASSHMEIPTPIPILYNGVPVQYGA
MA_124244g0020    -----LYAQTFGREENG-SL-ASSA----EGV--E--I-REEPVET-G---------D----C
LOC_Os02g40510    --------E------------------------------------------------------
LOC_Os11g05930    -----LSTE-KNVR-SKFLN-GITS----AKVAGQ---IMDNALRF-A---------DSSSLR
LOC_Os03g17570    -----VNPTEKQHE-TLMPQ-SKTT----RET---------DSRNT-Q---------N----E
LOC_Os07g49460    -----IKPTDRRNE-Y--PL-QNNS----KEA---------AMENL-E--------------E
LOC_Os09g36220    -----LIPR-REED-ASLRR-MTCS----NDINCEKAS---R---------------------
At2g46790         ---------------------------------------------------------RDEFKE
At5g02810         -----KTTGIMRQD-NSFEK-SSSK----WKM--K--V-GKGPLDL-S---------S----E
At5g24470         ---------------------FNET----RLLANELQSKQAE---------------------
At5g60100         --------GDLEKN-AKYSV-QALE----R---------------------------------
At5g61380         -------HE------------------------------------------------------
```

```
cons


Ot13g01890          ---------------------------------------------------------------
Cre16.g676421.t     -SSR---KRAATSEAVPP---G--ARD-RPDT-MLT-DRDRERDR-DRDLIGVSGGGGGSGSG
Cre02.g094150.t     -GQA---AGAATGGIAAA---G--TVI-GWS--------HADMD-----VD---------GG-
kfl00165_0030_v     -SSE---KAA------DL---IGNFVE-EKA--VEIT-SPRGGEG-CQPAGA-----------
kfl00059_0230_v     -----------------------PPP-------------------------------------
Pp1s81_131V6.2      -SSP---KAI------DL---INVVACQ-PQT-QDA-EQPQESEN-DFDELD-----------
Pp1s412_35V6.1      -SSP---KAV------DL---SHRRRVQ-SKL-KGV-RTMT--EN-----WI-----------
Pp1s412_23V6.1      -SSL---KAV------DL---INGIACQ-PQT-QGA-EQAEGSEN-DDGELD-----------
Pp1s81_144V6.2      -SSP---KII------DL---INVIACQ-PQT-QDA-E-PQESEN-DDEELD-----------
Smo16699|PACid_     ---------------------------------------------------------------
Smo38684|PACid_     ---------------------------------------------------------------
Smo438647|PACid     -----------------------TTEI-G----------------------------------
MA_71728g0010       -----------------------APEL-G----------------------------------
MA_10316458g001     -AFP---AMF------HPQTCALSRSSG-TEH-MTE-RPERQEVY-DFACHH-----------
MA_124244g0020      PSSEVATKA--A----DL---IGAIAN-KPNS--GNSKLEDNRSR-E--ALD-----------
LOC_Os02g40510      -----------------------GGDQ-A----------------------------------
LOC_Os11g05930      -SSD---PGK------DL---LVVAQT-TADRKCKSSA-L-ENNAVM--ENN-----------
LOC_Os03g17570      -PTT---QTV------DL---ISSIAR-STDD-KQVVRINNAPDC-S--SKV-----------
LOC_Os07g49460      -SSV---RAA------DL---IGSMAK-NMDA-QQAARAANAPNC-S--SKV-----------
LOC_Os09g36220      --------DM------EL---VHIIDN-QQ-----K------NNTHM--EMD-----------
At2g46790           -TFD---VTM------DL---IGGIDK-RPDSI--------Y--KDK--SR------------
At5g02810           SPSSK--QMH-E----DG---GSSFKAMSSHL--QDNREPEAPNT-H--LKT-----------
At5g24470           --------AI------DF---MGASFR-RT-----G------R--------------------
At5g60100           ----------------------------NNDD--TLNRSSGNSQV-E--SKA-----------
At5g61380           -----------------------PMKR-N----------------------------------

cons


Ot13g01890          -----------RLYNEGNEKSRRREAEAMDRWASREGGSGDCGSGGSGQGTGSGSGSHEGSGS
Cre16.g676421.t     RH-G-DRADRDALMRDERAREREDNTGGTGGVSGMPAAAEGTATGGGGTGGGSAGGVRGGSGD
Cre02.g094150.t     ---------E-AGAQDEDDEDEDDGVEAPAGTQNRKRAADDSGCDGAAANNNGNTAAKAGAAA
kfl00165_0030_v     ER-G-GVFSEDGEG--R-----------GPMPGL-----------------------------
kfl00059_0230_v     ---------------------------------------------------------------
Pp1s81_131V6.2      PQ-G-S-SPKV--N--S---G------SDSGPML-----------------------------
Pp1s412_35V6.1      SG-G-GAVRKT--T--P---G------Q-----------------------------------
Pp1s412_23V6.1      QR-G-RSSPKD--H--S---G------SDFGSML-----------------------------
Pp1s81_144V6.2      PR-G-RSSPKN--N--S---A------SDSGTSL-----------------------------
Smo16699|PACid_     ---------------------------------------------------------------
Smo38684|PACid_     ---------------------------------------------------------------
Smo438647|PACid     ---------------------------------------------------------------
MA_71728g0010       ---------------------------------------------------------------
MA_10316458g001     -------------K--Q---L------IMPPQTL-----------------------------
MA_124244g0020      ND---------KIG--T---D------FKHLPFL-----------------------------
LOC_Os02g40510      ---------------------------------------------------------------
LOC_Os11g05930      LSENSK--GTATGH--A---E------SCPSHFV-----------------------------
LOC_Os03g17570      PD-G-----NDKNR--D---S------LIDMTSE-----------------------------
LOC_Os07g49460      PE-G-----KDKNR--D-----------NIMPSL-----------------------------
LOC_Os09g36220      VARANS--RGNDDK--C---F------SIPAHQL-----------------------------
At2g46790           --------------------D------ECVGPEL-----------------------------
At5g02810           LD-T-----NEASV--K---I------SEELMHV-----------------------------
At5g24470           -----R--NREESV--A---Q------Y--ESRI--EL-------------------------
At5g60100           ------------PS--S---N------REDLQSL-----------------------------
At5g61380           ---------------------------------------------------------------

cons


Ot13g01890          GQRDSNENEKFRGLSVQLIKA-----HGGAT-TMLELSLPEHSSEHVVVRRSNSRSAFKGFQT
Cre16.g676421.t     GGRRSKGSTQRD-------------------------------RAMASLRA-SGGSGVHTPSD
Cre02.g094150.t     IAAGGPGSSGRAKAT---------------------------DGARAEIRH-NGGPMAARMAA
kfl00165_0030_v     -----E---------LSLKRG-----RPAGDSSEE-A-QPPSKQQMRTLRQ-SGGSAFSRYSM
kfl00059_0230_v     ----------------------------NNS-LFG-H-----TPKRMRGGE-IESRGFSSSAR
Pp1s81_131V6.2      -----E---------LSLKRP-----RSAVD-NDG-E-----LEERQPLRY-SGGSAFSRYDS
Pp1s412_35V6.1      ----------------------------------------------TPLVK-CDIELGRWYGS
```

```
Pp1s412_23V6.1     -----E---------LSLKRP-----RSAVD-NDG-D-----TEERQPLRH-SGGSAFSRYGS
Pp1s81_144V6.2     -----E---------LSLKRP-----RSAVG-NGG-E-----LEERQPLRH-SGGSAFSRYGS
Smo16699|PACid_    ---------------------------------------------------------------
Smo38684|PACid_    ---------------------------------------------------------------
Smo438647|PACid    ---------------------------E-PE-RRP-S-----PPRKSELKL-GQSSAFLSYTR
MA_71728g0010      ---------------------------SFAG-KFA-S-----CPKKSELKF-GGSSAFLTYVN
MA_10316458g001    -----Q---------PSYQNSNHHYYHIQHP-EPH-E----DKQSHQHLEK-GEHSMNTLTMM
MA_124244g0020     -----E---------LTLKRP-----RQNGK-EDG-E-----PEDRHVLRQ-SGVSAFSRYNT
LOC_Os02g40510     ---------------------------SSPG-VMF-S-----RPIKTNLRV-AESSAFLAYVK
LOC_Os11g05930     -----E---------INLEKQ-----HHLNGYTNH-K-----LNEKDIFNH-SNSSAFSRYGN
LOC_Os03g17570     -----E---------LGLKRL-----KTTGS-ATE-I-H---DE-RNILKR-SDLSAFTRYHT
LOC_Os07g49460     -----E---------LSLKRS-----RSTGDGANA-I-Q---EEQRNVLRR-SDLSAFTRYHT
LOC_Os09g36220     -----E---------LSLRRS-----DYSRL-ESQ-E-----KNERRTLNH-STSSPFSLYNC
At2g46790          -----G---------LSLKRS-----CSVSFE-NQ-D-----ESKHQKLSL-SDASAFSRFEE
At5g02810          -----E---------HSSKRH-----RGTKDDGTL-V-----RDDRNVLRR-SEGSAFSRYNP
At5g24470          -----D---------LSLRRP-----NAS---ENQ-S-----SGDRPSLHP-SSASAFTRYVH
At5g60100          -----E---------QTLKKT-----REDR---DY-K-----VGDRSVLRH-SNLSAFSKYNN
At5g61380          ---------------------------SNPA-QFS-S-----APKKSRLKI-GESSAFFTYVK

cons

Ot13g01890         YLK-SEK-------KESSV--------------------------------------------
Cre16.g676421.t    GAG-AAGLLSGGGGNGGNGNN------------------------------------------
Cre02.g094150.t    AEG-SQG----SRAASGSAATGPGGAREGTATPSGDT--------------------------
kfl00165_0030_v    G-GAMTNS-S---------------------RQ------------------------------
kfl00059_0230_v    EAP-EQTSG----------------------ESQPPFHQPPQLHKFVPSSGWHPNGAQHAPQT
Pp1s81_131V6.2     GGT-IIQQ-H---------------------YQGGSS--------------------------
Pp1s412_35V6.1     GGT-IIQQ-C---------------------HQPGSS--------------------------
Pp1s412_23V6.1     GGT-IIQQ-C---------------------HQPGNS--------------------------
Pp1s81_144V6.2     GGT-IIQQ-Y---------------------HQTGGS--------------------------
Smo16699|PACid_    ---------------------------------------------------------------
Smo38684|PACid_    ---------------------------------------------------------------
Smo438647|PACid    VVS-RTTNA----------------------EKPDST--------------------------
MA_71728g0010      ASV-QANKT----------------------PNLSSI-----------------GE-------
MA_10316458g001    T--------------------------------VPNC--------------------------
MA_124244g0020     SGGQ---F-S---------------------NPSGDT--------------------------
LOC_Os02g40510     SST-PTTSS----------------------FDSELQ------------KGGNRLDS-SD-HR
LOC_Os11g05930     KRIE-SSA-Q-----------------------------------------------------
LOC_Os03g17570     T-VASNQG-G-----------------------------------------------------
LOC_Os07g49460     P-VASNQG-G-----------------------------------------------------
LOC_Os09g36220     RTAS-STI-N-----------------------------------------------------
At2g46790          SKSA-EKA-V-----------------------------------------------------
At5g02810          A-SNANKI-------------------------------------------------------
At5g24470          RPLQ-TQC-S-----------------------------------------------------
At5g60100          GATSAK-K-A-----------------------------------------------------
At5g61380          STV-LRTNG----------------------QDPPLV------------DGNGSLHL-HRGLA

cons

Ot13g01890         ---------------------------------------------------------------
Cre16.g676421.t    ---------------------------------------------------------------
Cre02.g094150.t    -----FAESPSTFTSI-----------------------------------------------
kfl00165_0030_v    ---------------------------------------------------------------
kfl00059_0230_v    G-SSST---------PHRSATAEGVPAWAAPRVSQQPPAQEHFSRGALVHSHGQEMQQRGHNV
Pp1s81_131V6.2     --------------L------------------------------------------------
Pp1s412_35V6.1     --------------L------------------------------------------------
Pp1s412_23V6.1     --------------L------------------------------------------------
Pp1s81_144V6.2     --------------L------------------------------------------------
Smo16699|PACid_    ---------------------------------------------------------------
Smo38684|PACid_    ---------------------------------------------------------------
Smo438647|PACid    --------------------TQPPTCMEVPVGTSQQPLDLEEPGQVGARGNQAAELTQTQEPD
MA_71728g0010      --NKAS---------QQETAIPQKHCVMGPPSADSGSIGLSHSSEAV-KSSNRTEFIPCEEDC
MA_10316458g001    --------------G------------------------------------------------
MA_124244g0020     --------------L------------------------------------------------
LOC_Os02g40510     GNFSST---------TDRSDTGTDVNIRDK---EAFEMPV-Q-YPVVCF-SSSNLHLER----
LOC_Os11g05930     ---------------------------------------------------------------
```

```
LOC_Os03g17570     ---------------------------------------------------------------
LOC_Os07g49460     ---------------------------------------------------------------
LOC_Os09g36220     ---------------------------------------------------------------
At2g46790          ---------------------------------------------------------------
At5g02810          ---------------------------------------------------------------
At5g24470          ---------------------------------------------------------------
At5g60100          ---------------------------------------------------------------
At5g61380          EKFQVV---------ASEGINNTKQARRATPKSTVLRTNG-Q-DPPLVN-GNGSHHLHRGA-A

cons


Ot13g01890         ---------------------------------------------------------------
Cre16.g676421.t    ---------------------------------------------------------------
Cre02.g094150.t    -------------------------------IN-------------------T---T--GSG-
kfl00165_0030_v    -----------------------------------------------------------SQN-
kfl00059_0230_v    ERRDSLPTPPEYGRTRHVGASSNAGNSAPPPEYHRSAFQGDARRGTEGGEGSWQNSHRPPYFP
Pp1s81_131V6.2     --------------------------------P-------------------LNGYPMCGAY-
Pp1s412_35V6.1     --------------------------------P-------------------VGGYPMSGGY-
Pp1s412_23V6.1     --------------------------------P-------------------VGGYPMSGGY-
Pp1s81_144V6.2     --------------------------------P-------------------LSGYPVSGGY-
Smo16699|PACid_    ---------------------------------------------------------------
Smo38684|PACid_    ---------------------------------------------------------------
Smo438647|PACid    DDPEQ-SHHHHHQHSI--------------------PWSSSSTTGAAEQY-------------
MA_71728g0010      RDRLE-ARPCNVSVSP--------------------EIPIGQVATAGEQF-------------
MA_10316458g001    --------------------------------L-------------------SSMVTFDGNH-
MA_124244g0020     --------------------------------P-------------------PNSLP--KGY-
LOC_Os02g40510     --------------------------------------SNEGQNDASGTPP------VYHFP-
LOC_Os11g05930     ---------------------------------------------------------------
LOC_Os03g17570     -----------------------------------------------------------AGF-
LOC_Os07g49460     -----------------------------------------------------------TGF-
LOC_Os09g36220     ---------------------------------------------------------------
At2g46790          ---------------------------------------------------------------
At5g02810          --------------------------------------------------------S--GGN-
At5g24470          ---------------------------------------------------------------
At5g60100          --------------------------------------------------------P--EEN-
At5g61380          EKFQVVAS-------E--------------------GINNTKQAHRSRGTE------QYHSQG

cons


Ot13g01890         ---------------------------------------------------------QMMS--
Cre16.g676421.t    -------------------G----------------SSYN-------NKQPTGSSE-RPG---
Cre02.g094150.t    ------SE----------AD----------------EQPV-------PLKHQEQQQ-QQQQQR
kfl00165_0030_v    ------QPPQSQHVGGYSSGPIGPPHNGGSAMGSFMPPEF-------QMPSAF----------
kfl00059_0230_v    EPQFAGMV----------PS----------------NMGAMAQLPPHFYMPPHVQGGGRESSK
Pp1s81_131V6.2     ------GV----------YG----------------MPGG-------GSGGSLRLG-MGMDRI
Pp1s412_35V6.1     ------GV----------YG----------------MSGG-------TSGGSLRLG-MGMERC
Pp1s412_23V6.1     ------GV----------YG----------------MSGG-------TSGGSLRLG-MGMERC
Pp1s81_144V6.2     ------GV----------YG----------------MSGG-------SPGGSLRLG-MGMDRS
Smo16699|PACid_    ---------------------------------------------------------------
Smo38684|PACid_    ---------------------------------------------------------------
Smo438647|PACid    ------------------------------------------------QVLQINDPRAAAAAA
MA_71728g0010      ------------------------------------------------PMVQGGLP-NEDSSM
MA_10316458g001    ------GE----------IG----------------CNGT-------GSVNKASSM-DDSENN
MA_124244g0020     ------MP----------SG----------------SLES-------EKTGPLQVA-VAIERV
LOC_Os02g40510     ---------------------------------------------FYYPGMMDHGMTHPPVQN
LOC_Os11g05930     -------------------R----------------PFPP-------SFRVVH----------
LOC_Os03g17570     ------GG----------SC----------------SPQD-------NSSEAL----------
LOC_Os07g49460     ------MG----------SC----------------SLHD-------NSSEAM----------
LOC_Os09g36220     -------------------A-------------------------------------------
At2g46790          -------------------V----------------ALEE-------STSGEP----------
At5g02810          ------LG----------ST----------------SLQD-------NNSQDL---------I
At5g24470          -------------------A----------------SP------------VVT----------
At5g60100          ------VE----------SC----------------SPHD-------SPIAK-----------
At5g61380          E---------------------------------------TLQNGASYPHSLERSRTLPTSME

cons
```

```
Ot13g01890          -------------------------------------------------L----DL-TQQ---
Cre16.g676421.t     --------------------------------------------------S--QGV-SPSHYA
Cre02.g094150.t     VGEGDRAK-P--------EPHPQ--------------N-----------PA--QAA-HLPHPS
kfl00165_0030_v     -QM----P-THLLP-----------------PDFSNPRTVEAGPEM-QGPSRLGSG-EV--SS
kfl00059_0230_v     ADY----------------------------------------------SS-GGSGRDH--S-
Pp1s81_131V6.2      GSS----K-ESV------------------------KG------LT-S-PLSHPQN-VE---K
Pp1s412_35V6.1      GSS----K-GSA------------------------EG------TT-P-PPLHPQS-AE---A
Pp1s412_23V6.1      GSS----K-GSA------------------------EG------TT-P-PPLHPQS-AE---A
Pp1s81_144V6.2      GSS----K-GSV------------------------EG------TT-P-PPSHPQS-ME---K
Smo16699|PACid_     ---------------------------------------------------------------
Smo38684|PACid_     ---------------------------------------------------------------
Smo438647|PACid     AHA--------------------------------------------A-TG-VFN-QAH--II
MA_71728g0010       NNR--------------------------------------------G-TS-SLS-ASH--FL
MA_10316458g001     GKN----D-HYN------------------------HL------HH-N-GMHHHL--------
MA_124244g0020      GSS----K-GSG-G-----------------P--D--P------S-TP-ALMHNQL--S--QS
LOC_Os02g40510      FQG----------------------------------------------NI-NNA-QVH----
LOC_Os11g05930      --Q-----------QPVYDKNPQSSRVLLSCEHNTREST------VQA-QVPLDRS-T-----
LOC_Os03g17570      -KT----D-SNCKV----------------------KS------NS-D-AAEIKQG-SN--GS
LOC_Os07g49460      -KT----D-SAYNM----------------------KS------NS-D-AAPIKQG-SN--GS
LOC_Os09g36220      --------------GDAQACSTSATHIDLENKNGDSKTP------SQD---------------
At2g46790           --K----TPTESHEKLRKVTSDQGSATTSSNQENIGS--------------------------
At5g02810           KKT----E-AAYDC--------------------H--S------NMNE-SLPHNHR-SH--VG
At5g24470           --D-----------QRKNVAASQDDNIVLMNQYNTSEPP------PNA-PRRNDTS-FY-TGA
At5g60100           -----------------------------------------------------LLG-SS--SS
At5g61380           SHG--------------------------------------------R-NY-QEG-NMN----

cons

Ot13g01890          --------------------------------------QHQSFYEASSMMP-------PA---
Cre16.g676421.t     SLHTHGGGGG------------GPSSGGTGSGGGA-A-AAAAPSAAL---G-H-----PPQ-G
Cre02.g094150.t     AAPCSGGGGI------------AQAALPLGLQELA-A-LGAARHKELWTQR-H-----LMH-Q
kfl00165_0030_v     AAKRQ----TGGWGAEPAGPAPP-QM-------------------K---E------------E
kfl00059_0230_v     -------------------------P----PTVTPPREER----S------------------
Pp1s81_131V6.2      AGGQD--------------------------GCSS-A-TQ----TT---E------------D
Pp1s412_35V6.1      AGGQD----G------------G-GA----DGYGS-A-RQ----SA---E------------E
Pp1s412_23V6.1      AGGQD----G------------G-GA----DGYGS-A-RQ----SA---E------------E
Pp1s81_144V6.2      VGGQD--------------------------GYGN-A-RQ----TT---E------------D
Smo16699|PACid_     ---------------------------------------------------------------
Smo38684|PACid_     ---------------------------------------------------------------
Smo438647|PACid     PPLMV--------------------P----HSIHPST--------------------------
MA_71728g0010       PDMMN-------------------------HSMSSSM--------------------------
MA_10316458g001     ------------------------------S-HED-R-LS----KQ---D------------K
MA_124244g0020      SNNQD---------------L-G-SS----VV------GP-----S----GQDMFPVPKPIND
LOC_Os02g40510      ----T--------------------P----QTLLPQYNVY----P------------------
LOC_Os11g05930      ---------------------------------------------------------------
LOC_Os03g17570      SNNND---------------M-G-SS-------------------T---K------------N
LOC_Os07g49460      SNNND---------------M-G-ST-------------------T---K------------N
LOC_Os09g36220      --------------------------------------------------------------K
At2g46790           ---------------------------------------------------------------
At5g02810           SNNFD---------------M-S-ST-------------------T---E------------N
At5g24470           DSPGP-------------------------------------------------------PFS
At5g60100           SD-------------------------------------------------------------
At5g61380           ----I--------------------P----QVAMNRSKDS----S------------------

cons

Ot13g01890          ----------------DFARFYGPIMP-----------------------------------A
Cre16.g676421.t     GLS--A------AAWLGPSVSAGAAAAPH-------------------------------PHT
Cre02.g094150.t     RQA--A------AAATAAAASAAAAAA-----------------------------------M
kfl00165_0030_v     -Y--SA------GPLGPSSMT-----IPP-------------------------------QG-
kfl00059_0230_v     ---------------------------------------------------------------
Pp1s81_131V6.2      -AL--I------VPGMPMAIP-----IPP-------------------------------PG-
Pp1s412_35V6.1      -AM--I------APGMPIAIP-----IPP-------------------------------PG-
Pp1s412_23V6.1      -AM--I------APGVPMAIP-----IPP-------------------------------PG-
```

```
Pp1s81_144V6.2     -AM--I------VPGMPMAIP-----LPP-------------------------------PG-
Smo16699|PACid_    ---------------------------------------------------------------
Smo38684|PACid_    ---------------------------------------------------------------
Smo438647|PACid    ---------------------------------------------------------------
MA_71728g0010      ---------------------------------------------------------------
MA_10316458g001    -QI--I------KNSG-----------------------------------------------
MA_124244g0020     ET--VSAPNACRQIGKPMSIH------PR-------------------------------RA-
LOC_Os02g40510     ---------------------------------------------------------------
LOC_Os11g05930     --------------EGA-AIL-----CSSSVREDAGTSSSSPRKDSLTHPSYGFIPVPIPVG-
LOC_Os03g17570     -A--IT------KPSSNRGKV-----ISP-------------------------------SA-
LOC_Os07g49460     -V--VT------KPSTNKERV-----MSP-------------------------------SA-
LOC_Os09g36220     RETNQ--------PPIR-VVP-----FPV------------------------------PVG-
At2g46790          -------------------------------------SSVSF-RNQVLQSTVTNQKQDSPIP-
At5g02810          NA--F------TKPGAPKVSS-----AGS-------------------------------SS-
At5g24470          NQLNS-------WPGQS-SYP-----TPT------------------------------PIN-
At5g60100          N--------------------------------------------------------------
At5g61380          ---------------------------------------------------------------

cons

Ot13g01890         GMPPPPMDLP-----------------------------------------------------
Cre16.g676421.t    PHSHLVGSAPG---VPPPGL-----PA-------GFQNMFTAPFGL-------PHGHVLSRM-
Cre02.g094150.t    PTAGASAAAPA---GPPSAR-----P-----------------------------SASLADT-
kfl00165_0030_v    ------FMP-P-G-FPEPGF-----GPFPPHFGYYFQ-----PQNT-------GTAPTWNSA-
kfl00059_0230_v    ---------QS---F-GEAASGMFGM----------------PP-QMQNGMPPQ-IPNGMASN
Pp1s81_131V6.2     ------MLAYD---GVGGSY-----GP-------AMH-----PMYY-------AHPSARIAA-
Pp1s412_35V6.1     ------MLAYD---GMGGAY-----GP-------AMH-----PMYY-------AHAGAWMAA-
Pp1s412_23V6.1     ------MLAYD---GMGGAY-----GP-------AMH-----PMYY-------AHASAWMAA-
Pp1s81_144V6.2     ------MLAYD---GVIGTY-----GP-------AMH-----PMYY-------AHPSAWMAA-
Smo16699|PACid_    ---------------------------------------------------------------
Smo38684|PACid_    ---------------------------------------------------------------
Smo438647|PACid    ---------------------------------------------------------------
MA_71728g0010      --------------------HLCHGV----------------PH-D--V--------------
MA_10316458g001    ---------------------------------------------------------------
MA_124244g0020     -------MPYDTTVPAPRAY-----GS-------AIH-----PIYY-------SHTGASLWG-
LOC_Os02g40510     ---------------------------------------------------------------
LOC_Os11g05930     ------A-------AIPYHY-----GA-------IMQ-----PMYY-------PQGAFMHCD-
LOC_Os03g17570     ------VK-----------------A---TQHTSAFH-----PVQR-------QTSPANVVG-
LOC_Os07g49460     ------VK-----------------A---NGHTSAFH-----PAQH-------WTSPANTTG-
LOC_Os09g36220     ------GLTFD---GQPFWN-----GA-------PVA-----SLFY-------PQSAP-----
At2g46790          ------V--------------------------------------------------------
At5g02810          -------------V-----K-----HS-------SFQ-----PLPC-------DH--------
At5g24470          ------NIQFR---DPNTAY-----TS-------AMA-----PASL-------SPS-PSSV--
At5g60100          ---------------------------------------------------------------
At5g61380          ---------QV---DGSG-FSAPNAY----------------PY-Y--MHGVMN------QVM

cons

Ot13g01890         ---------------------------------------------------------------
Cre16.g676421.t    ----------AHP--GLR---F-------------------GPGAGNGHGGNPQAAGAPLDPA
Cre02.g094150.t    ----------GGD--GPA---A------A-TAPETRADGPSGPATTQGPKRDAVAGAAAVGSS
kfl00165_0030_v    ----------PP-----------RKDPPNPPLPPA---------KPQN---------------
kfl00059_0230_v    GIPNGMAPQ-LP--NGMQ---LPS--D-----------------LL-----------------
Pp1s81_131V6.2     ----------P-P--PHM---GERGEVYN-QSPAF---------KEQD---------------
Pp1s412_35V6.1     ----------S-A--RHM---GERVDVYS-QTPAF---------QEQN---------------
Pp1s412_23V6.1     ----------S-A--RHM---GERVDVYS-QTPAF---------QEQN---------------
Pp1s81_144V6.2     ----------P-S--RHM---GERGDVYN-QSPAF---------QEQD---------------
Smo16699|PACid_    ---------------------------------------------------------------
Smo38684|PACid_    ---------------------------------------------------------------
Smo438647|PACid    ----------LL--Q------------------------------------------------
MA_71728g0010      -------GS-RV--T------------------------------------------------
MA_10316458g001    ---------------------------------------------------------------
MA_124244g0020     ----------PEA--QHV---NDRGDVCN-NSSEQ---------QLML---------------
LOC_Os02g40510     ---------------------------------------------------------------
LOC_Os11g05930     ----------SAA--INK---T-----------AI---------QHVS---------------
LOC_Os03g17570     ----------KD-----------KVDEGIANG--V---------NVGH---------------
```

```
LOC_Os07g49460    ----------KE-----------KTDEVANNA--A---------KRAQ---------------
LOC_Os09g36220    ------------P--IWNSKTSTWQDATT-QAISL---------QQNG---------------
At2g46790         ---------------------------------------------------------------
At5g02810         ---------------------------------------------------------------
At5g24470         ------------SPHEY----SSMFHPFN-SKP------------------------------
At5g60100         ---------------------------------------------------------------
At5g61380         MQSAAMMPQYGH--Q------------------------------------------------

cons

Ot13g01890        ---------------------------------------------------------------
Cre16.g676421.t   TQLQSMFHFPSAEMVALAAAASEAHAHAA-ARSHGHGPHGPGAHGLLLGPLPGGPGGGAEGPG
Cre02.g094150.t   ARSDSPLP-AAAAATAGANG-------------------------------------------
kfl00165_0030_v   --PKDVSIH-------H-----HHHHHHHTHH-H-----------------------------
kfl00059_0230_v   ----------------------M----------------------------------------
Pp1s81_131V6.2    --SGSGHHS-------Q-----AGQSHQHMHH-H-----------------------------
Pp1s412_35V6.1    --PCSGHHS-------Q-----AGPSHQHTHH-H-----------------------------
Pp1s412_23V6.1    --PGSGHHS-------Q-----AGPSHQHTHH-H-----------------------------
Pp1s81_144V6.2    --SGSGNHS-------Q-----AGQTHQHMHH-H-----------------------------
Smo16699|PACid_   ---------------------------------------------------------------
Smo38684|PACid_   ---------------------------------------------------------------
Smo438647|PACid   ---------------------------------------------------------------
MA_71728g0010     ---------------------------------------------------------------
MA_10316458g001   -------------------------LTPLLCG-S-----------------------------
MA_124244g0020    --PR--NQQ-------T-----QIHHHHHHHV-Q-----------------------------
LOC_Os02g40510    ---------------------------------------------------------------
LOC_Os11g05930    --CQSNYHE-------N-----LGKPPQIDEH-K-----------------------------
LOC_Os03g17570    --PV--DVQ-------N-----SFMQHHH----------------------------------
LOC_Os07g49460    --PG--EVQ-------S-----NLVQHPR----------------------------------
LOC_Os09g36220    --PKDTDTK-------Q-----VENVEEQTAR-------------------------------
At2g46790         ---------------------------------------------------------------
At5g02810         -------------------------H-NNH---------------------------------
At5g24470         ---------------------------------------------------------------
At5g60100         ---------------------------------------------------------------
At5g61380         ---------------------------------------------------------------

cons

Ot13g01890        ---------------------------------------------------------------
Cre16.g676421.t   NGGPAGGGGGGNGGLAGVPPPPTSGATNINAMILNQLLANGVTLQAFLGPQAGPLGIHHA---
Cre02.g094150.t   ASGAASDVLAGAGSLALLRHSDRSAFTAFTVFLPGRVAAAAAAAA--------------A---
kfl00165_0030_v   ---------------------------------------------------DANQR-HHDASP
kfl00059_0230_v   ---------------------------------------------------------------
Pp1s81_131V6.2    ---------------------------------------------------QGN--HHHH---
Pp1s412_35V6.1    ---------------------------------------------------QGNQHHHHH---
Pp1s412_23V6.1    ---------------------------------------------------------------
Pp1s81_144V6.2    ---------------------------------------------------QGNQ-HHHH---
Smo16699|PACid_   ---------------------------------------------------------------
Smo38684|PACid_   ---------------------------------------------------------------
Smo438647|PACid   ---------------------------------------------------------------
MA_71728g0010     ---------------------------------------------------------------
MA_10316458g001   ---------------------------------------------------CLIG--QSV---
MA_124244g0020    ----------------------------------------------------H----------
LOC_Os02g40510    ---------------------------------------------------------------
LOC_Os11g05930    ---------------------------------------------------QPEEN-HQL---
LOC_Os03g17570    ---------------------------------------------------------------
LOC_Os07g49460    ---------------------------------------------------------------
LOC_Os09g36220    -------------------------------------------------------S-H-L---
At2g46790         ---------------------------------------------------------------
At5g02810         ---------------------------------------------------------------
At5g24470         ---------------------------------------------------------------
At5g60100         ---------------------------------------------------------------
At5g61380         ---------------------------------------------------------------

cons
```

```
Ot13g01890          ----------------------------------LP-------PPPYIDM-------------
Cre16.g676421.t     ------------AAAAG--S------RG--GAQHPATSGLPPHQQQQAAA-------------
Cre02.g094150.t     ------------AAAAA--T------SA--GASTGTANGAPPAPGTALAA-------------
kfl00165_0030_v     RHHDGPPERHPKHNHHKVSHP--QATR--AADAPGGESGLAGSSRQEIAQSRAEQAGKVAAHL
kfl00059_0230_v     ----------------------------------------------NPHL-------------
Pp1s81_131V6.2      ------------HHHYH--H------GN--GAQHSGNAGVQDEQQQSVVT-------------
Pp1s412_35V6.1      ------------HHHHH--H------GD--GGQPSGTAGVQDEQQQSVIT-------------
Pp1s412_23V6.1      -------------------H------GD--GGQPSGNAGVQHEQQQSVIT-------------
Pp1s81_144V6.2      ------------HHHHH--H------GS--GAQPSGNAGVQDEQQQSVVP-------------
Smo16699|PACid_     ---------------------------------------------------------------
Smo38684|PACid_     ---------------------------------------------------------------
Smo438647|PACid     ----------------------------------------------PPIH-------------
MA_71728g0010       ----------------------------------------------PGLM-------------
MA_10316458g001     ------------SSNGYESV------NASEHGSNN----------------------------
MA_124244g0020      -------------VHYH--EHHHQTHQHKHNHTHTQDH-PKQD-EQTKNN-------------
LOC_Os02g40510      ---------------------------------------------------------------
LOC_Os11g05930      ------------HHSRQI-LR-----E---SGEPVD--------LAKAHM-------------
LOC_Os03g17570      ------------HVHY-------------------------------------------YVHV
LOC_Os07g49460      ------------PILH-------------------------------------------YVHF
LOC_Os09g36220      ------------SANRKH-LR-----I------------------------------------
At2g46790           ---------------------------------------------------------------
At5g02810           -------------ASYN--L-------------------------VHVAE-------------
At5g24470           -------------EGLQD-RD-----C------------------------------------
At5g60100           ---------------------------------------------------------------
At5g61380           ---------------------------------------------------------------

cons


Ot13g01890          -----------NQFT----A-------------------------------------------
Cre16.g676421.t     -----------AAVAAA-AAA----WRPP--------------------------------P-
Cre02.g094150.t     -----------AAAAAA-AAA----SAVPLPHPHTAPPALFGVPPPSSVPPSSLSVLPPVMP-
kfl00165_0030_v     DRAEHMEKRESHRMDGL-DTS----GGSDN---------P------------------N----
kfl00059_0230_v     -----------AMFNLP-P------GMM-------------------------------PPMP
Pp1s81_131V6.2      -----------LGSGAP-RCG----STGMD---------G------------------Q----
Pp1s412_35V6.1      -----------PMSGAP-RCG----STGVD---------G------------------Q----
Pp1s412_23V6.1      -----------PMSGAP-RCG----STGVD---------G------------------Q----
Pp1s81_144V6.2      -----------PGSSAP-RCG----STGVD---------G------------------R----
Smo16699|PACid_     ---------------------------------------------------------------
Smo38684|PACid_     ---------------------------------------------------------------
Smo438647|PACid     -----------YYQAVP-PPE----ATP-------------------------------IP-A
MA_71728g0010       -----------PFHALQ-PCH----GMP-------------------------------VN--
MA_10316458g001     ---------------------------RIN---------G------------------Q----
MA_124244g0020      -----------LPITPP-LCG----SSNMPGNNAPDGNNG------------------Q----
LOC_Os02g40510      ------------------QCH----GVS-------------------------------MM--
LOC_Os11g05930      -----------ERINQS-ASC----SQDIR---------K------------------G----
LOC_Os03g17570      MTQQQQQPSIERGSSDA-QCG----SSNVF---------D------------------P----
LOC_Os07g49460      DVSREN-----GGSGAP-QCG----SSNVF---------D------------------P----
LOC_Os09g36220      -------------------------EIPTD---------E------------------P----
At2g46790           ------------ESNRE-KAA----SKEVE---------A------------------G----
At5g02810           -----------RKKLPP-QCG----SSNVY---------N------------------E----
At5g24470           -------------------------SMDVD---------E------------------R----
At5g60100           --------------------P----LKQQS---------------------------------
At5g61380           ----------------IPHCQPNHPNGM-------------------------------TG--

cons


Ot13g01890          --------------------------AAFTSASIRPD----MMDS--------TAN-------
Cre16.g676421.t     --FNPAATMAA---------------AAAAAAGVMPF----PVGP---------PQ----P--
Cre02.g094150.t     --LHPAAAAAAATAGGG-KPSDAATYAAAAAAGLVPY----PG-FAPARPGPFPPP----P--
kfl00165_0030_v     --S--------SG-----GQSNANNT-----SGGGSN-------F--------GSN----GRS
kfl00059_0230_v     LPPGMPP----G-LAAGLM-GQMQFS-----PFFQNMLNPHLIRP--------PVQRVPTP-P
Pp1s81_131V6.2      --SGSSN----GYGSTG-NGNGSMNG-----SASGSN-------T--------GVNN---G--
Pp1s412_35V6.1      --SGSRN----GYGSTG-NGNGSMNG-----SASGSN-------T--------GVNN---G--
Pp1s412_23V6.1      --SGSSN----GYGSTG-NGNGSMNG-----SASGSN-------T--------GVNN---G--
Pp1s81_144V6.2      --SGSSN----GYGSTG-NGNGSMNG-----SASGSN-------T--------GVNN---G--
```

```
Smo16699|PACid_     ---------------------------------------------------------------
Smo38684|PACid_     ---------------------------------------------------------------
Smo438647|PACid     -AAFSYY----PFPLHIPS-QQ-----------V-------------------P---------
MA_71728g0010       -ATMPYY----PYGFLVAP-AQ-----------L-------------------GPSH------
MA_10316458g001     --AGHSS----GQSSGD-IVTGMNGE-----S--GT---------------------------
MA_124244g0020      --SGSNN----GYGNSG-NGNGSANG-----SATGSN-------N--------GSN----G--
LOC_Os02g40510      -PPFQYN----PAGMSIQS-NQ-----------L-------------------PTQN------
LOC_Os11g05930      --------------------SGCTG-------------------S--------GET----D--
LOC_Os03g17570      --P--------IEGH---AANYSVNG-----SFSGGH-------N--------GNN----G--
LOC_Os07g49460      --P--------VEGH---AANYGVNG-----SNSGSN-------N--------GSN----G--
LOC_Os09g36220      --R--------HVS------PTTGE-------------------S--------GSS----T--
At2g46790           --------------------SQSTN-------------------E--------GIA----G--
At5g02810           --T--------IEGNNN-TVNYSVNG-----SVSGSG-------H--------GSN----G--
At5g24470           --R--------YVS------SATEH-------------------S--------AIG----N--
At5g60100           ---------------------------------------------------------------
At5g61380           -YPYYHH----PMNTSLQH-SQ-----------M-------------------SLQNGQMS-M

cons

Ot13g01890          -------------P----------F---------WS------V----LQTAADHTQQ------
Cre16.g676421.t     --G--GGPPLPPD--ASAA--AALI---L------GQ-QGLQP----AMLGGTRPTA------
Cre02.g094150.t     --GS-GGPGAPPVYIPESV--LQLIAHLSGRAAAEIP-AVPAE----SVTAAPVVVQ------
kfl00165_0030_v     SATPGSGATTETGTGTGAS--GTGAE-----ANQQTP-AHAPQ----AGASDM--AL------
kfl00059_0230_v     PPPNAQPQSKPPQVRSARF--SPPPPT---------T-GGPVI------GVS---VGGPPAPM
Pp1s81_131V6.2      --QSG-------LGAMLMA--NDNSGSNGAGGTDPSV-DGVSG-------GN---GL------
Pp1s412_35V6.1      --QNG-------LGVTPMA--NANSGNNGVGGTDPAM-DGVSG-------GN---GL------
Pp1s412_23V6.1      --QNG-------LVVTPMA--NANSGNNGVGGTHPAM-DGVSG-------GN---GL------
Pp1s81_144V6.2      --QSG-------FGATPML--TDNSGSNGVGGTDAAM-DGVSG-------GN---GL------
Smo16699|PACid_     ---------------------------------------------------------------
Smo38684|PACid_     ---------------------------------------------------------------
Smo438647|PACid     ----------------------WNA-----------A-ALPQV------GL------------
MA_71728g0010       ---------------------AWPG-----------M-AN----------LS---VS------
MA_10316458g001     ---------------------------------VNGV-MRSTG-------TS---GG------
MA_124244g0020      --QNGYSNGQNSAAVTPGG--INGESDTGIGAVKSST-GGASG----TGSGS-----------
LOC_Os02g40510      ---------------------MWPQ-----------A-SS----------TP---MP------
LOC_Os11g05930      -----------AN------------T-----NTVIALESGNES--G-VQNC-----S------
LOC_Os03g17570      --QRGPSTAPNVGRPNMET--VNGIV------DENGA-GGGNG----SGSGS---GN------
LOC_Os07g49460      --QNGSTTAVNAERPNMEI--ANGTI------NKSGP-GGGNG----SGSGS---GN------
LOC_Os09g36220      --------VLDSARKTLSGSVCDSSS-----NHMIAP---TESSNV-VPE------N------
At2g46790           -----------Q-------------------------SSSTEK--P--KEE------------
At5g02810           --PYGSSNGMNAGGMNMGS--DNGAG-----KNGNGD-GSGSG----SGSGS---GN------
At5g24470           --------HIDQL--------------------IEK-----KN-EDGYSL------S------
At5g60100           -------------------------------------------------SGS-----------
At5g61380           V--------------HH----SWSP-----------A-GN----------PP---SN------

cons

Ot13g01890          --------TSSS--QA---AEHRAAAIRRFLKKRKERNFDKKVRYASRQQLAASRPRLRGQFV
Cre16.g676421.t     --------PGMP--EP---KRRRALALDKYRKKRKNLRFSKTIRYESRKQLAQQRPRVRGQFI
Cre02.g094150.t     --------KSGG--PA---SAARLAAVAKYLEKRKHRNFQKKVRYESRKRLAEARPRVRGQFV
kfl00165_0030_v     ------DGMEEV--DR---SARREAALNKFRQKRKERNFEKKVRYQSRKRLAEQRPRIRGQFV
kfl00059_0230_v     WPLLQAGVDRKM--DQ---AERREAALSKFRQKRKDRCFAKKIRYASRKKLAEARPRIKGQFV
Pp1s81_131V6.2      --------CTDQ--MR---FARREAALNKFRQKRKERCFEKKVRYQSRKRLAEQRPRVRGQFV
Pp1s412_35V6.1      --------CTEQ--IR---FARREAALNKFRQKRKERCFEKKVRYQSRKKLAEQRPRVRGQFV
Pp1s412_23V6.1      --------CTEQ--IR---FARREAALNKFRQKRKERCFEKKVRYQSRKKLAEQRPRVRGLFV
Pp1s81_144V6.2      --------CTEQ--MR---FARREAALNKFRQKRKERCFEKKVRYQSRKRLAEQRPRVRGQFV
Smo16699|PACid_     ---------------------------------------------------------------
Smo38684|PACid_     ---------------------------------------------------------------
Smo438647|PACid     --------ERKA--GV---AERREAALHKFRQKRKDRCYEKKIRYASRKRLAEQRPRVKGQFV
MA_71728g0010       --------GPKI--SQ---VERREAALNKFRQKRKDRCFDKKIRYVSRKRLAEQRPRVRGQFV
MA_10316458g001     --------GVDQ--NH---SARREAALKKFRLKRKERCFEKKVRY------------------
MA_124244g0020      --------GIDI--NR---SAQREAALTKFRQKRKERCFEKKVRYQSRKKLAEQRPRVRGQFV
LOC_Os02g40510      --------EETC--SR---SERRAAALAKFRLKRKERCFDKKVRYVNRKKLAETRPRVRGQFV
LOC_Os11g05930      --------NNVLD-GD---RSRREAALLKFRMKRKDRCFEKKVRYHSRKKLAEQRPRVKGQFV
LOC_Os03g17570      --------DLYQ--NG---VCYREAALNKFRQKRKVRNFGKKVRYQSRKRLAEQRPRIRGQFV
LOC_Os07g49460      --------DMYL--KRFTQREHRVAAVIKFRQKRKERNFGKKVRYQSRKRLAEQRPRVRGQFV
```

```
LOC_Os09g36220     ---------PDGL--RH---LSQREAALNKFRLKRKDRCFEKKVRYQSRKLLAEQRPRVKGQFV
At2g46790          ---------ESAKQRWS---RSQREAALMKFRLKRKDRCFDKKVRYQSRKKLAEQRPRVKGQFV
At5g02810          ---------LADE--NK---ISQREAALTKFRQKRKERCFRKKVRYQSRKKLAEQRPRVRGQFV
At5g24470          ---------VGKI--QQ---SLQREAALTKFRMKRKDRCYEKKVRYESRKKLAEQRPRIKGQFV
At5g60100          ---------------DR---WAQREAALMKFRLKRKERCFEKKVRYHSRKKLAEQRPHVKGQFI
At5g61380          ---------EVRV--NK---LDRREEALLKFRRKRNQRCFDKKIRYVNRKRLAERRPRVKGQFV

cons

Ot13g01890         RNAEETTTENGSNGSDGK---------------------------------------------
Cre16.g676421.t    KHGDGDGGAGGGLEGAGGSGLMLVDDGAATASNGAKSGTGGAGGVGGLEDAGAEDEVQTNSR
Cre02.g094150.t    KAGTAGAAAAAAAAAAAAAAGT-----------------------------------------
kfl00165_0030_v    RQATFPNAV------------------------------------------------------
kfl00059_0230_v    RQKEGGAPGG-----------------------------------------------------
Pp1s81_131V6.2     RQAVYDPSA------------------------------------------------------
Pp1s412_35V6.1     RQAAHDPSA------------------------------------------------------
Pp1s412_23V6.1     RQAAHDPSA------------------------------------------------------
Pp1s81_144V6.2     RQAVHDPSA------------------------------------------------------
Smo16699|PACid_    ---------------------------------------------------------------
Smo38684|PACid_    ---------------------------------------------------------------
Smo438647|PACid    RRAEEYEDED-----------------------------------------------------
MA_71728g0010      RQTNDMEAGA-----------------------------------------------------
MA_10316458g001    ---------------------------------------------------------------
MA_124244g0020     RQTTHETAN------------------------------------------------------
LOC_Os02g40510     RQANYTDITS-----------------------------------------------------
LOC_Os11g05930     SQKLKSAIT------------------------------------------------------
LOC_Os03g17570     RQSGQEDQA------------------------------------------------------
LOC_Os07g49460     RQAVQDQQQ------------------------------------------------------
LOC_Os09g36220     RQDHGVQG-------------------------------------------------------
At2g46790          RTVNSDAST------------------------------------------------------
At5g02810          RKTAAATDD------------------------------------------------------
At5g24470          RQVQSTQA-------------------------------------------------------
At5g60100          RKRDDHKSG------------------------------------------------------
At5g61380          RKMNGVNVDL-----------------------------------------------------

cons

Ot13g01890         ----------------KSNEFNASAA-----------------------------------KG
Cre16.g676421.t    GYYEEEDYDAEEAEDETEAEPDDMDAAQPASQHAQPQQTNDTPDDMDVDTAAGLGGAAAAAAD
Cre02.g094150.t    ---------------------------------------------------------------
kfl00165_0030_v    ---------------------------------------------------------------
kfl00059_0230_v    ---------------------------------------------------------------
Pp1s81_131V6.2     ---------------------------------------------------------------
Pp1s412_35V6.1     ---------------------------------------------------------------
Pp1s412_23V6.1     ---------------------------------------------------------------
Pp1s81_144V6.2     ---------------------------------------------------------------
Smo16699|PACid_    ---------------------------------------------------------------
Smo38684|PACid_    ---------------------------------------------------------------
Smo438647|PACid    ---------------------------------------------------------------
MA_71728g0010      ---------------------------------------------------------------
MA_10316458g001    ---------------------------------------------------------------
MA_124244g0020     ---------------------------------------------------------------
LOC_Os02g40510     ---------------------------------------------------------------
LOC_Os11g05930     ---------------------------------------------------------------
LOC_Os03g17570     ---------------------------------------------------------------
LOC_Os07g49460     ---------------------------------------------------------------
LOC_Os09g36220     ---------------------------------------------------------------
At2g46790          ---------------------------------------------------------------
At5g02810          ---------------------------------------------------------------
At5g24470          ---------------------------------------------------------------
At5g60100          ---------------------------------------------------------------
At5g61380          ---------------------------------------------------------------

cons
```

```
Ot13g01890         EVQGREEQY-------VDRHG--------------------------------------SNTG
Cre16.g676421.t    GTAAGGAAAAAAGADGVARLAGSAVGGGSGGSEGQPSSGPDGGMAAAVGAADAGQGSGAAAGD
Cre02.g094150.t    -------AAT-AAGTGTARGAATASGAAGKPEL----QGPDTAEEAAAATLLSAAAAMAAAAA
kf100165_0030_v    ---------------------------------------------------------------
kf100059_0230_v    ---------------------------------------------------------------
Pp1s81_131V6.2     ---------------------------------------------------------------
Pp1s412_35V6.1     ---------------------------------------------------------------
Pp1s412_23V6.1     ---------------------------------------------------------------
Pp1s81_144V6.2     ---------------------------------------------------------------
Smo16699|PACid_    ---------------------------------------------------------------
Smo38684|PACid_    ---------------------------------------------------------------
Smo438647|PACid    ---------------------------------------------------------------
MA_71728g0010      ---------------------------------------------------------------
MA_10316458g001    ---------------------------------------------------------------
MA_124244g0020     ---------------------------------------------------------------
LOC_Os02g40510     ---------------------------------------------------------------
LOC_Os11g05930     ---------------------------------------------------------------
LOC_Os03g17570     ---------------------------------------------------------------
LOC_Os07g49460     ---------------------------------------------------------------
LOC_Os09g36220     ---------------------------------------------------------------
At2g46790          ---------------------------------------------------------------
At5g02810          ---------------------------------------------------------------
At5g24470          ---------------------------------------------------------------
At5g60100          ---------------------------------------------------------------
At5g61380          ---------------------------------------------------------------

cons

Ot13g01890         TSN----EGS-------------------KEGSRSSS--------------------------
Cre16.g676421.t    GSGGSRAAGGAGSGVSGADEEVEAVVVRSLVGLREAAIAARSRPLGSMDAAPLLQEAKASRSV
Cre02.g094150.t    GTSGPSGSGSGAMDVDGADPEADADVMDEDDGEDDGS--------------------------
kf100165_0030_v    -------------------------------------QQFV--RP------------------
kf100059_0230_v    --------------------------------------GGD--EE------------------
Pp1s81_131V6.2     -------------------------------------VTSR--SF------------------
Pp1s412_35V6.1     -------------------------------------GDA-----------------------
Pp1s412_23V6.1     -------------------------------------GDA-----------------------
Pp1s81_144V6.2     -------------------------------------GDA-----------------------
Smo16699|PACid_    ---------------------------------------------------------------
Smo38684|PACid_    ---------------------------------------------------------------
Smo438647|PACid    --------------------------------------EDD----------------------
MA_71728g0010      --------------------------------------NGV--IY------------------
MA_10316458g001    ---------------------------------------------------------------
MA_124244g0020     -------------------------------------GEG-----------------------
LOC_Os02g40510     --------------------------------------TGD--DI------------------
LOC_Os11g05930     -------------------------------------TEAE----------------------
LOC_Os03g17570     -------------------------------------GQ------------------------
LOC_Os07g49460     -------------------------------------QGGG--RE------------------
LOC_Os09g36220     ---------------------------------------------------------------
At2g46790          -------------------------------------K-------------------------
At5g02810          -------------------------------------NDIK--NI------------------
At5g24470          ---------------------------------------------------------------
At5g60100          -------------------------------------SED-----------------------
At5g61380          --------------------------------------NGQ--PD------------------

cons

Ot13g01890         ---------------------------------------------------------------
Cre16.g676421.t    GSQGSLSRDRGYSRGHSRGHSRGHSHGHSHSQQQQQQVVAQQQTGGLGAAGGPTSQGISHGRN
Cre02.g094150.t    ---------------------------------------------------------------
kf100165_0030_v    ---------------------------------------------------------------
kf100059_0230_v    ---------------------------------------------------------------
Pp1s81_131V6.2     ---------------------------------------------------------------
Pp1s412_35V6.1     ---------------------------------------------------------------
Pp1s412_23V6.1     ---------------------------------------------------------------
Pp1s81_144V6.2     ---------------------------------------------------------------
Smo16699|PACid_    ---------------------------------------------------------------
```

```
Smo38684|PACid_         ---------------------------------------------------------------
Smo438647|PACid         ---------------------------------------------------------------
MA_71728g0010           ---------------------------------------------------------------
MA_10316458g001         ---------------------------------------------------------------
MA_124244g0020          ---------------------------------------------------------------
LOC_Os02g40510          ---------------------------------------------------------------
LOC_Os11g05930          ---------------------------------------------------------------
LOC_Os03g17570          ---------------------------------------------------------------
LOC_Os07g49460          ---------------------------------------------------------------
LOC_Os09g36220          ---------------------------------------------------------------
At2g46790               ---------------------------------------------------------------
At5g02810               ---------------------------------------------------------------
At5g24470               ---------------------------------------------------------------
At5g60100               ---------------------------------------------------------------
At5g61380               ---------------------------------------------------------------

cons


Ot13g01890              ---------------------------------------------------------------
Cre16.g676421.t         IIMPQPTGAPSLSAHLQSQLLQAAAPAAPSAHSSPTNSQAALAAVTGQAPSSAPQQAQMLVAS
Cre02.g094150.t         ---------------------------------------------------------------
kfl00165_0030_v         ---------------------------------------------------------------
kfl00059_0230_v         ---------------------------------------------------------------
Pp1s81_131V6.2          ---------------------------------------------------------------
Pp1s412_35V6.1          ---------------------------------------------------------------
Pp1s412_23V6.1          ---------------------------------------------------------------
Pp1s81_144V6.2          ---------------------------------------------------------------
Smo16699|PACid_         ---------------------------------------------------------------
Smo38684|PACid_         ---------------------------------------------------------------
Smo438647|PACid         ---------------------------------------------------------------
MA_71728g0010           ---------------------------------------------------------------
MA_10316458g001         ---------------------------------------------------------------
MA_124244g0020          ---------------------------------------------------------------
LOC_Os02g40510          ---------------------------------------------------------------
LOC_Os11g05930          ---------------------------------------------------------------
LOC_Os03g17570          ---------------------------------------------------------------
LOC_Os07g49460          ---------------------------------------------------------------
LOC_Os09g36220          ---------------------------------------------------------------
At2g46790               ---------------------------------------------------------------
At5g02810               ---------------------------------------------------------------
At5g24470               ---------------------------------------------------------------
At5g60100               ---------------------------------------------------------------
At5g61380               ---------------------------------------------------------------

cons


Ot13g01890              ---------------------------------------------------------------
Cre16.g676421.t         AAAAGGVAGSVGGGLQAPGAGLQHPPGSLGCGLAGATPSGLRGSSGSGGGGHSHGAAAGGATN
Cre02.g094150.t         ---------------------------------------------------------------
kfl00165_0030_v         ---------------------------------------------------------------
kfl00059_0230_v         ---------------------------------------------------------------
Pp1s81_131V6.2          ---------------------------------------------------------------
Pp1s412_35V6.1          ---------------------------------------------------------------
Pp1s412_23V6.1          ---------------------------------------------------------------
Pp1s81_144V6.2          ---------------------------------------------------------------
Smo16699|PACid_         ---------------------------------------------------------------
Smo38684|PACid_         ---------------------------------------------------------------
Smo438647|PACid         ---------------------------------------------------------------
MA_71728g0010           ---------------------------------------------------------------
MA_10316458g001         ---------------------------------------------------------------
MA_124244g0020          ---------------------------------------------------------------
LOC_Os02g40510          ---------------------------------------------------------------
LOC_Os11g05930          ---------------------------------------------------------------
LOC_Os03g17570          ---------------------------------------------------------------
LOC_Os07g49460          ---------------------------------------------------------------
LOC_Os09g36220          ---------------------------------------------------------------
```

```
At2g46790          ---------------------------------------------------------------
At5g02810          ---------------------------------------------------------------
At5g24470          ---------------------------------------------------------------
At5g60100          ---------------------------------------------------------------
At5g61380          ---------------------------------------------------------------

cons


Ot13g01890         ---------------------------------------------------------------
Cre16.g676421.t    DSALQPGSLQQQQQPGGLAAGGAAAAAQAAREPPHSREGRGEHRGETGAVGRDRDREPGGMAM
Cre02.g094150.t    ---------------------------------------------------------------
kfl00165_0030_v    ---------------------------------------------------------------
kfl00059_0230_v    ---------------------------------------------------------------
Pp1s81_131V6.2     ---------------------------------------------------------------
Pp1s412_35V6.1     ---------------------------------------------------------------
Pp1s412_23V6.1     ---------------------------------------------------------------
Pp1s81_144V6.2     ---------------------------------------------------------------
Smo16699|PACid_    ---------------------------------------------------------------
Smo38684|PACid_    ---------------------------------------------------------------
Smo438647|PACid    ---------------------------------------------------------------
MA_71728g0010      ---------------------------------------------------------------
MA_10316458g001    ---------------------------------------------------------------
MA_124244g0020     ---------------------------------------------------------------
LOC_Os02g40510     ---------------------------------------------------------------
LOC_Os11g05930     ---------------------------------------------------------------
LOC_Os03g17570     ---------------------------------------------------------------
LOC_Os07g49460     ---------------------------------------------------------------
LOC_Os09g36220     ---------------------------------------------------------------
At2g46790          ---------------------------------------------------------------
At5g02810          ---------------------------------------------------------------
At5g24470          ---------------------------------------------------------------
At5g60100          ---------------------------------------------------------------
At5g61380          ---------------------------------------------------------------

cons


Ot13g01890         ----------------------------------------------------RDG--------
Cre16.g676421.t    AAGGKNGSDSGSNSPDENGGGGGSGGGGGGGRVNKGGADKAGHVGHKSGSTHGHG--------
Cre02.g094150.t    --------DE------------------------------------------SAG--------
kfl00165_0030_v    ---------------------------GSQ-----------------------GE--------
kfl00059_0230_v    ---------------------------ELEEE------------------EE------EEEED
Pp1s81_131V6.2     ---------------------------QLAL----------------------VS--------
Pp1s412_35V6.1     ---------------------------------------------------------------
Pp1s412_23V6.1     ---------------------------------------------------------------
Pp1s81_144V6.2     ---------------------------------------------------------------
Smo16699|PACid_    ---------------------------------------------------------------
Smo38684|PACid_    ---------------------------------------------------------------
Smo438647|PACid    ---------------------------DE----------------------------------
MA_71728g0010      ---------------------------DVDSS------------------EDEDDRYAHGSSE
MA_10316458g001    ---------------------------------------------------------------
MA_124244g0020     ---------------------------------------------------------------
LOC_Os02g40510     ---------------------------SEDED------------------DD------PSSRE
LOC_Os11g05930     ---------------------------------------------------------------
LOC_Os03g17570     ----------------------------DE---------------------------------
LOC_Os07g49460     ---------------------------AAA---------------------------------
LOC_Os09g36220     ---------------------------------------------------------------
At2g46790          ---------------------------------------------------------------
At5g02810          ------------------------------------------------------E--------
At5g24470          ---------------------------------------------------------------
At5g60100          ---------------------------------------------------------------
At5g61380          ---------------------------SADYD------------------DE------EEEEE

cons


Ot13g01890         -------------------S---------------K
```

```
Cre16.g676421.t     -------------------H---------------R
Cre02.g094150.t     -------------------E---------------P
kf100165_0030_v     -------------------S---------------L
kf100059_0230_v     VDEEEEDENKGAQLPDISGSLNGSNDSQSALNIESR
Pp1s81_131V6.2      -------------------N---------------I
Pp1s412_35V6.1      -----------------------------------E
Pp1s412_23V6.1      -----------------------------------E
Pp1s81_144V6.2      -----------------------------------E
Smo16699|PACid_     ------------------------------------
Smo38684|PACid_     ------------------------------------
Smo438647|PACid     TG------RDMSPESGVEVT---------------Q
MA_71728g0010       LG------LASSPESLAGDPENAIQYQ--------R
MA_10316458g001     ------------------------------------
MA_124244g0020      -----------------------------------D
LOC_Os02g40510      VE------MVSSP----------------------E
LOC_Os11g05930      -------------------T---------------D
LOC_Os03g17570      -------------------D---------------R
LOC_Os07g49460      -------------------D---------------R
LOC_Os09g36220      -----------------------------------S
At2g46790           -----------------------------------S
At5g02810           -------------------D---------------S
At5g24470           -----------------------------------P
At5g60100           -----------------------------------N
At5g61380           EEEEEN--RDSSPQDDALG----------------T

cons
```

LUX

```
T-COFFEE, Version_11.00.d625267 (2016-01-11 15:25:41 - Revision d625267 - Build 507)
Cedric Notredame
CPU TIME:0 sec.
SCORE=429
*
 BAD AVG GOOD
*
Cpa_tig00001336   :  34
Popu_2051.13      :  47
Cre02.g083750.t   :  24
Cre09.g410450.t   :  31
kf100118_0040_v   :  28
kf100537_0090_v   :  47
Pp1s104_175V6.1   :  56
Pp1s29_32V6.1     :  55
Pp1s27_359V6.1    :  54
Pp1s29_23V6.1     :  54
Smo36646|PACid    :  64
MA_3352g0010      :  58
MA_303578g0010    :  53
LOC_Os01g74020    :  60
At3g46640         :  52
At5g59570         :  62
cons              :  42

Cpa_tig00001336    MREIK-------------R-----------AR-------------------------------
Popu_2051.13       MSSDDAVRHPATQRPSVYQ-----------RK-------------------------------
Cre02.g083750.t    MRLQ-----------QPNL-----------DPNTLGKSFDLDY--SSWSAGLWAPASGQERRT
Cre09.g410450.t    MAFD-------------------------------ASGALQSCYGAKEDANM-----------
kf100118_0040_v    MSEA-----------GTELNLKLLGAEVGELP-------------------------------
kf100537_0090_v    ---------------------------------------------------------------
Pp1s104_175V6.1    MHAC-----------ERRR-----------VV-------------------------------
Pp1s29_32V6.1      MHAF-----------EIAR-----------AL-------------------------------
Pp1s27_359V6.1     M--------------------------------------------------------------
Pp1s29_23V6.1      --MR-----------VRAN-----------VV-------------------------------
Smo36646|PACid_    ---------------------------------------------------------------
MA_3352g0010       ---------------------------------------------------------------
MA_303578g0010     ---------------------------------------------------------------
LOC_Os01g74020     MGEE------------APE-----------EY-------------------------------
At3g46640          MGEE-----------VQMS-----------DY-------------------------------
At5g59570          MGKE-----------VMVS-----------DY--------------G----------------

cons

Cpa_tig00001336    ---------------------------------------------HL----------------
Popu_2051.13       ---------------------------------------------GLSQR-ERGQ----HSEF
Cre02.g083750.t    HSSPQGPAAAAKAAAVAPVGSEAAAGSCGGGKGSGAPAAGAETGPSCPGG-DQGS----EGTA
Cre09.g410450.t    ---------------------------------------------------------------
kf100118_0040_v    ---------------------------------------------GMTAGGQRESIDNDESKV
kf100537_0090_v    -------------------------------------------------M-DKEI----TRRW
Pp1s104_175V6.1    ---------------------------------------------DMTMK-DGDH----VVEW
Pp1s29_32V6.1      ---------------------------------------------GMTMK-EDDH----VVDW
Pp1s27_359V6.1     -----------------------------------------------MTK-DDDQ----VSDW
Pp1s29_23V6.1      ---------------------------------------------GMTMK-DEDY----VLDW
Smo36646|PACid_    ---------------------------------------------------ENDR----VEEW
MA_3352g0010       ------------------------------------------------MK-EEDW----LGKW
MA_303578g0010     -----------------------------------------------MTK-LDDR----VTEW
LOC_Os01g74020     ---------------------------------------------ELGGG-EDER----VMEW
At3g46640          --------------------------------------------D--VSG-DGDR----VSEW
At5g59570          ----------------------------------------DDDGEDAGGG-DEYR----IPEW

cons

Cpa_tig00001336    -ES-----------GAPQSEA---K--------AAPES------TASVMSMP--------QVD
Popu_2051.13       -AA-----------ATADADPPSASEW-----QSSSAQQPVAGRQAAGRGREGDDGDG--GSN
Cre02.g083750.t    ARAAAADGDPLAAPKAAQQQQ-----------QQPRSPAITAGVPGAGTSTASLPASR--AAR
```

```
Cre09.g410450.t     ----------------DPAAG-----------LVPLTSSLIPPLLAHAFALPQL---------
kfl00118_0040_v     -TL-----------GLGSGED-----QDQPGSLTSLEQELLTLGA------QPQDAPQMLPSD
kfl00537_0090_v     -GV-----------GIPSPEE-----------LAPLSTRLITPVLACAFNIEGDSGAS--LTE
Pp1s104_175V6.1     -NP-----------GLPTGEE-----------LNPTSKSLISLVLASGLSMKPEPLKT--AAD
Pp1s29_32V6.1       -NH-----------SLPTGEE-----------VTPLSKSLISPVLASAFSIMPDLAKT--AAH
Pp1s27_359V6.1      -TK-----------GLPTSEE-----------LTPLSHTLISRILASAFRIKHEEPMT--EED
Pp1s29_23V6.1       -KH-----------RQPKGEE-----------LTPSSKSLKSSAMVSCFSIQPVSSKS--TAD
Smo36646|PACid_     -EE-----------GLPTPEE-----------LTPLNQSLITPELASAFSISQEAAKS--SSD
MA_3352g0010        -ED-----------ELPSPEE-----------LMPLTQNLITPDLAAAFKIHPSSASA--SAP
MA_303578g0010      -EA-----------GLPGPDE-----------LTPLSQVLITPELACAFSISPEPCRS--QLD
LOC_Os01g74020      -ET-----------GLPGADE-----------LTPLSQPLVPAGLAAAFRIPPEPGRT--LLD
At3g46640           -EM-----------GLPSDED-----------LASLSYSLIPPNLAMAFSITPERSRT--IQD
At5g59570           -EI-----------GLPNGDD-----------LTPLSQYLVPSILALAFSMIPERSRT--IHD

cons                                                 .


Cpa_tig00001336     IK--KESFAMQ----------------------------------------------------
Popu_2051.13        SR--RPAETGE----------------------------------------------------
Cre02.g083750.t     VH--GVAAGVARPAAVVPTGSGITGTAADVRRPHPYHNHPNHHGRDMTNGDNGNSAHGTNDGS
Cre09.g410450.t     ---------------------------------------------------------------
kfl00118_0040_v     LAGSLAGSSVE----------------------------------------------------
kfl00537_0090_v     E---EVDEAAL----------------------------------------------------
Pp1s104_175V6.1     VS--GESRASF----------------------------------------------------
Pp1s29_32V6.1       VS--RETRPSL----------------------------------------------------
Pp1s27_359V6.1      VR--RESQATI----------------------------------------------------
Pp1s29_23V6.1       LS--RESHASF----------------------------------------------------
Smo36646|PACid_     VL--HASIATV----------------------------------------------------
MA_3352g0010        TE--SGS--------------------------------------------------------
MA_303578g0010      VS--RASKNTM----------------------------------------------------
LOC_Os01g74020      VH--RASAATV----------------------------------------------------
At3g46640           VN--RASETTL----------------------------------------------------
At5g59570           VN--RASQITL----------------------------------------------------

cons


Cpa_tig00001336     ---------------------------------------------------------------
Popu_2051.13        ---------------------------------------------------------------
Cre02.g083750.t     GSGSGNGDSINNNSGGGVANAAAGGPAAPAPASGAAGGTNVPHSHHHHHSHHYHSHHHSTELA
Cre09.g410450.t     ---------------------------------------------------------------
kfl00118_0040_v     ---------------------------------------------------------------
kfl00537_0090_v     ---------------------------------------------------------------
Pp1s104_175V6.1     ---------------------------------------------------------------
Pp1s29_32V6.1       ---------------------------------------------------------------
Pp1s27_359V6.1      ---------------------------------------------------------------
Pp1s29_23V6.1       ---------------------------------------------------------------
Smo36646|PACid_     ---------------------------------------------------------------
MA_3352g0010        ---------------------------------------------------------------
MA_303578g0010      ---------------------------------------------------------------
LOC_Os01g74020      ---------------------------------------------------------------
At3g46640           ---------------------------------------------------------------
At5g59570           ---------------------------------------------------------------

cons


Cpa_tig00001336     ---------------------------------------------------------------
Popu_2051.13        ---------------------------------------------------------------
Cre02.g083750.t     HEGSTSTGAPAPPGLAPLTRGNDRASSAFTAYVRRVPYAATGADAVSAHGAEPAANTPNASGG
Cre09.g410450.t     ---------------------------------------------------------------
kfl00118_0040_v     ---------------------------------------------------------------
kfl00537_0090_v     ---------------------------------------------------------------
Pp1s104_175V6.1     ---------------------------------------------------------------
Pp1s29_32V6.1       ---------------------------------------------------------------
Pp1s27_359V6.1      ---------------------------------------------------------------
Pp1s29_23V6.1       ---------------------------------------------------------------
Smo36646|PACid_     ---------------------------------------------------------------
```

```
MA_3352g0010       ---------------------------------------------------------------
MA_303578g0010     ---------------------------------------------------------------
LOC_Os01g74020     ---------------------------------------------------------------
At3g46640          ---------------------------------------------------------------
At5g59570          ---------------------------------------------------------------

cons

Cpa_tig00001336    ---------------------------------------------------------------
Popu_2051.13       ---------------------------------------------------------------
Cre02.g083750.t    NGNENGSGSAHGSAHGSGHASGHGSGSNGNGAGAGTGSSGNGNSSSGGQPTTTTTTTTNGRHH
Cre09.g410450.t    ---------------------------------------------------------------
kfl00118_0040_v    ---------------------------------------------------------------
kfl00537_0090_v    ---------------------------------------------------------------
Pp1s104_175V6.1    ---------------------------------------------------------------
Pp1s29_32V6.1      ---------------------------------------------------------------
Pp1s27_359V6.1     ---------------------------------------------------------------
Pp1s29_23V6.1      ---------------------------------------------------------------
Smo36646|PACid_    ---------------------------------------------------------------
MA_3352g0010       ---------------------------------------------------------------
MA_303578g0010     ---------------------------------------------------------------
LOC_Os01g74020     ---------------------------------------------------------------
At3g46640          ---------------------------------------------------------------
At5g59570          ---------------------------------------------------------------

cons

Cpa_tig00001336    ---------------------------------------------------------------
Popu_2051.13       ---------------------------------------------------------------
Cre02.g083750.t    SNGGGGNSNGNGSHGQGPTAASALVGSASSRRMGTPHQRCDGGPREHNSLDRNDSGSNGNGNG
Cre09.g410450.t    -----------------------------AQAPAPAQLQPQAT--------------------
kfl00118_0040_v    ---------------------------------------------------------------
kfl00537_0090_v    ---------------------------------------------------------------
Pp1s104_175V6.1    ---------------------------------------------------------------
Pp1s29_32V6.1      ---------------------------------------------------------------
Pp1s27_359V6.1     ---------------------------------------------------------------
Pp1s29_23V6.1      ---------------------------------------------------------------
Smo36646|PACid_    ---------------------------------------------------------------
MA_3352g0010       ---------------------------------------------------------------
MA_303578g0010     ---------------------------------------------------------------
LOC_Os01g74020     ---------------------------------------------------------------
At3g46640          ---------------------------------------------------------------
At5g59570          ---------------------------------------------------------------

cons

Cpa_tig00001336    ---------------------------------------------------------------
Popu_2051.13       ----------------------------------------------------------LDA--
Cre02.g083750.t    NGSSSERGPACGSSKHAGSHGHAAPPPPPTAIGHAGAGPHLASQHQQHRTSHAQLPGGGRLAG
Cre09.g410450.t    ---------------------------------------------------------------
kfl00118_0040_v    ----------------------------------------------------------QAI--
kfl00537_0090_v    ----------------------------------------------------------NTV--
Pp1s104_175V6.1    ----------------------------------------------------------LDS--
Pp1s29_32V6.1      ----------------------------------------------------------LDL--
Pp1s27_359V6.1     ----------------------------------------------------------HNL--
Pp1s29_23V6.1      ----------------------------------------------------------LDL--
Smo36646|PACid_    ----------------------------------------------------------TAL--
MA_3352g0010       ---------------------------------------------------------------
MA_303578g0010     ----------------------------------------------------------SAL--
LOC_Os01g74020     ----------------------------------------------------------SRL--
At3g46640          ----------------------------------------------------------SSL--
At5g59570          ----------------------------------------------------------SSL--

cons
```

```
Cpa_tig00001336   ---------------------------------------------------------------
Popu_2051.13      ---------------------------------------------------------------
Cre02.g083750.t   STCSGGGGGVGAGCGVSNRLGEFVAAAAAAAVGNAGSGGNSGSGSGSGSGSGSGSGSGSGSAS
Cre09.g410450.t   ---------------------------------------------------------------
kfl00118_0040_v   ---------------------------------------------------------------
kfl00537_0090_v   ---------------------------------------------------------------
Pp1s104_175V6.1   ---------------------------------------------------------------
Pp1s29_32V6.1     ---------------------------------------------------------------
Pp1s27_359V6.1    ---------------------------------------------------------------
Pp1s29_23V6.1     ---------------------------------------------------------------
Smo36646|PACid_   ---------------------------------------------------------------
MA_3352g0010      ---------------------------------------------------------------
MA_303578g0010    ---------------------------------------------------------------
LOC_Os01g74020    ---------------------------------------------------------------
At3g46640         ---------------------------------------------------------------
At5g59570         ---------------------------------------------------------------

cons

Cpa_tig00001336   ---------------------------------------------------------------
Popu_2051.13      ---------------RRQEGGEDDD-VVHTKRQRVETAEQAEYAQEHQL--------------
Cre02.g083750.t   PCTLLKTEQEEGAAAGPD--IGL----D------G-GA----------DADAARAEGAADGTD
Cre09.g410450.t   ---------------------------------------------------------------
kfl00118_0040_v   ---------------AAE------------------EA----------V--------------
kfl00537_0090_v   ---------------RGGMERATPS--Q------V-SP-------------------------
Pp1s104_175V6.1   ---------------QVQ--RFSPM--S------F-EA----------L--------------
Pp1s29_32V6.1     ---------------RVQ--SSISM--S------F-DP----------F--------------
Pp1s27_359V6.1    ---------------FKQ--KPIP---S------F-DA----------F--------------
Pp1s29_23V6.1     ---------------QVQ--RSTPM--S------F-NP----------F--------------
Smo36646|PACid_   ---------------RRQ--PSSSPGGV------F-ES----------I--------------
MA_3352g0010      -------------------------------------V----------F--------------
MA_303578g0010    ---------------RRQ--NSA-PVGS------M-KG----------F--------------
LOC_Os01g74020    ---------------RRA--SSS----S------S-SS----------F--------------
At3g46640         ---------------RGG--SSGPN-TS-----SS-NN----------N--------------
At5g59570         ---------------RSS----T-N-AS-----SV-ME----------E--------------

cons

Cpa_tig00001336   ---------------------------------------------------------------
Popu_2051.13      ---------------------------------------------------------------
Cre02.g083750.t   IDMDDADGDGDGDGDGDGDGDGDGDGDADADGDGDGDGDGEADAEADMDDGMIPYDAKPLE
Cre09.g410450.t   ---------------------------------------------------------------
kfl00118_0040_v   ---------------------------------------------------------------
kfl00537_0090_v   ---------------------------------------------------------------
Pp1s104_175V6.1   ---------------------------------------------------------------
Pp1s29_32V6.1     ---------------------------------------------------------------
Pp1s27_359V6.1    ---------------------------------------------------------------
Pp1s29_23V6.1     ---------------------------------------------------------------
Smo36646|PACid_   ---------------------------------------------------------------
MA_3352g0010      ---------------------------------------------------------------
MA_303578g0010    ---------------------------------------------------------------
LOC_Os01g74020    ---------------------------------------------------------------
At3g46640         ---------------------------------------------------------------
At5g59570         ---------------------------------------------------------------

cons

Cpa_tig00001336   ---------------------------------------------------------------
Popu_2051.13      ---------------------------------------------------------------
Cre02.g083750.t   LAMLAAGTTASGGADRGGGGGGGGFGGGAAGAPPASLSNQQAAAAMLELATGGGGSSSAGVGT
Cre09.g410450.t   ---------------------------------------------------------------
kfl00118_0040_v   ---------------------------------------------------------------
```

```
kfl00537_0090_v     ---------------------------------------------------------------
Pp1s104_175V6.1     ---------------------------------------------------------------
Pp1s29_32V6.1       ---------------------------------------------------------------
Pp1s27_359V6.1      ---------------------------------------------------------------
Pp1s29_23V6.1       ---------------------------------------------------------------
Smo36646|PACid_     ---------------------------------------------------------------
MA_3352g0010        ---------------------------------------------------------------
MA_303578g0010      ---------------------------------------------------------------
LOC_Os01g74020      ---------------------------------------------------------------
At3g46640           ---------------------------------------------------------------
At5g59570           ---------------------------------------------------------------

cons


Cpa_tig00001336     ---------------------------------------------------------------
Popu_2051.13        ---------------------------------------------------------------
Cre02.g083750.t     GVGVGSSSGRLGSAGGLSGLLLAVGGGGSGGTGGMMPVQLLQMLTSTPGGLALLTPSQLSELV
Cre09.g410450.t     ---------------------------------------------AQGYYGSMLSPALMGALG
kfl00118_0040_v     ---------------------------------------------------------------
kfl00537_0090_v     ---------------------------------------------------------------
Pp1s104_175V6.1     ---------------------------------------------------------------
Pp1s29_32V6.1       ---------------------------------------------------------------
Pp1s27_359V6.1      ---------------------------------------------------------------
Pp1s29_23V6.1       ---------------------------------------------------------------
Smo36646|PACid_     ---------------------------------------------------------------
MA_3352g0010        ---------------------------------------------------------------
MA_303578g0010      ---------------------------------------------------------------
LOC_Os01g74020      ---------------------------------------------------------------
At3g46640           ---------------------------------------------------------------
At5g59570           ---------------------------------------------------------------

cons


Cpa_tig00001336     -PPA-----------------------------------------------------------
Popu_2051.13        -PQR--LEPSLMPS----------------T--------------------------------
Cre02.g083750.t     VLVASGGSGSGGSGGGPGPQQGPAATAVLASPELQ-LQQLLQQQQQQHQQQQLQLLLQLQQVM
Cre09.g410450.t     YATG--------ASYAPVQQQQPSHLASLQYGYLHALATASQQQQQQQQQQQHSAAALLAHAH
kfl00118_0040_v     -PG------------------------------------------------------------
kfl00537_0090_v     --QE--NPQVGPPL-------------------------------------------------
Pp1s104_175V6.1     -PLF--KERKDAGS-------------------------------------------------
Pp1s29_32V6.1       -SPF--KQRNDAGV-------------------------------------------------
Pp1s27_359V6.1      -PAF--QEHDDAGG-------------------------------------------------
Pp1s29_23V6.1       -PPF--KELKNAGV-------------------------------------------------
Smo36646|PACid_     -PAF--PQSSDLG--------------------------------------------------
MA_3352g0010        -P-------------------------------------------------------------
MA_303578g0010      -PAF--SVEEHEGH-------------------------------------------------
LOC_Os01g74020      -PAF--ASKGAGT--------------------------------------------------
At3g46640           -VEE--EDRVGSSS-------------------------------------------------
At5g59570           -VV----DRVESSV-------------------------------------------------

cons


Cpa_tig00001336     ---------------------------------------------------------------
Popu_2051.13        --------AEAQTEDGLDF-----------L----------VSDENPLPVD------------
Cre02.g083750.t     NGGGPLGPGGLGPGAAAALVGPGSLPGLAGLGGLLGNPLASLGGLGGLTALSGLGSLASGLAG
Cre09.g410450.t     NA-----------------------AALAAVR-------------------------------
kfl00118_0040_v     ---------EG----------------------------------------------------
kfl00537_0090_v     --V-----GFADGR-------------------------------------------------
Pp1s104_175V6.1     --F-----VCASEVRDDGG----------------------G-----RGDG------------
Pp1s29_32V6.1       --Y-----GCLAEAGDDRD----------------------G-----RGVG------------
Pp1s27_359V6.1      --Y-----GYGVEGRSSGE-----------E----------F-----RGNR------------
Pp1s29_23V6.1       --Y-----GCLAEAGDDRD----------------------G-----RGVG------------
Smo36646|PACid_     ---------------------------------------------------------------
MA_3352g0010        ------------------------------------------------GSG------------
MA_303578g0010      --E-----LDGGQTRVNGG-----------------------------GGG------------
```

```
LOC_Os01g74020     ---------------------------------------------------------------
At3g46640          --P-----GSDSKKQK-----------------------------------------------
At5g59570          --P-----GSDPKKQK-----------------------------------------------

cons


Cpa_tig00001336    ---------------------------------------------------------------
Popu_2051.13       ---------------------------------------------PWVHDDPFYSR--NDFYD
Cre02.g083750.t    GGLGGFAGGALGGGGVLGQALGLLGPHPLTA-AAAGGGAGGGAGGSSSGGVLAS-LLGGAGPT
Cre09.g410450.t    ----------------AGQATSSAMSHPLPPAASADTGSARGAGPSASEPERAA-SASVA-TN
kfl00118_0040_v    ---------------------------------------------------------------
kfl00537_0090_v    ---------------------------------------------------------------
Pp1s104_175V6.1    ---------------------------------------------HVGGRGGSI-AT-GEGRS
Pp1s29_32V6.1      ---------------------------------------------QFDKRGKSI-T--ADFTA
Pp1s27_359V6.1     ---------------------------------------------RVEEEFREN-RGDEEFRK
Pp1s29_23V6.1      ---------------------------------------------QFDKRGKSI-T--ADFTA
Smo36646|PACid_    ---------------------------------------------------------------
MA_3352g0010       ---------------------------------------------QQQQR-------------
MA_303578g0010     ---------------------------------------------QAGVSQVA-----HVP--
LOC_Os01g74020     ---------------------------------------------------------------
At3g46640          ---------------------------------------------------------------
At5g59570          ---------------------------------------------------------------

cons


Cpa_tig00001336    --------------------APAALSGAGARKPS----VSGNLS-------------------
Popu_2051.13       T----QYVQLATDSTAAAMPYGAPDDGLYNEYAIDLAPALPDMIPEDHAEGY-----------
Cre02.g083750.t    AG-----------------GGGLGGASSGSATAALAAAVAAQQQQQQQQQQQQQQQQLLMLQQ
Cre09.g410450.t    SS-----------------GSALRGSSASYGSAAAAAAYAHPLMGLYAALSQQQQQ-----AA
kfl00118_0040_v    -------------------AQAGQAAGRGSAGG----QVPVGMGAPDYSHA------------
kfl00537_0090_v    ---------------------------------------------------------------
Pp1s104_175V6.1    D----A-------------RGEVRGETRGAEEPEIEAEVEVGLGLGLGSGG------------
Pp1s29_32V6.1      E----G-------------RGDARGDGRGTAEP----EVEVGLGLGLGSGGS-----------
Pp1s27_359V6.1     EFRGNR-------------RTEERRDGRSLAIE----DRMDYRGSAFGSEIP-----------
Pp1s29_23V6.1      E----G-------------RGDARGDGRGTAEP----EVEVGLGLGLGSGGS-----------
Smo36646|PACid_    ---------------------------------------------------------------
MA_3352g0010       ---------------------------------------------------------------
MA_303578g0010     ---------------------------------------------------------------
LOC_Os01g74020     ---------------------------------------------------------------
At3g46640          ---------------------------------------------------------------
At5g59570          ---------------------------------------------------------------

cons


Cpa_tig00001336    ---------------------------------------------------------------
Popu_2051.13       ------------------DASRHDT--------------------------------------
Cre02.g083750.t    SSQKP-SSSTG--APFPYAQQQAAHTQTPSGGERNSPAILSAPEPPASAQPAPLLPMPQPSGG
Cre09.g410450.t    AYGASPANSYGAMAPFTTHLHPA---------------YYAR---------------PQDAGQ
kfl00118_0040_v    ---------------------------------------------------------------
kfl00537_0090_v    ---------------------------------------------------------------
Pp1s104_175V6.1    -------------------GGRDND--------------------------------------
Pp1s29_32V6.1      ------------------CSNRECD--------------------------------------
Pp1s27_359V6.1     ------------------KQTRASG--------------------------------------
Pp1s29_23V6.1      ------------------CSNRECD--------------------------------------
Smo36646|PACid_    ---------------------------------------------------------------
MA_3352g0010       -------------------PGVEPP--------------------------------------
MA_303578g0010     ---------------------------------------------------------------
LOC_Os01g74020     ---------------------------------------------------------------
At3g46640          ---------------------------------------------------------------
At5g59570          ---------------------------------------------------------------

cons
```

```
Cpa_tig00001336     ---------------------------------------------------------------
Popu_2051.13        ---------------------------------------------------------------
Cre02.g083750.t     AGAAAAAAAGAPTAAGGGGGGGRRSSGSADGSVPVRLAPTPGDLLHGSGLAHQSPTVAVETVI
Cre09.g410450.t     AA-------------------------------------------------------------
kfl00118_0040_v     ---------------------------------------------------------------
kfl00537_0090_v     ---------------------------------------------------------------
Pp1s104_175V6.1     ---------------------------------------------------------------
Pp1s29_32V6.1       ---------------------------------------------------------------
Pp1s27_359V6.1      ---------------------------------------------------------------
Pp1s29_23V6.1       ---------------------------------------------------------------
Smo36646|PACid_     ---------------------------------------------------------------
MA_3352g0010        ---------------------------------------------------------------
MA_303578g0010      ---------------------------------------------------------------
LOC_Os01g74020      ---------------------------------------------------------------
At3g46640           ---------------------------------------------------------------
At5g59570           ---------------------------------------------------------------

cons


Cpa_tig00001336     ---------------------------------------------IT----------------
Popu_2051.13        -----SPAAVQET---TTAEAVSVVAGEASATTVPGHARNPTTHHTD----------------
Cre02.g083750.t     KAEPEADAEAEEEAAAAANAAAA-----------------AAAAAAF----------N-----
Cre09.g410450.t     -----------------AAAAAA-----------------AAAAGAY----------G-----
kfl00118_0040_v     ---------GMQPQEYGAGDYNH-----------------YGAQVKL----------E-----
kfl00537_0090_v     ---------------------------------------------------------------
Pp1s104_175V6.1     -----RATVAEKQ---NCCSHS------------------ASYSHGH----------------
Pp1s29_32V6.1       -----RVTVAERQ---RSGSHGG-----------------GSFSQGH----------------
Pp1s27_359V6.1      -----SDTTTPPKGSMKSNTGAG-----------------GSVGQGLQGSVSITATNTGREHS
Pp1s29_23V6.1       -----RVTVAERQ---RSGSHGG-----------------GSFSQGH----------------
Smo36646|PACid_     --------E------------------------------------------------------
MA_3352g0010        ---------------------------------------------------------------
MA_303578g0010      ---------------------------------------------------------------
LOC_Os01g74020      ---------------------------------------------------------------
At3g46640           ---------------------------------------------------------------
At5g59570           ---------------------------------------------------------------

cons


Cpa_tig00001336     --VP-----------------------------------------------------------
Popu_2051.13        --EDTGEYHARA---ESSADISIWGPTYPSG--------------------------------
Cre02.g083750.t     ---VFADIADAQ---LDEAMGVEDGEETEDGGAGPRQDDEGDEETGTDTDGRQDRRGGSGSPL
Cre09.g410450.t     ---Y------AQ---PTASYGSSFG-HPASS-YGGAPS----------------------SIL
kfl00118_0040_v     ---DNSRRGH-------------------QE--------------------------------
kfl00537_0090_v     ------WDGGGA---DGAEGYPGE--EPT-N--------------------------------
Pp1s104_175V6.1     -------------------HQPSEGRSEHSS--------------------------------
Pp1s29_32V6.1       --PMSGVFRGGS---DGATYNPSEGRSEQSS--------------------------------
Pp1s27_359V6.1      STPV-GNTRPARYGHGA--DTANTHERPIQP--------------------------------
Pp1s29_23V6.1       --PMSGVFRGGS---DGATYNPSEGRSEQSS--------------------------------
Smo36646|PACid_     ---------------------------------------------------------------
MA_3352g0010        ---------------------------------------------------------------
MA_303578g0010      ---------------------------------------------------------------
LOC_Os01g74020      ---------------------------------------------------------------
At3g46640           ---------------------------------------------------------------
At5g59570           ---------------------------------------------------------------

cons


Cpa_tig00001336     -----------------------------------------------------------S---
Popu_2051.13        ----------------------ELGVA--------------------------------DSDG
Cre02.g083750.t     DMVAAAAAAAAEAEAEAEAAAEARVASRMTIITGDGGDTDKICQVSLLDAPPLVRLSAQH-NG
Cre09.g410450.t     DFSSSLAAAYRPVAMPAMPAASALAA-------------------------------------
kfl00118_0040_v     ----------------------MPAD--------------------------------S---G
kfl00537_0090_v     ----------------------GVA-------------------------------------I
Pp1s104_175V6.1     ----------------------GLPF--------------------------------MM-NG
Pp1s29_32V6.1       ----------------------GLPF--------------------------------MK-NG
```

```
Pp1s27_359V6.1      ----------------------SLPY--------------------------------IM-NS
Pp1s29_23V6.1       ----------------------GLPF--------------------------------MK-NG
Smo36646|PACid_     ---------------------------------------------------------------
MA_3352g0010        ---------------------------------------------------------------
MA_303578g0010      ---------------------------------------------------------------
LOC_Os01g74020      ---------------------------------------------------------------
At3g46640           ---------------------------------------------------------------
At5g59570           ---------------------------------------------------------------

cons


Cpa_tig00001336     ---------------------------------------------------------------
Popu_2051.13        GQQARRVYGTNSEG------SSLQGRAGVKSRVKELLNVPRQPRARLEPRPVLDRTSTVWKRE
Cre02.g083750.t     ATAEAAAAAAAAATSSAGGAGGAAGVFMARRL-------------------------------
Cre09.g410450.t     AAAVAAAAGAMPQTSQSVGMTGAV---------------------------------------
kfl00118_0040_v     AAGEAGVGGKRPLE------GAHAGLGLSRQ--------------------------------
kfl00537_0090_v     LQG---VHGNMPAY------GAFSGGVENSQD-------------------------------
Pp1s104_175V6.1     LPAYGSPYERRPDS------SSYGGRGDDSSK-------------------------------
Pp1s29_32V6.1       LQGYGSGYERRPES------SSYGGRGDDSSK-------------------------------
Pp1s27_359V6.1      LPY--GPYERVPES------SYGGPREDESSR-------------------------------
Pp1s29_23V6.1       LQGYGSGYERRPES------SSYGGRGDDSSK-------------------------------
Smo36646|PACid_     ---------------------------------------------------------------
MA_3352g0010        ---------------------------------------------------------------
MA_303578g0010      -------------------------FGGESSD-------------------------------
LOC_Os01g74020      ---------------------------------------------------------------
At3g46640           ------------------------TSNGDGDD-------------------------------
At5g59570           ------------------------KSD------------------------------------

cons


Cpa_tig00001336     -----------------------------------------------G---------------
Popu_2051.13        VTKANN----------------------------------QSISTGVA---------------
Cre02.g083750.t     -GGAAAAMAVTAAVAAEVTAAAGSGSAGGGATASGGGGSVIGVKRKASALGLARTASGLLSGG
Cre09.g410450.t     ---------------------AAGGGANGAVSSLGGG-AA-----------------------
kfl00118_0040_v     --STEE----------------------------------SLGR---H---------------
kfl00537_0090_v     -GGGKK----------------------------------ARARPAES---------------
Pp1s104_175V6.1     -GGGCA----------------------------------KNARKLAD---------------
Pp1s29_32V6.1       -GGGSA----------------------------------KNARKLAD---------------
Pp1s27_359V6.1      -GGGST----------------------------------DFGRKMAD---------------
Pp1s29_23V6.1       -GGGSA----------------------------------KNARKLAD---------------
Smo36646|PACid_     ---------------------------------------------------------------
MA_3352g0010        --------------------------------------------KRIE---------------
MA_303578g0010      -GGGF-----------------------------------KKGRNRET---------------
LOC_Os01g74020      ---------------------------------------------------------------
At3g46640           -GGGV--------------------------------------DPDSA---------------
At5g59570           -GGEA--------------------------------------AAVED---------------

cons


Cpa_tig00001336     ---------------------QTSAALPSPSTPAAP-A--TPSASSASLQPKRRLVWTPELHQ
Popu_2051.13        ---------------------VGT--GHSN-QAGTSSR--AGTDDGTDSDMRTRLVWTPELHD
Cre02.g083750.t     AGGGGGGGAVSSPAAAGTAGASS-G-EEPT-GGGGSVAGGDGADLSCKAPKKPRINWSQELHA
Cre09.g410450.t     ------------------------G-MMG-------EE--YADDGTTRAVKRPRLVWTPQLHR
kfl00118_0040_v     ---------------------ME-G-GREG-EDGGTEG--EEQEGRGQGNKKQRLVWTAELHS
kfl00537_0090_v     ------------------------M-EGED-ELEGMEH--TSDDPPGRALKRPRLVWTPQLHK
Pp1s104_175V6.1     ---------------------SD-F-EDTD-SGGGPVN--SNEETNARTLKRPRLVWTPQLHK
Pp1s29_32V6.1       ---------------------SD-F-EDTD-SGGGPVN--SNEEANARTLKRPRLVWTPQLHK
Pp1s27_359V6.1      ---------------------FE-L-EDAN-SAGGLMN--SNDEP----LKRARLVWTPQLHK
Pp1s29_23V6.1       ---------------------SD-F-EDTD-SGGGPVN--SNEEANARTLKRPRLVWTPQLHK
Smo36646|PACid_     ------------------------E-EEAD-SGGGPEN--SGEEPAARTLKRPRLVWTPQLHK
MA_3352g0010        ---------------------LE-E-EESS--VGGVTE--NVGEEPARTLKRPRLVWTPQLHK
MA_303578g0010      ------------------------E-EPES-SASGFEN--SSDEHSARTLKRPRLVWTPQLHR
LOC_Os01g74020      ----------------------G-A-DEAE-SGGGADG--GNGNTNNSSSKRARLVWTPQLHK
At3g46640           ---------------------MA-A-EEGD-------S--GTEDLSGKTLKRPRLVWTPQLHK
At5g59570           ---------------------ST-A-EEGD-------S--GPEDASGKTSKRPRLVWTPQLHK
```

```
cons                                                                 : *: *: :**


Cpa_tig00001336    RFVAAVNILGVHCAAPKAILQLMNIDGLTAEHVKSHLQKYRMNLK--KN-GGQDTLFDGAE--
Popu_2051.13       RFVQAVRVLGLGNAMPRTILQVMNVDGLTTEHIKSHLQKYRQSLK-KQT-ASAKE--EAAS--
Cre02.g083750.t    RFLNAMFQLGIKNAVPKTILQLMNVEGLTRENVASHLQKYRILLK-R-H-ANL--PANAPL--
Cre09.g410450.t    KFESAVIKLGEDKAVPKTIMQEMNIDGLTRENVASHLQKYRMIKR-R-DVTGT--SSDGGR--
kfl00118_0040_v    RFMNAVNHLGVKNAVPKTILQLMNVEGMTRENVASHLQKYRLYLK-R-L-AGL--PPNARLSP
kfl00537_0090_v    RFCDAVNHLGVENAVPKTIMQLMNVEGLTRENVASHLQKYRLYLRQR-F-QTD--APAVPS--
Pp1s104_175V6.1    RFVDAVGHLGIKNAVPKTIMQLMNVEGLTRENVASHLQKYRLYLK-R-M-QGL--SSDGPP--
Pp1s29_32V6.1      RFVDAVGHLGIKNAVPKTIMQLMNVEGLTRENVASHLQKYRLYLK-R-M-QGL--PSDGPM--
Pp1s27_359V6.1     RFVEAVGHLGIKNAVPKTIMQLMNVEGLTRENVASHLQKYRLYLK-R-M-QGL--SNDGPS--
Pp1s29_23V6.1      RFVDAVGHLGIKNAVPKTIMQLMNVEGLTRENVASHLQKYRLYLK-R-M-QGL--PSDGPM--
Smo36646|PACid_    RFVDAVAHLGIKNAVPKTIMQLMNVEGLTRENVASHLQKYRLYLK-R-M-QGL--SSEGPS--
MA_3352g0010       RFVDAVAHLGIKNAVPKTIMQLMNVEGLTRENVASHLQKYRLYLK-R-M-QGL--SSEGPS--
MA_303578g0010     RFVDVVSHLGIKNAVPKTIMQLMNVEGLTRENVASHLQKYRLYLK-R-M-QGL--SNEGPS--
LOC_Os01g74020     RFVEVVAHLGMKNAVPKTIMQLMNVEGLTRENVASHLQKYRLYVK-R-M-QGL--SNEGPS--
At3g46640          RFVDVVAHLGIKNAVPKTIMQLMNVEGLTRENVASHLQKYRLYLK-R-M-QGL--TNEGP---
At5g59570          RFVDVVAHLGIKNAVPKTIMQLMNVEGLTRENVASHLQKYRLYLK-R-I-QGL--TTEEDPY-

cons               :*  .:  **   * *::*:* **::*:* *:: *******   :


Cpa_tig00001336    FSS-GADTKP---SAALSEASSSP--------------------------------A------
Popu_2051.13       -QS---RSMKMVMRAV----FDPE--------------------------------LPS----
Cre02.g083750.t    -NP---DNLR----------KLEVVQQAVQQSMQQSLLPPGATNAGGAAGGLISVAVGGGLGG
Cre09.g410450.t    -DS---GTTA----------APTSA-------------GTA----------------------
kfl00118_0040_v    ELVQGGSHLY----------PYVH--------------------------------QPH--EV
kfl00537_0090_v    -SS---DAVF----------ANGP--------------------------------QPP----
Pp1s104_175V6.1    -AN---DQLF----------SSTP--------------------------------LPP----
Pp1s29_32V6.1      -AN---DQLF----------ASTS--------------------------------LPS----
Pp1s27_359V6.1     -AS---DHLF----------ASMP--------------------------------LPP----
Pp1s29_23V6.1      -AN---DQLF----------ASTS--------------------------------LPS----
Smo36646|PACid_    -AS---DHLF----------ASTP--------------------------------VP-----
MA_3352g0010       -SC---DQLF----------ASTP--------------------------------VPP----
MA_303578g0010     -LS---DHLF----------ASTP--------------------------------VPY----
LOC_Os01g74020     -PS---DHIF----------ASTP--------------------------------VPH----
At3g46640          SAS---DKLF----------SSTP--------------------------------VPP----
At5g59570          SSS---DQLF----------SSTP--------------------------------VPP----

cons


Cpa_tig00001336    PKEAAA---SS----------------------SA---------V----ASS-SAAVSSSTAA
Popu_2051.13       GSAEPFSPETA-----P---G-----T-L----LED-QS-----I----AGG-PA------RM
Cre02.g083750.t    TLSY-----QD-----L---------I-N----QLS-AM----SGH-HSAVS-AP-P-----P
Cre09.g410450.t    -AAA-----QR-----QAQQQQRPSD-------GAT--A----AD--GTAGC-----------
kfl00118_0040_v    PPPM--MMAPPL--HQG-------YPP-HGAPYYPPPHR----TDTGGPPLM-GS-P-----A
kfl00537_0090_v    RGGG-----S---------------------------A---GP----------AL-S-----G
Pp1s104_175V6.1    NLGL-----H-----YM---------A-N----QRE-DV--GGPSF-PPATVPPM-P-----Y
Pp1s29_32V6.1      NLGM-----Q-----YM---------T-N----QRE-EV--GPASY-PAVGV-AM-P-----F
Pp1s27_359V6.1     GIMA-----PGMNPHFM---GS--S-S-S----QRD-D---GGSAF-ATPIM-PM-P-----F
Pp1s29_23V6.1      NLGM-----Q-----YM---------T-N----QRE-EV--GPASY-PAVGV-AM-P-----F
Smo36646|PACid_    ---------------------------------------------------------------
MA_3352g0010       SHFL-----H------------------------RD-DM--VV-PL-VPMGM-GV-P-----G
MA_303578g0010     NHAA-----S-----AF---------PGN----HGD-EHMQMPMPY-GSPLV-RM-P-----V
LOC_Os01g74020     ASLH-------------------------------D-Q-------------V-PS-P-----Y
At3g46640          QSFQ-----DI-----G-------G-G-G----GSS-GN----------VGV-PI-P-----G
At5g59570          QSFQ-----DG-----------------G----GSN-GK----------LGV-PV-P-----V

cons


Cpa_tig00001336    S-----SSASVEKSPAE--IAR-APATRSGRSA-SPD-GSAT---------------------
Popu_2051.13       T-----SSG-KPPAKAE--VAP-AASESAQAHTHDRTAGGSAQ--------------------
Cre02.g083750.t    ASAAAISGPPAVAAPRA-V-AV-GP-----P------GPSAPSPAVAAALQAAAGASAGP---
```

```
Cre09.g410450.t   SPAAVSSPAVAAAAPPS-T-AA-AA-----A------TPSAPHSA-H----------------
kfl00118_0040_v   P------------A-SG-D-ASAP------G------PK------PGEGRSGEAGAPPYPAPM
kfl00537_0090_v   -------------G-GE-G-DL-----------------------------------------
Pp1s104_175V6.1   K------------GVGG-G-PL----------------------------------G------
Pp1s29_32V6.1     P------------GGGV-G-PL----------------------------------G------
Pp1s27_359V6.1    Q------------GLVRPG-PL-----------------------------------------
Pp1s29_23V6.1     P------------GGGV-G-PL----------------------------------G------
Smo36646|PACid_   ---------------------------------------------------------------
MA_3352g0010      L------------GHGH-M-VP----------------------------------G------
MA_303578g0010    V------------GMAH-GHAH-G------H------GHM-----TGHGHGHMTAAGM-----
LOC_Os01g74020    ---------------------------------------------------------------
At3g46640         A------------Y-G----TQ--------Q--------------------------------
At5g59570         ---------------------P--------S--------------------------------

cons

Cpa_tig00001336   -----RDGSD------------------------------ASSSARRTTASQSQEL--QRHLK
Popu_2051.13      GA--SFGGSE----------------------------------SARGRLSIKEQI--ERQLE
Cre02.g083750.t   AA--AVGAVP---------------------QA--------AAL----QGLH-RVA--AAAAA
Cre09.g410450.t   -----------------------------------------------------KPS--THGQG
kfl00118_0040_v   GS--IPPSPPHFGYTPEMYSSFSSMAHGGWHPLPGPGPGQPQPG----QYRT-ADPLP-GSPA
kfl00537_0090_v   -----GRERG-------------------------L----RMDT----QSNG-HSI-G-LTDG
Pp1s104_175V6.1   PA--HFSVYD------------------------------RMPY----GVVN-RGF-L-QRPP
Pp1s29_32V6.1     TA--QFGGYE------------------------------QMSY----DGVN-RRF-V-QRPA
Pp1s27_359V6.1    ------GGYE------------------------------YMPY----GPMG-GGF-M-QRAP
Pp1s29_23V6.1     TA--QFGGYE------------------------------QMSY----DGVN-RRF-V-QRPA
Smo36646|PACid_   ---------------------------------------------------------------
MA_3352g0010      PP--HYGGFD------------------------------PRYL----TTLS-RQQ-Q-QP-P
MA_303578g0010    NPSTSYAVFD------------------------------PHAY----GPAYARSI-P-QRIP
LOC_Os01g74020    ---------------------------------------------------------------
At3g46640         MM--QMPVYA-------------------------H----HMGM----QGYH-HQN-HNHDPY
At5g59570         MV--PIPGYG-------------------------N----QMGM----QGYY-QQY-------

cons

Cpa_tig00001336   MQERTLQLQMQ-----------------------------------LQMQMHSQLALQRQLQV
Popu_2051.13      MQERTMQLQLE-----------------------------------MQMIAHRTVSLQRKLQF
Cre02.g083750.t   SAG-----ASASSAASLLGITSGGGGGAPSATGTSGGSPAVSLPLATLPLPALAGGLNGLGGL
Cre09.g410450.t   SSG-----SGGSG-------------------------------------------CGGSGSG
kfl00118_0040_v   PPG-----QGA-----------------------------------QYSSPGGGHYSGPVGGS
kfl00537_0090_v   AP------PQQ-----------------------------------DVG--------Y-----
Pp1s104_175V6.1   MKD-----RTE-----------------------------------HIS--------E-----
Pp1s29_32V6.1     TKD-----RTE-----------------------------------HIS--------E-----
Pp1s27_359V6.1    MPD-----PRE-----------------------------------AAT--------E-----
Pp1s29_23V6.1     TKD-----RTE-----------------------------------HIS--------E-----
Smo36646|PACid_   ---------------------------------------------------------------
MA_3352g0010      PPQ-----RLA-----------------------------------PSA--------A-----
MA_303578g0010    SGD-----HHD-----------------------------------SVA--------D-----
LOC_Os01g74020    --H-----PHP-----------------------------------HHH--------S-----
At3g46640         HQN-----HRH-----------------------------------HHG--------A-----
At5g59570         -SN-----H------------------------------------------------------

cons

Cpa_tig00001336   SISQQKELLQKQMREQQEQLRLLM-QD----------------------------------Q-
Popu_2051.13      TLERQSEVLQACERNP-----------------------------------------------
Cre02.g083750.t   TIGGGAGGGAGGLSAVIGGLAG----GLAGPLAGLTGPNGLNGPLAAAAAGLGGLGGLPAGL-
Cre09.g410450.t   S-----GGHGSGSSA------------RAGSKRSEPEPPSRPTPQRAVAVTEAA---------
kfl00118_0040_v   MYNAGAGAVAGVGSPAPLQGSAYGYQG----------------------------------A-
kfl00537_0090_v   --------------------------R----------------------------------DY
Pp1s104_175V6.1   --------------------------S----------------------------------E-
Pp1s29_32V6.1     --------------------------S----------------------------------E-
Pp1s27_359V6.1    --------------------------N----------------------------------E-
Pp1s29_23V6.1     --------------------------S----------------------------------E-
Smo36646|PACid_   ---------------------------------------------------------------
```

```
MA_3352g0010     --------------------------T----------------------------------P-
MA_303578g0010   ---------------------------------------------------------------
LOC_Os01g74020   --------------------------Y----------------------------------N-
At3g46640        --------------------------G----------------------------------G-
At5g59570        -------------------------------------------------------------G-

cons


Cpa_tig00001336  --------------EAAEKN---SELS---SPERAERR---G---------------------
Popu_2051.13     ---------------GASEV---VKEH---SRLLAEQRQMQE---------------------
Cre02.g083750.t  --------------S---GALAAAAVS---G---L-PGGLGQAGGALGGLGGLNLNVGALGGL
Cre09.g410450.t  --------------------L------------------------------------------
kf100118_0040_v  --------------A---GG---H-YG---S---P-GMGSPG---------------------
kf100537_0090_v  GGYPASMQLSGRPAP---EP--------------LEMDAFGR---------------------
Pp1s104_175V6.1  --------------N---GN---HQS----V---LSQNHHSY---------------------
Pp1s29_32V6.1    --------------N---GN---HAS----P---QSQNAHSY---------------------
Pp1s27_359V6.1   --------------S---RS---HASNNRSN---KSQSQSQS---------------------
Pp1s29_23V6.1    --------------N---GN---HAS----P---QSQNAHSY---------------------
Smo36646|PACid_  ---------------------------------------------------------------
MA_3352g0010     --------------R---GE---YVV----V---EDLSST-----------------------
MA_303578g0010   --------------R---M--------------------------------------------
LOC_Os01g74020   --------------N---AA---YAAT-------VSSYHH-----------------------
At3g46640        --------------NGAFES---NPYM---M---Q-QNKFGS---------------------
At5g59570        -----------------NES---NQYM---M---Q-QNKFGT---------------------

cons


Cpa_tig00001336  -----------------------QAERLKELHMVLAKRIAEQQQEVAKQEDQLKA------LL
Popu_2051.13     -----------------------DLRRQQV------------------LLKSEME------EQ
Cre02.g083750.t  NLGSALGMVGPGVLASALVA---AGAAG------------GGGGSA-----GM-TGATGSGGG
Cre09.g410450.t  -------------------A---SSAHP------------AGSSGS-----GR-NSAGGSAAA
kf100118_0040_v  ------GTGSPPSAQRGSAPAMQQYAAP------------TWQQDPPGGGFGQ------QFWM
kf100537_0090_v  -----------------------PLKRQ------------KFVDQ-------R----------
Pp1s104_175V6.1  -----------------------MEKRA------------NSPEK------RL----------
Pp1s29_32V6.1    -----------------------VENRA------------GSPPR------RL----------
Pp1s27_359V6.1   -----------------------HQSLP------------SSSYQ------HA----------
Pp1s29_23V6.1    -----------------------VENRA------------GSPPR------RL----------
Smo36646|PACid_  ---------------------------------------------------------------
MA_3352g0010     ----------------------------------------VTPPR------QV----------
MA_303578g0010   ----------------------------------------EM----------T----------
LOC_Os01g74020   --------------------------------------------Y------------------
At3g46640        -----------------------M---------------------------------------
At5g59570        -----------------------M---------------------------------------

cons


Cpa_tig00001336  EISEKNVTQLAKPDVTILIARQY
Popu_2051.13     ---ERLRRELLHESTMEDSSNAE
Cre02.g083750.t  AREAPGGGGAATPPPAAGGTPGA
Cre09.g410450.t  ATAAAAGNGVAVM---------A
kf100118_0040_v  QPAAQDWQQKRWPDAGQPQQRKW
kf100537_0090_v  --------AL---AA---HGQLQ
Pp1s104_175V6.1  --------LSLFPTS---S---R
Pp1s29_32V6.1    --------LSLFPTS---S---G
Pp1s27_359V6.1   --------LPLFPT--------N
Pp1s29_23V6.1    --------LSLFPTS---S---G
Smo36646|PACid_  -----------------------
MA_3352g0010     --------LTLFPTG---D---N
MA_303578g0010   --------SS-YHNA---AGPQE
LOC_Os01g74020   ----------HHAN--------H
At3g46640        ---------ASYPSVGGGSANEN
At5g59570        ---------VTYPSVGGGDVNDK

cons
```

```
T-COFFEE, Version_11.00.d625267 (2016-01-11 15:25:41 - Revision d625267 - Build 507)
Cedric Notredame
CPU TIME:0 sec.
SCORE=358
*
 BAD AVG GOOD
*
kf100240_0090_v    :  24
Pp1s87_90V6.1      :  37
Pp1s86_214V6.1     :  38
Pp1s11_285V6.1     :  37
Smo415241|PACid    :  33
LOC_Os06g05060     :  35
LOC_Os01g38530     :  37
At2g25930          :  35
At3g21320          :  34
cons               :  35
```

ELF3

```
kf100240_0090_v    ------------------MA--KQGES--AGKRGPVE--AAALFPRLHVSHAKRDNGPRPPPR
Pp1s87_90V6.1      MPDADDEDLQTKSYRNIKMKGTKDEKA--DGS--KGA--GTALFPRLHVAETKKA-GPRGPPR
Pp1s86_214V6.1     ------------------MKREKGERS--DGG--KQQEPEIALFPRLHVAETKKA-GPRGPPR
Pp1s11_285V6.1     ------------------MKGAKEGKA--DGI--KGA--GPALFPRLHVAETKKA-GPRGPPR
Smo415241|PACid    ------------------ME--D--KN-------APP--GGALFPRLHVKETKNA-GPRAPPR
LOC_Os06g05060     --------------MA--TRGGGGGGGGKEAK--GKV--MGPLFPRLHVNDAAKGGGPRAPPR
LOC_Os01g38530     ------------------MRGGGGGGKEVEER--GKV--MGPLFPRLHVNDAAKGGGPRAPPR
At2g25930          ------------------MKRG----K--D-E--EKI--LEPMFPRLHVNDADKG-GPRAPPR
At3g21320          ------------------MGGMK---D--EAK--RIT--IPPLFPRVHVNDTGRG-GL-----

cons                                                        .:***:** .: .  *

kf100240_0090_v    NKMALYEQVTLQASHPKAAGASGASAPTSQDEGPGGSGMQAPKARTQPDGNLP--SMS-----
Pp1s87_90V6.1      NKMALYEQLAVPS-RYKQSPMLLPSSGRMVTT---------PSMTTSIFQPQA--QAS-----
Pp1s86_214V6.1     NKMALYEQLTVPSHKFKPSPMPLPPNGRTIQS---------PSMSTPIYLPQV--WFTNTHEL
Pp1s11_285V6.1     NKMALYEQLTIPSHRYKMSPLPLPSSGRVMST---------PSMTTPLFNPQV--QAS-----
Smo415241|PACid    NKMALYEQLTIPSHRFQQQQSDGAPPQPF-------------VPQVCKFE--P--MIS-----
LOC_Os06g05060     NKMALYEQFTVPSHRFSGGGGGGGV-GGSP-----------AHSTSAASQSQSQSQVY-----
LOC_Os01g38530     NKMALYEQFTVPSHRFSGGGGALASARGSL-----------ARSTSAASQSQ----VY-----
At2g25930          NKMALYEQLSIPSQRFGDHGTMNSR----------------SNNTSTLVHPGP----------
At3g21320          ------------SQQFDGKTMSLVSSKRPNLP----------SPTNNISDS-----LS-----

cons                           : :

kf100240_0090_v    ---------------------------------------------------------------
Pp1s87_90V6.1      ---------------------------------------------------------------
Pp1s86_214V6.1     SQLSDFLRSFRYMSWMLHENSVCSWRSAVCYLLPKYTCIYQSLLVMKISGSRFSFMSCRLLFT
Pp1s11_285V6.1     ---------------------------------------------------------------
Smo415241|PACid    ---------------------------------------------------------------
LOC_Os06g05060     ---------------------------------------------------------------
LOC_Os01g38530     ---------------------------------------------------------------
At2g25930          ---------------------------------------------------------------
At3g21320          ---------------------------------------------------------------

cons

kf100240_0090_v    ----------------------------------------------SYGSRYDYPQHSMPHPP
Pp1s87_90V6.1      ----------------------------------------------SYDSRYSYMPMSYMSPT
Pp1s86_214V6.1     IVRSWRVLMIYRAMWRYVSEKSHSHLSLHMRSFLTLPIIELQMPVGSYESMYSYMPFSYMKPA
Pp1s11_285V6.1     ----------------------------------------------SYDSRLPYMPMGYMGST
Smo415241|PACid    ----------------------------------------------PALNRGYYFPY-YMVPS
LOC_Os06g05060     ----------------------------------------------GRDSSL-FQPFNVPSN-
LOC_Os01g38530     ----------------------------------------------GCDMPL-FEPFNVPSN-
At2g25930          ---------------------------------------------------S-SQPCGVERN-
At3g21320          ----------------------------------------------TF-------SLSLPPPP
```

```
cons

kfl00240_0090_v   HLPQTSFS----S--APS-------------YY---PQNTFQSRSMTGSQGTALSLRLPSGA-
Pp1s87_90V6.1     VIPTMAYM----PIMNVGLYGVGVG----DGAAPTSSRNASSTR-STVVDGTTESQPAPWGG-
Pp1s86_214V6.1    VVPVMTCM----PVMNSGQCGV---------EAKSSKITSSNSD-STVMDGASESPSL---G-
Pp1s11_285V6.1    IIPTMAFM----PIMNMGLYGVGAGADAGTGASASSSMNASSSK-STVVDGTTESQRVPWGG-
Smo415241|PACid   PV---AFQ---------------------------------------PMN-------VVLGQ-
LOC_Os06g05060    -RPGHSTEKINSDKINKKI-------------------SGSRKELGMLSSQ-------TKGMD
LOC_Os01g38530    -GPGQSVEKMNSNSVNRQI-------------------NGSRKDSGMLSTQ-------PKGID
At2g25930         -----L--------------------------------SVQHLD-SSAANQATEK---FVSQ-
At3g21320         ---------------NNAR--------------------------------------------

cons

kfl00240_0090_v   --S-SVGSASEWDPSEAQALP---AASH-LSQIVPGPP--LGRQQSEG--PSRPRQGEEEGCI
Pp1s87_90V6.1     --E-TLSRGNISSGYSKQSLP---SRKGKEDDDFAVPTYSAT-QA--R--SQTRP-SIN----
Pp1s86_214V6.1    --R-AVGRKSESLGHCAPTNK---ES---LGDDFAVPTYSSASQS--S--SQTVL-TTV----
Pp1s11_285V6.1    --E-APSRANKSSDHAKHLAP---SKKGKEDDDFAVPTYSSATQG--S--SQTRP-SIN----
Smo415241|PACid   --H-SQSVANETEQRELQQVP---GDGGSNRQLARIPSL-HSRAGSGK--TKTRAPRVDDVAA
LOC_Os06g05060    IYASRSTAE-APQRRAENTIKSSSGKRLADDDEFMVPSVFNSRFP--QYSTQENA-GV-QDQS
LOC_Os01g38530    KYGSGSRAECAPQQRVEKGIKSSSGRKLADDDEFIVPSVFSARFP--QYSTKERA-GV-QEES
At2g25930         --M-SFMENVRS---SAQHDQ---RKMVREEEDFAVPVYINSRRS--QSHGRTKS-GIEKEKH
At3g21320         ----------------------------------------------------LID-GPEKNQF

cons

kfl00240_0090_v   ----VPSFQTQAHLVPIPQARANPGTSAAHDASGRWAWWSWQQQQASF-RAQRPQEGLSEFEE
Pp1s87_90V6.1     ----PPQVPQ------TLRGGGGDVRKGKE-----------------L-VTDKRSP-------
Pp1s86_214V6.1    ----PPNRQG------SR--QQRAFVRGKE-----------------R-SSPCMNQVRR----
Pp1s11_285V6.1    ----PPQVQQ------SF-GGGRSVQKGKG-----------------L-VTEKQSP-------
Smo415241|PACid   ----VPTYPSKPA-AV------------------P-----ARSSK-QE-HFASASVAP-----
LOC_Os06g05060    TPLVAANPHK------SP-S-TVSKSSTKC-----------------Y-NTVSKK--------
LOC_Os01g38530    TPLVALSPHK------SP-P-AVSKSPTKC-----------------Y-NTVSKN--------
At2g25930         TPMVAPSSH------------HS-IRFQEV-----------------NQTGSKQNV-------
At3g21320         SPIYNTKFE------------GKLNKKGIN------------------YTSPKGSS-------

cons                                                           .

kfl00240_0090_v   QSRQRRVGPVSLRAPAYSGPKPAESA-YAPTE-QDRKMP------------EAVWKGAAGEEG
Pp1s87_90V6.1     ---------------A-CSTQLRRTR-------KERSME-D----------DASWTSASV-EC
Pp1s86_214V6.1    ---------------L-LTEKSTEVV-------SSVRKD-D----------G-RVPTASG-EC
Pp1s11_285V6.1    ---------------A-FSTQLRRIS-------KEKSME-D----------DASWTSASD-DC
Smo415241|PACid   -----------------------------------------------------------SN--
LOC_Os06g05060    ------------------LERIHVSDVKSRTPLKDKEME------------AAQT-SKNV-EV
LOC_Os01g38530    ------------------LERINVSDVKSRGSQKDKETG------------PAQT-LKNV-EV
At2g25930         -----------------CLATCSKPE-------VRDQVKANARSGGFVISLDVSVT-EEI-DL
At3g21320         ----------------VTNTKPSSIK-------QNEYLK-N----------LTSLDSIKS-PI

cons

kfl00240_0090_v   LRAQRLALQK-Q--------AAED-------------WRMAAEAS-GRQLEVAAQQTEASQHQ
Pp1s87_90V6.1     LT-------A-S--------AIDN-------------DVNIQDIS-NRKVE------------
Pp1s86_214V6.1    AV-------V-S--------VVDN-------------DVGSEEVN-PSELD------------
Pp1s11_285V6.1    LT-------V-S--------FIDN-------------DLNTQEIS-NRKLD------------
Smo415241|PACid   ------ASTSAT--------KSQR-------------QVEVRETT-QARKDS-----------
LOC_Os06g05060    EK----SSS--FHASKDMFESRHAKVYPKMDKTGIINDSDEPHGG-NSGHQ------------
LOC_Os01g38530    EH----FSS--FEASKDMFGSKHAKVCPK---TGTINDLDEPHLE-NSEHQ------------
At2g25930         EKS--------A--------SSHD-------------RVNDYNAS-LRQES------------
At3g21320         VIH--------S--------EIDP-------------QANT-DLSLQF-CT------------
```

```
cons                                  .                   .                   

kfl00240_0090_v  AQLDCKGVAEEVLFAAERRAGAVAAQQEVA-AQIKAAMGSHSE-GEKQGLVPVLDAGGSEGLP
Pp1s87_90V6.1    -------------FGPREREGGQTKLYPVG-IGLDIAKGQKAD-GE-----------------
Pp1s86_214V6.1   -------------GAPVACDGGFVQPSTHE-IGQEELLLQQRDEAR-----------------
Pp1s11_285V6.1   -------------GVSRDCDGHQTELGRNA-VGKKKSKGNKVD-GG-----------------
Smo415241|PACid  -----------RPRNRA---P------------------------------------------
LOC_Os06g05060   -------------ATS--RNGGSMKF-------------------------------------
LOC_Os01g38530   -------------ATS--RNGSSVKF-------------------------------------
At2g25930        -------------RNRLYRDGGKTRL-------------------------------------
At3g21320        -------------SGS-S------KPGGEAVVGSKILLSERLE-DE-----------------

cons                                                                          

kfl00240_0090_v  KRPPGKPTLNIEVPELSTQAELDGAVAGEPEGSITQEVASGLSEPAVARAAPSVN---MSSGG
Pp1s87_90V6.1    --------------QSCA-NLA-------------------------------KP---SDARA
Pp1s86_214V6.1  --------------EKVV-RPI-------------------------------NA---SETFA
Pp1s11_285V6.1   --------------QIDE-DLT-------------------------------KP---SDTSA
Smo415241|PACid  --------------------------------------------------GDLGD---QENGS
LOC_Os06g05060   -----------------------------------------------------QNPPMRRNEI
LOC_Os01g38530   -----------------------------------------------------QNPPVRRNTI
At2g25930        -----------------------------------------------------KD---TDNGA
At3g21320        ----------------------------------------------------------NQNGS

cons                                                                          

kfl00240_0090_v  G-----AAKQRPPKPRSEGTYGFVRHFQSCVQPVSLKGNGDSSPEVIEGSEGGGAAGFKDAGA
Pp1s87_90V6.1    A-----AALPDSVHE------------------------------------DGGLRVVPE-Q-
Pp1s86_214V6.1   A-----IVPPVSVHR------------------------------------ANSLTGASE-R-
Pp1s11_285V6.1   V-----VIPPEILLE------------------------------------DESLRVVPE-Q-
Smo415241|PACid  E-----VRVMASSSP------------------------------------SHSYESNED-N-
LOC_Os06g05060   SSNPSS--ENTDRHY------------------------------------NLPQGGIE----
LOC_Os01g38530   SAKPSPGIENTNGHC------------------------------------NLPQGGLK----
At2g25930        E-----SHLATENHS------------------------------------QEGH-GSP----
At3g21320        P-----NVMKTQSYR------------------------------------RNFA-E------

cons                                                                          

kfl00240_0090_v  SQRAGFQVGKRAEGTEGPGQGHAKGRKRASAQANRATQEAAAAVAAKAGPSPSGSSSGDRSGG
Pp1s87_90V6.1    ---DIFS----SQSSE--------NRSGEASE-------------------------------
Pp1s86_214V6.1   ---NDFS----NQTSE--------NQRGEGSK-------------------------------
Pp1s11_285V6.1   ---DIFS----SQSTE--------NRSREVSE-------------------------------
Smo415241|PACid  ---GRI----DNQSDE--------ELEGRC---------------------------------
LOC_Os06g05060   ---E-TG----TKRKR--------LLEQHDAE-----------------------KSDDVSRL
LOC_Os01g38530   ---E-AG----TKRKR--------LEAQDNAE-----------------------K-------
At2g25930        ---EDID----NDREY--------SKSRACAS-------------------------------
At3g21320        -----FN----NETQK---------KPKTL---------------------------------

cons                         .                                                

kfl00240_0090_v  AGDGDPPSVCD-VESNGADSAAAAAGILPKDVVSVIGQHGFWKSRKAMQQQQKKFSAQLFEMH
Pp1s87_90V6.1    FSQRKRGGSAE-TESSML-ENLPPGSICFRDVVDAFGQQEFWKSQKTIMRQQKLFSRQVFELH
Pp1s86_214V6.1   PSRLRRDSSME-TGSSML-ENVVMGSIEFRDVVDAFGQQEFWKVQMMIIRQQKLFALQVFELH
Pp1s11_285V6.1   TSQRSRGGSAE-TESSIL-EDLPLGSSCFQELLDHFGQQELWKAQKAIIRQQKVFSRQVFELH
Smo415241|PACid  -SPEEVEASDD-SATSVVENNAAPSAITSKEIMSAVGDEEFWKMRKAMQRQQTIFQKQLFELH
LOC_Os06g05060   LEQHDAENIDD-VSDSSV-ECITGWEISPDKIVGAIGTKHFWKARRAIMNQQRVFAVQVFELH
LOC_Os01g38530   --------IDD-LSDSSV-ECITAWEISPDEIVGAIGAKHFWKARRAIINQQRVFAAQVFELH
At2g25930        LQQINEEASDDVSDDSMV-DSISSIDVSPDDVVGILGQKRFWRARKAIANQQRVFAVQLFELH
At3g21320        ----PRREQVA-SNCSAI-ESLSGISASSYDIARVIGEKRFWKMRTYMINQQKIFAGQVFELH

cons                            .   .           .:  .* . :*: :  : .**  *  *:**:*
```

```
kfl00240_0090_v   RLVKVQKMFAENPDFTTEQPPPLTSTKRLHPRDSRWPEPSTAVTPERSAVSDV--RRNPFSIP
Pp1s87_90V6.1     RLIEVQHLLAKSPSLSHNLD----DVLEAE-------GDNVPLPSSPV-------AE----VP
Pp1s86_214V6.1    RAIEVQQLLAKALCFSLHVD----EVPQKV--------DNILRPSSPV-------AE----VP
Pp1s11_285V6.1   RVIEVQRLLAKLPSLSFRLD----EVIEAE-------GDNVPLLDSPV-------AE----VP
Smo415241|PACid   RLTKVQHLMANSKI---SDPSKTQ--------D-EKTDKRVGSEPTHC-------GE----AP
LOC_Os06g05060    KLVKVQKLIAASPHVLIESD----PCLG----------NALLGSKNKL-------VEE---NL
LOC_Os01g38530    KLVKVQKLIAASPHVLIEGD----PCLG----------NALLASKKKM-------AEE---NL
At2g25930         RLIKVQKLIAASPDLLLDEI----SFLG-----------KVSAKSYPVK---KLLPSE---FL
At3g21320         RLIMVQKMVAKSPNLFLESK------LNGV-------KHG--TMRS---------------S-

cons              :   **::.*


kfl00240_0090_v   Q---NSGS---------VSHADAFKAAKTSE----------FGN---------PSSTRYVASK
Pp1s87_90V6.1     E---IPD------------FPESSGRLLERE----------VSR---------FSEE------
Pp1s86_214V6.1    E---TPEALEIPKATEIPEVPEVSERRFETK----------VCK---------DLNE------
Pp1s11_285V6.1    E---VPE------------VPEVSGRSLEKD----------ISR---------PSDE------
Smo415241|PACid   K---TSKP---------GPKPDQAPAPKTPS----------QTQ---------PLST------
LOC_Os06g05060    KAQPLLVA----------TIDDV--EPSLQQPE--------VSKENTEDSPPSPHDT---GLG
LOC_Os01g38530    KAQPVLVA----------TNDDV--QPSLQEPE--------LSKENSEENPPSPRDT---APV
At2g25930         VKPPLPHV----------VVKQRGDSEKTDQHKMESSAENVVGR---------LSN-------
At3g21320         ----------------------HQLAMAASK----------VRK---------PNTE------

cons                                            .            .


kfl00240_0090_v   VAQISTQYGPREAVMAAQEAARRSFQEVSSATVAPVNVQAAEALALVAQRVGESC---SKLM-
Pp1s87_90V6.1     ----I--S----------------------------------------RPVSESR---SQPL-
Pp1s86_214V6.1    ----N--S----------------------------------------RSVPESV---SQPL-
Pp1s11_285V6.1    ----I--S----------------------------------------SQVPESL---SQPA-
Smo415241|PACid   ---------------------------TPYAYTTHPRPIQAA---------------------
LOC_Os06g05060    ---SG--QRDQA------------------------------------ATNGVSK---SNRR-
LOC_Os01g38530    ---SG--HHDQT------------------------------------AKIGASK---SNLR-
At2g25930         ---QG------------------------------HH------------QQSNYMPFANNP--
At3g21320         ----N--H----------------------------------------KPVPEEY---PEHMK

cons


kfl00240_0090_v   -A-HRAESEKSGGQAVSAEDSGAQVVLEEATRLSATLEAMRPHLQGNTAPAM--PNPWLQNAL
Pp1s87_90V6.1     -QLPPSITSQQN--------------FNSG--AAWQ----------A-APYS--SNPYAARIS
Pp1s86_214V6.1    --YPLPLPFQQG--------------FNSG--SAWP----------A-GPYA--ANPCATRMM
Pp1s11_285V6.1    --YPLSTPPQQG--------------FNPA--SPWQ----------A-ATYS--SNPYAARMS
Smo415241|PACid   ----------NT------------------------------INYA-TSHPA--YNQWYAPLP
LOC_Os06g05060    -ATPVASDNKQN---------------NWG--VQ--------------LQPP--QNQWLVPVM
LOC_Os01g38530    -ATPVASDNRQN---------------NCG--VQ--------------LQPP--QNQWLIPVM
At2g25930         ---PASPAP-NGY-------------CFPP--Q-------------P--PPSGNHQQWLIPVM
At3g21320         PKLPLPSISKEL--------------VTPI--WPQQ-----------LLPPP--GNQWLVPVI

cons                        .                                            :


kfl00240_0090_v   AGAYGAYYPQMTAFSNPYLNPAMYMRQAQSMMAGYP-SY-D--SASASFFG-GEAMSAPAGGG
Pp1s87_90V6.1     PP---Y----------------MYVPYPGPCSPGYG-VY-P--MM-----G--APMSMY----
Pp1s86_214V6.1    SP---Y----------------MYVPYSGPCPPGYG-AY-P--MM-----D--APMPRF----
Pp1s11_285V6.1    SP---H----------------MYVPYPGPCSPGYG-VY-P--MM-----G--APMSMY----
Smo415241|PACid   ------FQF-------PFQQP---------F------------PVTAQVFN-TPYYPAPLYGG
LOC_Os06g05060    SPLEGL----------------VYKPYSGPCPPAGS-ILAP--FY-----ANCTPLSLP----
LOC_Os01g38530    SPSEGL----------------VYKPYSGPCPPAGS-ILAP--FY-----ANCTPLRLP----
At2g25930         SPSEGL----------------IYKPHPGMAHTGHYGGY-YGHYM-------PTPMVMP----
At3g21320         TDSDGL----------------VYKPFPGPCPPSSS-AF-M--------------VPVY----

cons
```

```
kfl00240_0090_v   PYGSAAGGYMPMY-PKYSELQGAAGLNPALNPW-MQNP-----DPVTAAWLATMAG--GGVAP
Pp1s87_90V6.1     --GNS-GGMQASRFPIWQQ-PG------MSQPWCPQDPTAAAAAAAAAVWYGQQVV--PG-GP
Pp1s86_214V6.1    --GYP-GVMQTVRFPTWSQ-PS------MPQTWTSLNA-----AAAAAAWYGQHVS--AA-VP
Pp1s11_285V6.1    --GNF-GGMQASRFPTWQQ-QG------MSQPWGPSDP-----AAAGAVWYGQQMV--PAVGP
Smo415241|PACid   -------GAAPPFLPPPDPRQFMS-PPIQLQPW-PQHPGSLYSDPAALQWMGFV-------NP
LOC_Os06g05060    --STA-GDFMNSAYG---------------VPM-PHQPQ----------HMGAPGPPSMP--M
LOC_Os01g38530    --STT-GDFMNSAYG---------------VPI-PHQPQ----------HMGAPGTPTMP--M
At2g25930         --Q------------------Y--------------HPG-----------MGFP-P--PG--N
At3g21320         --GQD-SLETPFRFPVSS-------------------------------------P--FS--H

cons

kfl00240_0090_v   -WNLPNNANHPAGFTNPQSGFPGGANPMGPTPSH-S-------SQSAMPVQSSW--ANFRGPE
Pp1s87_90V6.1     TPT-M-------GVVSLGTNSERLTSSRATRPLQ-GSNALKGNAWQGEETTDVEVKRSNPVNA
Pp1s86_214V6.1    TAN-T-------GVASPARNSQRLISSGSGQSVE--------NTQQAGVGSDVGVNLVNKVGP
Pp1s11_285V6.1    ATN-M-------GVIPMVTNCERQTSSGAAQLIQ-RNYAQKGSARQAGEKSDVGGNRSKLVNA
Smo415241|PACid   ---------------------PGGATY-RPPAAESS--------LQASSIHDDHQSRKDRGIH
LOC_Os06g05060    ------------NYFPPFSI--PVMNPTAPAPVV----------EQGRH--------------
LOC_Os01g38530    ------------NYFPPFSV--PVMNPVALASAV----------EQGRH--------------
At2g25930         ------------GYFPPYGMMPTIMNPYCSSQQQ-----------QQQQPNEQ----------
At3g21320         ------------SYFPPPNA----------RTTV----------D------------------

cons

kfl00240_0090_v   EYSSGAGDAFAYDQFRPRS--SAGRDG--PT--QSFSGLSHSWAGGM------PQGG-AASTR
Pp1s87_90V6.1     VENSGRTKDRGTTE--NRWDREEPYTS--QN--NQ--------CGAV------PQSG-EIQCR
Pp1s86_214V6.1    LDDRGWAKGWGSSE--RQRASGEGNPQ--NK--TS--------FAGLRGDNGNCQNS-EDH-R
Pp1s11_285V6.1    VESCGWAKGWGSTE--DRRGHREPDAS--RN--KE--------FGAV------PLGD-ESERQ
Smo415241|PACid   LRSQGSSSGFSLIQRADS---SSPAPA--PS--PSPA--AENRRQQH------QEPR-QKLVG
LOC_Os06g05060    PS---MPQPYGNFE--QQSWISCNMSH--PS----G-------IWRF------HASRDS--EA
LOC_Os01g38530    PS---MPQPYGNLE--QHSRMSCNMSH--PS----G-------IWRF------HASRDS--EA
At2g25930         MNQFGHPGNLQNTQ--QQQQRSDNEPA--PQQQQQP-------TKSY------PRAR-K--SR
At3g21320         -----QTNPFGQFQ--RWSNTSSHMTQAIPF----S-------LKKS------QESNDS--DI

cons                           :

kfl00240_0090_v   LNFASQSSDAHGESRQGD--WSEQTFSAGGGDGMLGKRKEVDAHPRWSGWPQRQDSRGTAED-
Pp1s87_90V6.1     ------D----YFTGREETGGGLGQPSDVG-RMVEREE-------HRGEHSCRGGEPFGLGEE
Pp1s86_214V6.1    ------Y----GETERDVSGCGVRQRTNEDDRGVECD---------------------VDSRE
Pp1s11_285V6.1    ------D----GETRRVETGGGVGQPNDEGVRMVEREKNSGVLSAHGREVSTRGGELCDHGEE
Smo415241|PACid   RFCGEKQSGGESEHGVS----------------------------------------------
LOC_Os06g05060    ----------QASSASS---------------------------------------PFDRFQC
LOC_Os01g38530    ----------QASSASS---------------------------------------PFDRLQC
At2g25930         ----------QGSTGSS---------------------------------------PSGPQGI
At3g21320         ----------HGSTASS---------------------------------------PPEKHKL

cons

kfl00240_0090_v   ----SFQHGGRSEMTHAGSFFSQSHRIKGGGNDSDEDALRAGRLPGSQFYSSPDFSDQGGAGD
Pp1s87_90V6.1     SAGPKLEKKWPAGARGEEP-----LRS---------TGL---KRPR---------EVQ-----
Pp1s86_214V6.1    YREPDSEKSWPAETSHAGP-----LQG---------RSL---KHMRS--------ESL-----
Pp1s11_285V6.1    LGGLELERNLPAGARHKEP-----LEV---------RTL---KRPRT--------EFP-----
Smo415241|PACid   -------------------PYSRP----------------------SGFQSV-----------
LOC_Os06g05060    ------------------------------------------SGSGP--------VSA-----
LOC_Os01g38530    ------------------------------------------GGSGP--------VSA-----
At2g25930         SG-SKSF------RPFAAV-----DED---------SNI---NNAPE--------QTM-----
At3g21320         ------------------------------------------EVLPL----------------

cons
```

```
kfl00240_0090_v   GGKEVNELQLFPVKSD-AEKKRGAAAPIKVVPRA-ASETPDLGVSILKSIQRER--------
Pp1s87_90V6.1     ---VSDMSSWFSIMSPAK-RIAQQNGVIKVVPRA-VSATPESAASILLSIQMERQR------
Pp1s86_214V6.1    ---EPSASRWFPTLSPAKRVMHKYGGVIKVVPRA-VSATQESTASILLSIQTERRR------
Pp1s11_285V6.1    ---KSDGFPWFPIISPAK-RVMQQCGVIKVVPRA-VSATQESAASILLSIQKERQR------
Smo415241|PACid   -----KNS---SSSSKEQRSSQGSHKAIKVTPRA-APVTAESAAEILHSIQKERPS------
LOC_Os06g05060    ---FPTVSAQNNQPQPSYSSRDNQTNVIKVVPHN-SRTASESAARIFRSIQMERQRDD----
LOC_Os01g38530    ---FPTASAQNTQPQPSSGSRDNQTNVIRVIPHNNSQTASESAARIFRSIQMERQQDDS---
At2g25930         ---TTTTTTTRTTVTQTTRDGGGVTRVIKVVPHN-AKLASENAARIFQSIQEERKRYDSSKP
At3g21320         ---FPTEPTHQT-D-EYKQKQQPMLRAIKAVPHN-STSASESAARIFRFIQEERRDSDHMIS

cons                                         *:. *:     : :  . *:  ** **
```

```
T-COFFEE, Version_11.00.d625267 (2016-01-11 15:25:41 - Revision d625267 - Build 507)
Cedric Notredame
CPU TIME:0 sec.
SCORE=831
*
 BAD AVG GOOD
*
kf100065_0150_v   :  70
kf100184_0080_v   :  85
Pp1s180_31V6.1    :  82
Smo118674|PACid   :  87
Smo91083|PACid_   :  88
MA_4172g0010      :  94
MA_57007g0010     :  92
MA_8565294g0010   :  93
MA_866497g0010    :  90
MA_99665g0010     :  93
LOC_Os03g29680    :  88
LOC_Os08g27860    :  76
LOC_Os11g40610    :  92
At1g17455         :  84
At1g72630         :  91
At2g06255         :  91
At2g29950         :  73
At2g40080         :  82
cons              :  83
```

ELF4

```
kf100065_0150_v   -------------------MASLMPCQDLPWQKGYSENEP-S---------------------
kf100184_0080_v   -------------------M------DLD----------------------------------
Pp1s180_31V6.1    -------------------M------DRE--------RG------------------------
Smo118674|PACid   -------------------M------EAD--------ASS-A---------------------
Smo91083|PACid_   -------------------M------ESS----------------------------------
MA_4172g0010      -------------------M------EGD--------AYA-A---------------------
MA_57007g0010     -------------------M------EGD--------TYY-A---------------------
MA_8565294g0010   -------------------M------EGE--------AYS-A---------------------
MA_866497g0010    AEGIRRTKDLYLFLLLHLKM------EGD--------AYS-I---------------------
MA_99665g0010     -------------------M------EGD--------AYS-I---------------------
LOC_Os03g29680    -------------------M------DGD--------TLS-A---------------------
LOC_Os08g27860    -------------------M------EED--------SVI-SNGGREEEVVVANGSGGGVGGT
LOC_Os11g40610    -------------------M------EGD--------SFS-G---------------------
At1g17455         -------------------M------EGD--------VLS-G---------------------
At1g72630         ---------------MESRM------EGD--------VYS-G---------------------
At2g06255         -------------------M------EGD--------TIS-R--------------------M
At2g29950         ------------------ME-------AS--------RNRSL-VGNNRSPEMNEN---DGEDV
At2g40080         ------------------MKR------NGE-------TKRRR-NVAEEAEQGE----------

cons

kf100065_0150_v   TAPEALGISKEWPGAAN-FDKVQMILDQNRLLIKEINQNHCARRTEGLSRNVLLIRELHSNVA
kf100184_0080_v   TGPCPVFDPHEWPGFEK-FIIVQAILDHNKLLINEINLNHERRRPEGLTRNVQLIRELNENVT
Pp1s180_31V6.1    -DKAPPVDRRVSSIFNNVSRQVQFLLDHNRLLINEINQNHEAKIPEGLTRNVMLIRQLNSNIG
Smo118674|PACid   PAKAPPVDRKMWAAFERGFSQVQFLLDHNRLLINEINQNQESKVPESLSRNVVLIKELNKNIG
Smo91083|PACid_   L---GGAEGKAWGVFQRTFHQVQFLLDHNRLLIKEINVNQESQIPESLSRNVMLIKELNYNIK
MA_4172g0010      LGNGNQIDDKVLQTFQKSFGQVQNILDQNRLLINEINQNHESKVPDNLSRNVALIRELNNNIT
MA_57007g0010     PANGNQIDGKVMQTFQKSFGQVQNILQHNRLLINEINQNQESRTPDNLSRNVGLIRELNNNIT
MA_8565294g0010   PGNGNQIDGKVLQTFQKSFGQVQSILDQNRLLINEINQNHESKIPDNLSRNVSLIRELNNNIS
MA_866497g0010    PGNGNQMDSKVMQTFQKGFGQVQNILDENRLLIKEINQNQESKIPDNLSRNVALIRELNNNIT
MA_99665g0010     PWNGIQIDGKVMQTFQKSLGQVQNILDQNRLLIKEINQNHESKMSDNLTRNVTLIREFNNNIT
LOC_Os03g29680    ---AAAEDGKVLHAFQTSFVQVQSLLDQNRVLINEINQNHESKVPGDLSRNVGLIRELNNNIR
LOC_Os08g27860    ARSSGGGGGKVVQVLQRNFGEVQGILEQNRVLIQEISQNHEARDADGLTRNVALIRELNTNIA
LOC_Os11g40610    MANGGQVDNKLIQTFHKSFVQVQSILDQNRMLINEINQNHESRAPDNLTRNVGLIRELNNNIR
At1g17455         FGDRHNMDGKLLQSFQKSFVDVQDILDQNRLLINEINQNHESKQPDNLGRNVGLIKELNNNIR
At1g72630         FGERYQMDGKLLQNFQKSFVQVQDILDQNRLLINEINQNHESKQADHLGRNVGLIRELNNNIR
At2g06255         MGSGVQMDGKILQTFEKSFVQVQNILDHNRLLINEINQNHESKIPDNLGRNVGLIRELNNNVR
At2g29950         AASAAVEDVEVWDTLSNGFKRAQLYLDQNRDLIQRVNENHMSRIPDNVSRNVGLINEINGNIS
At2g40080         -------DPAMWENLDRNFRQVQSVLDRNRSLIQQVNDNHQSRMADNMSKNVALIQELNGNIS
```

```
cons                                     .*  *:.*: **:.:. *:  : .  : :** **.::: *: 


kfl00065_0150_v      KVVELYREISESFVVVFGNHQVQ----------------------------------------
kfl00184_0080_v      KVMKLYEELSQAFVQSFGKNAGQP---------------------------------------
Pp1s180_31V6.1       KVVDLYANLSSNFSNVFENLQEVNSDPINTTSRPVSNSSGRSVPGVAATQSSGQN-PHKKLRS
Smo118674|PACid      QVVSLYSTISSSFVKSFENSQEGDSIPAPGTAKAMDSP-------AAA---AGAV-GHKRVRS
Smo91083|PACid_      RVVELYAGLPTSFIRSFSNLPSQVTAA-TGTY--P------------A-----EQ-GLKRARS
MA_4172g0010         RVVDLYACLSTSVTKEI--ETS-----------------------------------------
MA_57007g0010        RVVDHYAGLSTSFTKSI--ETSSDGDSA-GTLTSDGK----------P---VLVS-GQKRFRT
MA_8565294g0010      RVVDLYSGLSTSFTKAV--ETSSEGDSA-GTLKSDGK----------P---AVAS-GQKRFRP
MA_866497g0010       RVVDVYAGLSTSVTKAI--ETS-EGNSA-GTLKSDGN----------P---AGSSSGQKRFRF
MA_99665g0010        RVVDLYAGLSTSFTKAI--ETSSEGDSA-GTLRSDGR----------H---AGSS-GQKRFRL
LOC_Os03g29680       RVVDLYADLSSLFAASSPGPAASEGASV-GTA---VR----------H------A-GHKRVRS
LOC_Os08g27860       RVVDLYANLSGSFSRSVTAAASANNTNA-TNSTSPSA----------A---AAAK-ASKRSRA
LOC_Os11g40610       RVVGLYADLSASFARTM--DASSEGDSS-GTLRSSDG--------------AGRS-GQKRVRP
At1g17455            RVASLYGDLSHSFARSV--DASSEGESS-GTLKSDGK-----------------A-NQKRFRS
At1g72630            TVASLYGDLSHSFARSV--DASSEGEST-GTLKSDGK----------------AN-NQKRFRS
At2g06255            RVAHLYVDLSNNFSKSM--EASSEGDS------SEGR-------------------GNRRIRP
At2g29950            QVMEIYSDLSLNFAKKFDQRRRTTKDGD-TT--------------------------T--TTT
At2g40080            KVVNMYSDLNTSFSSGFHGG--KNGHDG-GG--------------------------AAGTR-

cons                  *   *  :   .                                                  


kfl00065_0150_v      --R-
kfl00184_0080_v      AKS-
Pp1s180_31V6.1       TDFP
Smo118674|PACid      S---
Smo91083|PACid_      T---
MA_4172g0010         ----
MA_57007g0010        ----
MA_8565294g0010      Q--F
MA_866497g0010       ----
MA_99665g0010        ----
LOC_Os03g29680       THLD
LOC_Os08g27860       TDTE
LOC_Os11g40610       G---
At1g17455            G---
At1g72630            G---
At2g06255            A---
At2g29950            GS--
At2g40080            -A--

cons                     
```

ZTL

```
T-COFFEE, Version_11.00.d625267 (2016-01-11 15:25:41 - Revision d625267 - Build 507)
Cedric Notredame
CPU TIME:0 sec.
SCORE=881
*
 BAD AVG GOOD
*
kf100059 0220 v     :  85
Smo174189|PACid     :  89
MA 70291g0010       :  89
LOC Os11g34460      :  83
LOC Os02g05700      :  82
LOC Os06g47890      :  81
At2g18915           :  81
At1g68050           :  81
At5g57360           :  85
cons                :  88

kf100059_0220_v     ----MSYPGR--------------DAYEEMDDDGVPLFLRGD----------DEKTRRRIEVL
Smo174189|PACid     ---------------------------------------------------------------
MA_70291g0010       ---------------------------------------------------------------
LOC_Os11g34460      MF----DAGDRGG-GGGVVAVKRMKLCEEEEEEEEGMEVDEEEEEVGWVWRPPGGLAGEDEAA
LOC_Os02g05700      ----MEWDSDSEGSGD--------EEEEEEEEEEEGVEVGGG-GDGGVGVGVGGGFALAIEGV
LOC_Os06g47890      ----MEWDSESDGAGS-IGA---GEEEEEEEEEEEGGFGG----GGGGGGGGGGMFSFAIEGM
At2g18915           MQNQMEWDSDSDLSGG--------DE--VAED---GWFG-----------GDNGAIPFPVGSL
At1g68050           MA----REHAIGEATG-KRK-KRGRVEEAEEYCNDGIEEQVE----------DEKLPLEVGMF
At5g57360           ----MEWDSGSDLSAD--------DASSLADDEEGGLFP-----------G-GGPIPYPVGNL

cons


kf100059_0220_v     L-HSSPHGLIVTDALAEDHPITYVNTIFQYYTGYDAESILGRNCRFLQMRGEFADKRHPAVDL
Smo174189|PACid     --MLGPCSVVVTDALDVDFPIIYVNNIFEFITGYKAEEVLGRNCRFLQFRGPFAQRRHPLVDS
MA_70291g0010       --------------------------------------------RFLQYRGPFAQRRHPLVDS
LOC_Os11g34460      AWEGRAAAIVVSDAVEVDFPVIYVNAAFEAATGYRADEVLGRNCRFLQFRDPRAQRRHPLVDP
LOC_Os02g05700      L---GACGLVVSDALEPDFPIIYVNRGFEDATGYRAEEVLGRNCRFLQCRGPFAKRRHPLVDT
LOC_Os06g47890      LRASGPCGLVVTDALEPDCPIIYVNCGFEEATGYRAEEVLGRNCRFLQCRGPFAQRRHPLVDA
At2g18915           P-GTAPCGFVVSDALEPDNPIIYVNTVFEIVTGYRAEEVIG----------PFTKRRHPMVDS
At1g68050           YYPMTPPSFIVSDALEPDFPLIYVNRVFEVFTGYRADEVLGRNCRFLQYRDPRAQRRHPLVDP
At5g57360           L-HTAPCGFVVTDAVEPDQPIIYVNTVFEMVTGYRAEEVLGGNCRFLQCRGPFAKRRHPLVDS

cons                                                                  :.:*** **


kf100059_0220_v     KTVRKMREAIAAGQEFKGELLNFKKDGTPLINNLLMTPIHGEDGIVTHFIGIQSFRPIKMDLG
Smo174189|PACid     ATVTEIRRCMREGIEFWGELLNFRKDGTPLMNKLCLKPIRGEDGRITHIIGIQSFSEVKLDLG
MA_70291g0010       MVVSEIRRCLDEGVEFQGELLNFRKDGTPLMNRLRLTPIHGDDGIITHIIGIQLFTEANIDLG
LOC_Os11g34460      MVVSEIRRCLNEGIEFQGELLNFRKDGAPLYNRLRLIPMHGDDGFVTHVIGIQLFSEANIDLS
LOC_Os02g05700      TVVTDIRRCLEEGTVFQGDLLNFRKDGSPFMAKLQLTPIYGDDETITHYMGMQFFNDSNVDLG
LOC_Os06g47890      MVVSEIRKCIDNGTEFRGDLLNFRKDGSPLMNKLHLTPIYGDDETITHYMGIQFFTNANVDLG
At2g18915           TIVAKMRQCLENGIEFQGELLNFRKDGSPLMNKLRLVPIREEDE-ITHFIGVLLFTDAKIDLG
At1g68050           VVVSEIRRCLEEGIEFQGELLNFRKDGTPLVNRLRLAPIRDDDGTITHVIGIQVFSETTIDLD
At5g57360           MVVSEIRKCIDEGIEFQGELLNFRKDGSPLMNRLRLTPIYGDDDTITHIIGIQFFIETDIDLG

cons                  *  .:*..:   *   *  *:****:***:*:   .*  :  *:   :*   :**  :*:   *     :**.


kf100059_0220_v     PLPERPWKDVPPVRHSELPPVQPSEESFAAGTFGG----ASRHNPAVFEALSDEVLVSRILGR
Smo174189|PACid     PLPPPLWRNQSSQHWLVRRHLLDDGYSYASSPVG--PGQSSKDRCGIL-RLSDEVLVQKILAQ
MA_70291g0010       PMPLPFYKEAITR---CADRSLSDPFLYRPAAIG--QGHFCRE--------------------
LOC_Os11g34460      NVSYPVYKQQSNHRPNI------QEINPASHEHI--PKIQSSEYCCIL-QLSDEVLAHNILSR
LOC_Os02g05700      PLSVSTTKEIVRSTL-----ITPDNT-IRPSPMGKG---FCSEHSDLF-LLSDEVLCQKILSR
LOC_Os06g47890      PLPGSLTKEPVRSTR-----FTPDNF-FRPISTGPGQSNFCREYSSLF-QLTDEVLCQSILSR
At2g18915           PSPDLSAKEIPRIS----------RSF-TSALPIGER--NVSRGLCGIF-ELSDEVIAIKILSQ
At1g68050           RVSYPVFKHKQQLDQTS-------ECLFPSGSPR--FKEHHEDFCGIL-QLSDEVLAHNILSR
At5g57360           PVLGSSTKEKSI-----------DGI-YSALAAGER--NVSRGMCGLF-QLSDEVVSMKILSR
```

```
cons                      :.                    .

kfl00059_0220_v      LAPKDVAICSMVCRRFRRLGQDDYIWKRVCRNSWGHNTAAAIQAALDSQTPSLGWARIARELT
Smo174189|PACid      LTPRDVSSVALVCRRFNEMTKNTDLWRLVCRNAWGLETTAVLERVHN--PRSIDWGMLARELT
MA_70291g0010        ----------------------------IC-------------------AKRLGWGRLARELT
LOC_Os11g34460       LSPRDVASIGSVCTRMHELTKNDHLRKMVCQNAWGRDVTVRLEM--S--TKMLGWGRLARELT
LOC_Os02g05700       LSPRDIASVNSVCKRLYHLTRNDDLWRMVCQNAWGSEATQVLETVAG--TRSLAWGRLARELT
LOC_Os06g47890       LSPRDIASVSSVCRRLYLLTRNEDLWRMVCQNAWGSETTRALETVPA--AKRLGWGRLARELT
At2g18915            LTPGDIASVGCVCRRLNELTKNDDVWRMVCQNTWGTEATRVLESVPG--AKRIGWVRLAREFT
At1g68050            LTPRDVASIGSACRRLRQLTKNESVRKMVCQNAWGKEITGTLEI--M--TKKLRWGRLARELT
At5g57360            LTPRDVASVSSVCRRLYVLTKNEDLWRRVCQNAWGSETTRVLETVPG--AKRLGWGRLARELT

cons                                            :*                   .  : *  :***:*

kfl00059_0220_v      TLEAAAWRKFTVGGSVEPSRCNFSECAVGNKLVLFGGEGVNMQPMNDTFVLDLSLEHPAWRHV
Smo174189|PACid      TLEAAAWRKLKVGGAVEPSRCNFSACAVGNKVVLFGGEGVNMQPMNDTFVLDLSAACPEWRHV
MA_70291g0010        TLEAAAWRKLTVGGAVEPSRCNFSACAVGNRLVLFGGEGVNMQPMNDTFVLDLSAANPEWRHV
LOC_Os11g34460       TLEAASWRKFTVGGRVEPSRCNFSACAVGNRLVLFGGEGVNMQPMDDTFVLNLESAKPEWRRV
LOC_Os02g05700       TLEAVTWRKLTVGGAVEPSRCNFSACAAGNRVVLFGGEGVNMQPMNDTFVLDLNASKPEWRHI
LOC_Os06g47890       TLEAVAWRKLTVGGAVEPSRCNFSACAVGNRVVLFGGEGVNMQPMNDTFVLDLNASNPEWRHV
At2g18915            THEATAWRKFSVGGTVEPSRCNFSACAVGNRIVIFGGEGVNMQPMNDTFVLDLGSSSPEWKSV
At1g68050            TLEAVCWRKFTVGGIVQPSRCNFSACAVGNRLVLFGGEGVNMQPLDDTFVLNLDAECPEWQRV
At5g57360            TLEAAAWRKLSVGGSVEPSRCNFSACAVGNRVVLFGGEGVNMQPMNDTFVLDLNSDYPEWQHV

cons                 * **. ***:.*** *:******* **.**::*:**********::*****:*    * *: :

kfl00059_0220_v      DVSAAPPGRWGHTLCCLNGSWLVVFGGCGTDGPLNDVFVLDLDAEHPAWREVAGAGPPLPRSW
Smo174189|PACid      DVGSAPPGRWGHTLSCLNGSWLVVFGGCGRQGLLNDVFVLDLDAKQPSWREVAGVGPPVPRSW
MA_70291g0010        NVSSPPPGRWGHTLSCLNGSWLVVFGGCGRQGLLNDVFILDLDAQQPTWREVAGSAPPLPRSW
LOC_Os11g34460       KVSASPPGRWGHTLSWLNGSWLVVFGGCGQQGLLNDVFVLDLDAKQPTWREVASEGPPLPRSW
LOC_Os02g05700       NVRSAPPGRWGHTLSCLNGSRLVLFGGCGRQGLLNDVFMLDLDAQQPTWREIPGLAPPVPRSW
LOC_Os06g47890       NVSSAPPGRWGHTLSCLNGSLLVVFGGCGRQGLLNDVFTLDLDAKQPTWREIPGVAPPVPRSW
At2g18915            LVSSPPPGRWGHTLSCVNGSRLVVFGGYGSHGLLNDVFLLDLDADPPSWREVSGLAPPIPRSW
At1g68050            RVTSSPPGRWGHTLSCLNGSWLVVFGGCGRQGLLNDVFVLDLDAKHPTWKEVAGGTPPLPRSW
At5g57360            KVSSPPPGRWGHTLTCVNGSNLVVFGGCGQQGLLNDVFVLNLDAKPPTWREISGLAPPLPRSW

cons                  * :.*********  :*** **:*** * .* ***** *:***. *:*:*:..  **:****

kfl00059_0220_v      HSSCTVDGTYLVVFGGCTDTGRLLSDTFLLDLTAQKPVWREIVGGFKPPSRLGHSLSVLEGSK
Smo174189|PACid      HSSCTLDGTQLVVYGGCADSGVLLSDTYMLDISKEKPMWREIPVAWTPPSRLGHSLSAYGGRK
MA_70291g0010        HSSCTLDGTKLVVSGGCADSGVLLSDTFLLDLTMEKPIWKEIPVSWTPPSRLGHSLTVYGGRK
LOC_Os11g34460       HSSCTLDGSKLVVSGGCTESGVLLSDTFLLDLTKEKPAWKEIPTSWSPPSRLGHTLSVFGKTK
LOC_Os02g05700       HSSCTLDGTKLVVSGGCADSGVLLSDTYLLDVTMERPVWREIPASWTPPCRLGHSLSVYDGRK
LOC_Os06g47890       HSSCTLDGTKLVVSGGCADSGVLLSDTYLLDVTMDKPVWREVPASWTPPSRLGHSMSVYGGRK
At2g18915            HSSCTLDGTKLIVSGGCADSGALLSDTFLLDLSMDIPAWREIPVPWTPPSRLGHTLTVYGDRK
At1g68050            HSSCTIEGSKLVVSGGCTDAGVLLSDTFLLDLTTDKPTWKEIPTSWAPPSRLGHSLSVFGRTK
At5g57360            HSSCTLDGTKLIVSGGCADSGVLLSDTFLLDLSIEKPVWREIPAAWTPPSRLGHTLSVYGGRK

cons                 *****::*: *:* ***:::* *****::**:: : * *:*:   : **.****:::.    *

kfl00059_0220_v      VLMFGGLAQSGPLRLRSSDVFTIDVGIQEPNWNYLTGSMLPGGAPATG-PSPPPRLDHVAATL
Smo174189|PACid      ILLFGGLAKSGPLRFRSSDAFTIDLGEEEPTWKYVTGSTLPGGANIGG-TTPPPRLDHVAVTL
MA_70291g0010        ILMFGGLAKSGPLRLRSSDVYTIDLSEEEPKWRYLTGSGMPGAGNPGG-KAPPPRLDHVAVSL
LOC_Os11g34460       LFMFGGLAKSGSLRLRSCDAYTMDAGEDSPQWRQLATTGF-----PS--IGPPPRLDHVAVSL
LOC_Os02g05700       ILMFGGLAKSGPLRLRSNDVFTLDLSENKPCWRCITGSGMPGASNPAG-VGPPPRLDHVAVSL
LOC_Os06g47890       ILMFGGLAKSGPLRLRSSDVFTMDLSEEEPCWRCLTGSGMPGAGNPAG-AGPPPRLDHVAVSL
At2g18915            ILMFGGLAKNGTLRFRSNDVYTMDLSEDEPSWRPVIGYGSSLP---GGMAAPPPRLDHVAISL
At1g68050            ILMFGGLANSGHLKLRSGEAYTIDLEDEEPRWRELECSAF-----PGV-VVPPPRLDHVAVSM
At5g57360            ILMFGGLAKSGPLKFRSSDVFTMDLSEEEPCWRCVTGSGMPGAGNPGG-VAPPPRLDHVAVNL
```

```
cons               :::*****:.* *::** :.:*:*   :.* *. :                **********.:

kfl00059_0220_v    PGGRVLIFGGSVAGQHSPIQLYVLDPKEGRPSWRMLNAPGLLPQYAWGHSTCVVGGGTRAVVL
Smo174189|PACid    PGGRILIFGGSIAGLHSASQIYLLDPSEEKPTWRMLNVPGQKPKFAWGHSTCFVG-GTRAVVL
MA_70291g0010      PGGRVLIFGGSVAGLHSASQLYLLDPTEEKPTWRILNVPGQQPRFAWGHSTCVVG-GTRALVL
LOC_Os11g34460     PCGRIIIFGGSIAGLHSPSQLFLLDPAEEKPTWRILNVPGQPPKFAWGHSTCVVG-GTRVLVL
LOC_Os02g05700     PGGRILIFGGSVAGLHSASKLYLLDPTEEKPTWRILNVPGRPPRFAWGHSTCVVG-GTKAIVL
LOC_Os06g47890     PGGRVLIFGGSVAGLHSASQLYLLDPTEEKPTWRILNVPGRPPRFAWGHSTCVVG-GTKAIVL
At2g18915          PGGRILIFGGSVAGLDSASQLYLLDPNEEKPAWRILNVQGGPPRFAWGHTTCVVG-GTRLVVL
At1g68050          PCGRVIIFGGSIAGLHSPSQLFLIDPAEEKPSWRILNVPGKPPKLAWGHSTCVVG-GTRVLVL
At5g57360          PGGRILIFGGSVAGLHSASQLYLLDPTEDKPTWRILNIPGRPPRFAWGHGTCVVG-GTRAIVL

cons               * **::*****:** .*. :::::** * :*:**:**  *  *: **** **.** **: :**

kfl00059_0220_v    GGFEGEERLLNELHELSLLDVEGGDGALGTSELEGRPEGAKQPDGSESDDKESGESSMMEERS
Smo174189|PACid    GGHTGEDWILNELHELSLSSTS-----------------------------------------
MA_70291g0010      GGHTGEEWILNELHELSLASKH-----------------------------------------
LOC_Os11g34460     GGHTGEEWILNELHELCLASRP-----------------------------------------
LOC_Os02g05700     GGQTGEEWTLTELHELSLVSSL-----------------------------------------
LOC_Os06g47890     GGQTGEEWMLTEIHELSLASST-----------------------------------------
At2g18915          GGQTGEEWMLNEAHELLLATST-----------------------------------------
At1g68050          GGHTGEEWILNELHELCLASRQ-----------------------------------------
At5g57360          GGQTGEEWMLSELHELSLASYL-----------------------------------------

cons               **  **:  *.* *** *

kfl00059_0220_v    ALE---N
Smo174189|PACid    QWIRRLQ
MA_70291g0010      GL----D
LOC_Os11g34460     DED---E
LOC_Os02g05700     ------V
LOC_Os06g47890     ------V
At2g18915          TAS---T
At1g68050          DSD---L
At5g57360          ------T

cons
```

GI

```
T-COFFEE, Version_11.00.d625267 (2016-01-11 15:25:41 - Revision d625267 - Build 507)
Cedric Notredame
CPU TIME:0 sec.
SCORE=917
*
 BAD AVG GOOD
*
Smo140066|PACid    :  93
MA_19575g0010      :  93
LOC_Os01g08700     :  92
At1g22770          :  91
cons               :  91

Smo140066|PACid   M--SSPQQKWLTGLKSTSLFRAPPLDLHERQTKTVAYVELFGQFAS--DSFPEDIAELVRDHY
MA_19575g0010     ---------------------------------------------------------------
LOC_Os01g08700    M--SASNEKWIDGLQFSSLFWPPPQDSQQKQAQILAYVEYFGQFTADSEQFPEDIAQLIQSCY
At1g22770         MASSSSSERWIDGLQFSSLLWPPPRDPQQHKDQVVAYVEYFGQFTS--EQFPDDIAELVRHQY

cons


Smo140066|PACid   PHKEPCLLDDVLATFVLHHPEHGHTILHPLLSCVIDGTLAYSKTTPPFGSFVSVFGVSSERDL
MA_19575g0010     ---------------------------------------------------------------
LOC_Os01g08700    PSKEKRLVDEVLATFVLHHPEHGHAVVHPILSRIIDGTLSYDRNGFPFMSFISLFSHTSEKEY
At1g22770         PSTEKRLLDDVLAMFVLHHPEHGHAVILPIISCLIDGSLVYSKEAHPFASFISLVCPSSENDY

cons


Smo140066|PACid   TEQWALACGEILRLLTHYNRPIYKSESSADGEKRSS----SDSGDPADRDGSGSPDN-GRRAP
MA_19575g0010     ---------------------------------------------------------------
LOC_Os01g08700    SEQWALACGEILRVLTHYNRPIFKVDHQHSEAECSSTSDQASSCESMEKRANGSPRNEPDRKP
At1g22770         SEQWALACGEILRILTHYNRPIYKTEQQNGDTERNCLSKATTSGSPTSEPKAGSPTQ-HERKP

cons


Smo140066|PACid   KRLLTPWITDSLLAAPLGTKSDYFRWCGGVLGKYAGGGDLRPPTTGDGKGHGKHPQLLSSTPR
MA_19575g0010     ------------------------------MGKYVAGGELKPPTTVGGRGPGKHPQLMPSTPR
LOC_Os01g08700    LRPLSPWITDILLAAPLGIRSDYFRWCGGVMGKYAAGGELKPPTTAYSRGSGKHPQLMPSTPR
At1g22770         LRPLSPWISDILLAAPLGIRSDYFRWCSGVMGKYAAGE-LKPPTI-ASRGSGKHPQLMPSTPR

cons                                            :***..*  *:***   .:* ******:.****


Smo140066|PACid   WAVANGAAVISSVCDDEVLRYETADLTAAAVPALLLPPPSTSLDEHLVAGLPPLEPFARLFHR
MA_19575g0010     WAISNGVGVILSVCDDEVTCYETVNLIVVAIPALLLPPPTTALDEHLVAGLPPLEPYARLFHR
LOC_Os01g08700    WAVANGAGVILSVCDEEVARYETANLTAAAVPALLLPPPTTPLDEHLVAGLPPLEPYARLFHR
At1g22770         WAVANGAGVILSVCDDEVARYETATLTAVAVPALLLPPPTTSLDEHLVAGLPALEPYARLFHR

cons              **::**..** ****:**  ***. * ..*:********:*.**********.***:******


Smo140066|PACid   YYAIATPGATQRLLLGLLEAPASWAPDALDAAVQLVELLRAAEDYSSSFRLPENWFRLHFLR
MA_19575g0010     YYAIATPSATQRLLLGLLEAPPSWAPDALDAAVQLVELLRAAEDYA-STMRLPKNWLHLHFLR
LOC_Os01g08700    YYAIATPSATQRLLFGLLEAPPSWAPDALDAAVQLVELLRAAEDYD-SGMRLPKNWMHLHFLR
At1g22770         YYAIATPSATQRLLLGLLEAPPSWAPDALDAAVQLVELLRAAEDYA-SGVRLPRNWMHLHFLR

cons              *******.******:******.***********************  * .***.**::*****


Smo140066|PACid   PMGAAMTMKQGIASDAAAALLFRLFSQPALLFPPRGHAQGAQVVQPLYGPPIRIDVLFHAQME
MA_19575g0010     AIGTALSMRVGIAADAAAALLFRTLSQPALLFPPPRLAQGVDVQCDVYGAFG--PSSSGEEIE
LOC_Os01g08700    AIGTAMSMRAGIAADTSAALLFRILSQPTLLFPPLRHAEGVELHHEPLGGYV---SSYKRQLE
```

```
At1g22770          AIGIAMSMRAGVAADAAAALLFRILSQPALLFPPLSQVEGVEIQHAPIGGYS---SNYRKQIE

cons               .:* *::*: *:*:*::****** :***:*****   .:*.::     *           ::*


Smo140066|PACid    ALATQVNEEATAKGVASLMRDHGRDVEWRICVLWEAAYGLIPLDKSVVDLPEMVIATPLQPPL
MA_19575g0010      METYQASIEATAQGVASLMCLHGPEVEWRICTLWEAAYGLLPLSSSTVDLPEIVVATPLQPPV
LOC_Os01g08700     VPASEATIDATAQGIASMLCAHGPDVEWRICTIWEAAYGLLPLSSSAVDLPEIVVAAPLQPPT
At1g22770          VPAAEATIEATAQGIASMLCAHGPEVEWRICTIWEAAYGLIPLNSSAVDLPEIIVATPLQPPI

cons                 : :.. :***:*:**::  ** :******.:*******:**..*.*****:::*:*****


Smo140066|PACid    LSWTLFRPFLRVLEHVPKGCQSQTCLRRIFSATVDAILRRTFPLDDWKEQ--KNGNFRSASGS
MA_19575g0010      LSWNLFQPLLRVLEYLPRGSPSEASLMRIFTATVQAILQRTFPAEQSMEQTRTTRNAHAGVGP
LOC_Os01g08700     LSWSLYLPLLKVFEYLPRGSPSEACLMRIFVATVEAILRRTFPSETS-EQSRKPR-------S
At1g22770          LSWNLYIPLLKVLEYLPRGSPSEACLMKIFVATVETILSRTFPPESSRELTRKARSSFTT-RS

cons               ***.*: *:*:*:*::*:*. *::.* :** ***::** **** :   *   .         .


Smo140066|PACid    GVDPAGMAELRALVHCLFTEAFLGPALASQLLSDALTVCLSHDTLRQGNG----------SDS
MA_19575g0010      TSKNLAVAELRTMVHSLFTESCVPIDLASRLLFMVITVCLSHDAVQKGSRKTTNDRGVLSNSS
LOC_Os01g08700     QSKNLAVAELRTMIHSLFVESCASMDLASRLLFVVLTVCVSHQALPGGSKRPT-----G-SDN
At1g22770          ATKNLAMSELRAMVHALFLESCAGVELASRLLFVVLTVCVSHEAQSSGSKRPRSEYAST-TEN

cons                 .  .::***:::*.** *:     ***:**  .:***:**::   *.           ...


Smo140066|PACid    SKKRSTHSSN----------KDRGAVASFDSYLIAAVCALACEVQLCTFSAADGTAFN-----
MA_19575g0010      MRDQQAINGSQKVNGH-RAKKERGAVATFGSYILAA---------------------------
LOC_Os01g08700     HSSEEVTNDSRLTNGRNRCKKRQGPVATFDSYVLAAVCALSCELQLFPFISKNGNHSNLKDSI
At1g22770          IEANQPVSNNQTANRKSRNVKGQGPVAAFDSYVLAAVCALACEVQLYPMISGGGNFSNSAVAG

cons                  ..  ...          * :*.**:*.**::**


Smo140066|PACid    -----------------GVTNSAYQARRLMSVLEGLLVVEPFSPGVGPNTNSPNDLVEAAIVA
MA_19575g0010      ---------------------------------------------------------------
LOC_Os01g08700     KIVIPGKTTGISNELHNSISSAILHTRRILGILEALFSLKPSSVGT-SWSYSSNEIVAAAMVA
At1g22770          TITKPVKINGSSKEYGAGIDSAISHTRRILAILEALFSLKPSSVGT-PWSYSSSEIVAAAMVA

cons


Smo140066|PACid    AHISRLLGRSRACTHALTAIVRCKWDPGVSSKAASILALVDGNDKAVEAVFNYADKLSGDEKR
MA_19575g0010      ---------------------------------------------------------------
LOC_Os01g08700     AHVSELFRRSRPCLNALSALKQCKWDAEISTRASSLYHLIDLHGKTVTSIVNKAEPLEAHLTL
At1g22770          AHISELFRRSKALTHALSGLMRCKWDKEIHKRASSLYNLIDVHSKVVASIVDKAEPLEAYLKN

cons


Smo140066|PACid    TLSSKQAQKIISGFTKDG-----------------------------------------VKDA
MA_19575g0010      ---------------------------------------------------------------
LOC_Os01g08700     TPVKKDEPPIEEKNINSSDGGALEKKDASRSHRKNGFARPLLKCAEDVILNGDVASTSGKAIA
At1g22770          TPVQKDSVTCLNWKQENTCASTTCFDTAVTSASRTEMN-PRGNHKYARHSDEGSGRPSEKGIK

cons


Smo140066|PACid    SLSLNASDVTNLLCGC--NGVSTTVSDLLKAVLKQKRDLAVAIVPLLWQRLMSAEELPTSKEG
MA_19575g0010      -------------------------------------------------RLITAPEMQMSVES
```

```
LOC_Os01g08700      SLQVEASDLANFLTMDR-NGGYRGSQTLLRSVLSEKQELCFSVVSLLWQKLIASPEMQMSAES
At1g22770           DFLLDASDLANFLTADRLAGFYCGTQKLLRSVLAEKPELSFSVVSLLWHKLIAAPEIQPTAES

cons                                                                 :*::: *:  : *.


Smo140066|PACid     TSAKQGWRQVVDAVCNVVLTYPEKATSVVLLQAERGIQPWIIGDG--GEEKWRMNTRIVFLLS
MA_19575g0010       TSAKQGWRQVVDALCNIVLASPAKAATAIVIQAERDLQPWVARHDSQGQQIWRLNQRIVSLLA
LOC_Os01g08700      TSAHQGWRKVVDALCDIVSASPTKASAAIVLQAEKDLQPWIARDDEQGQKMWRVNQRIVKLIA
At1g22770           TSAQQGWRQVVDALCNVVSATPAKAAAAVVLQAERELQPWIAKDDEEGQKMWKINQRIVKVLV

cons                ***:****:****:*::* : * **::.:::***: :***:  ..  *:: *::* *** ::


Smo140066|PACid     ELLRLND-PQVLGLIANAGTLLYQATDGMSVDGEPCTLPQLELLEAIAMAIKSLCAWKVSSR-
MA_19575g0010       ELLRYHNAPEALMVLANASDLLMRATDGMLVDGEACTTPQLELLEAMAVTAQLSLGWGVPGKA
LOC_Os01g08700      ELMRNHDSPEALVILASASDLLLRATDGMLVDGEACTLPQLELLEVTARAVHLIVEWGDSGVS
At1g22770           ELMRNHDRPESLVILASASDLLLRATDGMLVDGEACTLPQLELLEATARAIQPVLAWGPSGLA

cons                **:* :: *: * ::*.*. ** :***** ****.** *******. * : :    *  ..


Smo140066|PACid     ---GLLILLKERLPAIVRCLSHDSPRIRASSASLLREIVSTDVLRASYP-----GDKAG----
MA_19575g0010       MAEGLWNLLKYRLPATVQCLSHSSAHVRALSTSVLRDILHAESLNFRYCKNF--SEKNHHSEH
LOC_Os01g08700      VADGLSNLLKCRLSTTIRCLSHPSAHVRALSMSVLRDILNSGQINS--SKLIQGEHRNGIQSP
At1g22770           VVDGLSNLLKCRLPATIRCLSHPSAHVRALSTSVLRDIMNQSSIPIKVTPKLPTTEKNGMNSP

cons                   **  *** **.: ::**** *.::** * *:**:*:    :           .:


Smo140066|PACid     ----------AWLEDVEQSIAWETHYRRAEGLSESFLASAAIALGCKLPP-S
MA_19575g0010       LYYGKDIVVQDWNKAVEQCLAWEAHNRQARGMSIALLALAANALGFSAN--V
LOC_Os01g08700      TYQCLAASIINWQADVERCIEWEAHSRRATGLTLAFLTAAAKELGCPLT--C
At1g22770           SYRFFNAASIDWKADIQNCLNWEAHSLLSTTMPTQFLDTAARELGCTISLSQ

cons                           *   ::..: **:*   :  :.  :*  **  **
```
